# CNVrd, a Read-Depth Algorithm for Assigning Copy-Number at the FCGR Locus: Population-Specific Tagging of Copy Number Variation at *FCGR3B*

**DOI:** 10.1371/journal.pone.0063219

**Published:** 2013-04-30

**Authors:** Hoang tan Nguyen, Tony R. Merriman, Michael A. Black

**Affiliations:** 1 Department of Biochemistry, University of Otago, Dunedin, New Zealand; 2 Department of Mathematics and Statistics, University of Otago, Dunedin, New Zealand; Democritus University of Thrace, Greece

## Abstract

The extent of contribution from common gene copy number (CN) variants in human disease is currently unresolved. Part of the reason for this is the technical difficulty in directly measuring CN variation (CNV) using molecular methods, and the lack of single nucleotide polymorphisms (SNPs) that can tag complex CNV that has arisen multiple times on different SNP haplotypes. One CNV locus implicated in human disease is *FCGR*. Here we aimed to use next-generation sequencing (NGS) data from the 1000 Genomes Project to assign CN at *FCGR3A* and *FCGR3B* and to comprehensively assess the ability of SNPs to tag specific CN variants. A read-depth algorithm was developed (CNVrd) and validated on a subset of HapMap samples using CN assignments that had previously been determined using molecular and microarray methods. At 7 out of 9 other complex loci there was >90% concordance with microarray data. However, given that some prior knowledge of CN is required, the generalizability of CNVrd is limited and should be applied to other complex CNV loci with caution. Subsequently, CN was assigned et *FCGR3B* using CNVrd in a total of 952 samples from the 1000 Genomes Project, using three classes and SNPs that correlated with duplication were identified. The best tag SNP was observed in the Mexican-American sample set for duplication at *FCGR3B*. This SNP (rs117435514, r^2^ = 0.79) also tagged similar duplication in Chinese and Japanese (r^2^ = 0.35–0.60), but not in Caucasian or African. No tag SNP for duplication at *FCGR3A* or deletion at *FCGR3B* was identified in any population. We conclude that it is possible to tag CNV at the FCGR locus, but CN and SNPs have to be characterized and correlated on a population-specific basis.

## Introduction

Genomic copy number changes are inherited, de novo and somatically acquired deviations from a diploid state within a particular chromosomal segment. Genome-wide approaches have strongly implicated rare structural genomic variants in common psychiatric disease [1], however the role of higher frequency structural variants in influencing risk of disease is less clear. The primary reason for this has been the technical challenge of using microarrays to accurately measure copy number (CN) on a genome-wide basis over thousands of samples, particularly over the relatively small physical distances affected by common structural variants [2]. At a single locus level, candidate gene studies based on single-probe quantitative polymerase chain reaction analysis with an internal diploid standard have implicated common CN variation (CNV) at a limited number of genes in auto-inflammatory diseases [3]–[12]. However, even these single gene studies are beset by technical challenges [6], [13]–[15], meaning that there is not widespread confidence in these data [16], [17]. Despite these challenges, meta-analysis using studies employing more robust methodologies provides strong evidence supporting a role for *FCGR3B* deletion in systemic autoimmunity [6].

The Wellcome Trust Case-Control Consortium (WTCCC) used a genome-wide custom microarray to assess the impact of approximately 50% of polymorphic CNV larger than 500 base pairs on eight common diseases in the British European Caucasian population [2]. Using 10 probes per locus, with the hybridization intensity compared to a pool of 9 males and 1 female, the Agilent comparative genome hybridization array reliably measured CNV at 3,432 polymorphic loci. Three loci were found to be associated with common disease (*IRGM, HLA, TSPAN8*), although all had previously been implicated via surrogate tagging single nucleotide polymorphisms (SNPs) in SNP-based genome-wide association studies. Based on these results, the WTCCC concluded that common CNVs able to be genotyped on existing platforms are unlikely to be a major factor in the genetic basis of common human disease [2]. Of the 3,432 polymorphic CNVs analyzed, 68% with minor allele frequency (MAF) >0.10 were tagged by SNPs. The WTCCC specifically examined three loci within which CNV had been implicated in auto-inflammatory disease (*CCL3L1, β-defensin, FCGR*), and were unable to replicate previous reports of association with disease [3–5,7], although there was a nominal *P* = 0.058 for association of *CCL3L1* with rheumatoid arthritis, in a direction of association consistent with that previously reported [4]. None of these loci were well tagged by SNPs (best tagging SNP r^2^ = 0.48 for *CCL3L1*, 0.03 for *β-defensin* and 0.01 for *FCGR3B*), suggesting that they involve multiple ancestral recombination events. Based on analysis of NGS data, it has been estimated that at least 11% of all CNV has arisen in this way [18].

When a CNV is well tagged by a common SNP, this indicates that the major variant allele of the CNV is likely to have arisen from a single ancestral recombination event in that population. In the case of a CNV not well tagged by a SNP, it is possible that better tagging SNPs have not yet been identified. It is also possible that multiple ancestral recombination events have occurred on different haplotypic backgrounds, increasing the genomic complexity at this locus, and reducing the ability to tag the variant alleles using a single SNP. At the CCL3L1-CCL4L1 and FCGR loci, for example, where both loci are characterized by closely related paralogous gene pairs, there is structural complexity, with evidence for multiple complex rearrangements [19], [20]. Loci such as these present challenges when analysed genome-wide using an array-based approach [2]. For example, deciding on which probes within a particular locus to use for assigning CN where there are multiple overlapping recombination events (the WTCCC aimed for 10 probes per locus) can lead to difficulties in ensuring that the signal represents the true CN and is not compromised by signal from overlapping events or probes mapping to additional homologous and paralogous loci. The WTCCC acknowledged that careful manual examination and curation of probe-level data can improve on automated procedures but that this was impractical on a genome-wide basis [2]. In the previous candidate gene studies, a single unique probe per locus was typically used. This approach, however would not detect CN changes at other places within the locus of interest.

Given the challenges of measuring CN in complex loci such as *FCGR* and *CCL3L1-CCL4L1* we aimed here to comprehensively investigate the possibility that a SNP tagging approach could be taken in assessing CN. Studying the FCGR locus, with the expectation that multiple ancestral recombination events would underlie CNV, we aimed to use the sequence data from the 1000 Genomes Project, and a new copy number assignation algorithm (CNVrd) to better characterize FCGR structural rearrangements and to determine if tag SNPs exist, particularly in non-Caucasian populations, that could be used to infer CN at this locus.

## Subjects and Methods

### 1000 Genomes Data

Aligned data from the 1000 Genomes Project were downloaded from the European Bioinformatics Institute (EBI) FTP server (ftp://ftp.1000genomes.ebi.ac.uk/vol1/ftp/). Although a number of distinct sequencing platforms have been used to generate sequence data from this project (e.g., Applied Biosystems SOLiD, Roche GS-FLX, Illumina Genome Analyzer), the vast majority of data has been generated using Illumina sequencing platforms. To avoid introducing cross platform variability into the analysis, sequence data was only included if it had been generated on an Illumina platform. As of May 3, 2011 this was 946 low-coverage Illumina samples and 6 high-coverage Illumina samples for which aligned data were available. Alignments of the 1000 Genomes Project data to the chosen reference genome (GRC37.1) are made available via the EBI FTP site, and these alignments were used as the source of data for the work described here. The Illumina data was aligned by the 1000 Genomes Project using the BWA aligner. BWA aligns reads to a reference genome to obtain align-able ‘hits’, for each of which an alignment quality score is calculated [21]. The position with the highest mapping quality is chosen by default and in the case where there are multiple equal best hits then the alignment position is randomly chosen and assigned a quality score of zero. After removing duplicate reads the 952 samples had a total 91,853,700 reads aligned in the 1 Mb region encompassing the FCGR locus. Read length ranged from 26 to 160 bp and mapping quality ranged from 0 to 70, with the median quality score of 60 most common (54.6% frequency) (Figure S1). Figure S2 shows the FCGR positions in the reference genome (GRC37.1).

### Subjects

The populations used were (number of individuals in brackets): ASW, African Ancestry in Southwest USA (50); CEU, (Centre d'Etude du Polymorphisme Humain) from Utah with Northern and Western European ancestry (84); CHB, Han Chinese Beijing (81); CHS, Han Chinese South (92); CLM, Colombian in Medellin (50); FIN, Finnish from Finland (75); GBR, British from England and Scotland (70); IBS, Iberian Spanish (6); JPT, Japanese from Tokyo (78); LWK, Luhya in Webuye, Kenya (83); MXL, Mexican Ancestry in Los Angeles (54); TSI, Toscani in Italy (98); YRI, Yoruba in Ibadan, Nigeria (79). In September 2012 a further 428 samples were downloaded, specifically to examine tagging of *FCGR3B* duplication in Asian and American populations: ACB, African Carribbeans in Barbados (64); CDX, Chinese Dai in Xishuangbanna, China (88); GIH, Gujarati Indian from Houston, Texas (78); KHV, Kinh in Ho Chi Minh City, Vietnam (78); PEL, Peruvians from Lima, Peru (49); IBS, Iberian population in Spain (71).

### Determination of Copy Number: CNVrd

Samtools [22] was used to extract reads mapping to a one megabase region around the FCGR locus (1000 Genomes Project co-ordinates - Chr 1: 161,100,000–162,100,000) to produce a BAM file of reads in this region for each of the total 1380 (428+932) samples. All duplicate reads were removed using the “rmdup” option in Samtools. In order to obtain an estimate of CN in this region a read-depth approach was used, with the region divided into a collection of non-overlapping 1000 base-pair windows (1000 windows in total). For each sample, the number of reads whose start site fell within each window was calculated, producing a set of 1000 counts for each sample. GC-content adjustment was performed using the method of Yoon et al. [23], prior to transforming the read counts for each sample by dividing by the sample median, to allow comparison across samples having different levels of sequencing coverage. No filtering based on mapping quality was used, with all reads mapped to this region used for estimating CN, thus maximizing the available coverage. The counts were further transformed on a per-window basis across samples, by standardizing each window to have a mean of 0 and a standard deviation of 1.

The standardized data were analyzed using the DNAcopy package [24] within Bioconductor [25]. Although this package was primarily designed for detecting CNV in microarray-based data, the data generated via the “window” approach is essentially identical to that expected from a high-density tiling array of this region, with the standardized count from each window analogous to a microarray probe intensity. DNAcopy segmented the standardardized counts into contiguous sections with similar signal intensities, whereupon the regions including *FCGR3A* and *FCGR3B* were extracted and segmentation results were used to assign CN status for each sample at each of the loci. When a CN event (gain or loss) spanned the entire gene locus, the continuous-valued DNAcopy output was used as the segmentation score for that gene, however when the CN event did not span the entire locus (e.g., gain or loss of only part of the gene), the estimate was assigned a segmentation score of zero. The segmentation scores for each sample at the two loci were then used as data in a normal mixture model containing three components relating to deletion, normal CN (CN = 2) or duplication for each of *FCGR3A* and *FCGR3B*. Prior knowledge of CN distribution at the FCGR locus was used to select the three-component model. A custom R function was used to fit the normal mixture model to the data. Based on the output of the segmentation step, at *FCGR3A*, segmentation scores which were less than −1.5 or larger than 2.1 were concluded to be deletions and duplications, respectively and at the FCGR3B locus, scores which were less than −2 or larger than 1.8 were concluded to be deletions and duplications, respectively. At both loci trimmed scores were then grouped into three classes by a three-component normal mixture model with equal variances. To ensure reliable convergence of the log-likelihood function, the “kmeans” function in R was first used to cluster the two vectors (the segmentation scores at each of the loci) into three groups, with the group centroids used as initial values for the normal mixture model (Figure S3).

As a comparative read-depth based method we also used the CNVnator software [18] to determine CN status of the 952 samples downloaded in May 2011, with the 1000 Genomes Project data divided into three groups according to average depth of coverage; <4, ≥4 and <6, ≥6. CNVnator was run with bin size 500 base pairs for the first group, 200 for the second and 100 for the third, as recommended by the developers of CNVnator (pers. comm. Alexej Abyzov).

**Figure 1:**
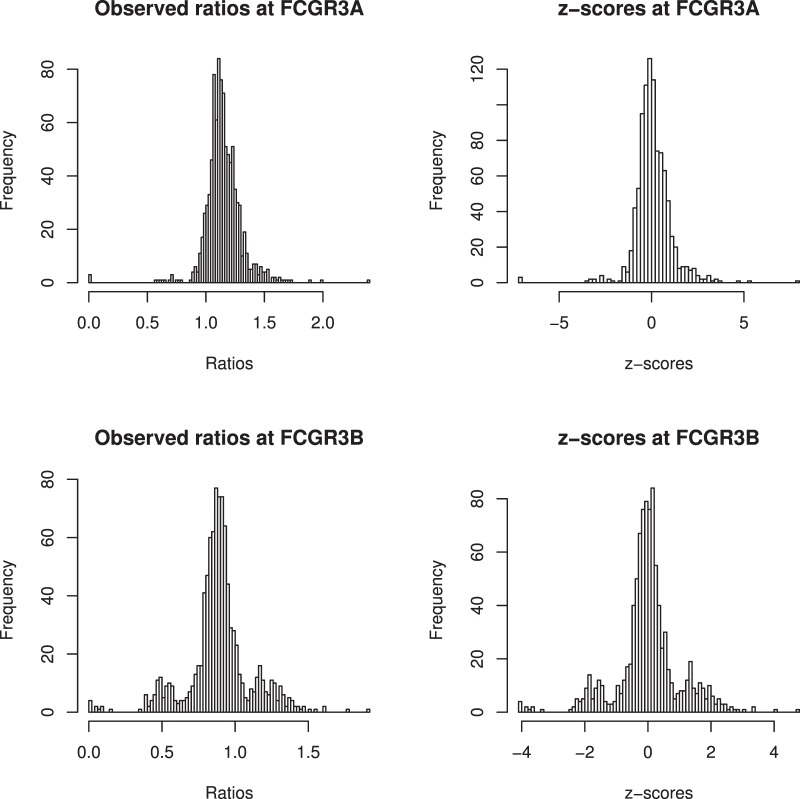
The frequency of real ratios of observed and expected read counts. The left graphs depict observed ratios while the right graphs depict z-scores (transformed ratios).

To further investigate CN at *FCGR3A* a standardized z-score was calculated (Figure 1). The ratio of observed read counts within each of *FCGR3A* and *FCGR3B* and expected read counts (ERC) was calculated, where ERC = (h*k)/M, and h = length of segment, k = the number of reads in the 1 Mb region for that sample, and M = 1 Mb. Z-scores were calculated for each window as (sample ratio minus median sample ratio for all 952 samples) divided by the standard deviation for all 952 samples.

### SNP Identification and CN Correlation

The Variant Call Format (VCF) [26] files for chromosome 1 from the 1000 Genomes Project (low coverage individuals, ftp://ftp.1000genomes.ebi.ac.uk/vol1/ftp/release/20101123/interim_phase1_release/, and high coverage individuals, ftp://ftp.1000genomes.ebi.ac.uk/vol1/ftp/pilot_data/release/2010_07/trio/snps/) were downloaded, Tabix [27] used to extract a 2 Mb region (161,000,000–163,000,000) from the VCF file, followed by VCFtools to extract genotypes of all relevant individuals. Given the need to correctly identify SNPs as genuine polymorphisms rather than paralagous sequence variants [28] we evaluated whether SNPs of interest were genuine polymorphisms (Methods S1 and Table S1) – on this basis SNP *rs117435514* was concluded to be a genuine SNP. Samtools [22] was used to call SNPs for the 428 samples downloaded in September 2012. For each SNP, Fishers Exact Test [29] was used to assess association between the presence of the SNP, and CN status at *FCGR3A* and *FCGR3B*. This was done for duplications and deletions separately, with each analysis focusing on four groups of samples, based on whether or not there was a CN difference at each locus (e.g., for normal (N) and duplicated (D) CN at each locus, the four possible 3A/3B copy number groups are: N/N, N/D, D/N, D/D). For deletion, because there were few deletions at *FCGR3A* we divided the data into two groups: normal (N) at both loci and deleted (D) at *FCGR3B*. P-values were adjusted for multiple testing using the False Discovery Rate controlling method [30]. To calculate the Spearman correlation between SNPs and CN status, we coded the major allele homozygote as 0, the heterozygote as 1 and the minor allele homozygote as 2; duplications at both *FCGR3A/3B* were coded as 4, duplication only at *FCGR3B* or *FCGR3A* as 3, 2 respectively, and normal copy number at both loci as 1. In the deletion analysis, deletion at *FCGR3B* was coded as 2, and normal as 1.

## Results

### Validation of Methodology

**Figure 2:**
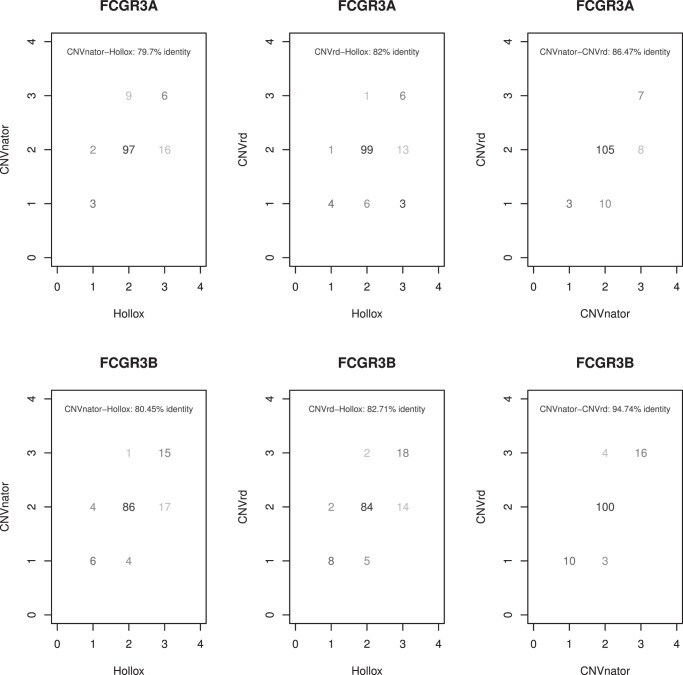
Concordance of the three methods: Hollox et al. (14), CNVnator and CNVrd. 0/1 is deletion, 2 is CN = 2 and 3/4 is duplication.

As an initial validation of this methodology we analyzed the 133 samples (127 low coverage and 6 high coverage) sequenced as part of the 1000 Genomes Project that had also been analyzed for FCGR locus copy number using an integrated approach involving five molecular assays [14]. Twenty-seven of these samples subsequently had CN assigned [11] using an approach modified from Hollox et al. [14]. Our approach (termed ‘CNV read depth’ (CNVrd)) was applied, along with the read depth approach implemented in the CNVnator software [18], and compared to the published CN data [11], [14] (Table S2). Agreement at *FCGR3B* was 82.7% between our results and those of Hollox et al. [14], 94.7% between our approach and CNVnator and 80.5% between CNVnator and Hollox et al. results (Table 1; Figure 2). A similar pattern was seen at *FCGR3A*, with 82.0%, concordance between our results and those of Hollox et al. [14], 79.7% agreement between CNVnator and Hollox et al. [14], and 86.5% agreement between CNVnator and our approach (Table 1, Figure 2). Discordant traces are presented in Figure S4, and consensus CN assignments are presented in Table S2. At both genes there was 100% concordance between CNVrd and the Robinson et al. [11] data, with 92.6% identity at *FCGR3A* and 100% at *FCGR3B* observed using CNVnator. The sequence between 161.4–161.5 Mb with increased read-depth was identified, using RepeatMasker (http://www.repeatmasker.org/cgi-bin/WEBRepeatMasker), to have GC-content of 64% and consist of repetitive LINE1 sequence, LTR elements and interspersed repeats.

**Table 1:** Genotype frequencies in HapMap sample sets.

	ASW	CEU	CHB	CHS	CLM	FIN	GBR	IBS	JPT	LWK	MXL	PUR	TSI	YRI	ACB *	CDX*	IBS*	GIH*	KHV*	PEL*
FCGR3A																				
Del	0 (0%)	1 (1.2%)	2 (2.5%)	0 (0%)	1 (2%)	0 (0%)	0 (0%)	0 (0%)	0 (0%)	1 (1.2%)	0 (0%)	0 (0%)	1 (1%)	1 (1.3%)	0 (0%)	2 (2.3%)	1 (1.4%)	2 (2.6%)	1 (1.3%)	0 (0%)
Normal	44(88%)	71(84.5%)	61(75.3%)	77(83.7%)	43(86%)	71(94.7%)	62(88.6%)	6(100%)	62(79.5%)	68(81.9%)	44(81.5%)	47(90.4%)	91(92.9%)	75(94.9%)	1(1.6%)	81(92%)	65(91.5%)	73(93.6%)	75(96.2%)	47(95.9%)
Dup	6(12%)	12(14.3%)	18(22.2%)	15(16.3%)	6(12%)	4(5.3%)	8(11.4%)	0(0%)	16(20.5%)	14(16.9%)	10(18.5%)	5(9.6%)	6(6.1%)	3(3.8%)	63(98.4%)	5(5.7%)	5(7%)	3(3.8%)	2(2.6%)	2(4.1%)
FCGR3B																				
Del	6(12%)	5(6%)	11(13.6%)	4(4.3%)	8(16%)	4(5.3%)	9(12.9%)	1(16.7%)	5(6.4%)	9(10.8%)	3(5.6%)	6(11.5%)	10(10.2%)	13(16.5%)	17(26.6%)	10(11.4%)	5(7%)	11(14.1%)	11(14.1%)	2(4.1%)
Normal	36(72%)	69(82.1%)	51(63%)	70(76.1%)	34(68%)	68(90.7%)	54(77.1%)	5(83.3%)	59(75.6%)	62(74.7%)	38(70.4%)	45(86.5%)	83(84.7%)	62(78.5%)	44(68.8%)	74(84.1%)	61(85.9%)	55(70.5%)	59(75.6%)	26(53.1%)
Dup	8(16%)	10(11.9%)	19(23.5%)	18(19.6%)	8(16%)	3(4%)	7(10%)	0(0%)	14(17.9%)	12(14.5%)	13(24.1%)	1(1.9%)	5(5.1%)	4(5.1%)	3(4.7%)	4(4.5%)	5(7%)	12(15.4%)	8(10.3%)	21(42.9%)

(*are 1000 Genomes samples downloaded in September 2012).

Copy number assignments were also compared to data from microarray-based approaches. Combined *FCGR3A* and *FCGR3B* copy number was derived from locus CNVR383.1 of the Affymetrix SNP 6.0 array assayed over 143 HapMap samples 31 also analyzed here and also from an oligonucleotide-tiling array assayed over 231 HapMap samples [32] also analyzed in common. CNVrd had correlations r^2^ = 0.83 and 0.82 with each of the microarray-based studies, compared to CNVnator having r^2^ = 0.77 and 0.72, respectively.

### Assigning Copy-number in the 1000 Genomes Project

**Figure 3:**
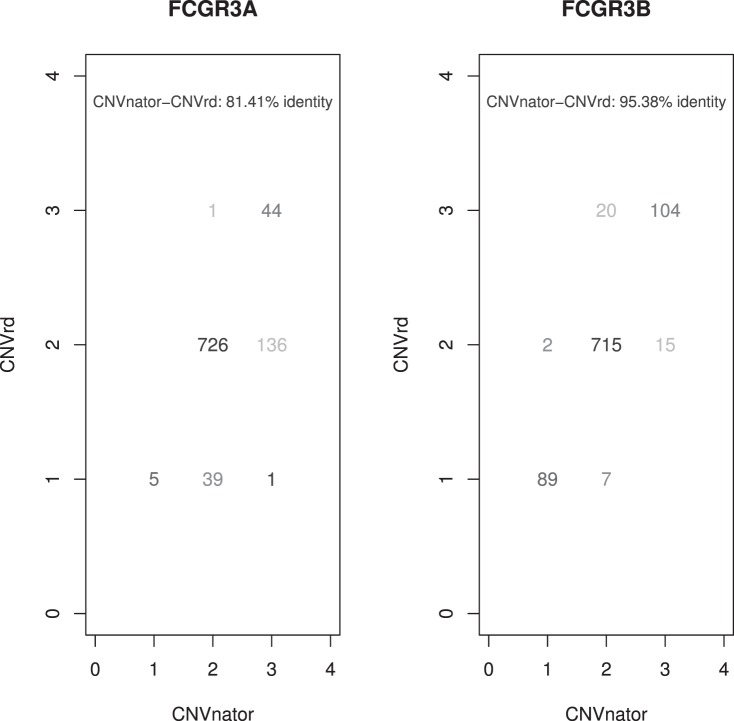
Concordance between CNVnator and CNVrd using the 952 1000 Genomes Project samples. 0/1 is deletion, 2 is CN = 2 and 3/4 is duplication.

**Figure 4:**
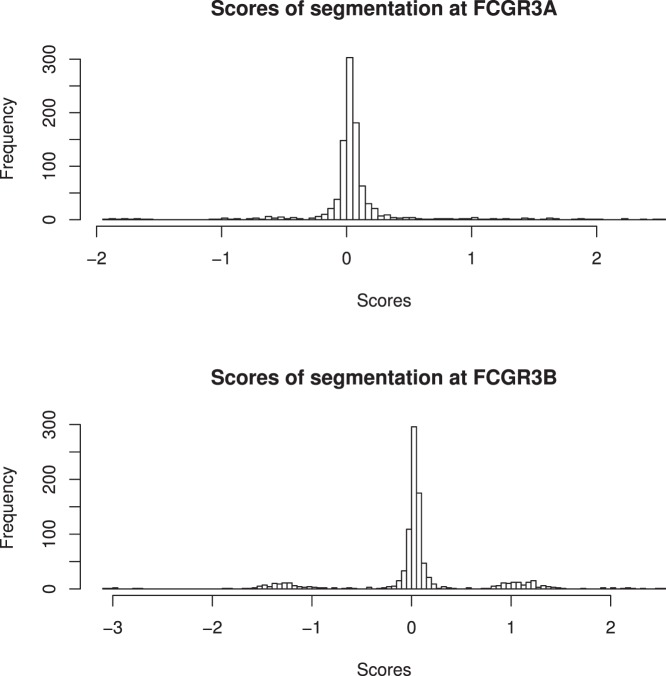
Segmentation scores at *FCGR3A* and *FCGR3B* derived from DNAcopy (23).

Based on the good level of agreement between the NGS read-depth-based copy number assessments (CNVnator and CNVrd) these methods were next applied to all 952 samples downloaded May 2011 (Figure 3). Once again, there was better concordance between CNVrd and CNVnator at *FCGR3B* (95.4%) than at *FCGR3A* (81.4%). Traces of read depth plots (before and after standardizing by windows) of samples with CN assignments discrepant between the two methods were compared and CN was assigned by visual inspection. At *FCGR3A* after visual inspection 65.0% of the discordant samples had the same assignment as CNVnator and 35.0% the same assignment as CNVrd, and at *FCGR3B* 36.4% had the same assignment as CNVnator and 63.6% the same assignment as CNVrd. The final CN distributions for *FCGR3A* and *FCGR3B* are presented in Table 1 and Figure 4. There was better clustering of CN at *FCGR3B* than *FCGR3A.* Deletion at *FCGR3A* was not as frequent as at *FCGR3B*, with duplication tending to be more frequent in South East Asian (CHB/CHS/JPT) sample sets.

### Identification of Tag SNPs

In order to identify possible tagging SNPs, duplication at the FCGR locus was first divided into four classes: no duplication at either *FCGR3A* or *FCGR3B* (CN = 2, class N), duplication at *FCGR3A* alone (class A), duplication at *FCGR3B* alone (class B) and duplication at both *FCGR3A* and *FCGR3B* (class AB). Individuals with deletion at either locus were excluded. Association between SNPs present in the 2.0 Mb region surrounding the FCGR locus and the three classes of duplication was evaluated in all 14 populations. Significant results were only obtained in four populations: MXL, JPT, CHB, and CHS (Table S3). The best tagging SNP for duplication at *FCGR3B* was observed in the Mexican American (MXL) population, with the minor allele of *rs117435514* present nearly exclusively on chromosomes with duplication at *FCGR3B* (*P = *6.9×10^−8^; r^2^ = 0.79), and only one individual (NA19785) having normal CN in the presence of the minor allele (verified by visual inspection). This SNP also showed a similar correlation with duplication at *FCGR3B* in the Han Chinese and Japanese samples (CHB, CHS, JPT; *P = *7.8×10^−6^ (r^2^ = 0.46), 1.5×10^−9^ (r^2^ = 0.49) and 2.9×10^−6^ (r^2^ = 0.60), respectively), although there were 9 (3.6%) normal copy number individuals with the minor allele, and six (2.4%) Han Chinese AB individuals with the major allele. SNP rs117435514 was the most correlated in MXL, CHB and CHS, however rs81045784 (and variants in linkage disequilibrium) was more strongly correlated in JPT (*P* = 3.1×10^−7^; r^2^ = 0.60); this SNP was also strongly correlated in CHB and CHS (*P*<5×10^−5^; r^2^ = 0.41 and 0.44, respectively) but weakly correlated in MXL (*P = *0.03; r^2^ = 0.29). As in the MXL samples, there was complete correlation in CHB/CHS/JPT between the minor allele of *rs117435514*/*rs81045784* and presence of a single duplication at *FCGR3B.* This variant was not correlated with duplication in African sample sets (*P*>0.05), although it was polymorphic in African (YRI MAF = 0.02). The SNP was monomorphic in Caucasian sample sets. We then examined other non-Caucasian and non-African populations, CLM (Colombian in Medellin) and PUR (Puerto Rican in Puerto Rico). In CLM the tag SNP (rs117435514) was the 11^th^ most associated with *FCGR3B* duplication (*P* = 0.44, r^2^ = 0.30) – there was one normal copy number individual with the minor allele and three with duplication at *FCGR3B* without the minor allele. The minor allele of *rs117435514* was not present in the PUR samples. Using a second set of 1000 Genomes data *rs117435514* was also observed as the strongest tag SNP for duplication in a third Chinese data set (CDX, *P* = 0.06, r^2^ = 0.36) (Table S4). Tagging of duplication at *FCGR3B* was also observed in one further South East Asian (KHV; *P* = 0.01, r^2^ = 0.36) and one further American (PEL; *P* = 5×10^−5^, r^2^ = 0.63) data set but not in a South Asian (GIH) or further African (ACB) and Caucasian (IBS) data sets (Table S4). Finally, tagging of duplication at *FCGR3B* was also observed in the CHB and JPT sample sets when we used data generated using methods not based on next generation sequencing (Methods S1).

In MXL, CHS, CHB and JPT SNP rs117435514 appeared to tag two distinct ancestral recombination events – duplication at *FCGR3B* alone and duplication at *FCGR3A* and *FCGR3B*. Given the biological implausibility of this we investigated the possibility in MXL that these groups represent a single recombination event, and the implausible results were caused by incorrect CN assignment. Initially, the read-depth graphs of the 13 MXL individuals with copy number increase at *FCGR3B* were visually inspected (Figure S5). This revealed that one MXL sample (NA19717) may have been duplicated at both *FCGR3A/3B* but *FCGR3A* duplication had not been called by either of CNVnator or CNVrd. Observing data not standardized by windows for this sample and four others called CN = 2 at *FCGR3A* (NA19651, NA19731, NA19749, NA19756), we noticed that the difference of depth of coverage between *FCGR3A* and its left region occurred very close to the boundary of the gene (Figure S5). Suspecting that segmentation was problematic in the event of tandem duplication where breakpoints occurred close to the gene boundary we calculated standardized ratios of observed and expected reads expressed as z-scores on the total 25 samples from CHB, CHS, JPT and MXL which were called CN = 2 at *FCGR3A* and CN = 3 at *FCGR3B* and were positive for the minor allele at *rs117435514* (Figure S6). All 25 samples had high z-scores at *FCGR3B* (>1.04), however z-scores were more variable at *FCGR3A* (ranging from −0.63 to 1.91), with six below zero and five above one (Table S5). Given that the 25 samples are highly likely to be CN = 3 at *FCGR3B*, the five samples with z-score >1 at *FCGR3A* are likely to have CN = 3 at this locus. The z-score distributions at each locus (Figure 1) are similar to the segmentation-based CN distributions (Figure 4).

For duplication at *FCGR3A* only there were no significant tag SNPs in any population. Hollox et al. [14] reported rs10800032 as the SNP in the FCGR region best able to tag any of HNA1 genotype (HNA1a-c comprising three antigens expressed by neutrophils on CD16 (FcGR)), total copy number, or *FCGR3A/FCGR3B* copy number – it tagged HNA1 genotype in Japanese at r^2^ = 0.38 (r^2^ was 0.06 in Caucasian, 0.003 in Chinese and was monomorphic in Yoruban). This SNP was not correlated with copy number in any population in our data (maximal r^2^ was 0.07 in Japanese).

For deletions (Table S3), because there were relatively few individuals exhibiting deletions at FCGR3A (0.74%; 7/952; 1 in each of CEU, CLM, LWK, TSI, YRI and 2 in CHB)), the samples were divided into two groups, A (normal copy number at both the FCGR3A and FCGR3B loci) and B (deletions at the FCGR3B locus), excluding samples with deletion at *FCGR3A* only and samples with duplication at either *FCGR3A* and/or *FCGR3B*. The best tagging SNP was *rs12076636* (P = 8.2×10^−4^, r^2^ = 0.75) in the LWK (Luhya in Webuye, Kenya) population, with no copy number normal samples (group A) and 77.8% of the group B samples having the minor allele of the SNP. However, this SNP did not tag *FCGR3B* deletion in any other population. Significant, albeit weak, correlations, were also found in the TSI (Toscani in Italy) samples: the minor allele of a group of SNPs (marked by rs61802308 and rs2002405) was present in most normal samples (81% and 96%, respectively) but at a lower frequency in most *FCGR3B* deleted samples (10% and 40%, respectively) (*P* = 0.008, r^2^ = 0.16 and *P* = 0.008, r^2^ = 0.05, respectively). However, there was no evidence that either of these SNPs tagged *FCGR3B* deletion in any other population.

### FCGR2

Based on the high concordances between CNVrd and array and molecular-based methods at the FCGR3A and FCGR3B locus, we used CNVrd to measure copy number counts of the FCGR2A/2B/2C genes (Figure S7). Segmentation scores at *FCGR2A* and *FCGR2B* indicated little or no CN variation with few values deviating from 0, consistent with previous data based on molecular methods [19]. At the FCGR2C locus, segmentation scores had clusters similar to that at the FCGR3B locus (Figure 1). There was a high correlation (90%) between CN assignments at *FCGR3B* and *FCGR2C* (Figure S8). As expected because of the correlation in CN between *FCGR2C* and *FCGR3A/FCGR3B* the best tagSNP for duplication at *FCGR2C* was *rs117435514* in four populations CHB (p = 3.51×10^−03^, r^2^ = 0.35), CHS (p = 2.17×10^−03^, r^2^ = 0.32), JPT (p = 2.2×10^−04^, r^2^ = 0.47) and MXL (p = 1.03×10^−02^, r^2^ = 0.48). There were no significant tag SNPs for deletion at *FCGR2C.*

### Application of CNVrd to other Common CNV Loci

**Figure 5:**
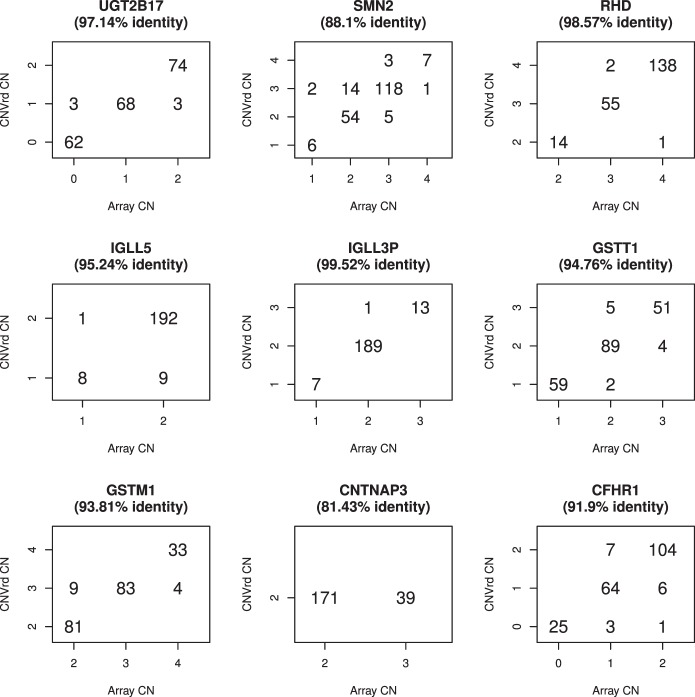
Concordance between CNVrd and microarray calls (35) for 9 CNV loci. The x-axis is microarray CN assignments (35) and the y-axis CNVrd CN assignments. No clear CNVrd clusters were seen for *CNTNAP3* or *IGLL5.* Window sizes were: *RHD* (2000 bp), *UGT2B17* (50 0 bp), *GSTT1* (1000 bp), *IGLL3P* (1000 bp), *SMN2* (1000 bp), *GSTM1* (200 bp), *CFHR1* (500 bp), *CNTNAP3* (2000 bp) and *IGLL5* (1000 bp).

CNVrd was developed here as a tool to test for the existence of tag SNPs at the FCGR locus. To further test this method it was applied to nine other complex CN loci for which CN assignments were available. Twenty-one CNV genes implicated in disease were identified from Table 1 of Armour et al. [33]. Of these, nine had previously had CN count measured by a high-resolution microarray method [34]. BAM files representing one megabase of sequence data flanking these genes were downloaded for 210 samples that overlapped with the 1000 Genomes sequencing data. CNVrd was applied with varying window sizes (0.2, 0.5, 1.0, 2.0, 5.0 and 10.0 kilobase pairs) owing to variation in gene length, with the final window size chosen so as to minimize the occurrence of segmentation scores of zero. Normal mixture models with different numbers of components were used to group segmentation scores into CN count distributions (Figure 5). Concordance rates were >90% for 8 of the 9 loci, however there were 2 genes (*CNTNAP3* and *IGLL5*) for which no clear clusters of CN groups were observed, and there was a high rate of segmentation scores of zero. Visual inspection of the read depth traces indicated that CNVrd tended to divide the CNTNAP3 and IGLL5 gene regions into different segments, possibly indicating altered CN for only part of the gene sequence. In situations involving CN changes for less than the entire gene, CNVrd automatically produces a segmentation score of zero for that sample, effectively assigning a value equal to the most common CN for that population.

## Discussion

Here we developed an algorithm (CNVrd) to assign CN at the *FCGR* locus from NGS data for the purpose of investigating the ‘taggability’ of CNV. The discovery of a SNP that is highly correlated with duplication at *FCGR3B* in several populations that have shared ancestry validates our approach. This SNP (*rs117435514*) will likely be useful in these populations to assay duplication at *FCGR3B.* However, the wide use of tag SNPs to genotype copy-number variation at the FCGR locus is unlikely to be universally useful; we were unable to identify tags for duplication in any Caucasian, South Asian or African populations, consistent with previous reports [2], [14], suggesting that the duplication events happened at multiple times in the respective population histories, in contrast to the major duplication event we discovered in South East Asian (CHB, CHS, JPT, CDX, KHV) and American (MXL, PEL) populations.

CNVrd and CNVnator were applied to a defined locus using a large amount of low coverage NGS whole genome data. There was good correlation between the NGS read-depth-based methods and CN assignments generated using molecular biological techniques ([14]; 80–83% at each of *FCGR3A* and *FCGR3B*: [11]; 100% at *FCGR3A* and *FCGR3B*). In comparison with microarray-based methods, the NGS based methods obtained good concordance (over r^2^>0.7 and r^2^>0.8 for CNVnator and CNVrd, respectively). Our estimates of genotype frequency of NGS-derived CN estimates were also very similar to those estimated using non-qPCR-based molecular biological approaches (summarized in [11]): at *FCGR3A* we documented a deletion frequency of approximately one percent in most populations with duplication more frequent (3.8–22.2%); at *FCGR3B* deletion was more common (4.3–16.5%) than at *FCGR3A* and duplication was also common (1.9%–24.1%). It is not possible to precisely quantitate how accurate our method is and to benchmark against other NGS CN assignment approaches because the CN of test samples is not known, only inferred by other methods. There are a number of other algorithms that have been developed for detection and assignment of CN from NGS data. Two other methods have been published for absolute assignment of CN from NGS data, mrCaNaVaR and CopySeq [35], [36]. However because the 1000 Genomes Project data were generated using different read lengths and BAM files were produced using different aligners (BWA, BFATS, MOSAIK) the other methods are less suitable for the FCGR locus than our method or CNVnator. CopySeq was designed to use equivalent read lengths - although it can be applied to data of different read lengths it is not as robust in repetitive regions because it does not use alignments with a quality score of zero. mrCaNaVaR was designed to use mrFAST or mrsFAST as the aligner, although other aligners could be used provided all map locations are reported (not the case for BWA, which reports only the best alignment by default – the option used for the 1000 Genomes Project alignments that were used here). Other methods (including cn.MOPs, RDXplorer, JointSLM, CNV-Seq) designed for CNV detection on a genome-wide basis from NGS data, generally suffer from a high false discovery rate (with the exception of cn.MOPS) especially in low coverage NGS data, as discussed in a recent review [37].

Given that deletion at *FCGR3B* is strongly implicated in autoimmune disease etiology [6] the lack of identification of a tag SNP here does not provide an immediate solution for association analysis of FCGR3B CN with disease in most populations. One other CN determination approach may be the identification of exact structural rearrangement breakpoints, the presence of which can be directly assayed in genomic DNA using similar methodologies as those employed for SNP genotyping. Success of this approach relies on having the ability to accurately detect the exact position of DNA breakpoints, as well as being able to associate each breakpoint position with a distinct CN genotype (e.g., multiple independent ancestral events may have resulted in distinct FCGR duplications and deletions, each associated with a different DNA breakpoint location). At the FCGR locus, this approach is made difficult by the amount of paralogous sequence, as the high degree of sequence similarity leads to some reads mapping ambiguously across the region. To overcome this challenge, algorithms that are able to account for both mapping ambiguity, and the occurrence of multiple ancestral duplication and deletion events will be required for reliable breakpoint-based genotyping of CN.

At *rs117435514* it was observed that the minor allele tagged 25 individuals with duplication of *FCGR3B* alone (class B), in addition to 33 individuals with a duplication of *FCGR3B* concurrently with duplication at *FCGR3A* (class AB) in JPT/CHB/CHS/MXL. This was unexpected, as we hypothesized that duplication classes B and AB would represent separate recombination events and thus would have distinct tag SNPs. One possibility is that individuals with the minor allele at *rs117435514* and duplication at *FCGR3B* but not *FCGR3A* may have duplication at *FCGR3A* and *FCGR3B* on one chromosome and deletion at *FCGR3A* on the other chromosome. Currently there is no way of identifying this genotype combination. Alternatively there may be a technical reason, for example in the 1000 Genomes Project alignments that were used in this study, the mapping quality threshold may have been too low, allowing reads from *FCGR3A* to map to *FCGR3B.* However multiplex ligation-dependent probe amplification (MLPA) assays to the FCGR2A-C, FCGR3A,B and HSPA6,7 genes (all within the FCGR locus) have been used to demonstrate the presence of duplication at *FCGR3B* alone and duplication at both *FCGR3A* and *FCGR3B* in Caucasian samples (10% frequency for *FCGR3B* alone and 0.8% for both *FCGR3A* and *FCGR3B*) [19], suggesting that the separate AB and B classes we detected using NGS do exist (although we detected the frequency of the AB class (duplication at *FCGR3A* and *FCGR3B*) to be higher at 7/84 (8.33%) in Caucasian). Whatever the phenomenon that underlies the tagging of both class B and AB duplications by *rs117435514*, it is likely to have hampered identification of tag SNPs for duplication at *FCGR3A*. We were also hampered by power, as the individual sample sets were small and structural rearrangement events infrequent, typically <10%. To further investigate tag SNPs for deletion in other populations, our data at the FCGR locus do suggest that larger better-powered population-specific sample sets with NGS data are required.

At *FCGR3A* in particular we encountered discrepancy between our method and CNVnator in CN assignment (Figure 1). In order to understand why this was the case, we examined the average levels of sequencing coverage across the FCGR3A region. In general, it was found that *FCGR3A* tended to have higher coverage than would be expected (Figure 1; real ratios at *FCGR3A* are >1), relative to the surrounding sequence. As a consequence, when samples are viewed individually, there appears to be an increase in coverage (and thus an increase in copy number could be inferred) at *FCGR3A*. If read counts are standardized across samples, however, this phenomenon does not occur. CNVnator does not standardize across samples, and is therefore more prone to interpreting increased coverage at *FCGR3A* as a CN change. The use of standardization, however, makes the assumption that the majority of individuals under analysis are CN = 2 at each locus, and if this is not the case, then subsequent copy number calls will be unreliable. The fact that most of the samples analyzed by Hollox et al. [14] were CN = 2 at *FCGR3A* provides strong support for the use of cross-sample standardization. We therefore believe that the disagreement between methodologies at *FCGR3A* is largely due to the coverage issue, and that comparing across samples (including cross-sample standardization) at the locus is the most appropriate way to deal with this.

There are some limitations to CNVrd. Firstly, it was designed to be applied to specific loci for which there is some prior knowledge of CN distribution, so that the segmentation scores can be arranged into suitable groups by using model-based clustering methods or heuristic thresholds. In some situations (e.g., *CNTNAP3* and *IGLL5* in Figure 5) sharp changes in segmentation scores can indicate CN differences in a sub-region of a gene. As our interest here is in making CN calls for an entire gene, CNVrd has been designed to assign a segmentation score of 0 when this occurs. Because of the standardization across samples used by CNVrd, a score of zero is equivalent to an “average” CN (i.e., the most common CN value in the population). In some cases, this effect is due to low sequencing coverage of the region of interest, and can be alleviated by selection of a larger window size. For this reason we recommend using CNVrd with a number of window sizes, and selecting that which results in the lowest number of samples with segmentation scores of zero. When the most common CN is not 2, CNVrd can incorrectly assign CN, for example *CNTNAP3* (Figure 5). One possible solution is to apply CNVrd to non-standardized data across samples to gain some prior knowledge of the copy-number status of a gene region. This, however, is only reasonable for simple CNV regions, because there are some complex regions where read counts are inherently different. For example, the upstream region of *FCGR3A* has read counts higher than other regions in the 1 Mb region, which can be accounted for by standardizing across samples. Secondly, CNVrd has not been evaluated on data sets generated using next-generation sequencing platforms and data sets other than Illumina-generated 1000 Genomes data. Finally, because CNVrd was designed specifically for complex CN loci, it is not currently applicable to genome-wide analysis, although it should be possible to modify the procedure for multiple loci if prior knowledge is available. Our analysis of 9 other loci revealed high concordance and good clustering at 7, however this number is too small to make any conclusions about the generalizability of CNVrd. This method should be applied to other regions of the genome with caution.

Our analysis of NGS data at the FCGR locus was in agreement with previous findings, namely that tag SNPs were not readily detected in most populations, including Caucasian and African, consistent with increased diversity of structural rearrangements. However, before concluding that tag SNPs for duplication and deletion at the FCGR locus do not exist, NGS data from larger population-specific data sets needs to be analyzed, in addition to the development of algorithms for accurate breakpoint detection at complex CN loci that can relate CN to specific structural rearrangement events.

### Code

Our analysis pipeline, CNVrd, runs on Linux, is released under the GPLv2 license, and is available at http://code.google.com/p/cnvrdfortagsnps/.

## Supporting Information

Figure S1 Top panel: Frequency of mapping quality (top) and read lengths (bottom) of the 91,853,700 reads mapped to the one Mb region in the 952 samples. Bottom panel: Frequency of mapping quality of reads aligned to the FCGR3A and FCGR3B genes. At the FCGR3A and FCGR3B gene (bottom panel), the rates of reads having mapping quality of 0 were higher than the average rate (18.7%, top panel), 22.8% (199776/876520) and 27.9% (191726/686374) respectively. Moreover, the most reliable mapping quality (60) in these two genes were both below 30% (25.8% and 22.4%, respectively) while the average rate of read having quality of 60 was approximately 54.6% in the entire 1 Mb region. The lower mapping quality in *FCGR3A* and *FCGR3B* occurs because there were reads from the two genes which had at least two hits (one from 3A and one from 3B) when they were aligned; as a result one alignment was randomly chosen and a quality score of zero was assigned by the BWA aligner used by the 1000 Genomes Project.
(PDF)

Figure S2 The FCGR locus on chr1:161479500–161650000 (hg19). The bottom picture depicts the genes and duplicating segmentations (yellow: 98–99% similarity and gray: 90–98% similarity) obtained from the UCSC Genome Browser. The top picture depicts r^2^-Spearman CN-SNP correlation values in four populations MXL, JPT, CHS and CHB. Y axis is r^2^ values while the x axis is the coordinates in base pairs. SNP rs117435514 maps 9617 bp downstream of *FCGR3B*, however we could not determine whether or not *rs117435514* was within the CNV region.
(PDF)

Figure S3 Segmentation scores (x-axis) and three classes of copy number variations, y-axis (deletion: 1, normality: 2, duplication: 3) at *FCGR3A* (above) and *FCGR3B* (below).
(PDF)

Figure S4 Traces of discordant samples between the three methods. On the left are the traces before standardizing across samples and on the right are the traces after standardizing across samples. 3a = position of *FCGR3A* and 3b = position of *FCGR3B.* The green horizontal lines are the mean values of regions from the step segmentation of the DNAcopy package. The file is Supplemental Figure 4.pdf.
(PDF)

Figure S5 The 13 MXL individuals with *FCGR3B* duplication. The green horizontal lines are the mean values of regions from the step segmentation of the DNAcopy package. The file is Supplemental Figure 5a.pdf.
(PDF)

Figure S6 The 25 MXL/CHB/CHS/JPT individuals CN = 2 at *FCGR3A*, CN = 3 at *FCGR3B* with minor allele of *rs117435514*. The green horizontal lines are the mean values of regions from the step segmentation of the DNAcopy package. The file is Supplemental Figure 5b.pdf.
(PDF)

Figure S7 Segmentation scores at *FCGR2A, FCGR2B* and *FCGR2C* derived from DNAcopy.
(PDF)

Figure S8 Concordance between CNVrd CN assignments at *FCGR2C* and *FCGR3B.* 0/1 is deletion, 2 is CN = 2 and 3/4 is duplication.
(PDF)

Table S1 (DOC)

Table S2 Copy number of the 1000 Genomes Project samples analyzed by Hollox, CNVrd, CNVnator at *FCGR3A* and *FCGR3B (1: deletion, 2: normal, 3: duplication).*
(DOC)

Table S3 Correlation of SNPs with copy number in the FCGR locus (A = *FCRG3A, B = FCGR3B*).
(DOC)

Table S4 Correlation of duplication at *FCGR3B* with SNPs in data sets downloaded from 1000 Genomes in September 2012.
(DOC)

Table S5 Standardized ratios of observed and expected reads at MXL, CHB, CHS and JPT samples called CN = 2 at *FCGR3A* and CN = 3 at *FCGR3B.*
(DOC)

Methods S1 (DOC)
