# Supplementary figures and images for: CNVrd, a Read-Depth Algorithm for Assigning Copy-Number at the FCGR Locus: Population-Specific Tagging of Copy Number Variation at FCGR3B
Source: PLoS One. 2013 Apr 30;8(4):e63219. doi: 10.1371/journal.pone.0063219 (PMC3640002; doi:10.1371/journal.pone.0063219)

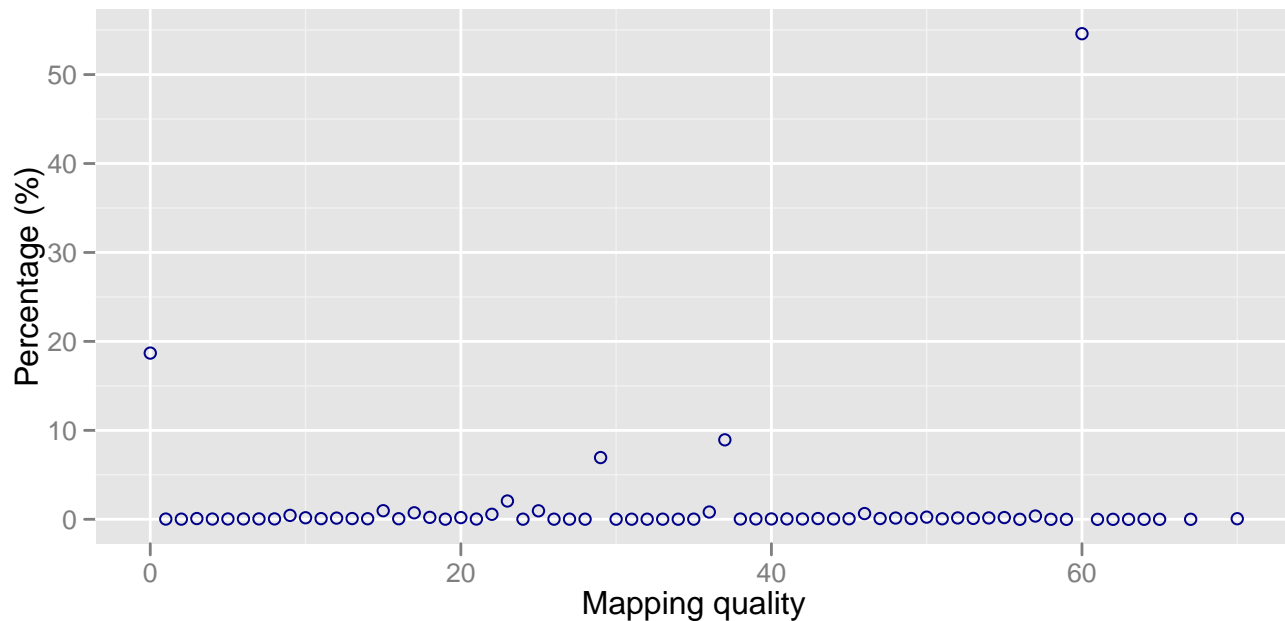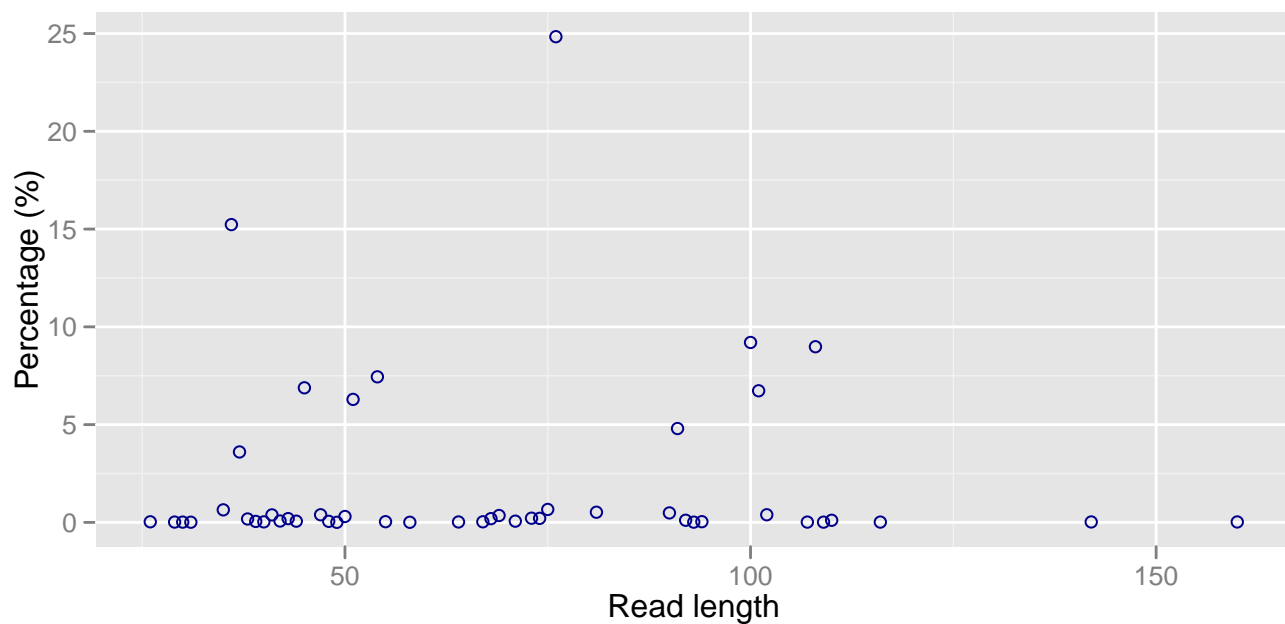

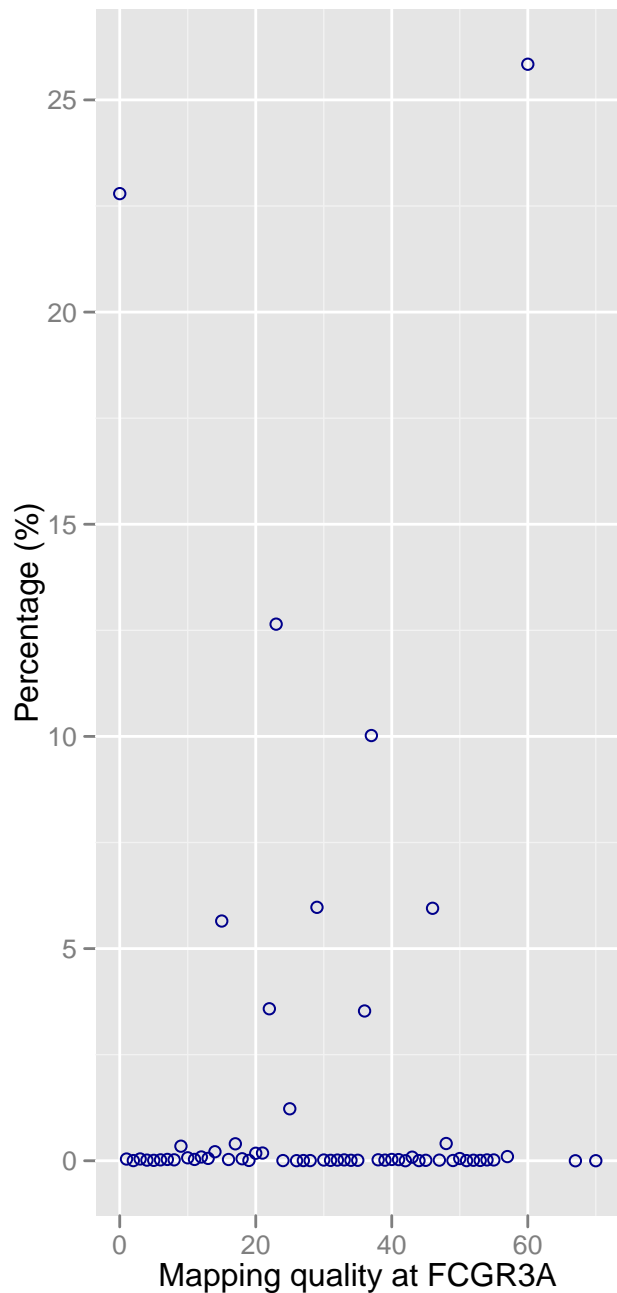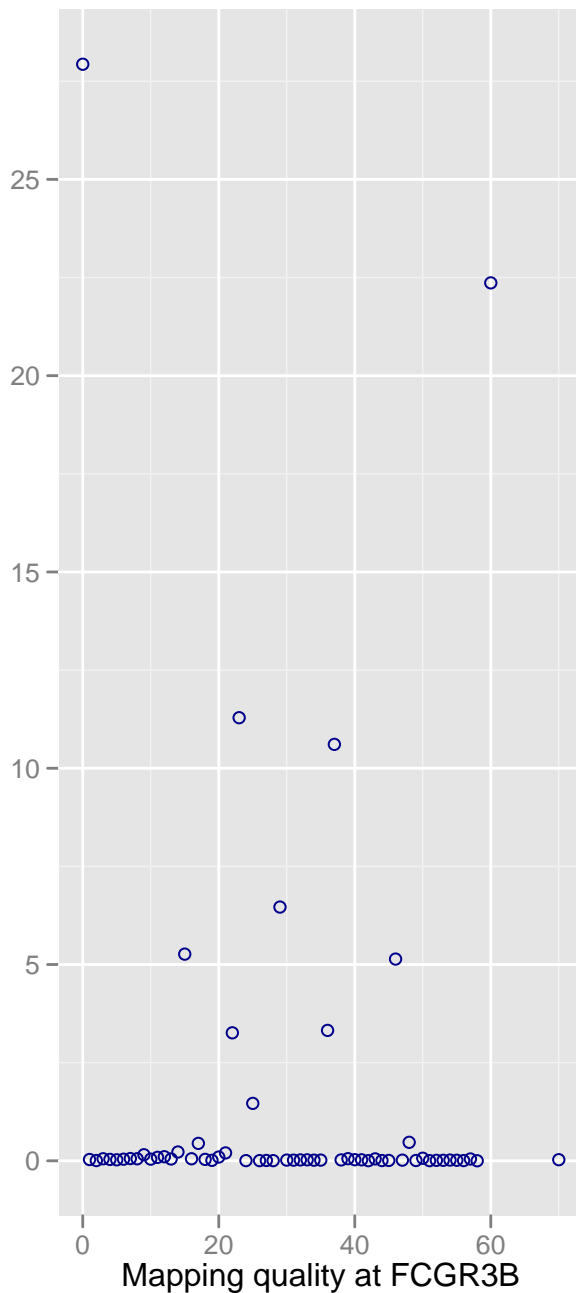

Supplement: Figure S1 — Top panel: Frequency of mapping quality (top) and read lengths (bottom) of the 91,853,700 reads mapped to the one Mb region in the 952 samples. Bottom panel: Frequency of mapping quality of reads aligned to the FCGR3A and FCGR3B genes. At the FCGR3A and FCGR3B gene (bottom panel), the rates of reads having mapping quality of 0 were higher than the average rate (18.7%, top panel), 22.8% (199776/876520) and 27.9% (191726/686374) respectively. Moreover, the most reliable mapping quality (60) in these two genes were both below 30% (25.8% and 22.4%, respectively) while the average rate of read having quality of 60 was approximately 54.6% in the entire 1 Mb region. The lower mapping quality in FCGR3A and FCGR3B occurs because there were reads from the two genes which had at least two hits (one from 3A and one from 3B) when they were aligned; as a result one alignment was randomly chosen and a quality score of zero was assigned by the BWA aligner used by the 1000 Genomes Project. (PDF) [file pone.0063219.s001.pdf]

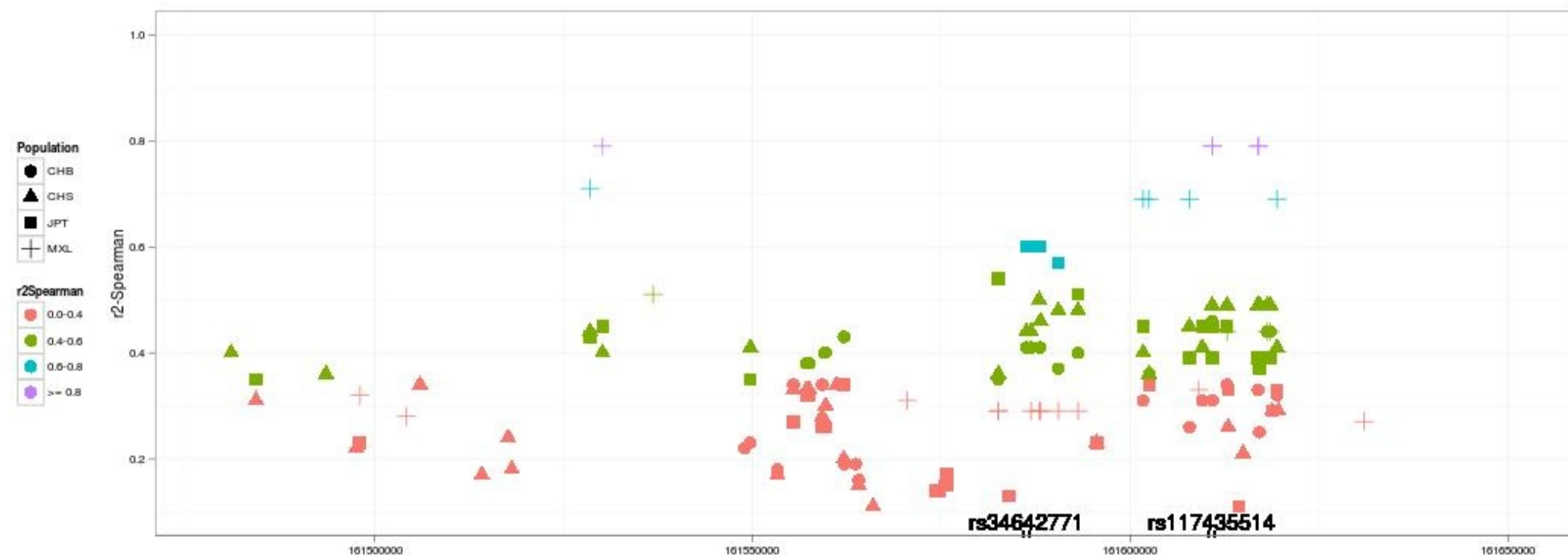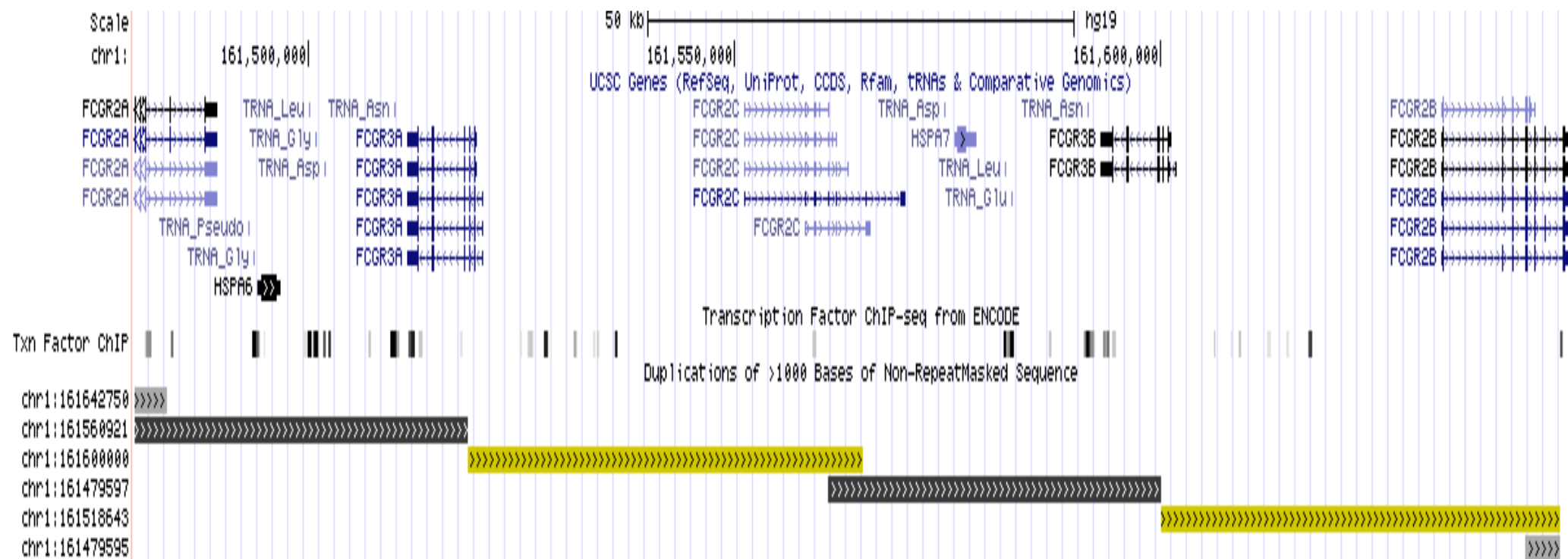

Supplement: Figure S2 — The FCGR locus on chr1:161479500–161650000 (hg19). The bottom picture depicts the genes and duplicating segmentations (yellow: 98–99% similarity and gray: 90–98% similarity) obtained from the UCSC Genome Browser. The top picture depicts r2-Spearman CN-SNP correlation values in four populations MXL, JPT, CHS and CHB. Y axis is r2 values while the x axis is the coordinates in base pairs. SNP rs117435514 maps 9617 bp downstream of FCGR3B, however we could not determine whether or not rs117435514 was within the CNV region. (PDF) [file pone.0063219.s002.pdf]

## FCGR3A

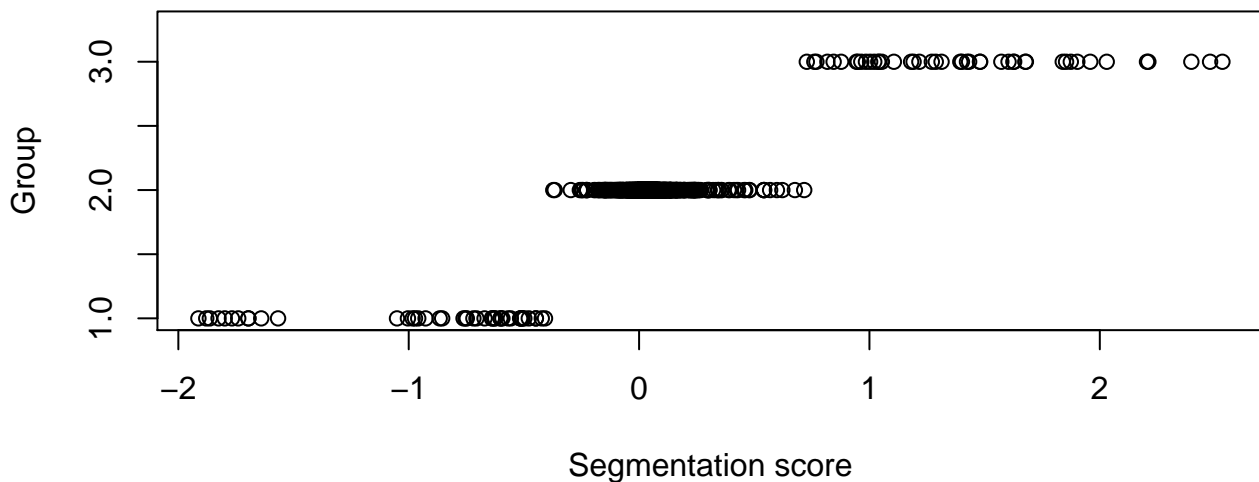

## FCGR3B

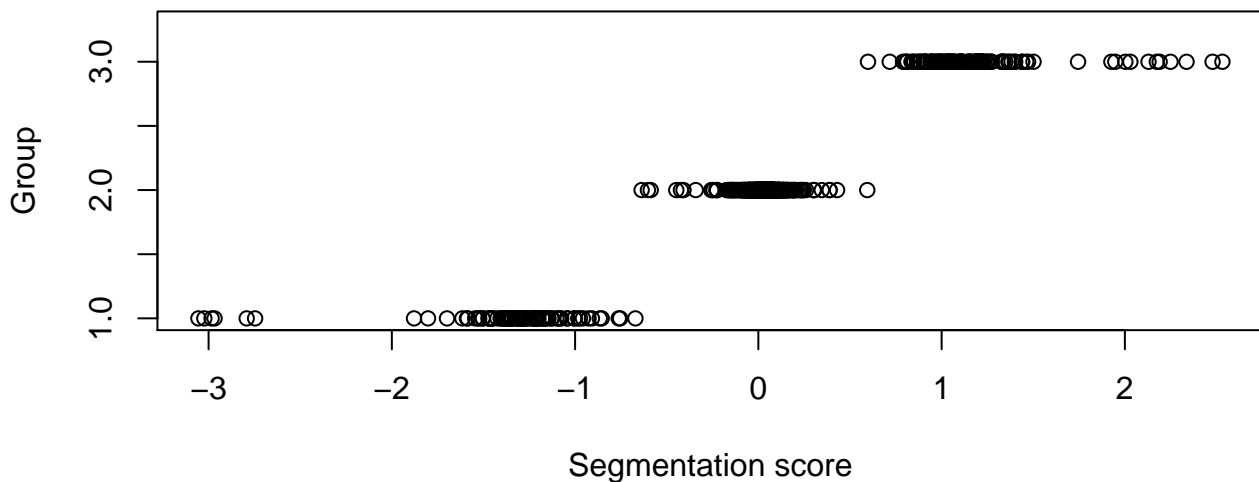

Supplement: Figure S3 — Segmentation scores (x-axis) and three classes of copy number variations, y-axis (deletion: 1, normality: 2, duplication: 3) at FCGR3A (above) and FCGR3B (below). (PDF) [file pone.0063219.s003.pdf]

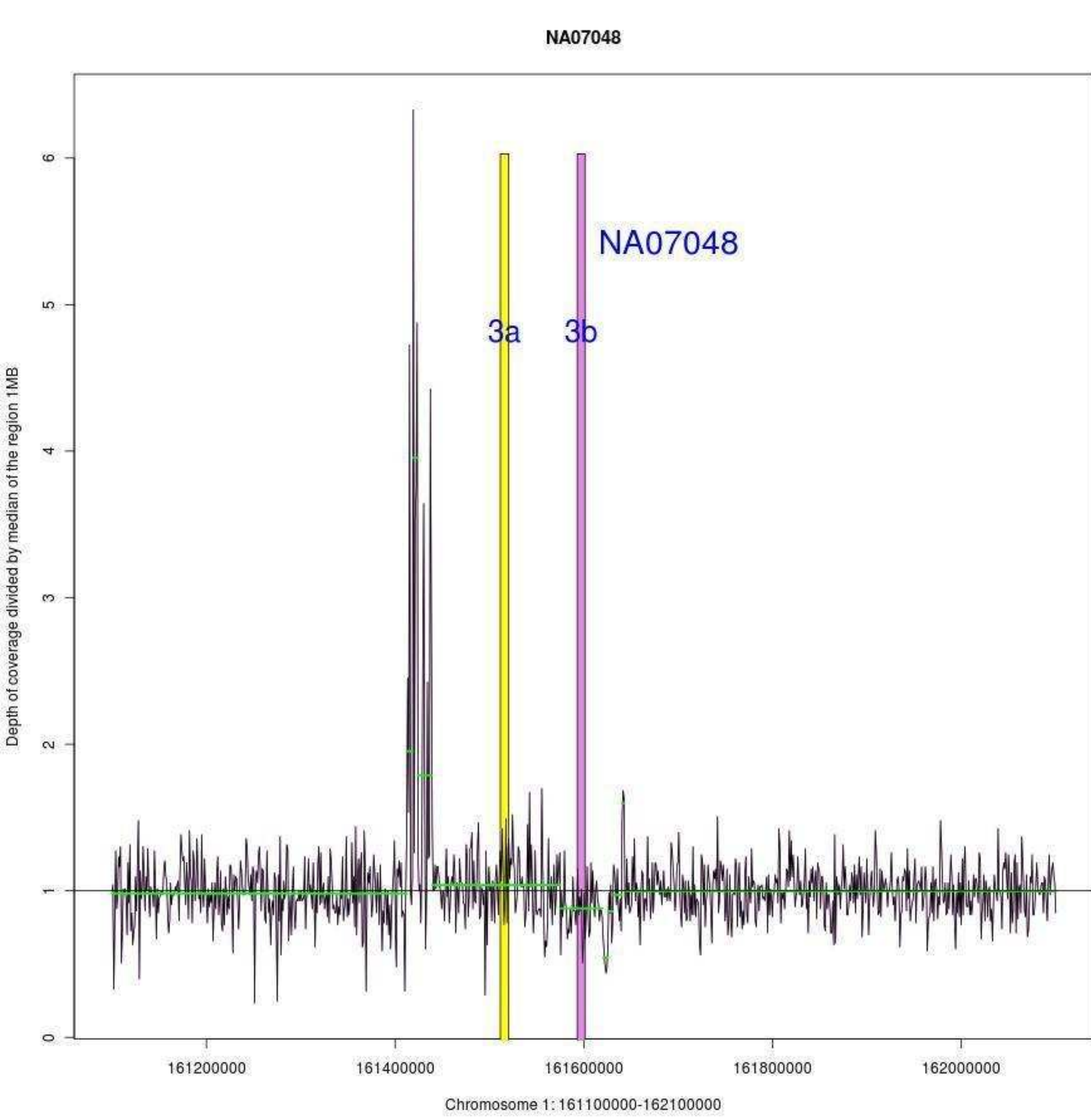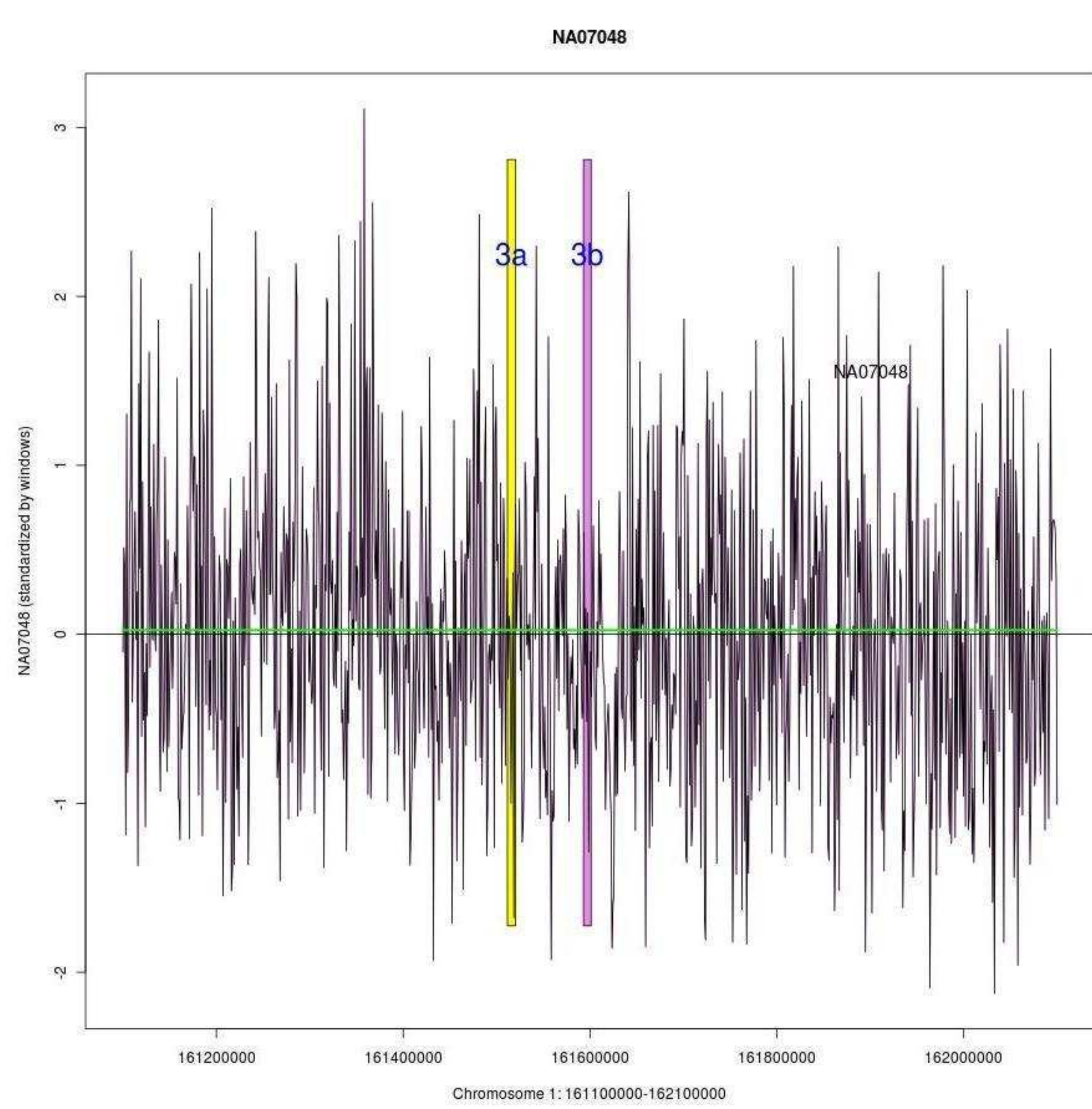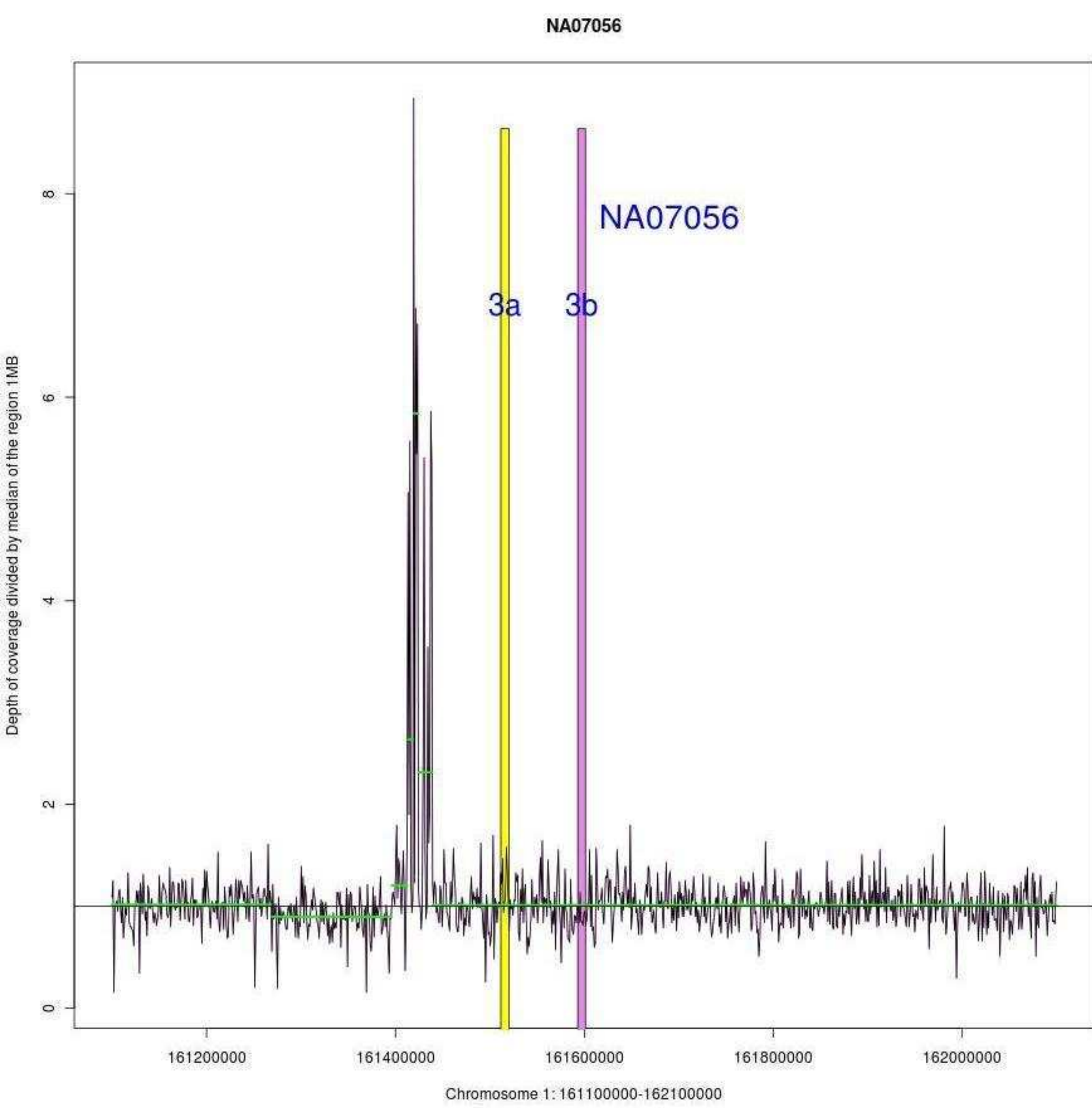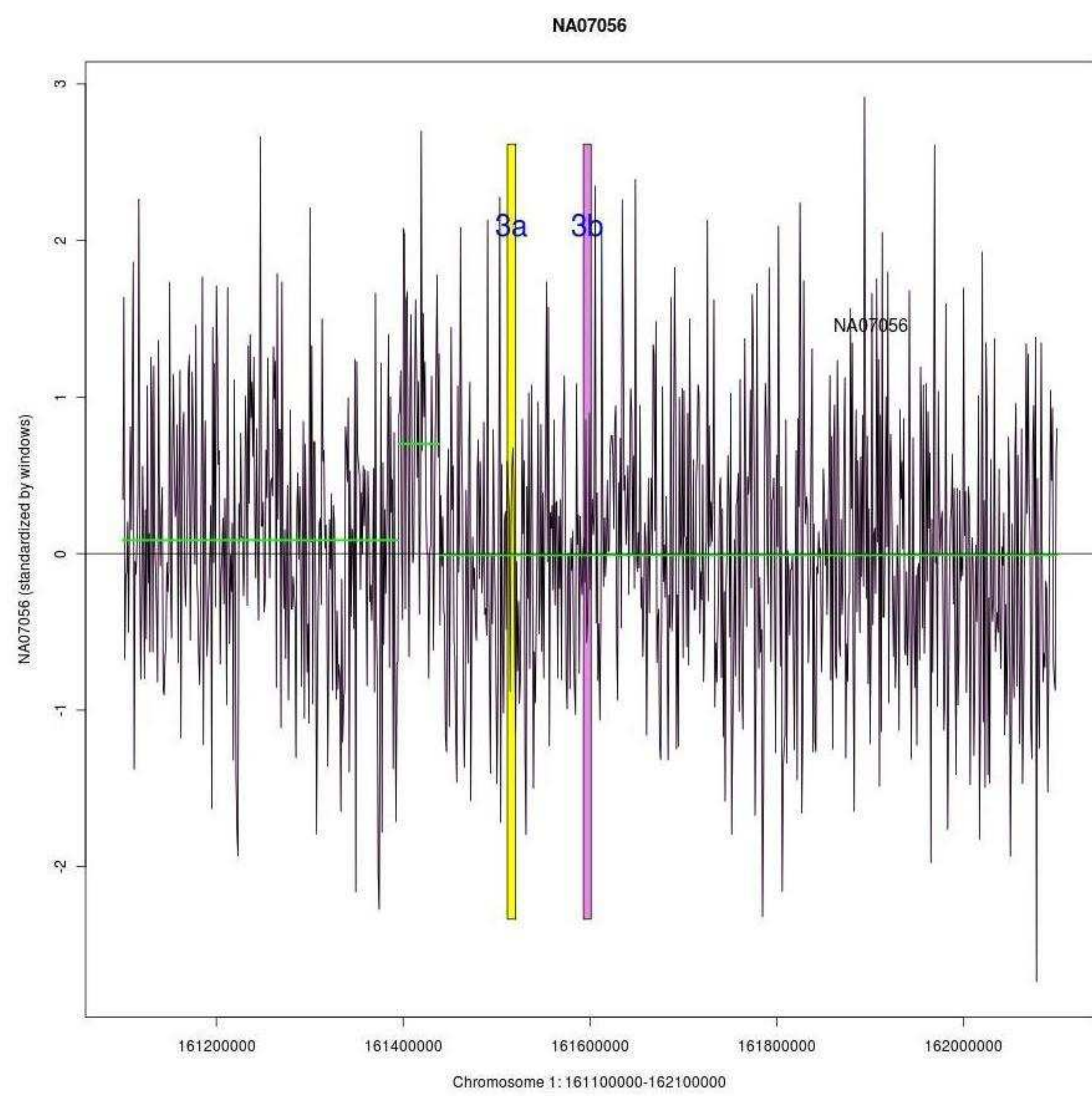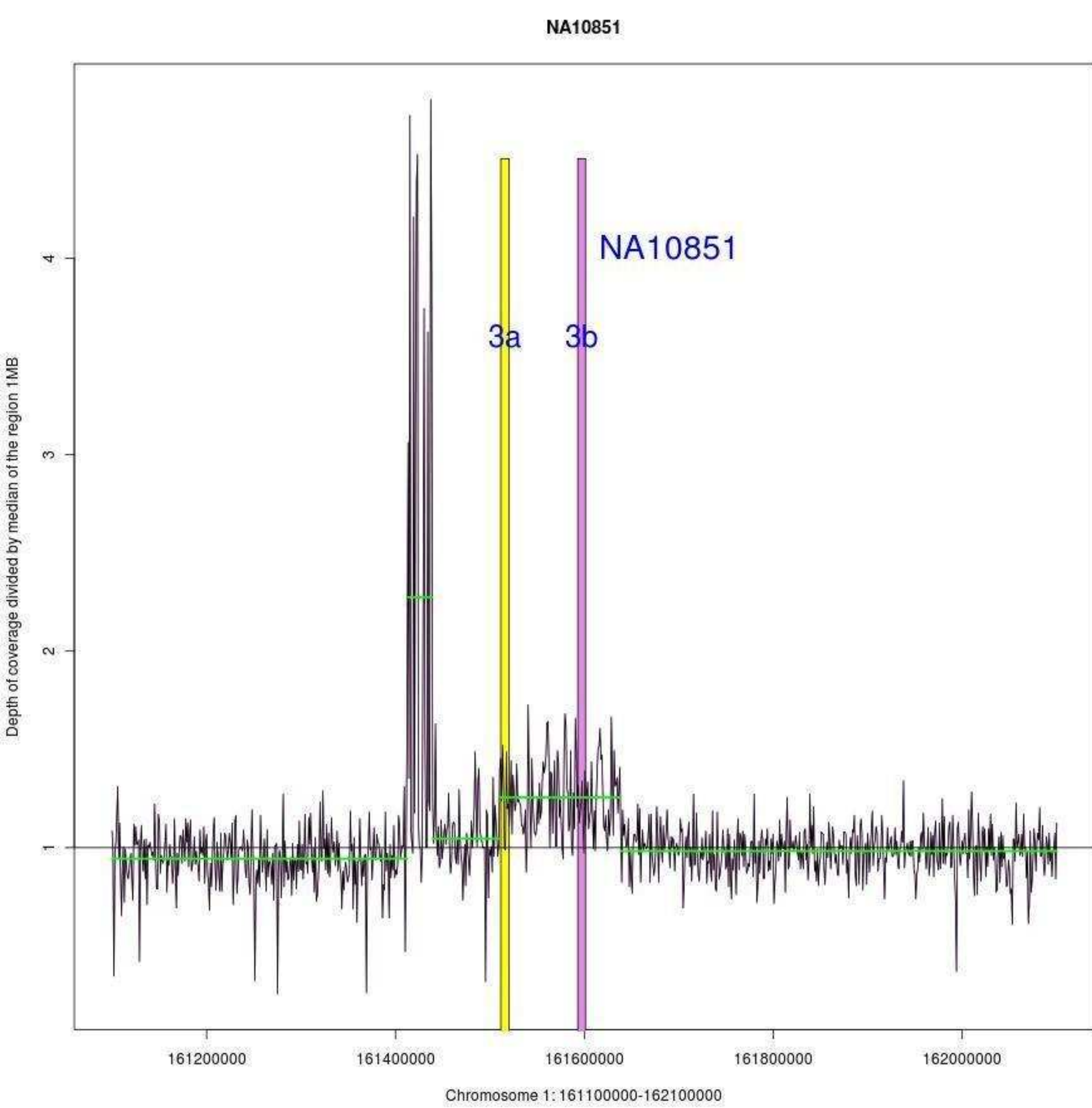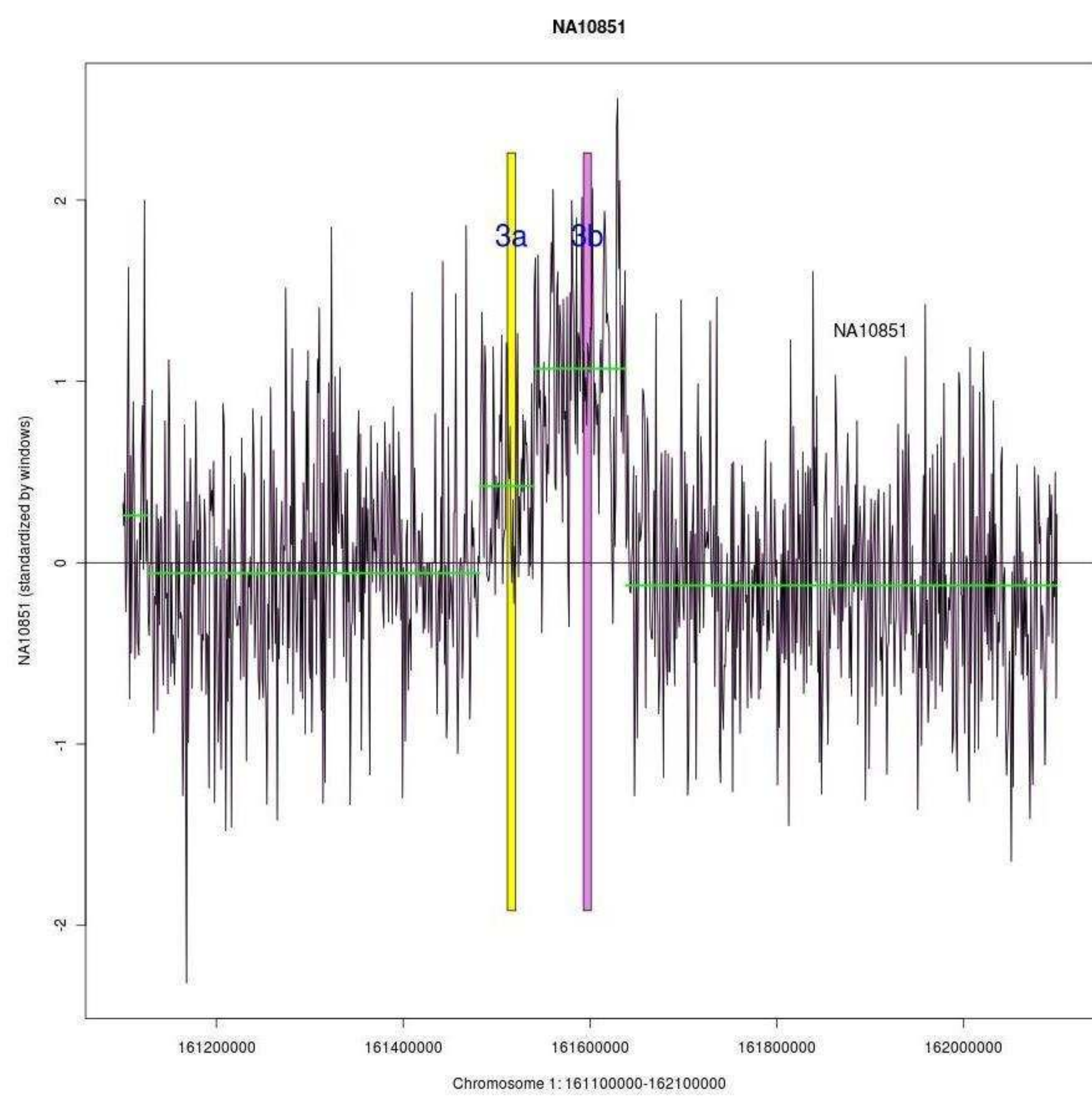

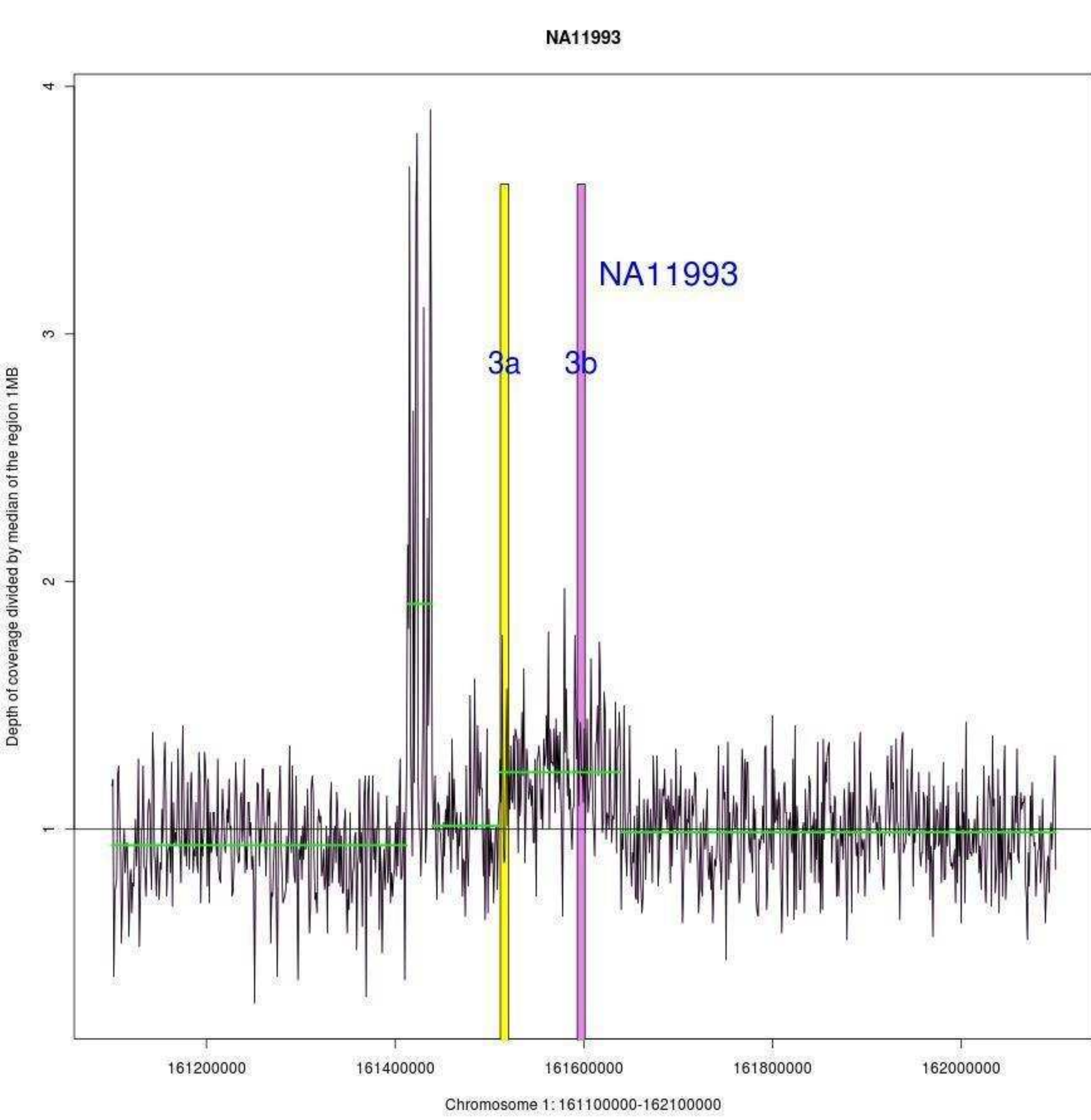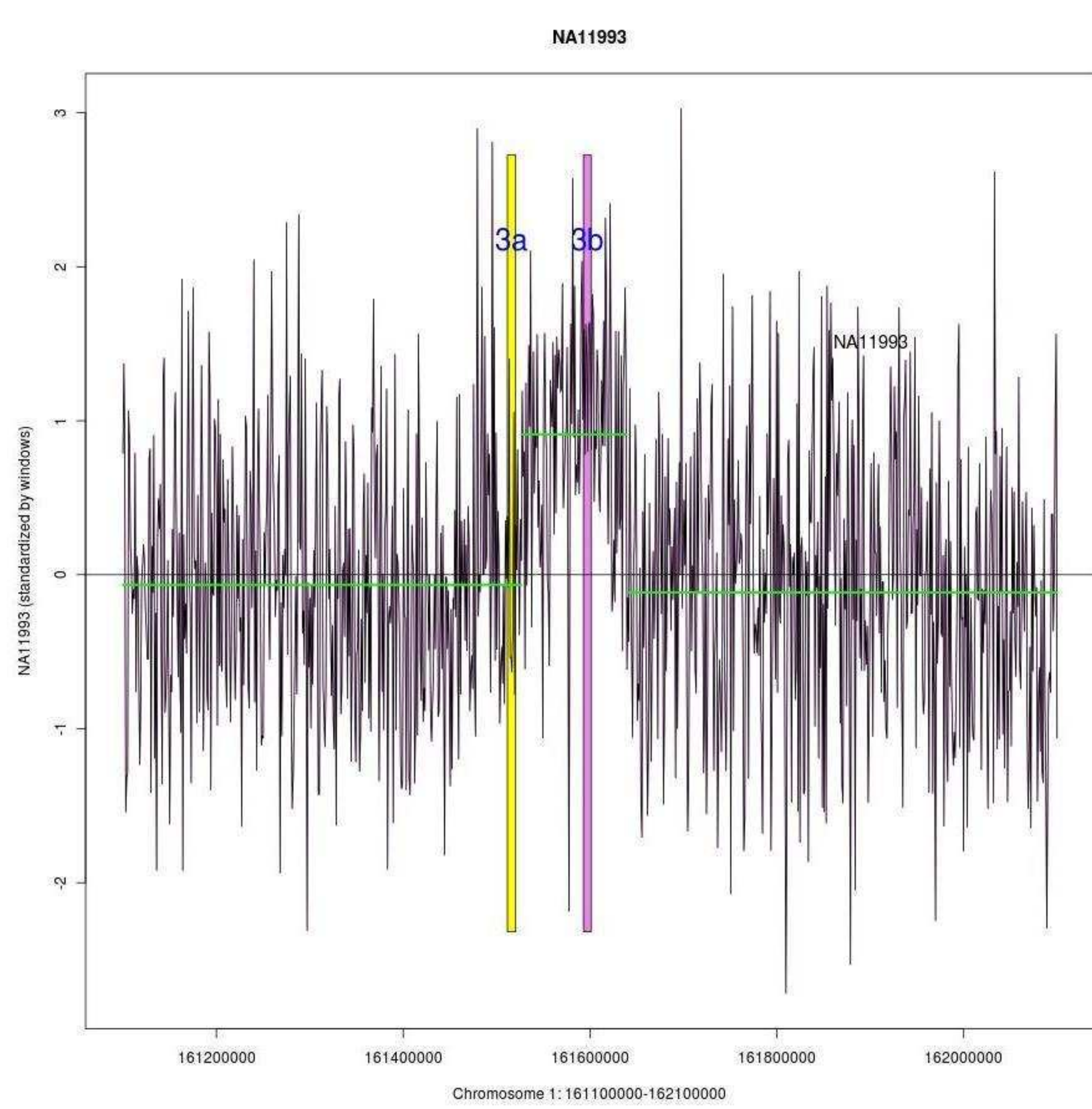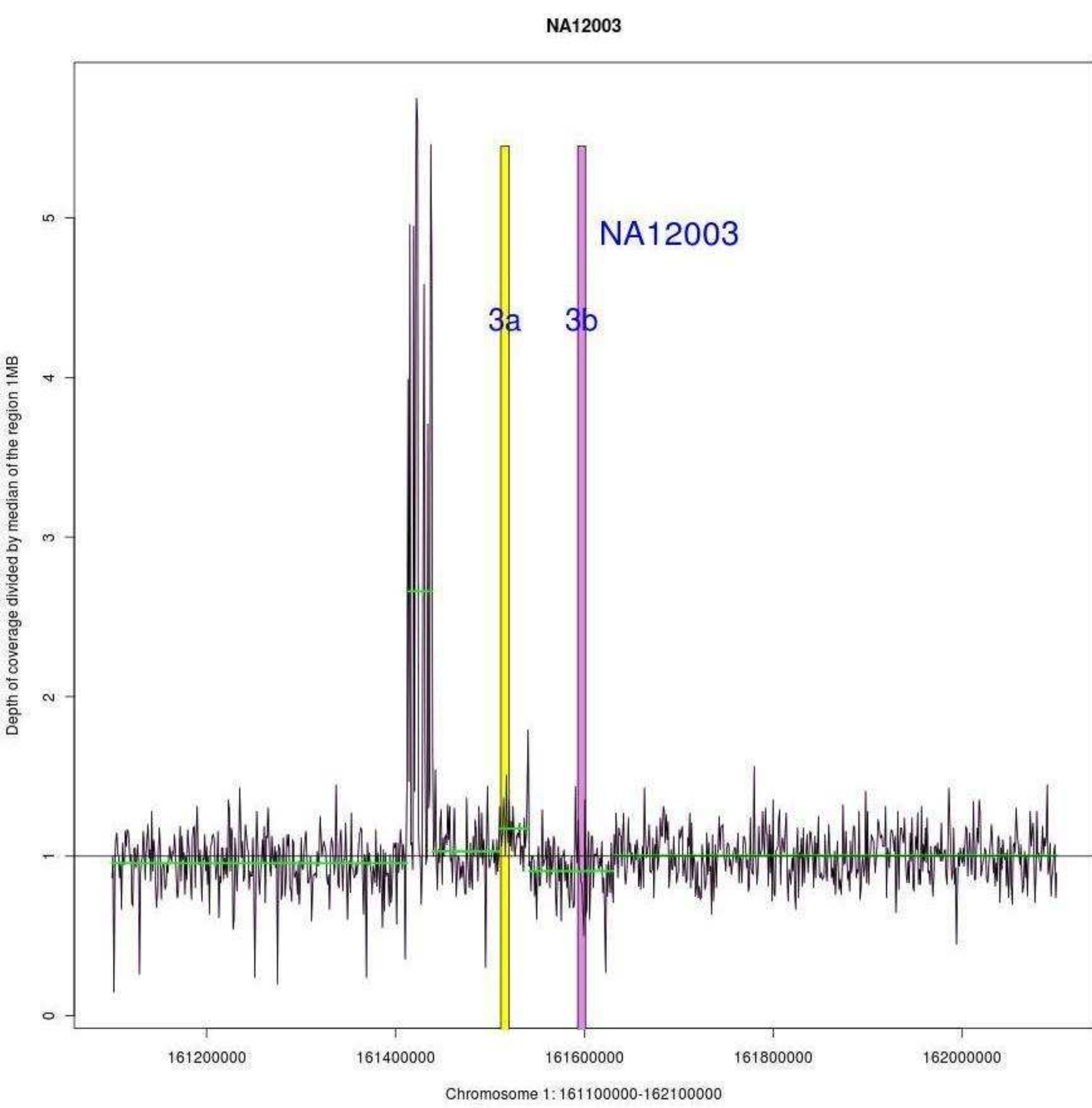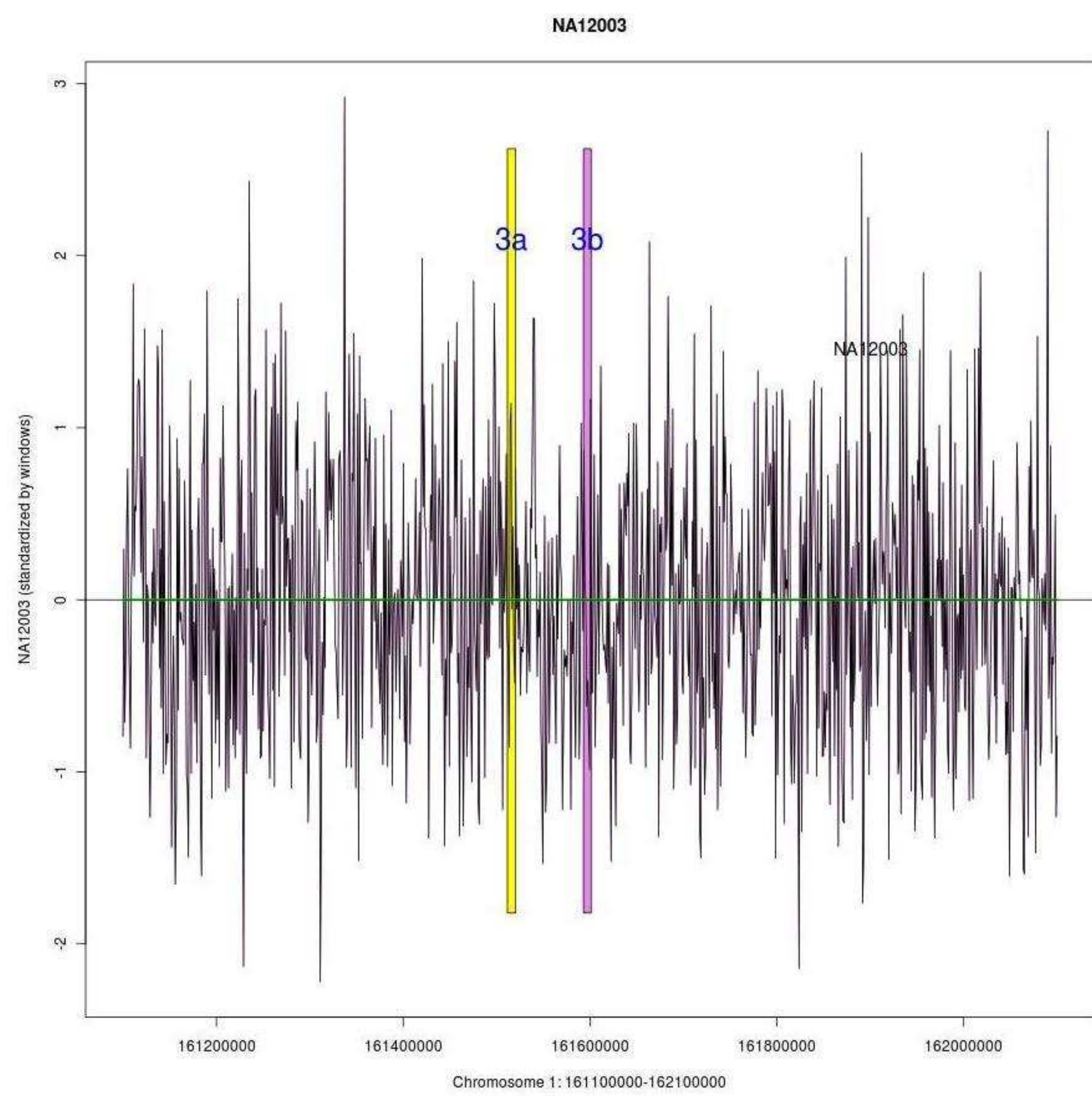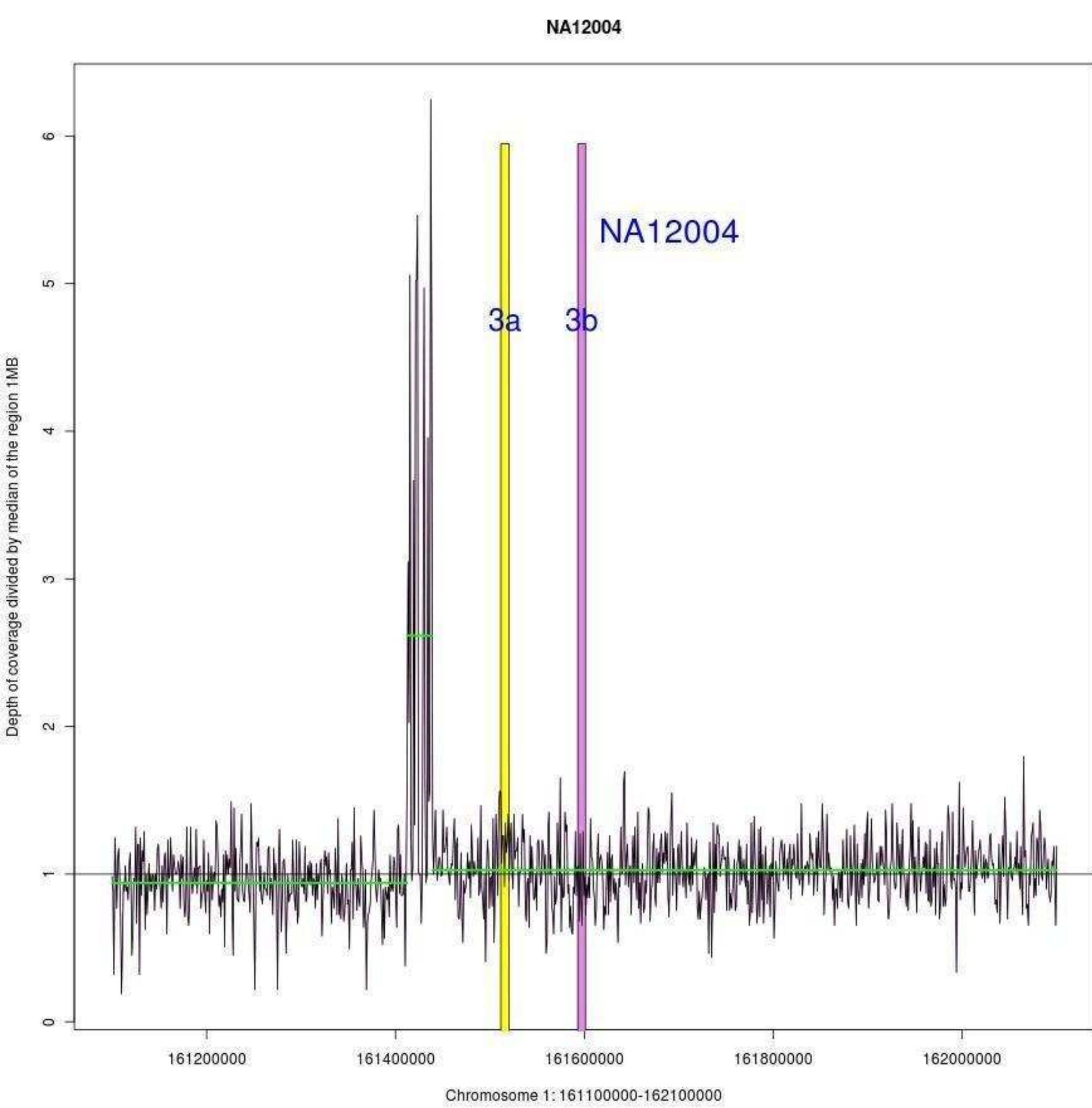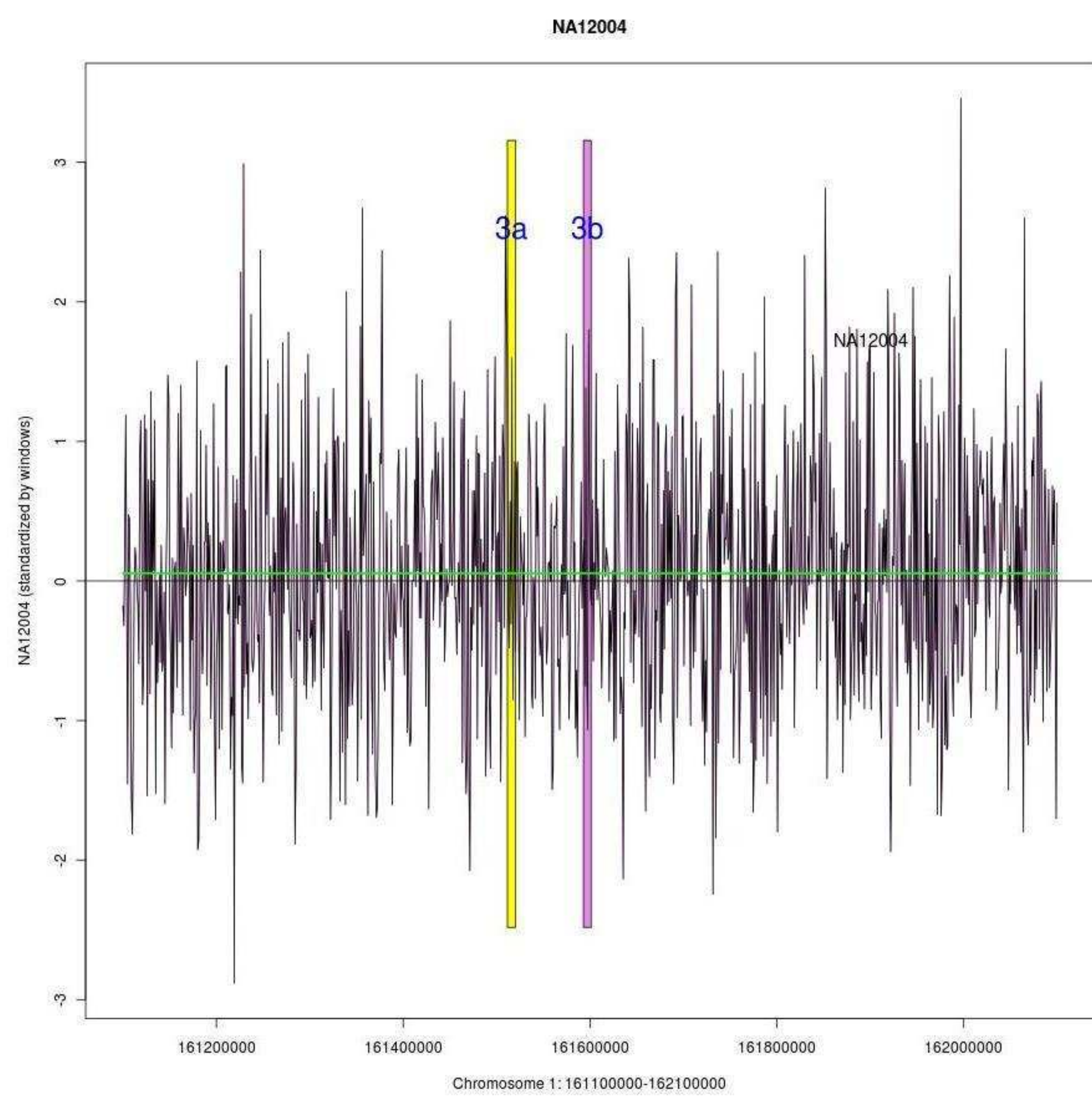

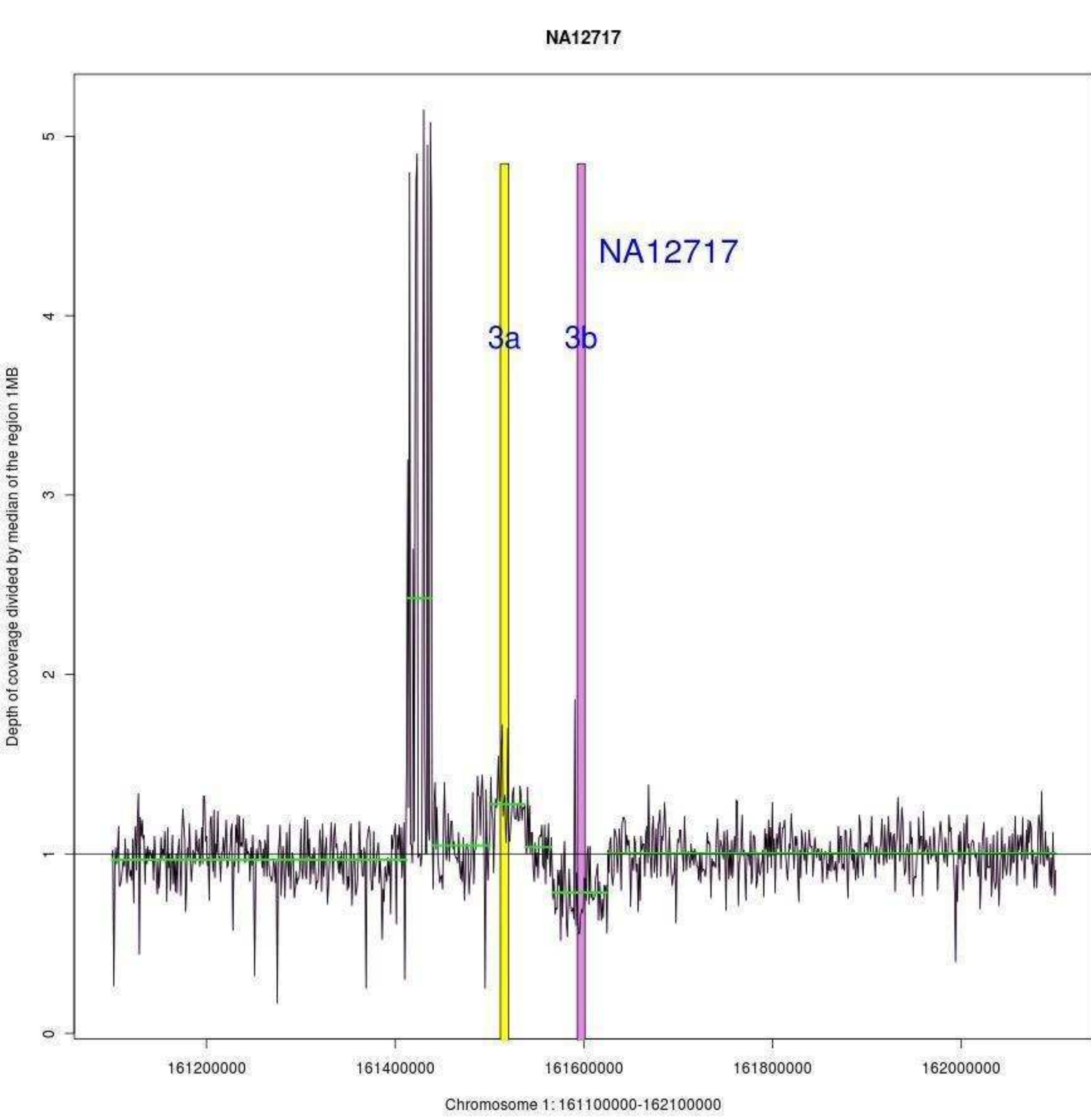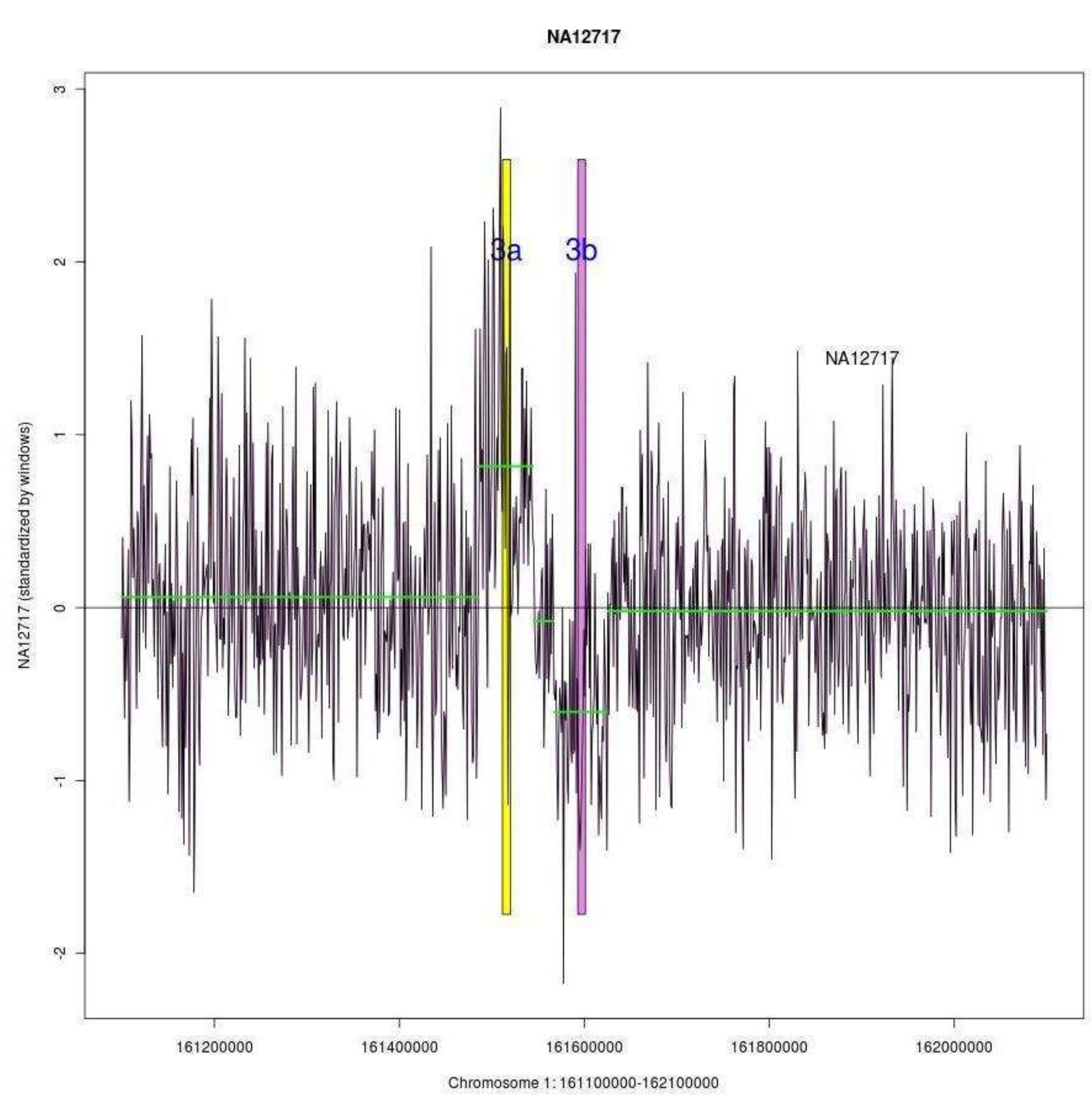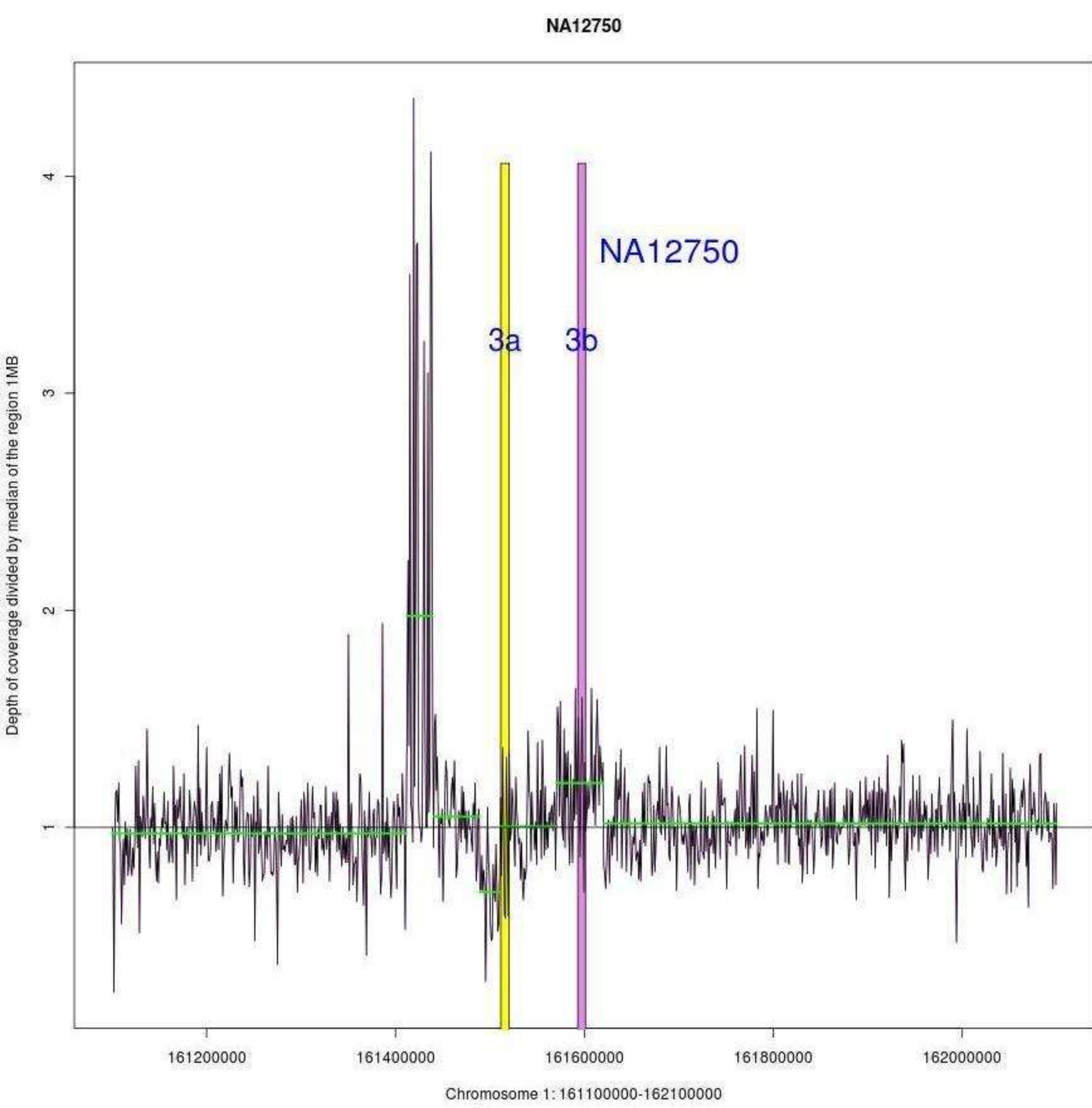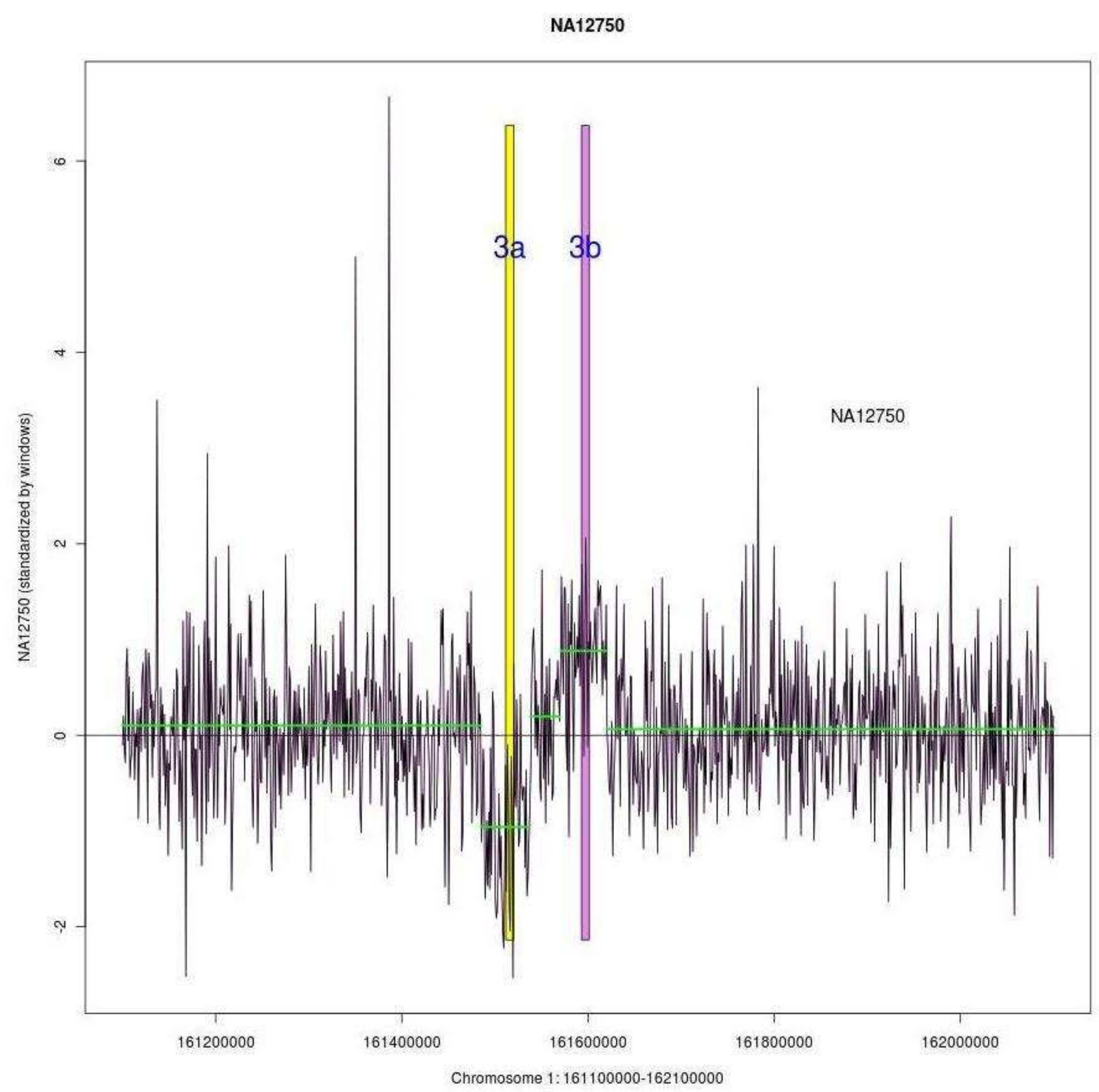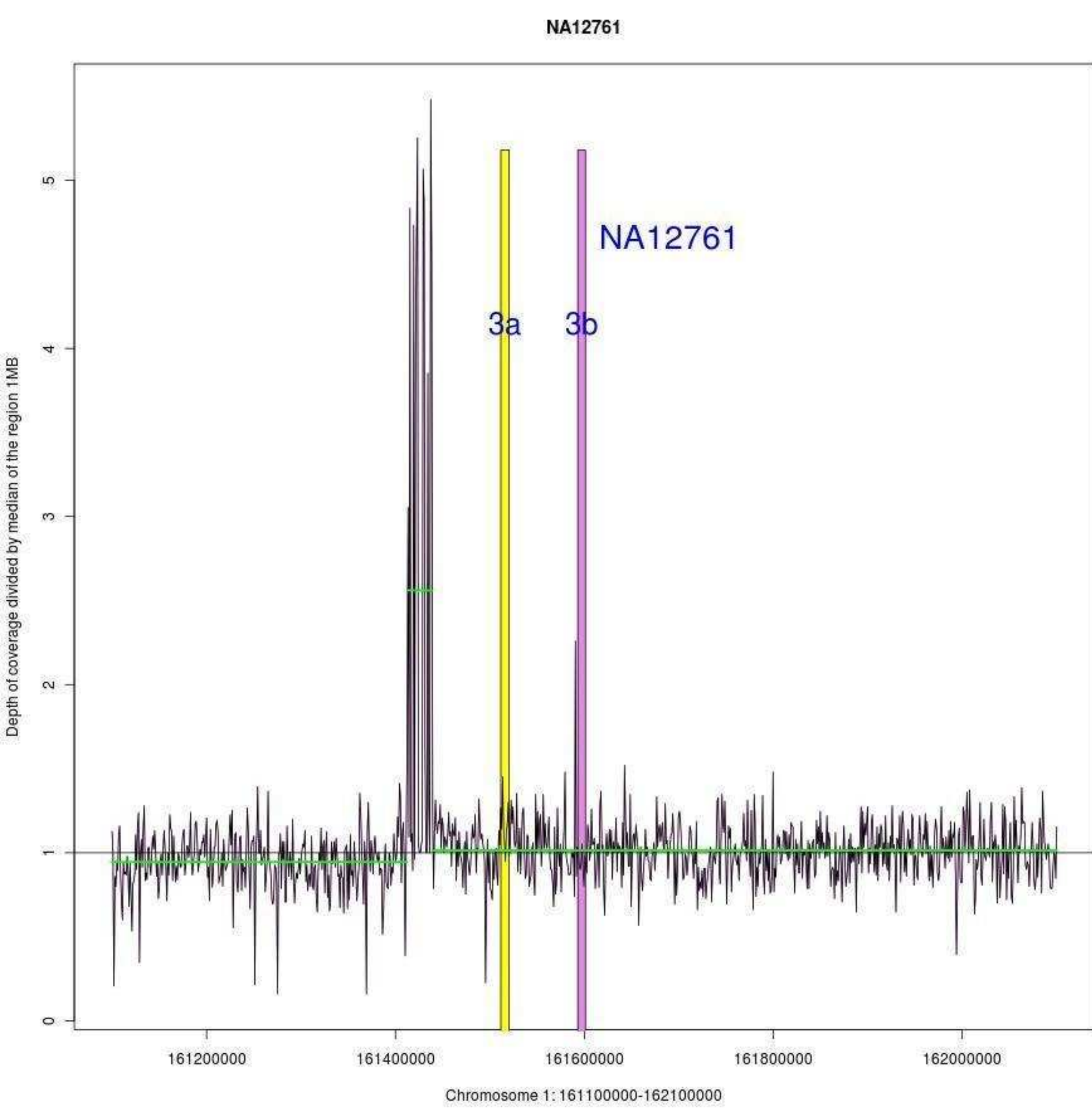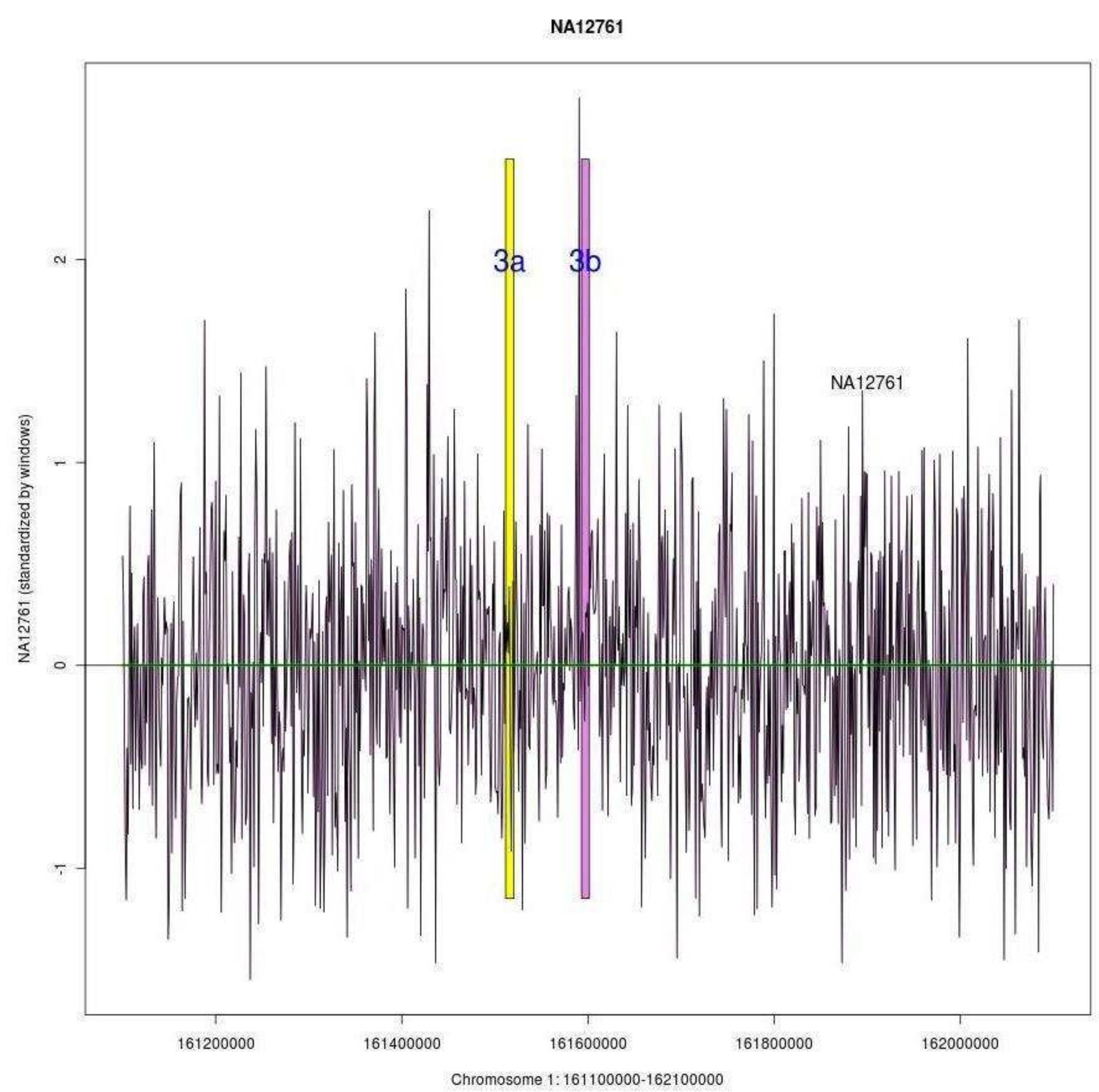

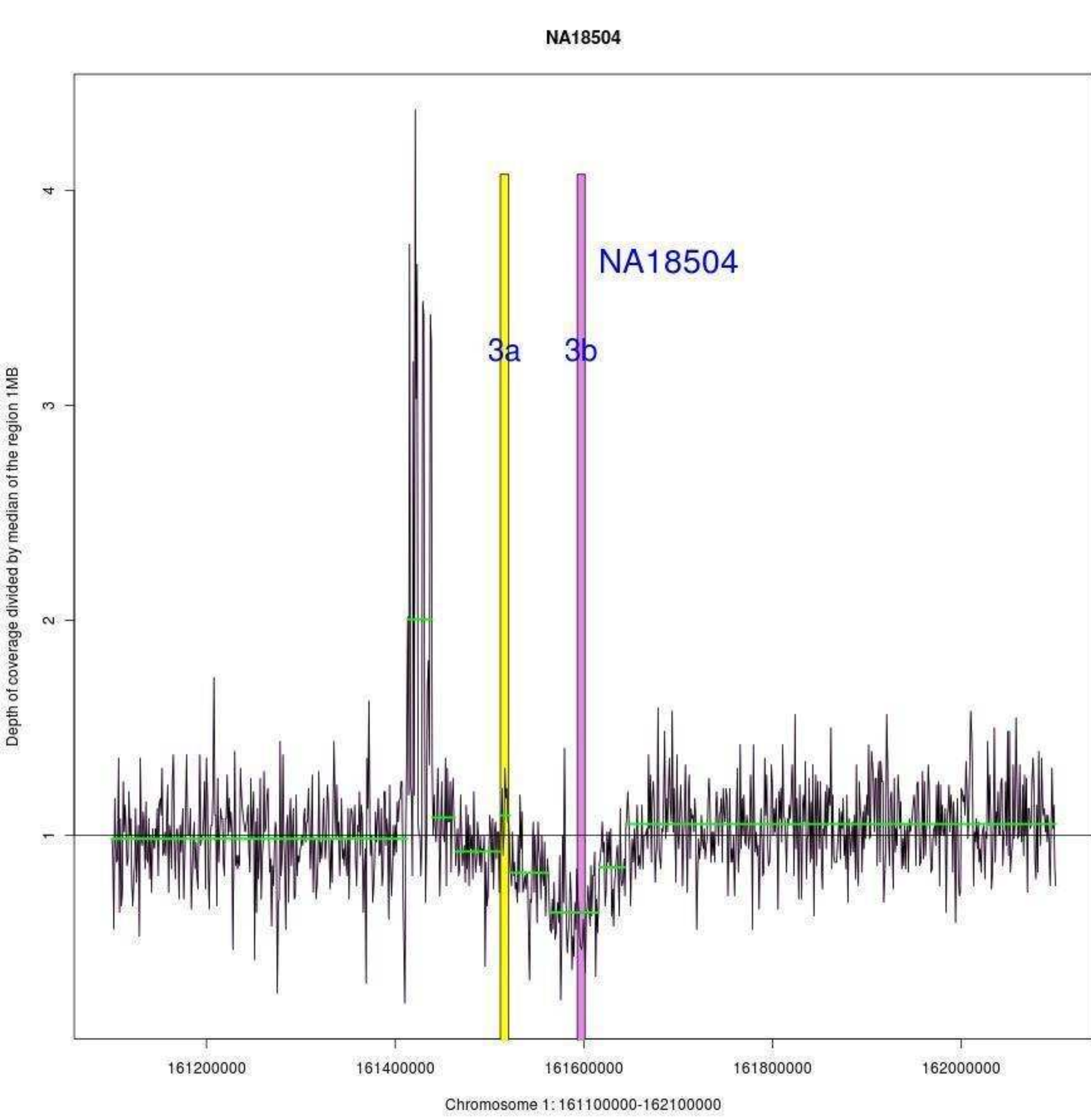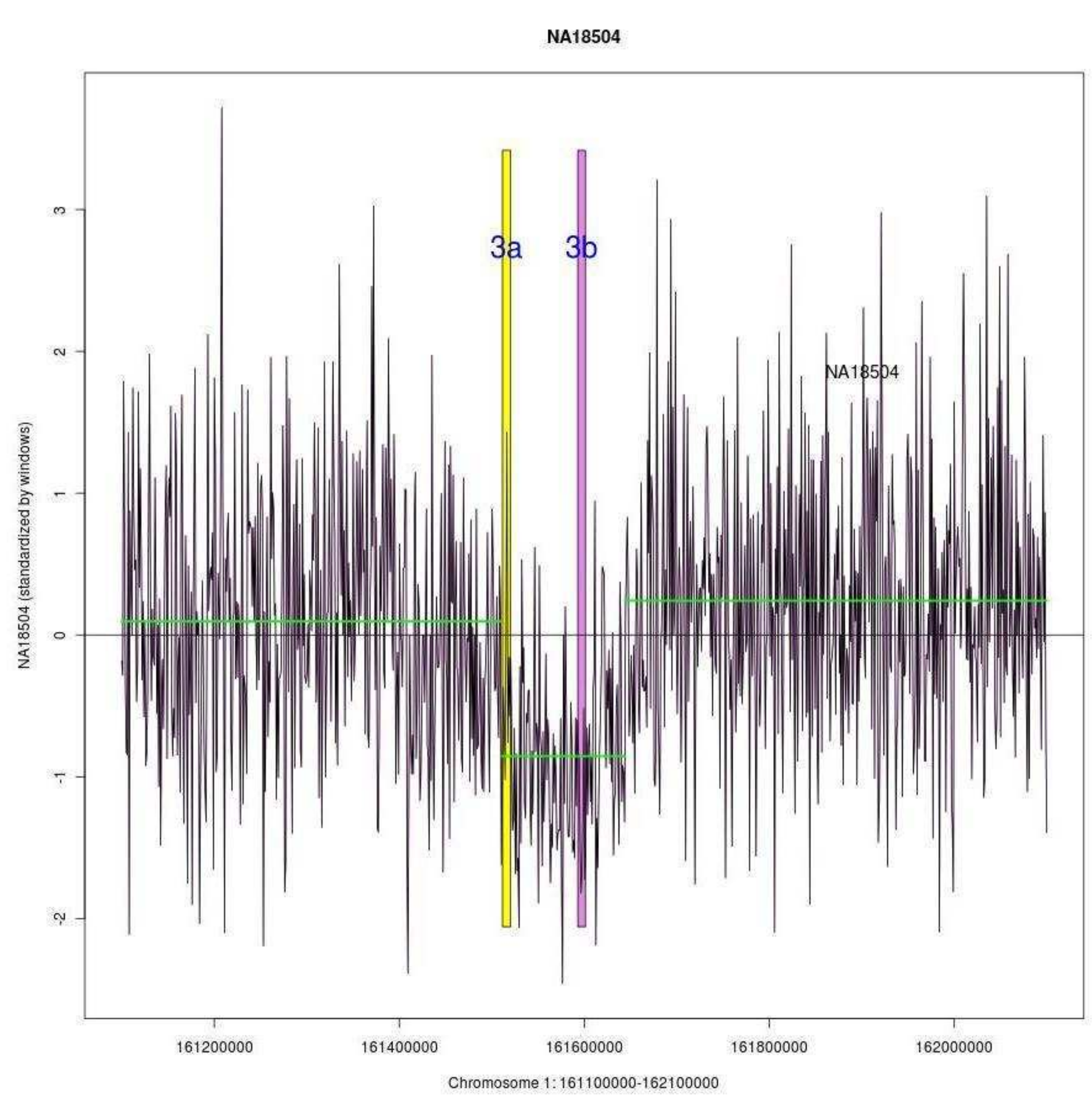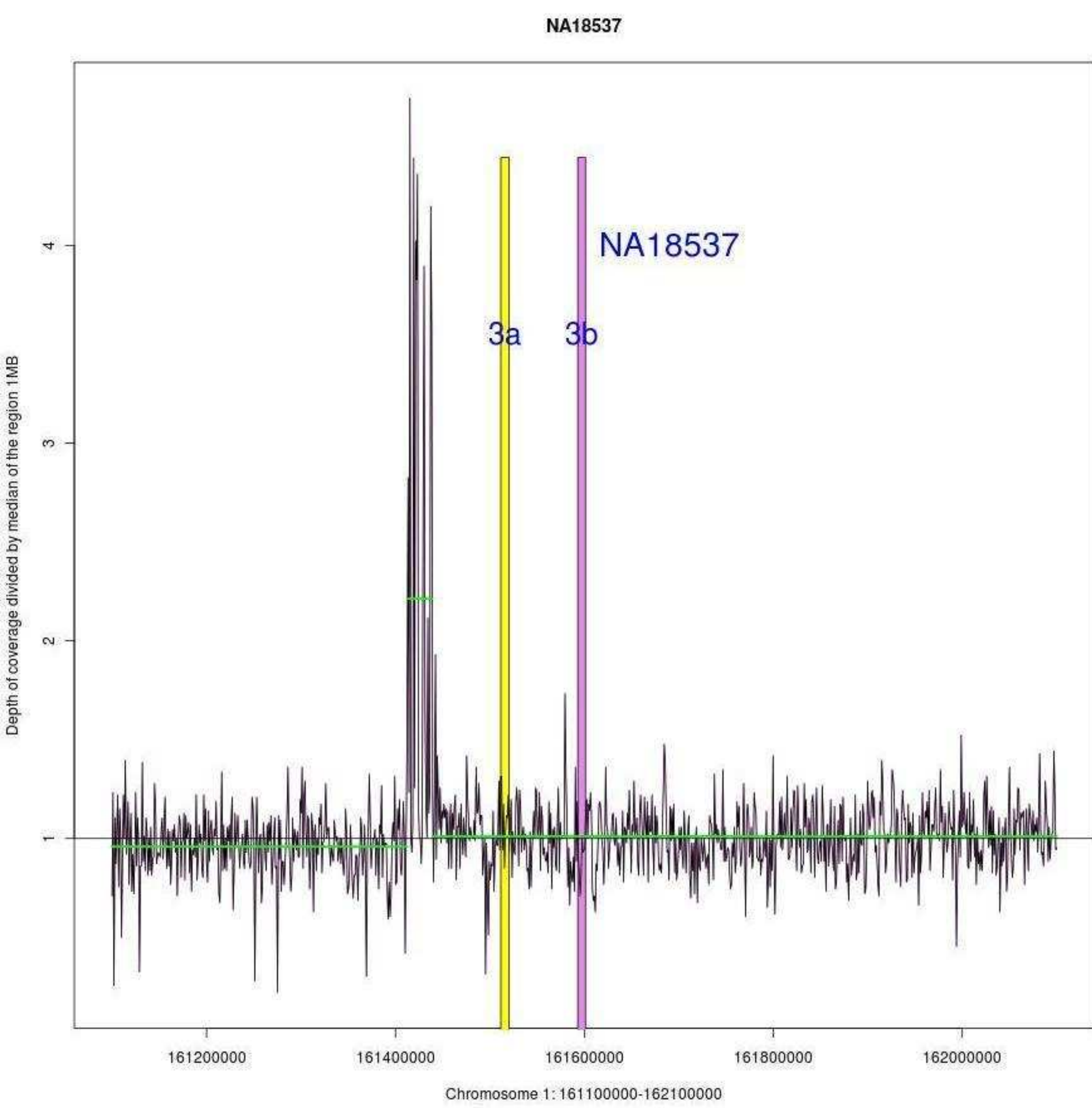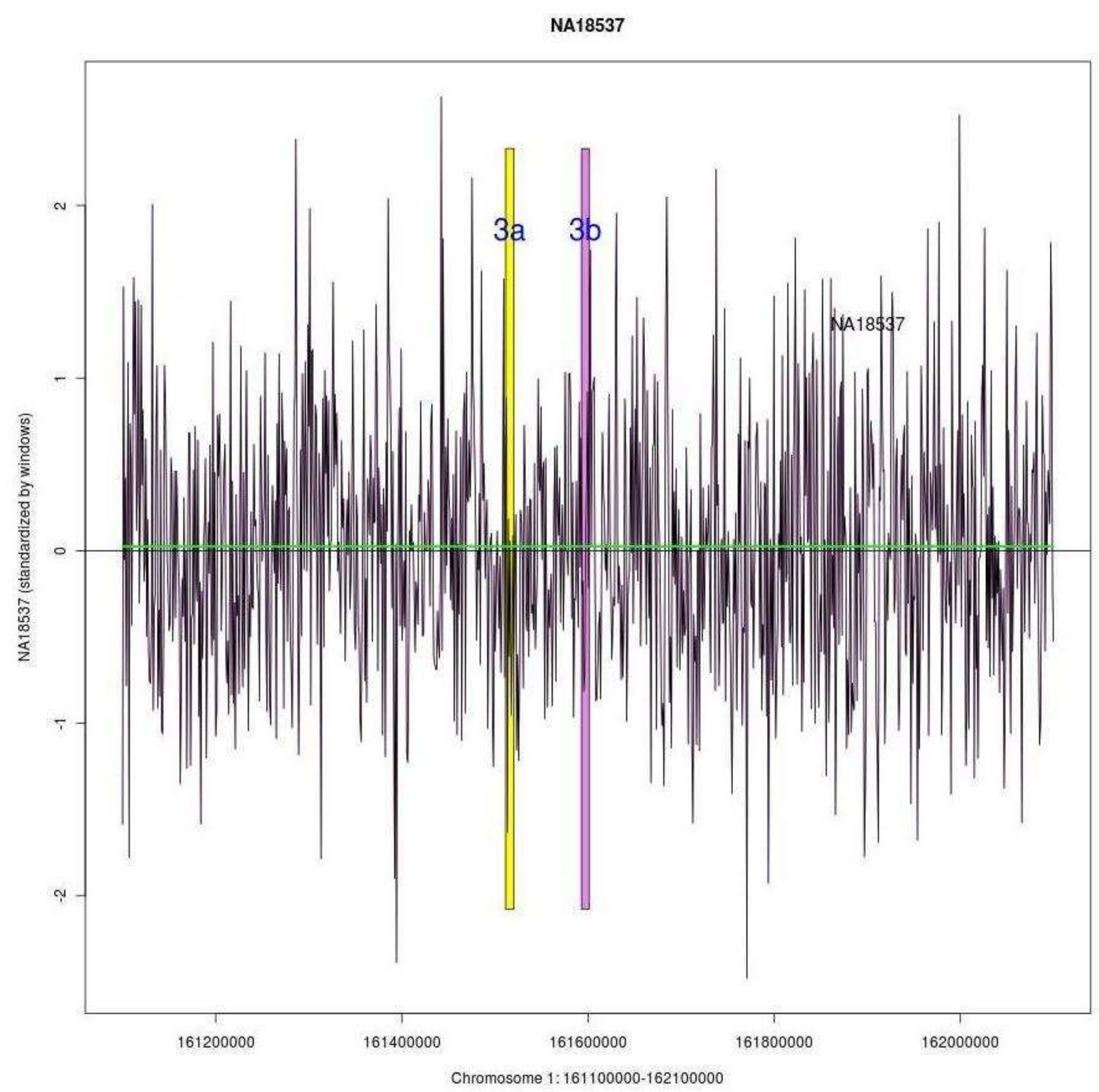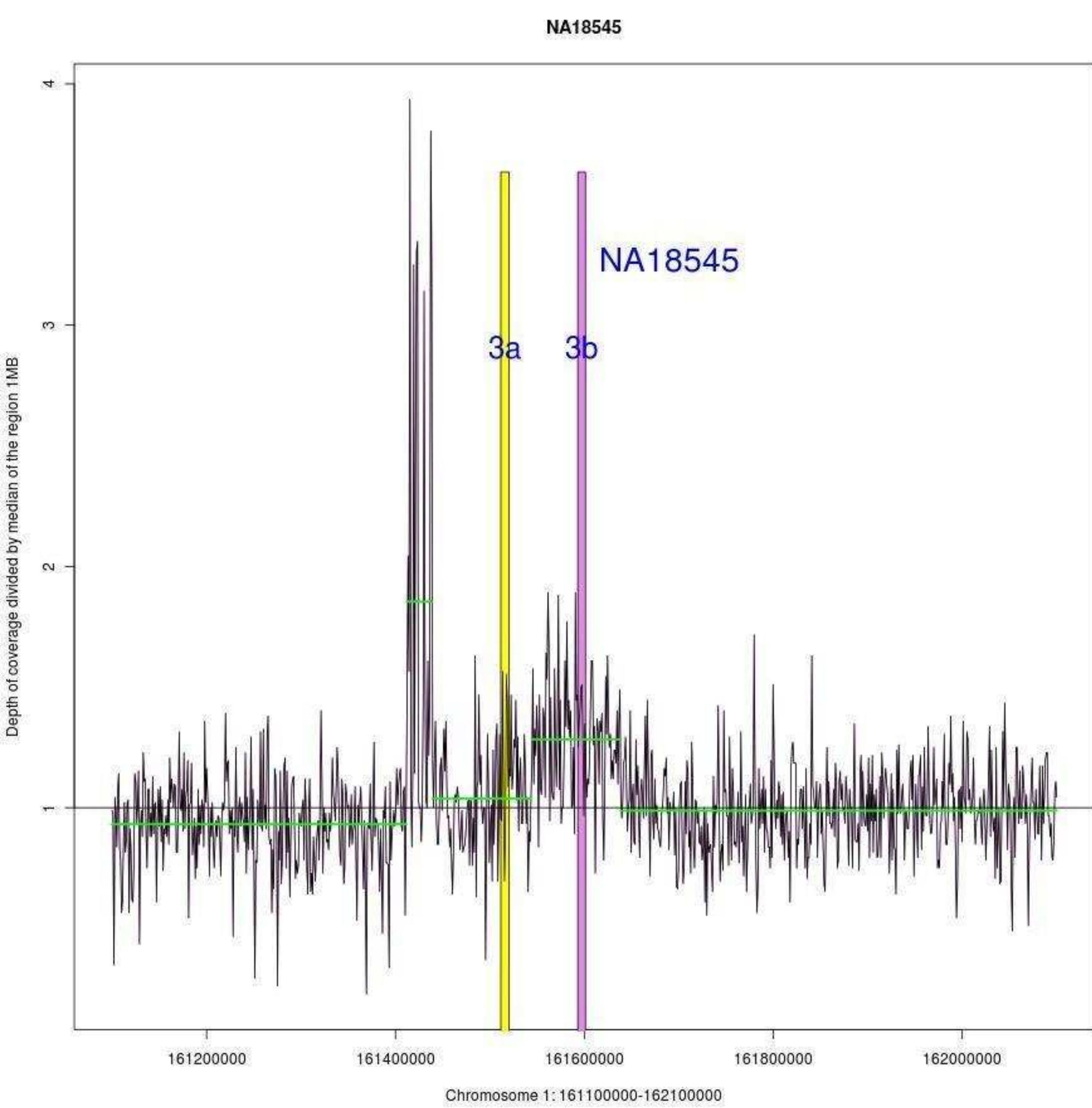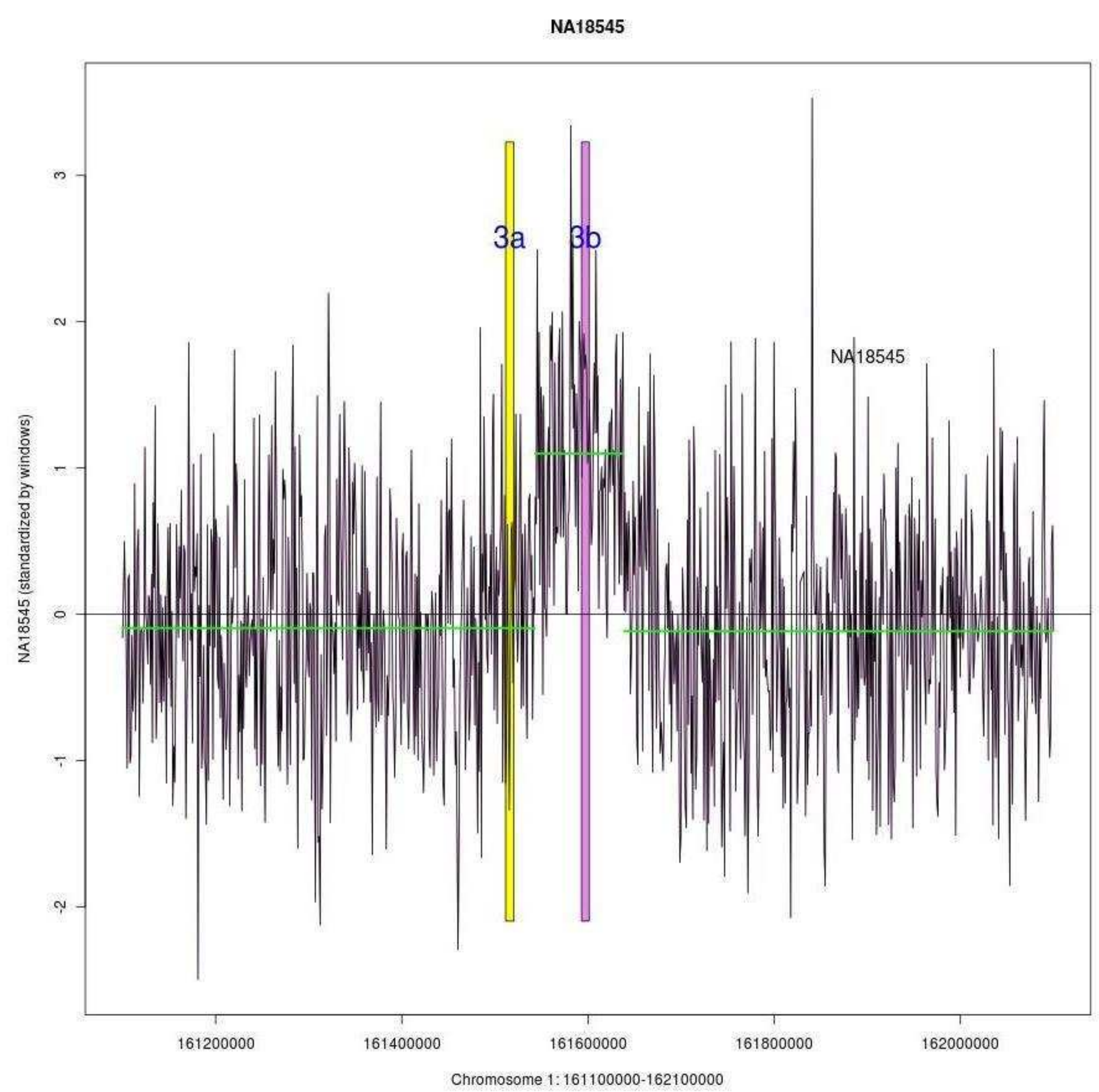

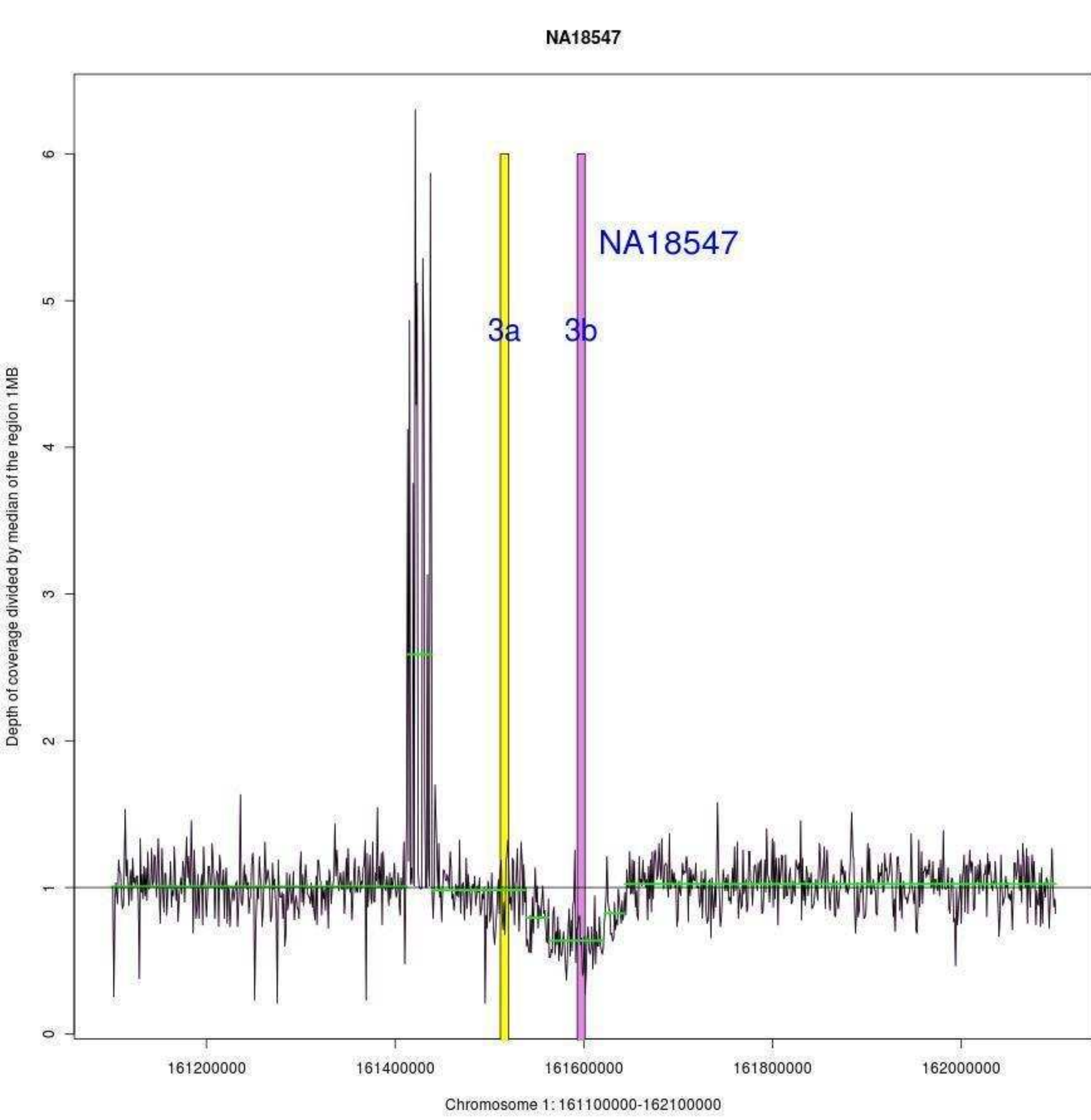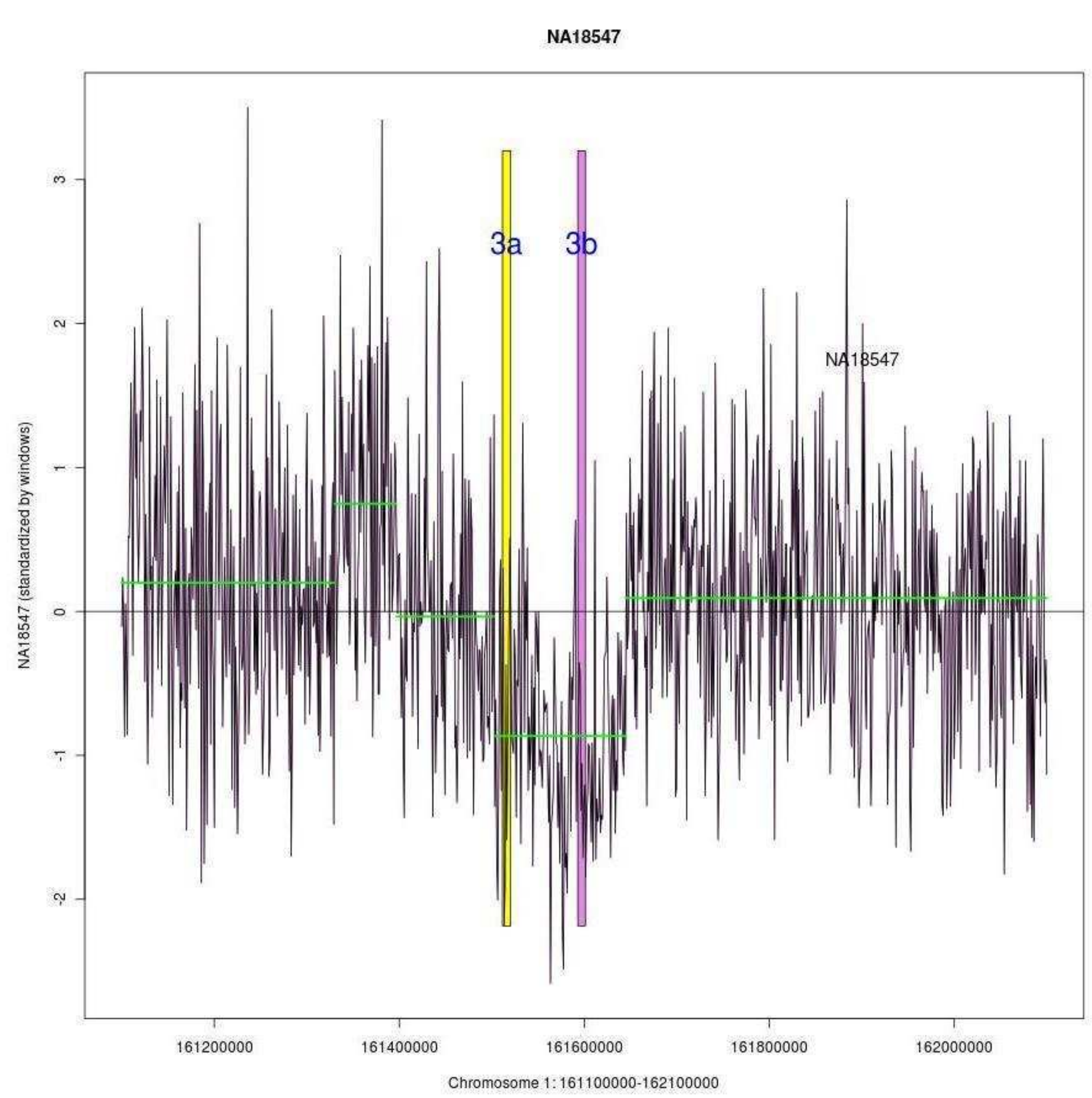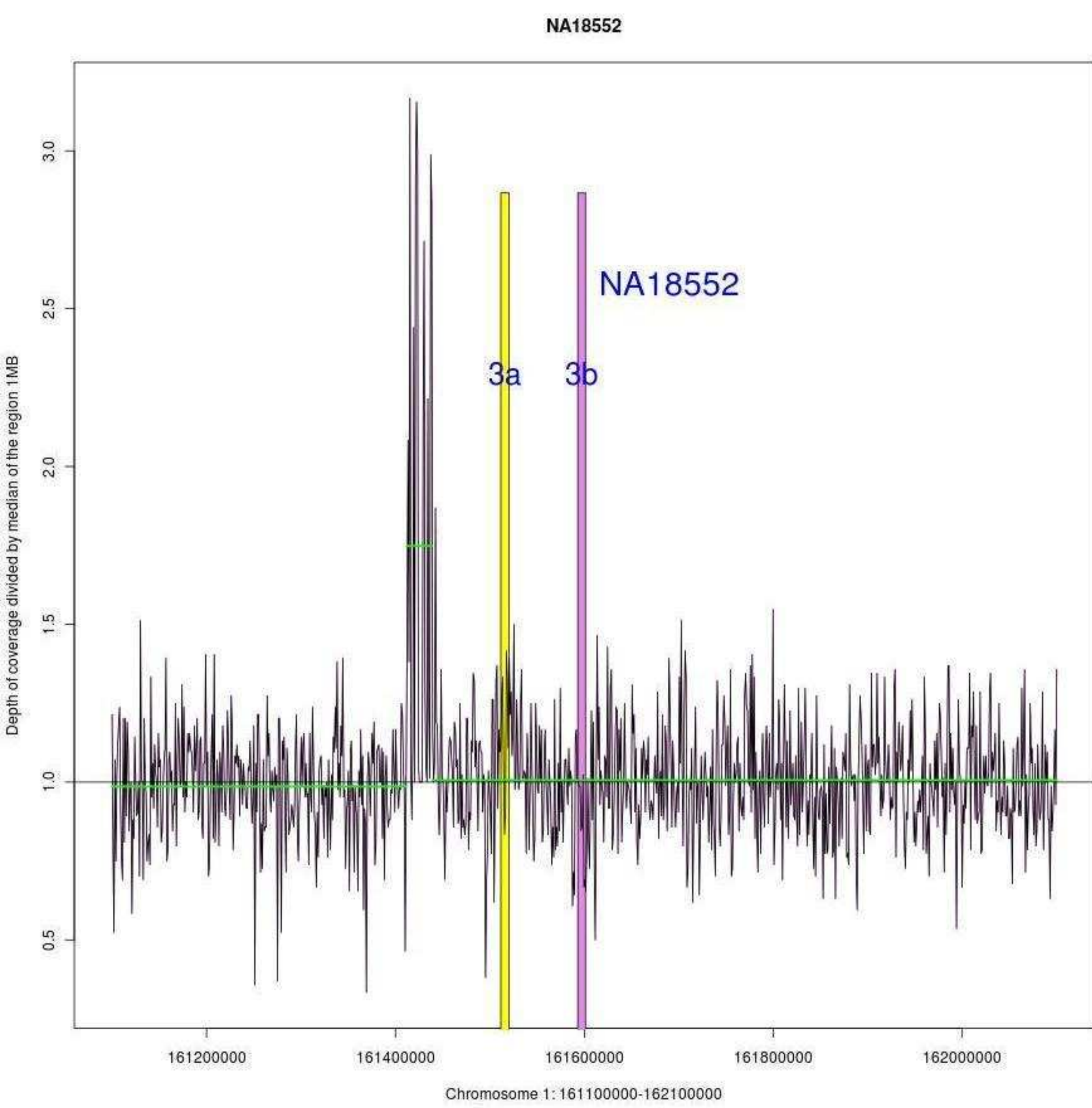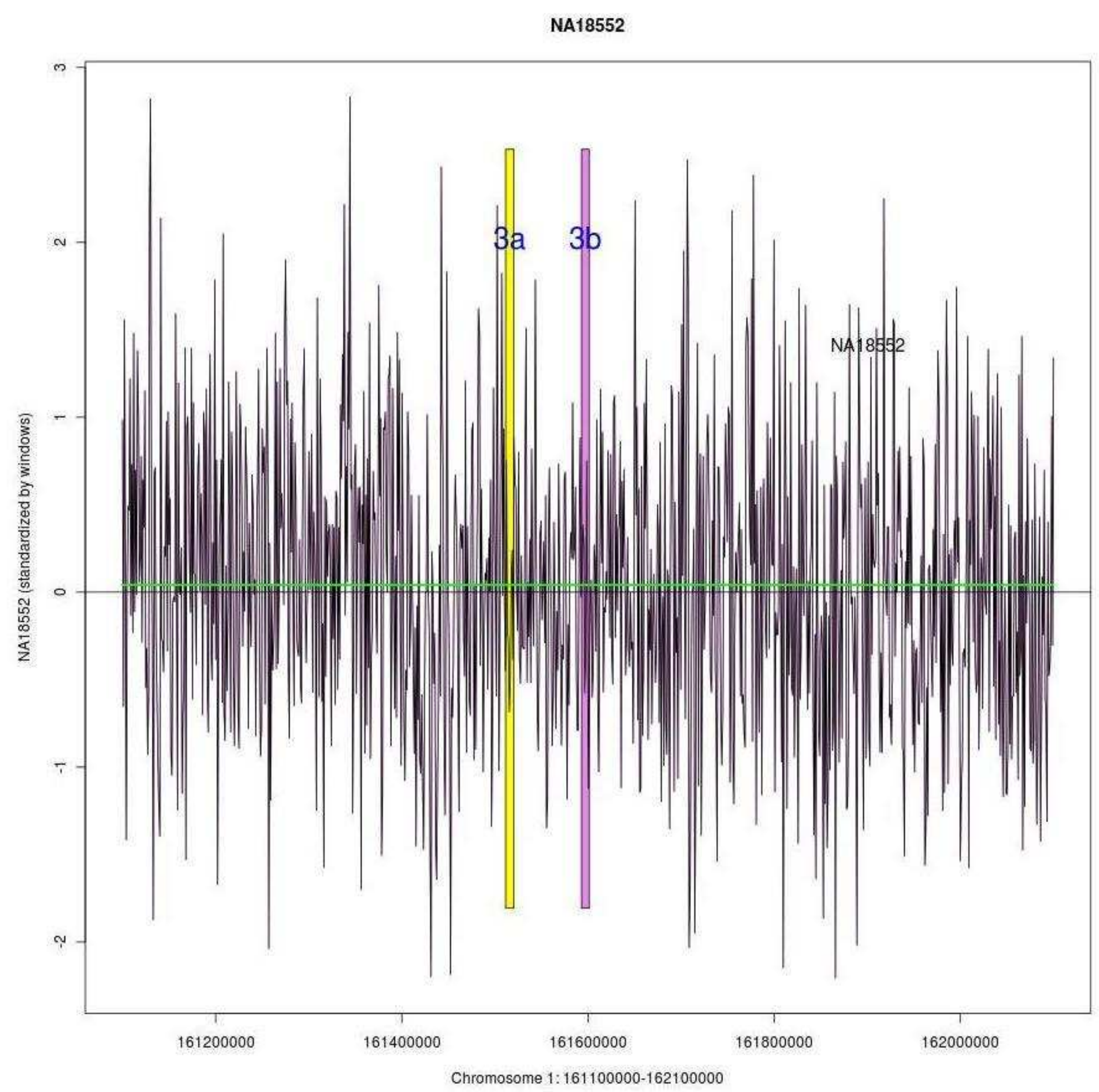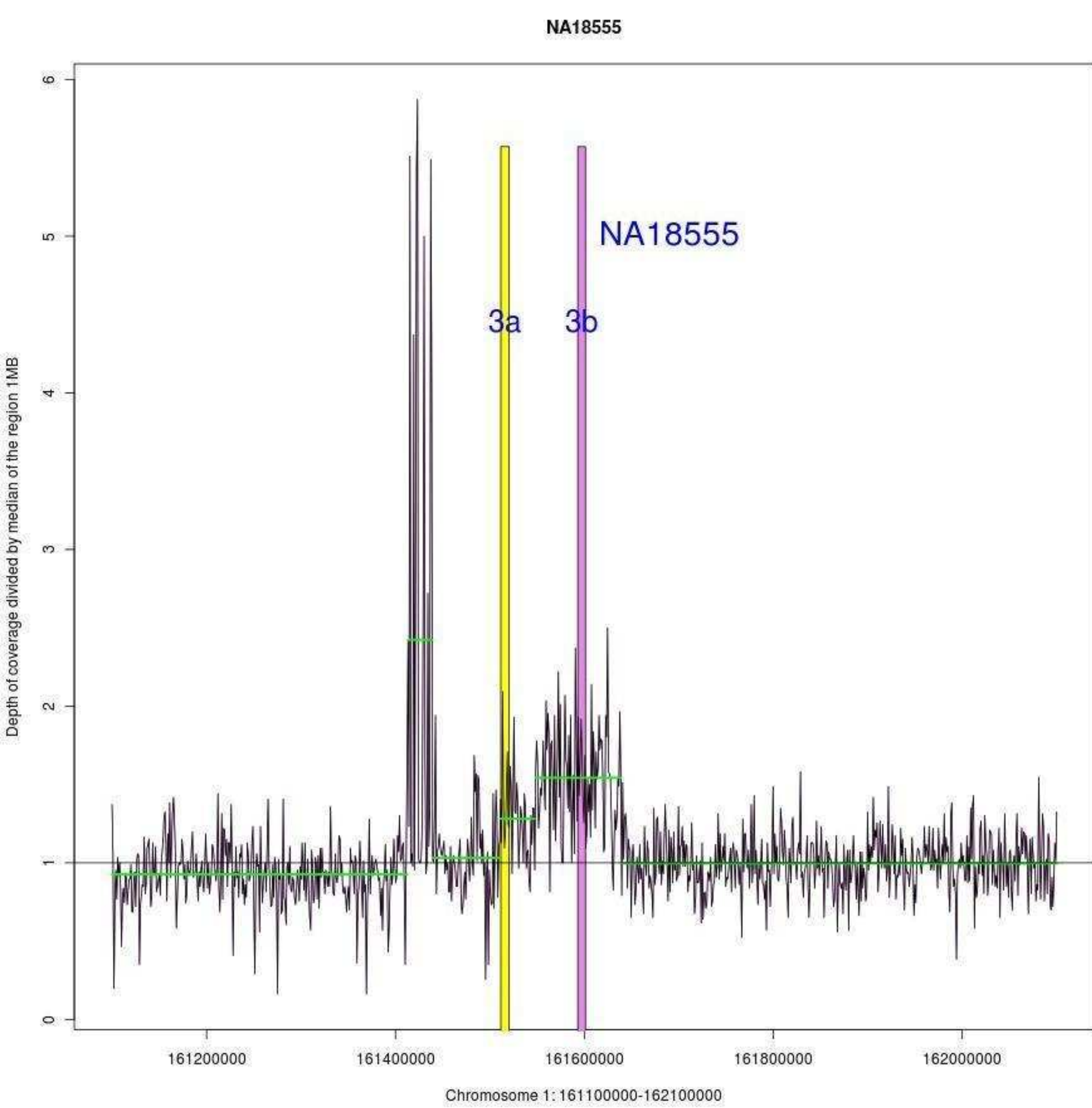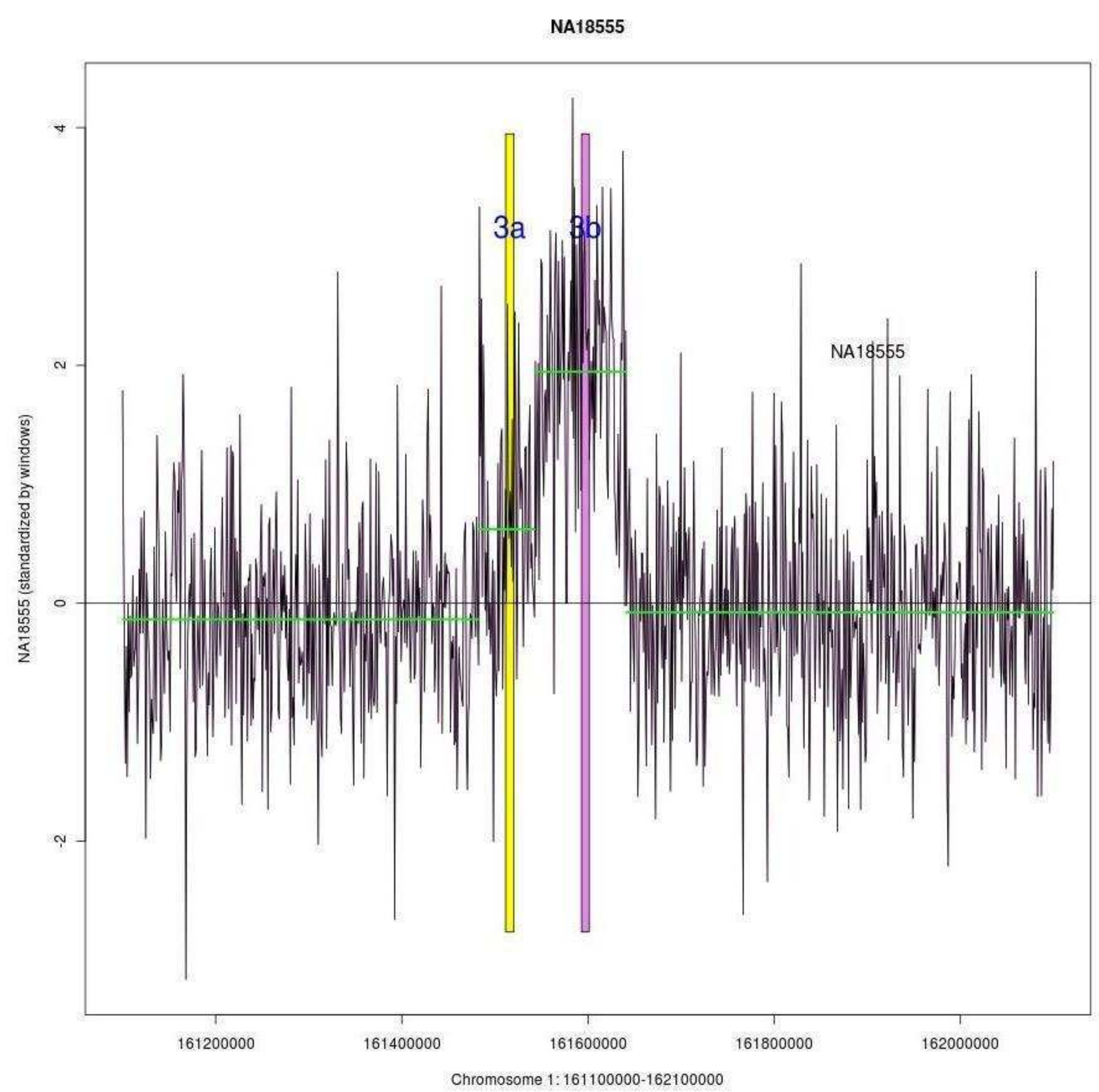

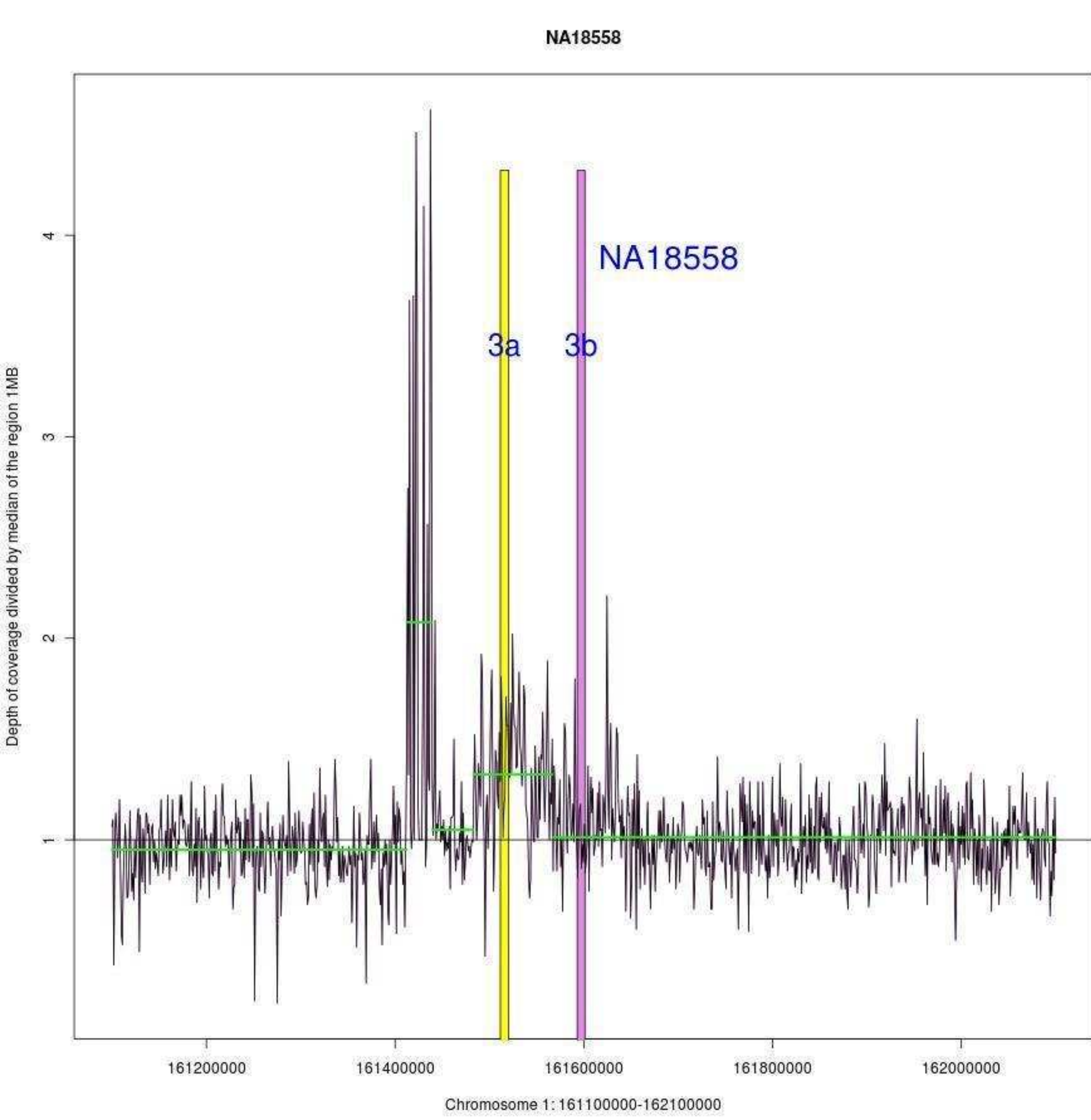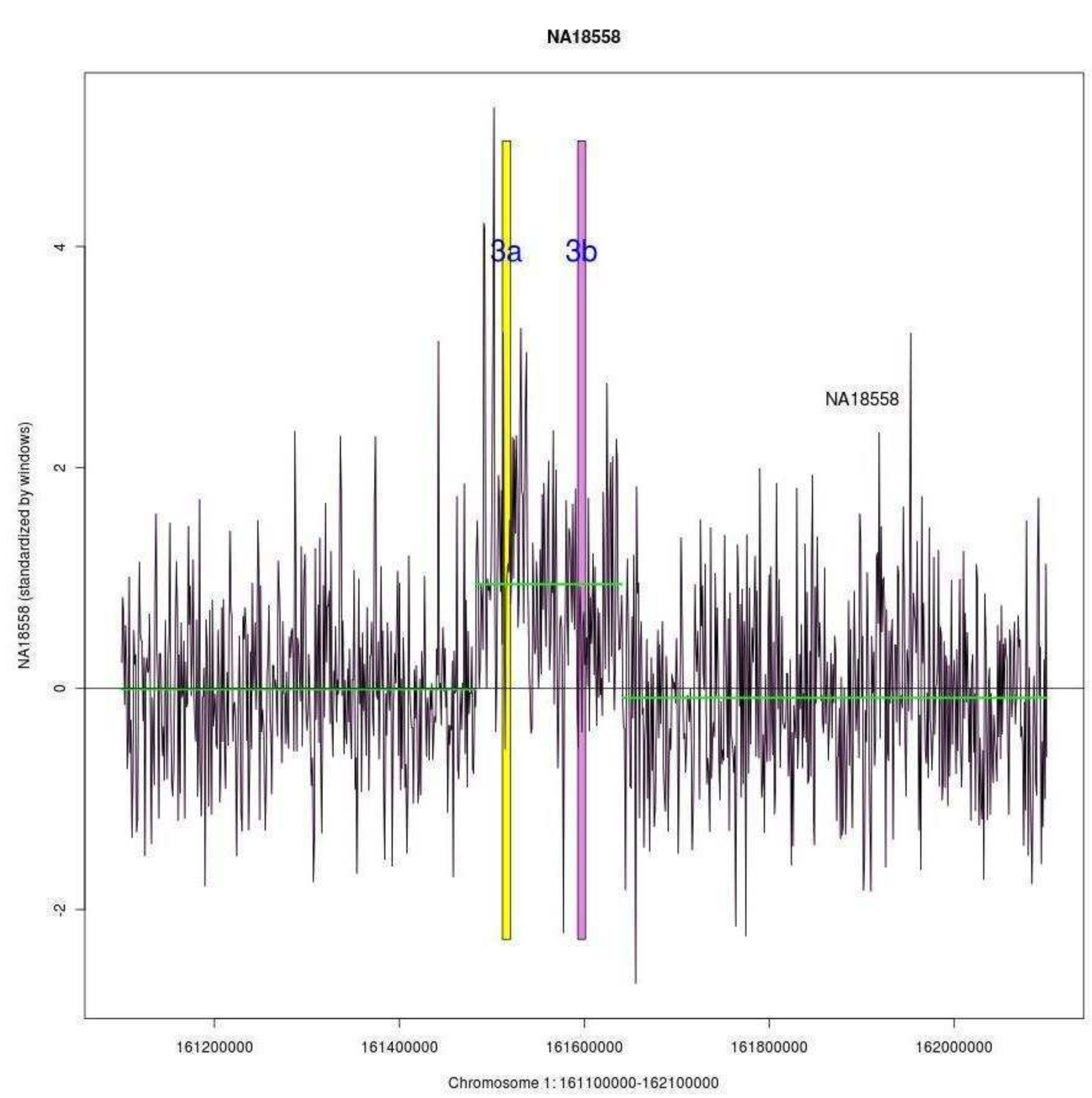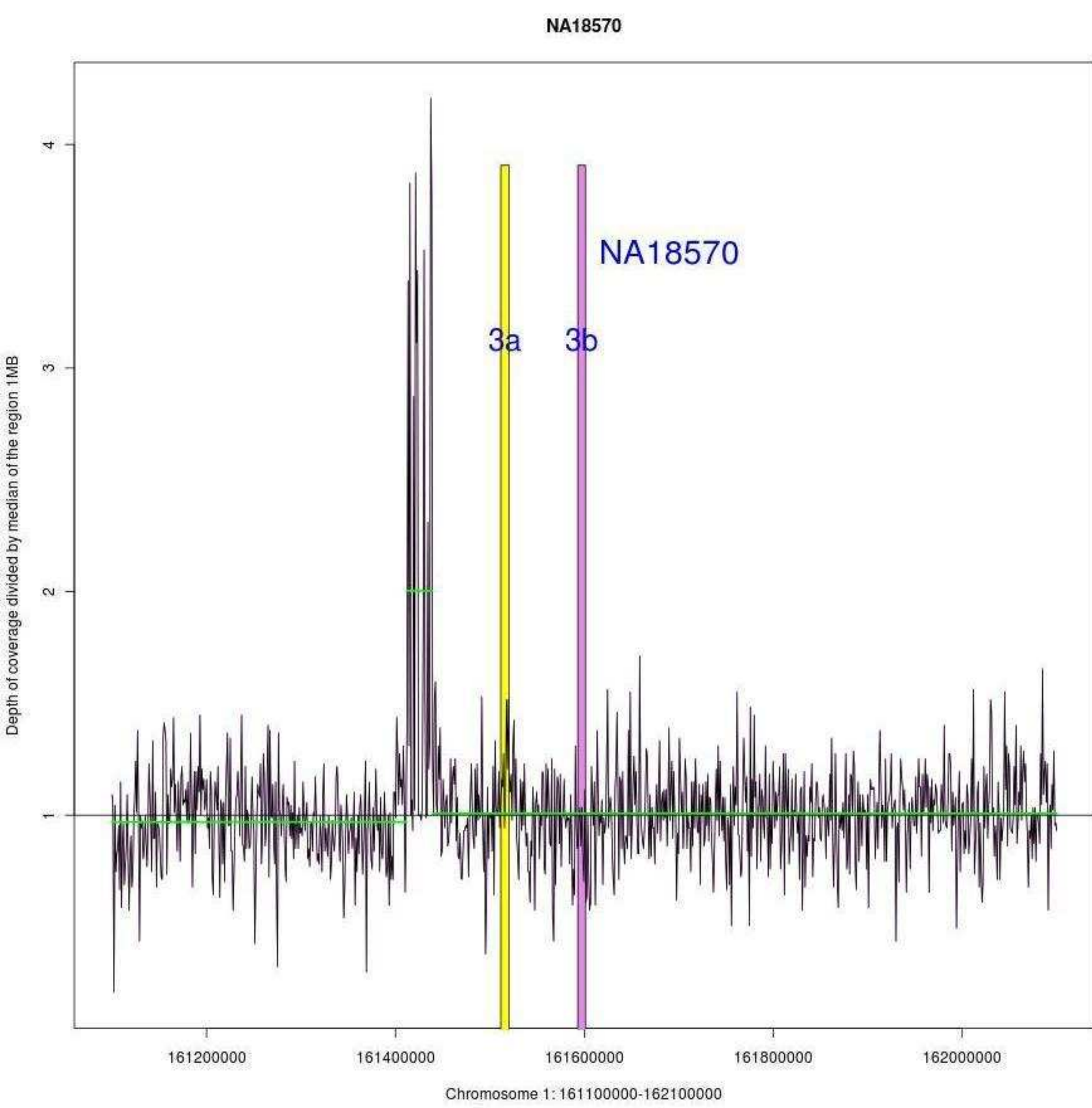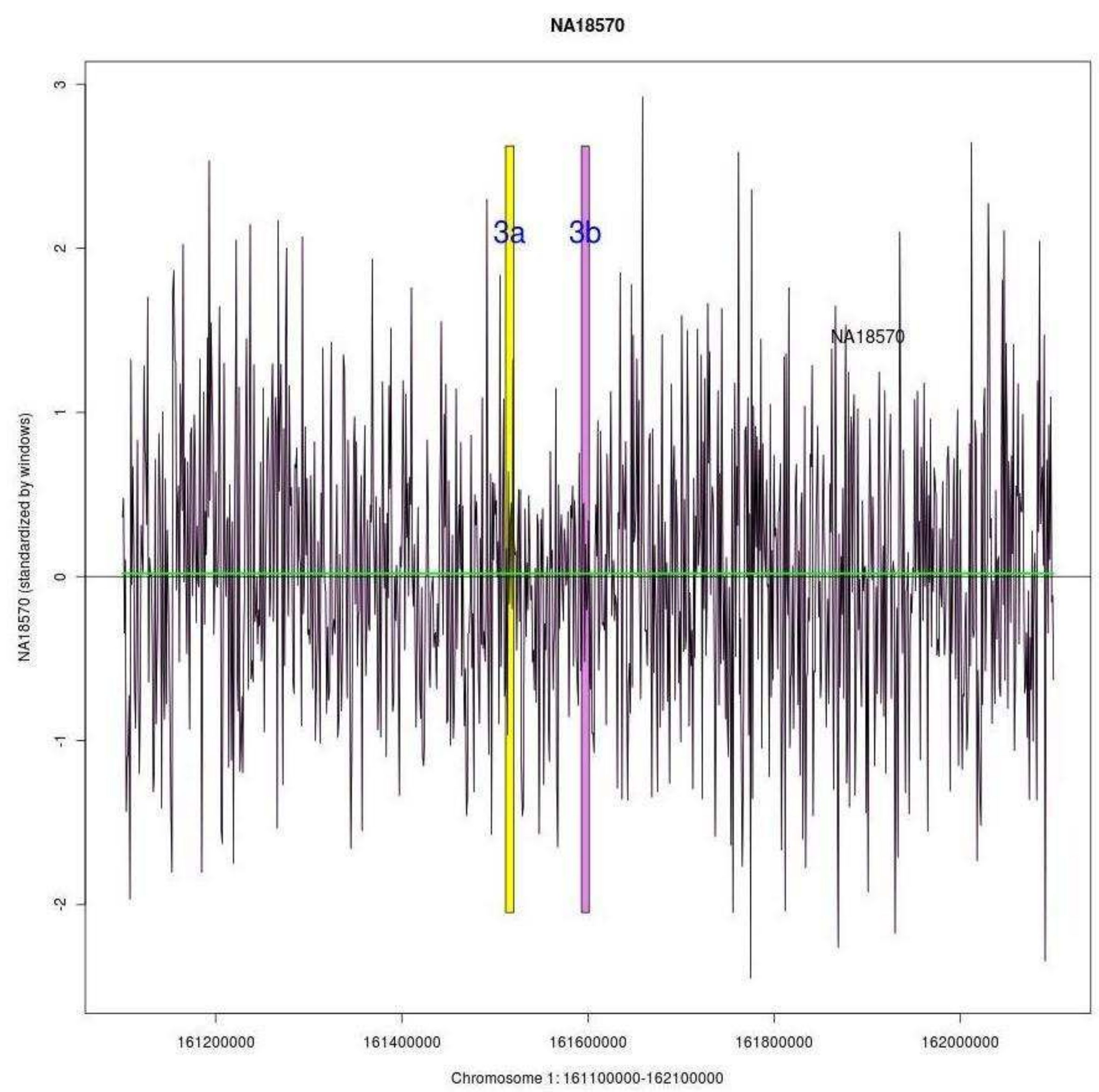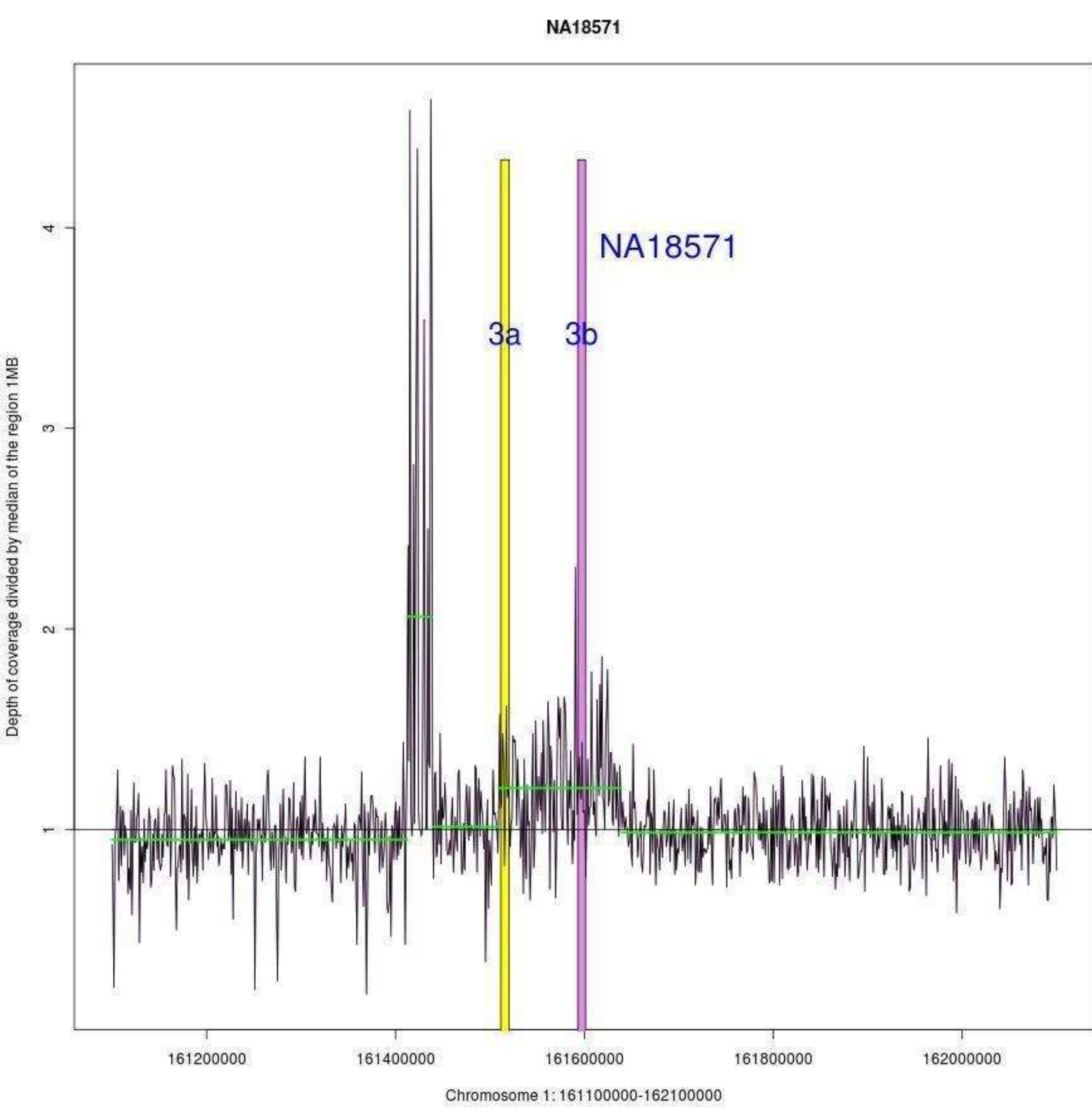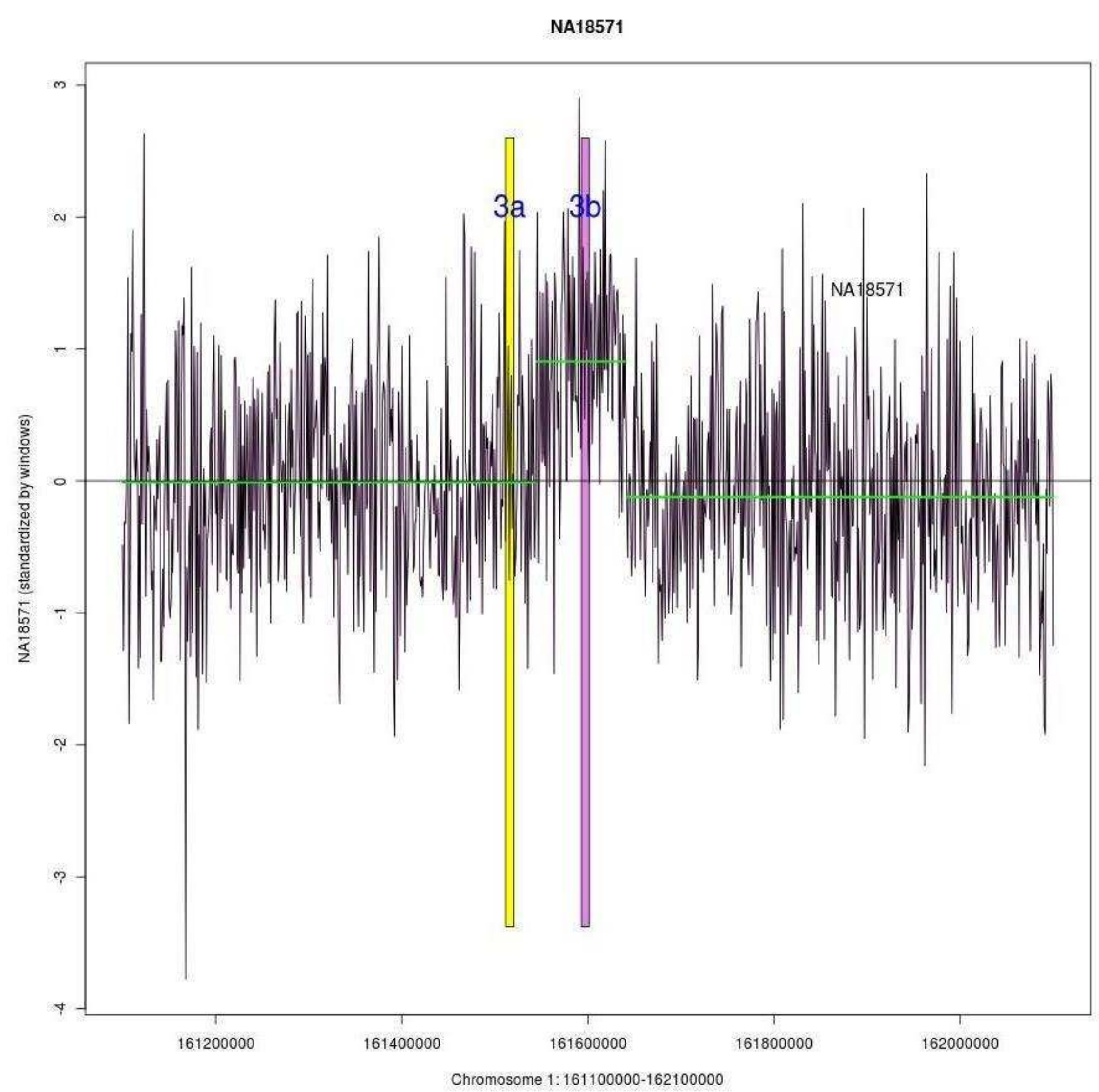

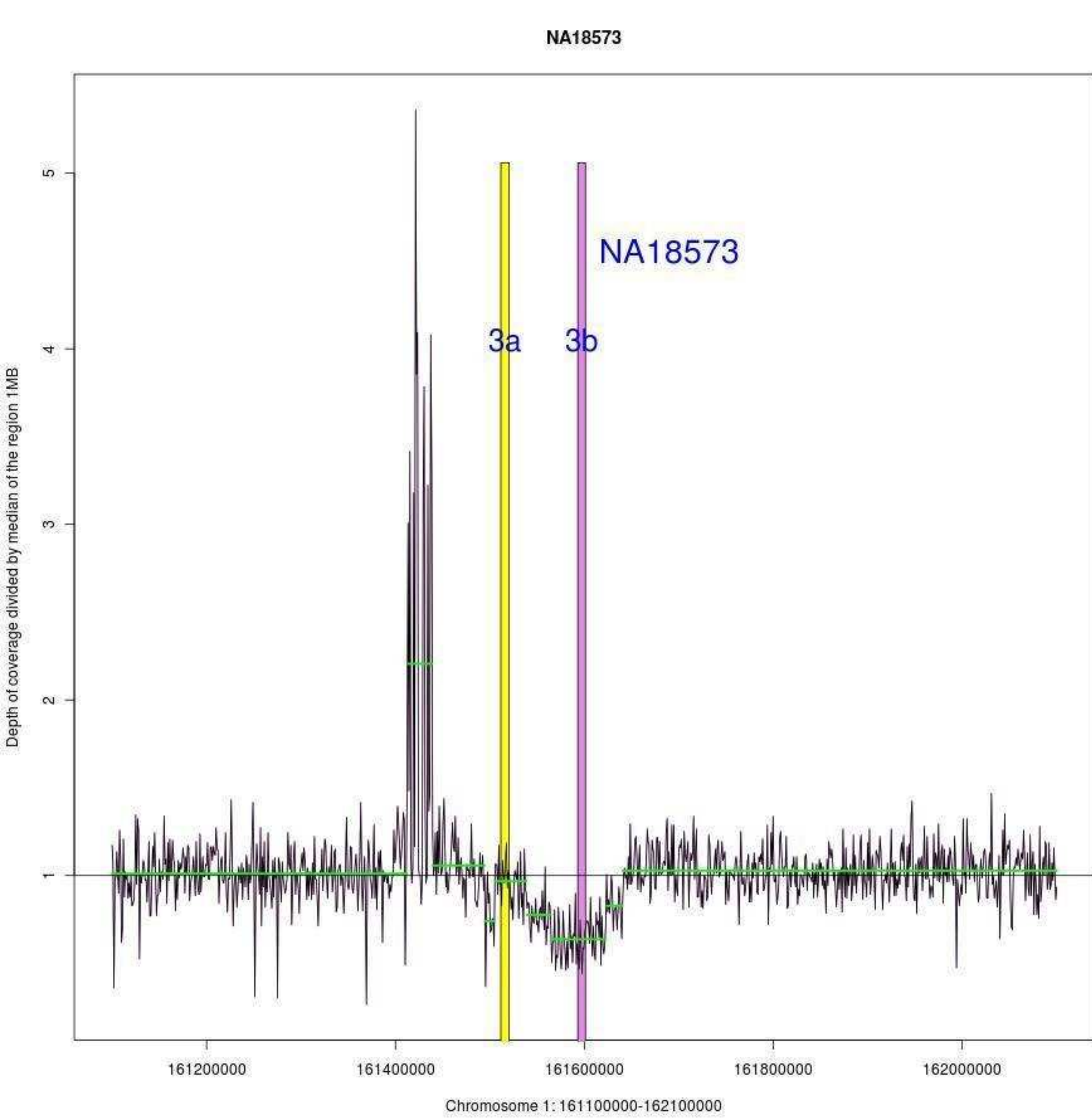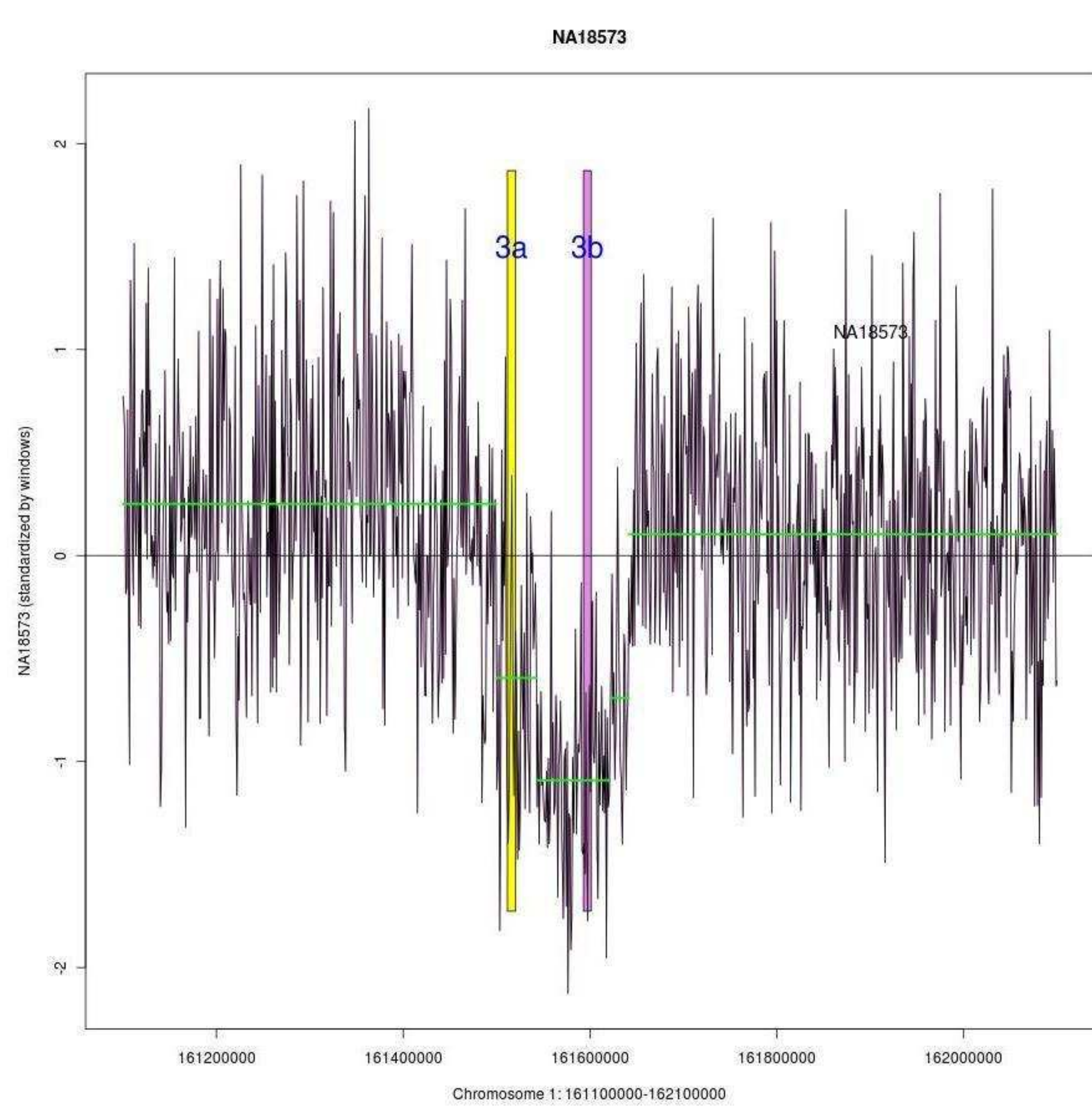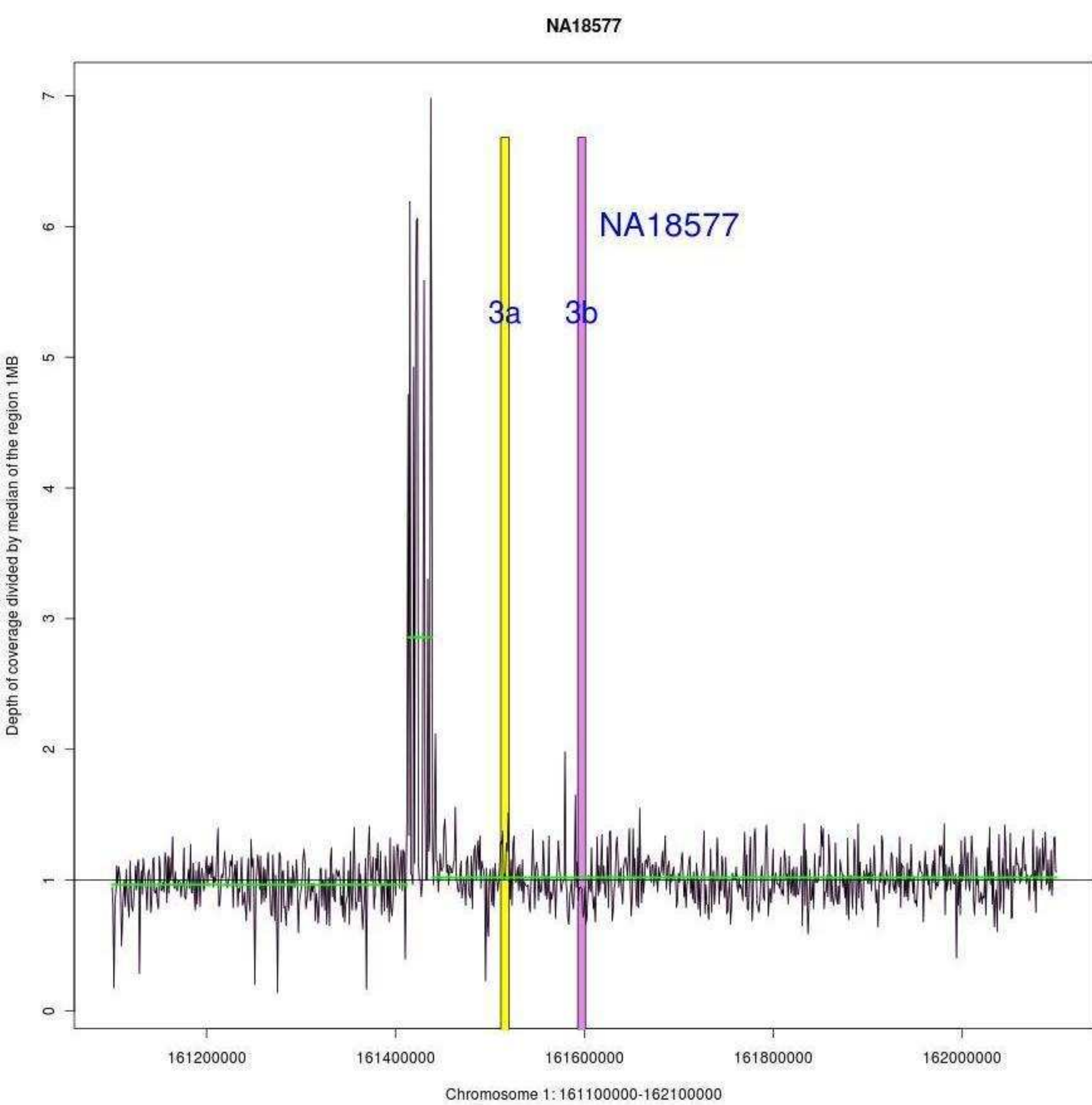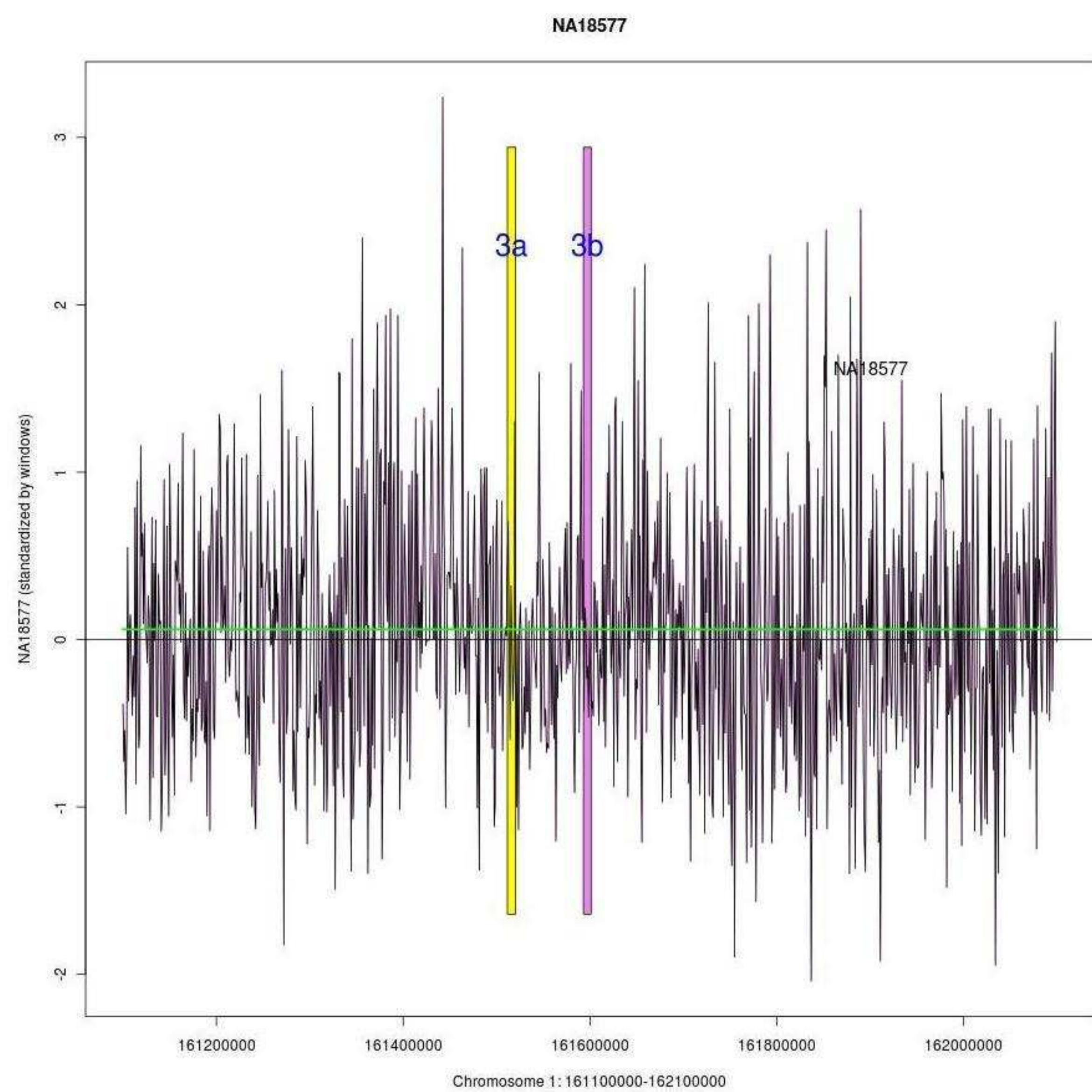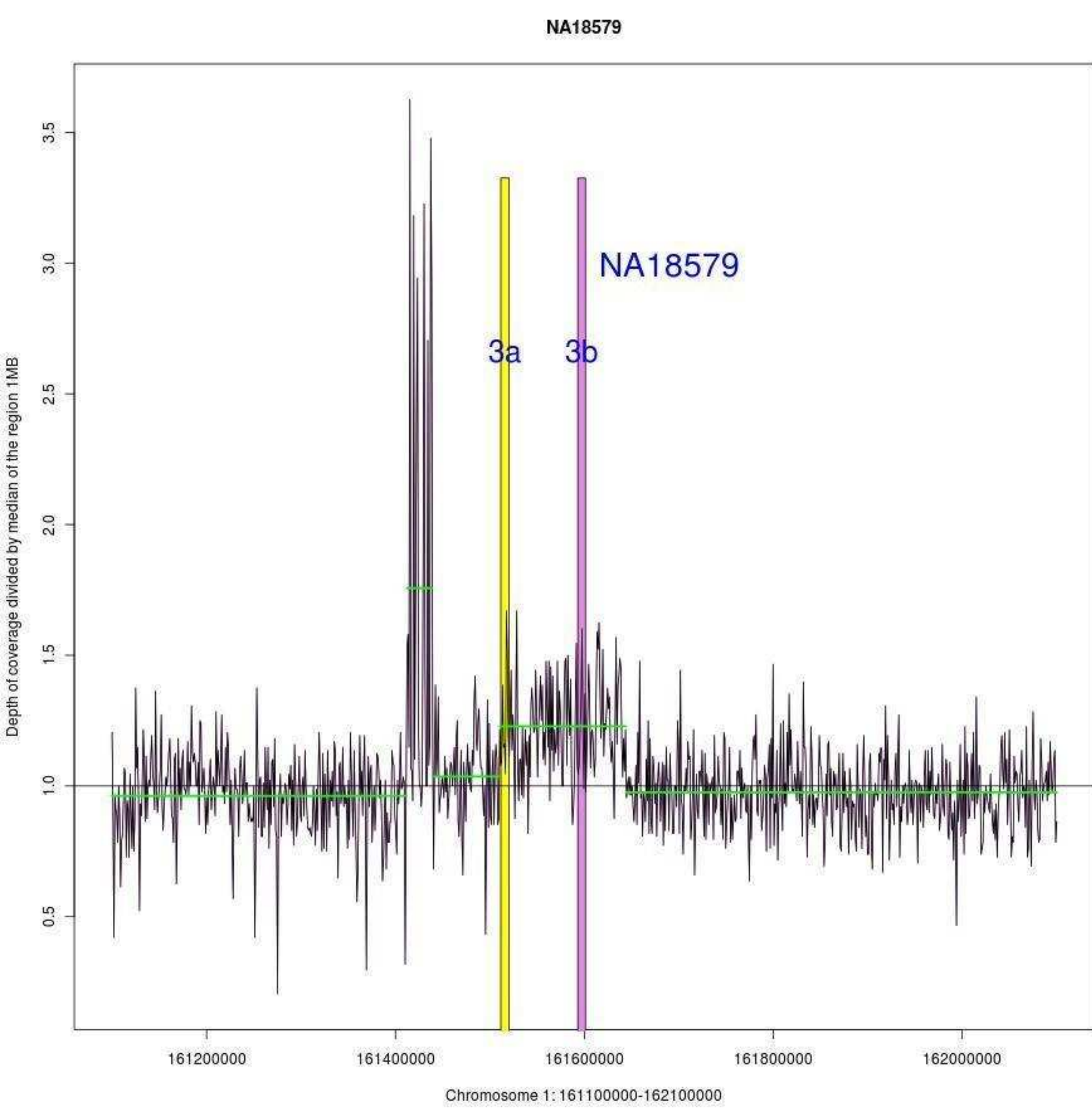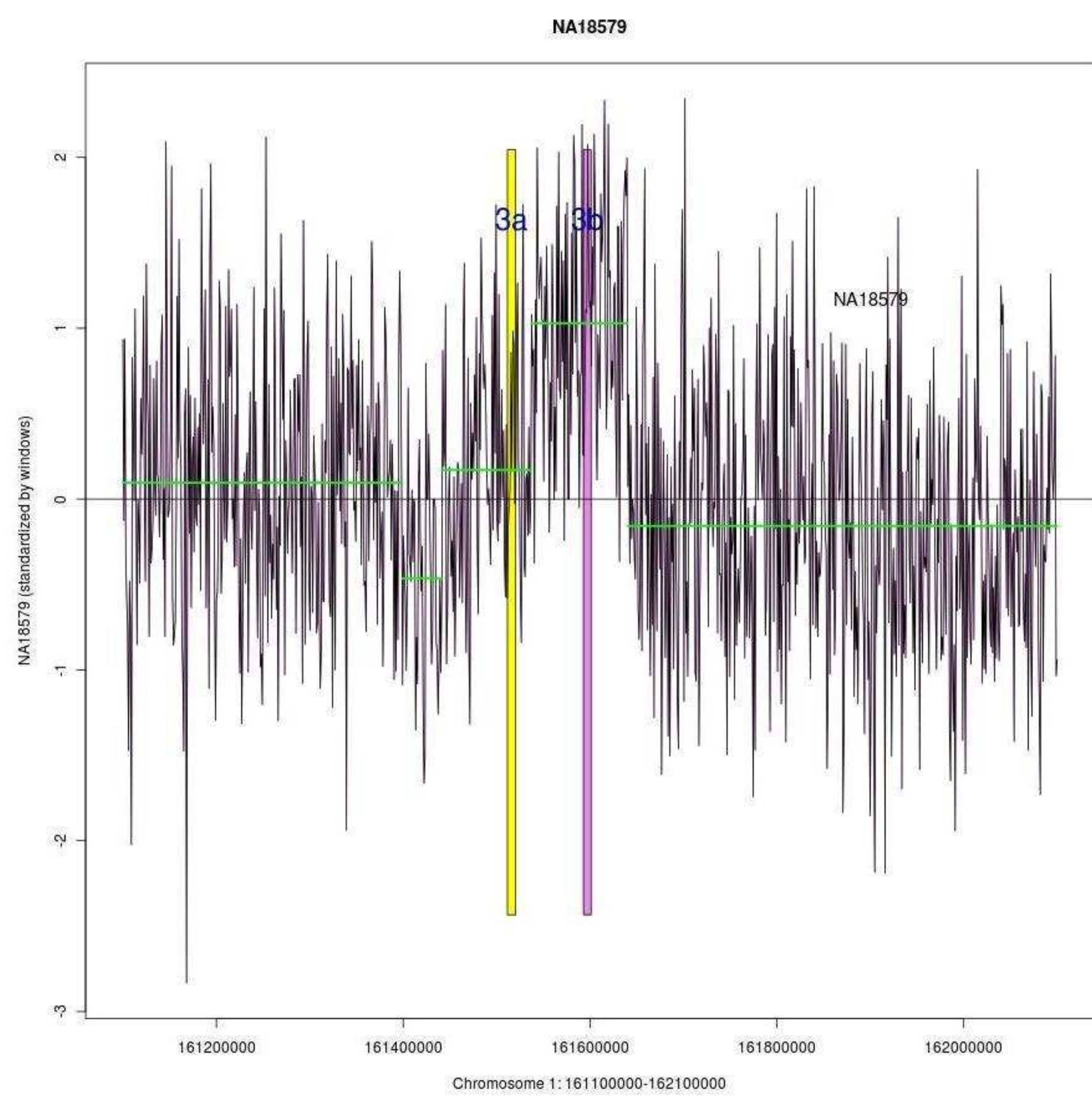

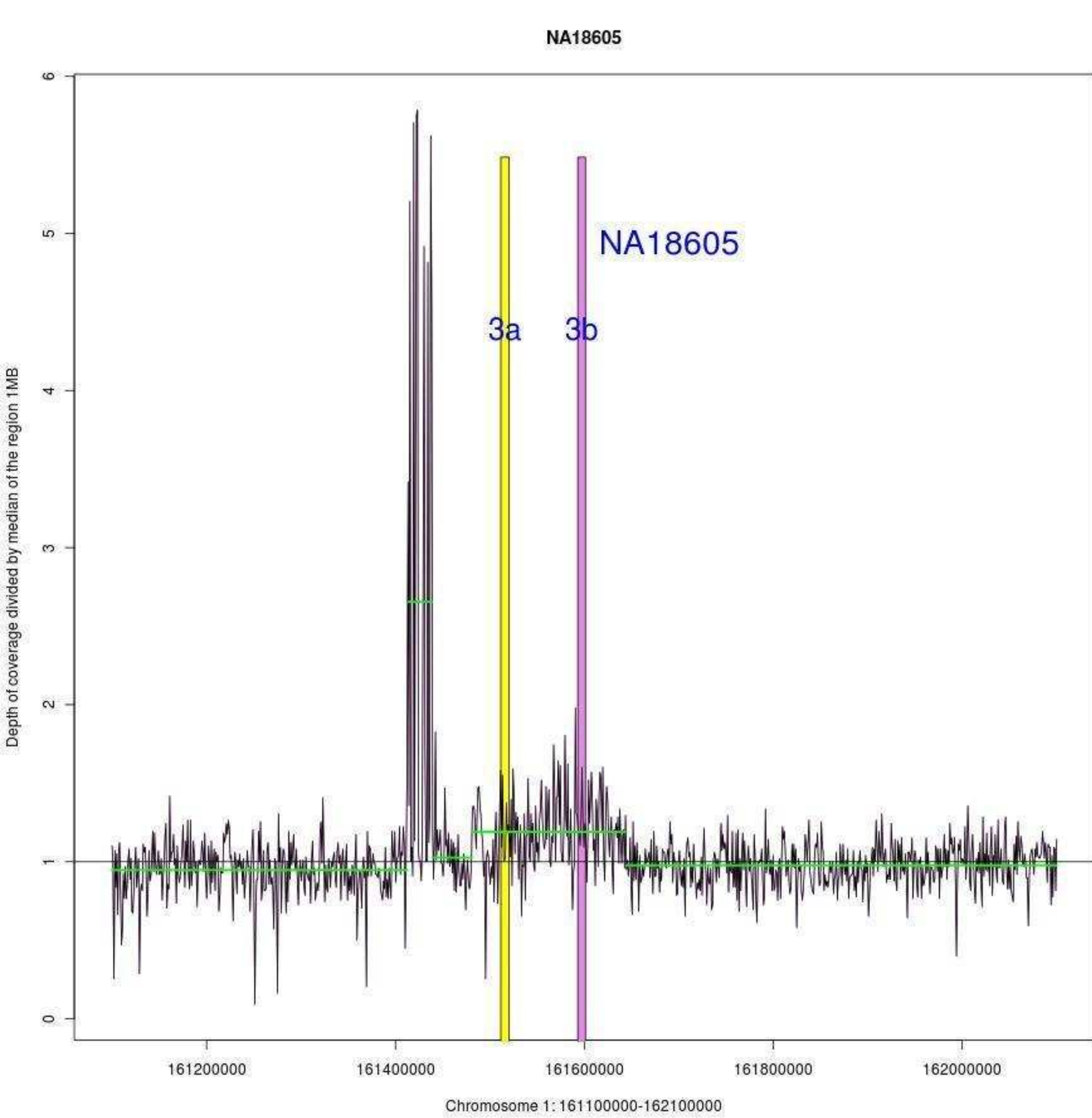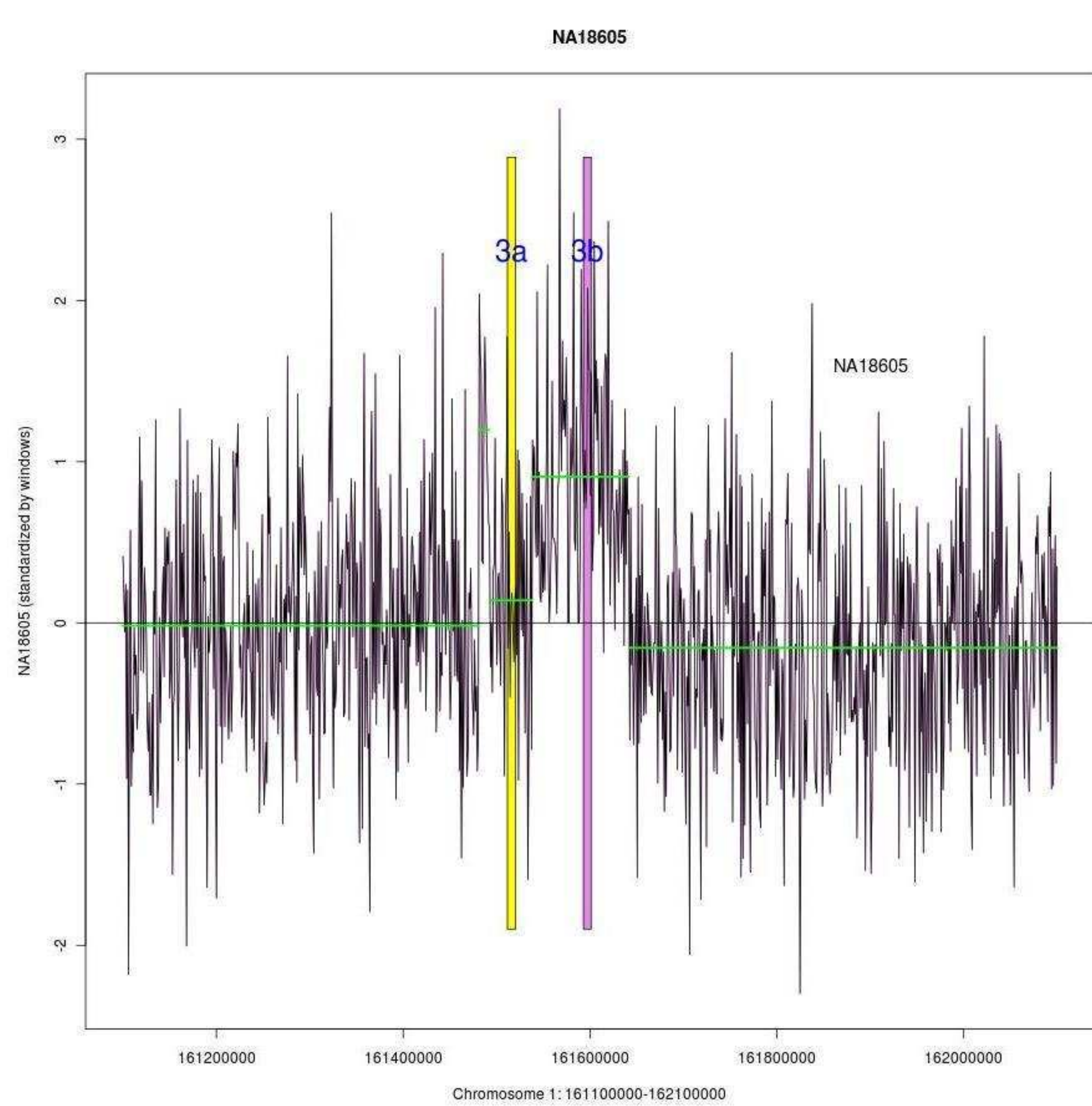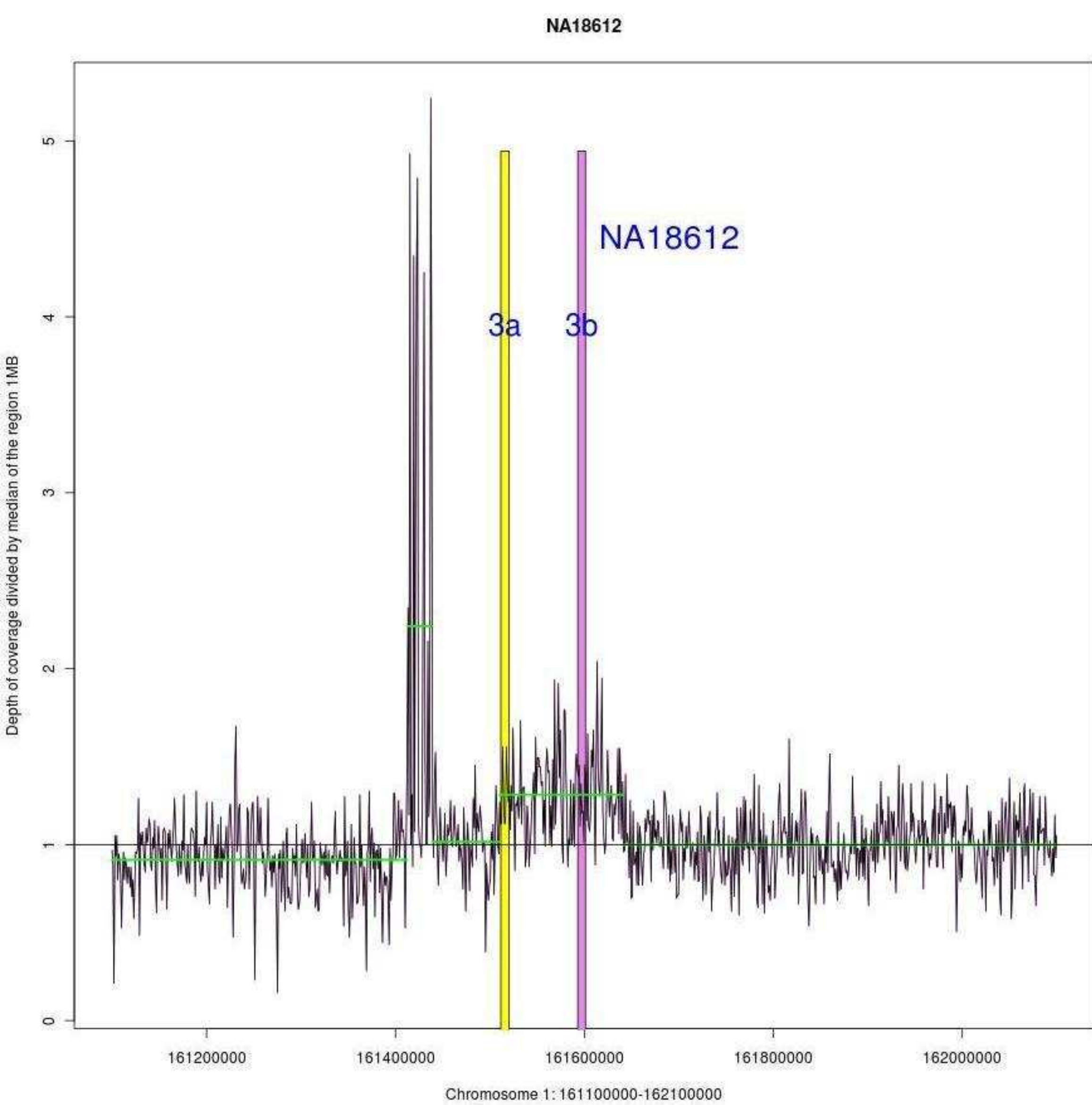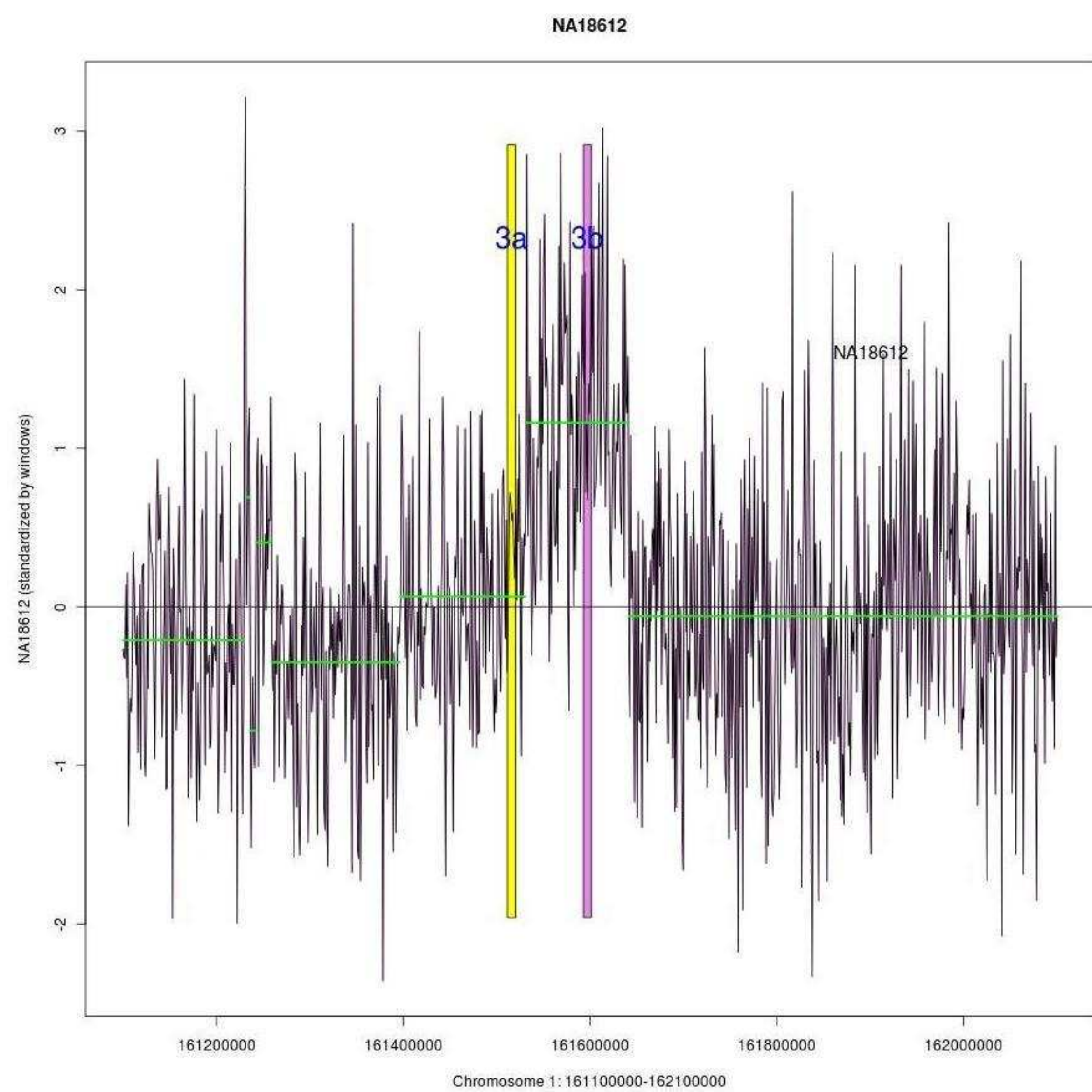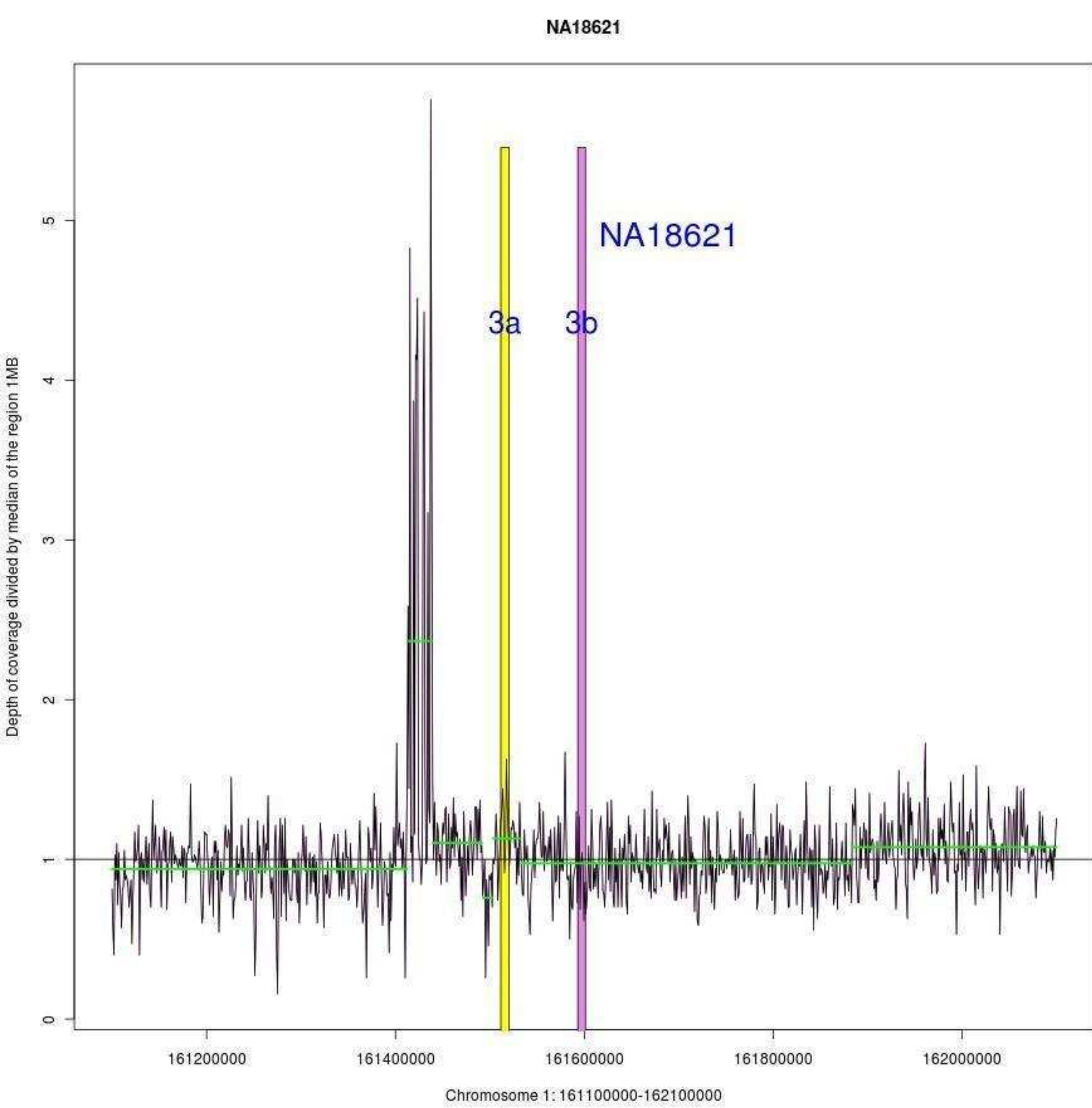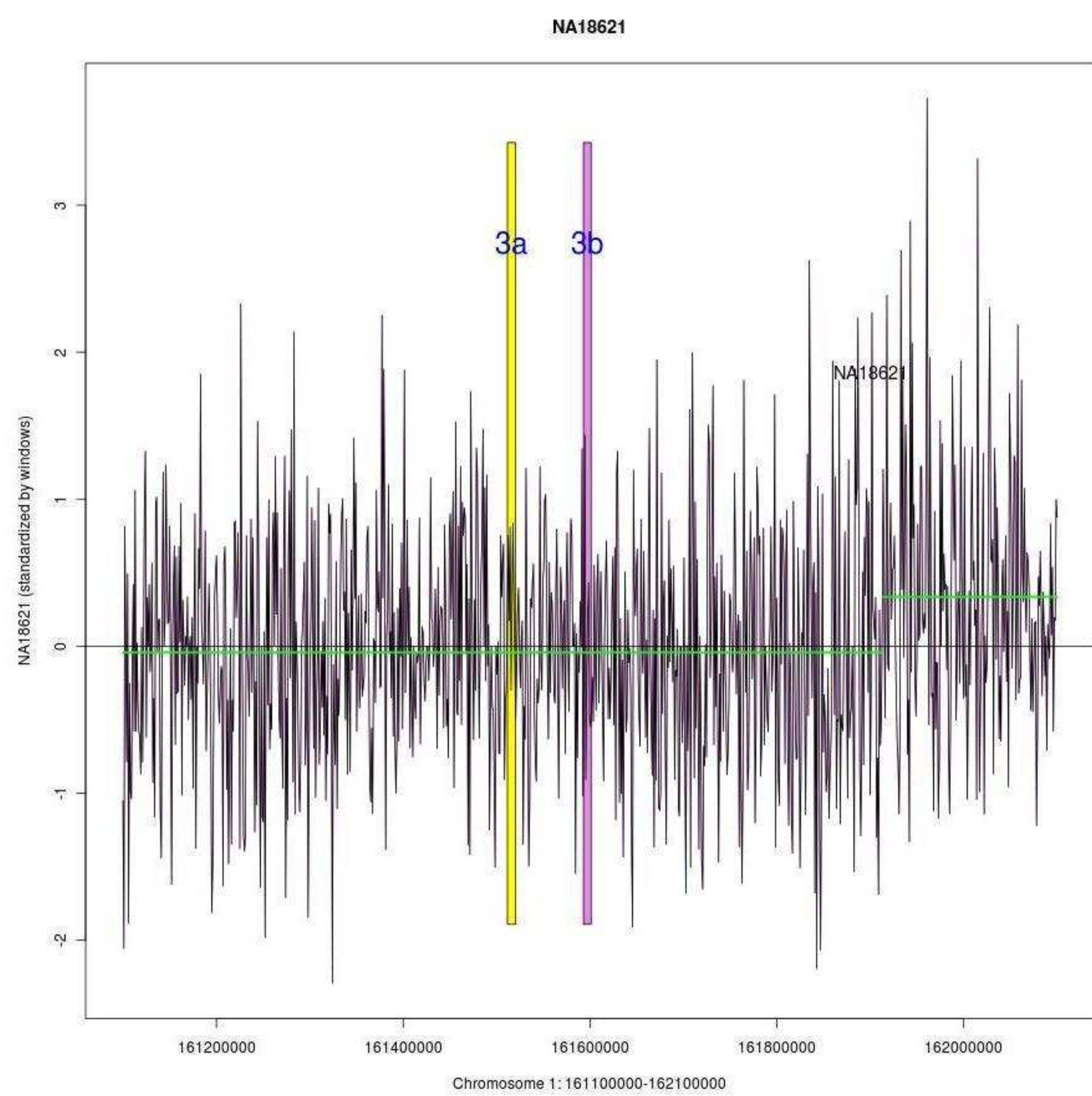

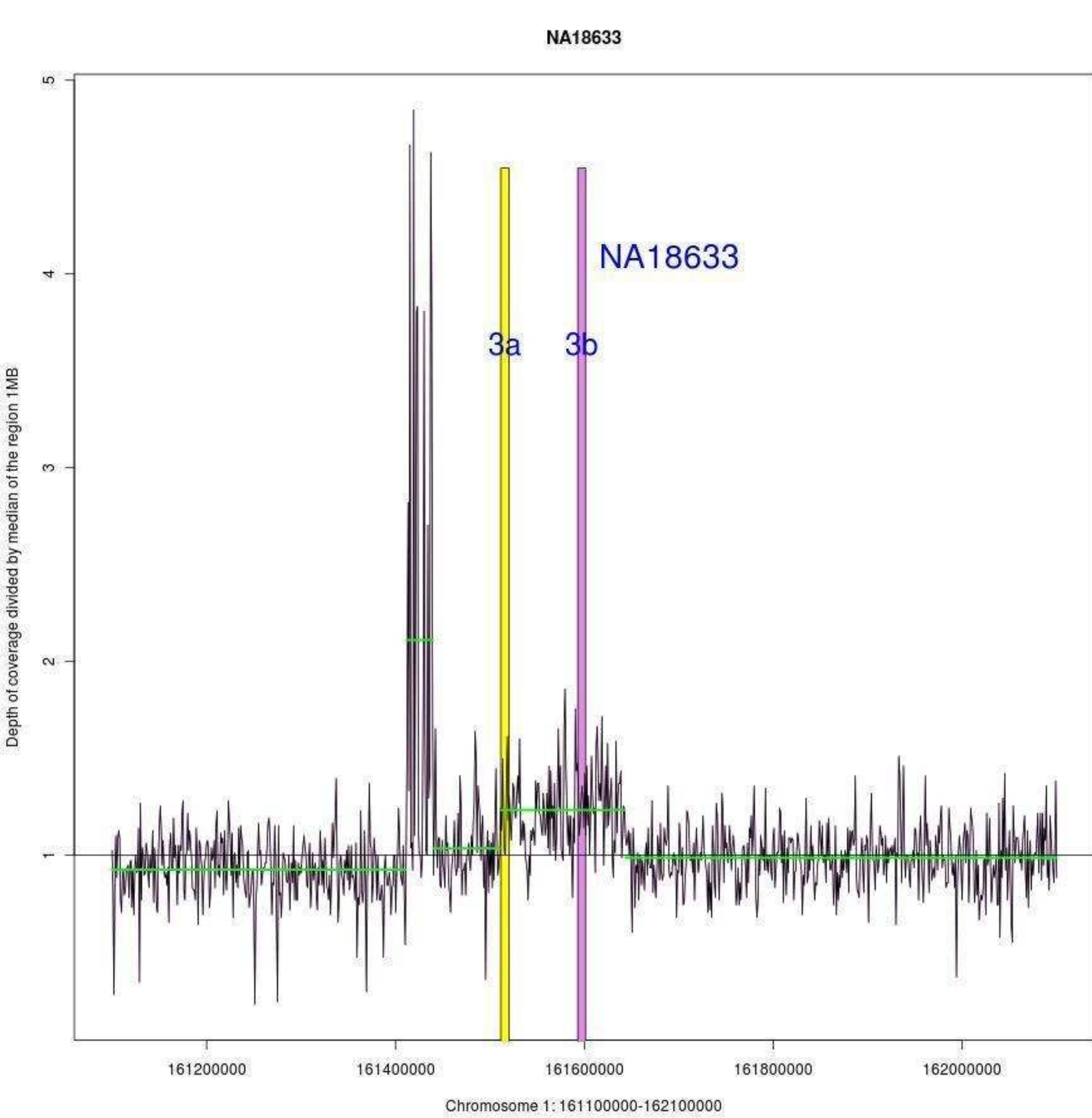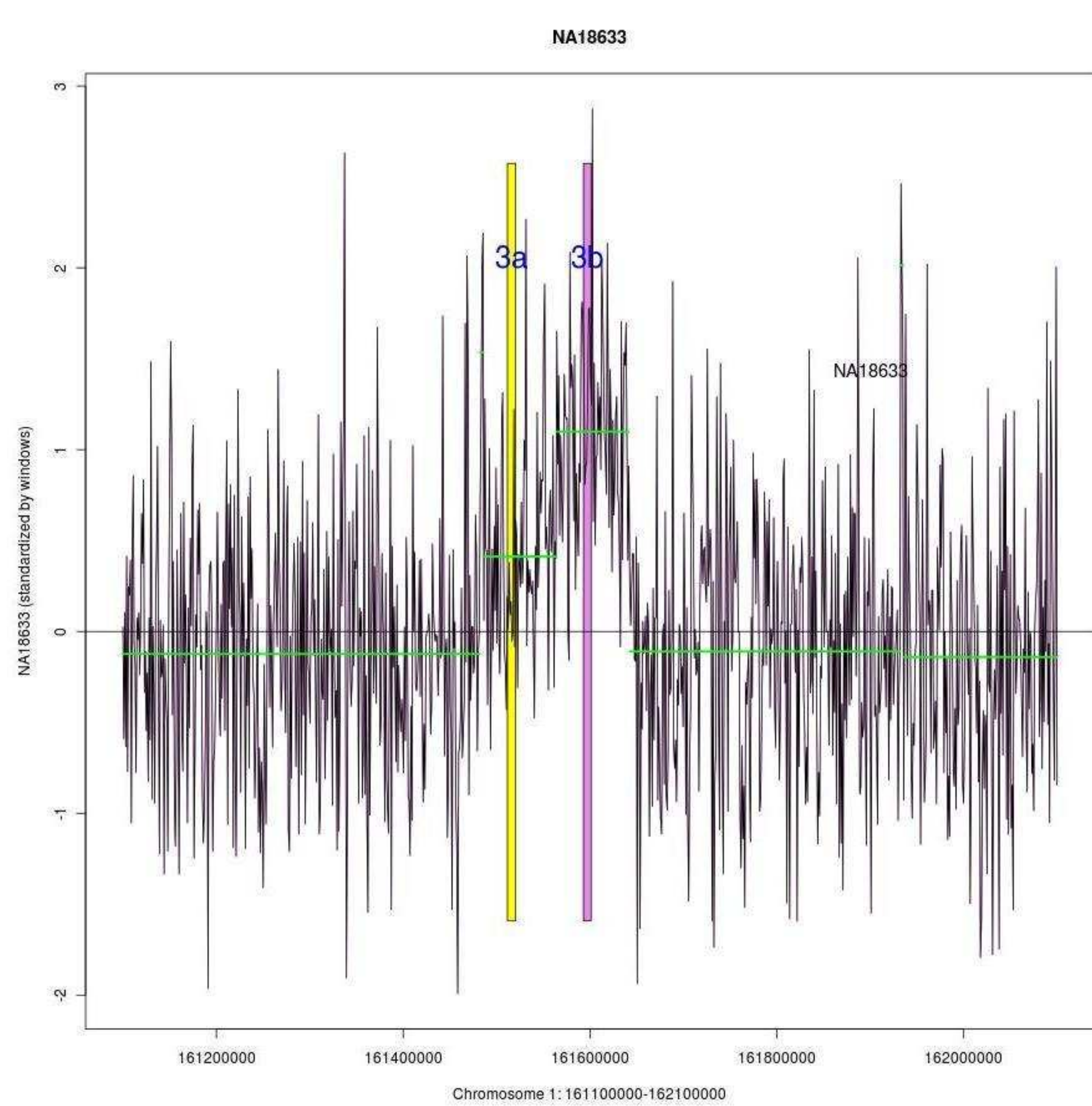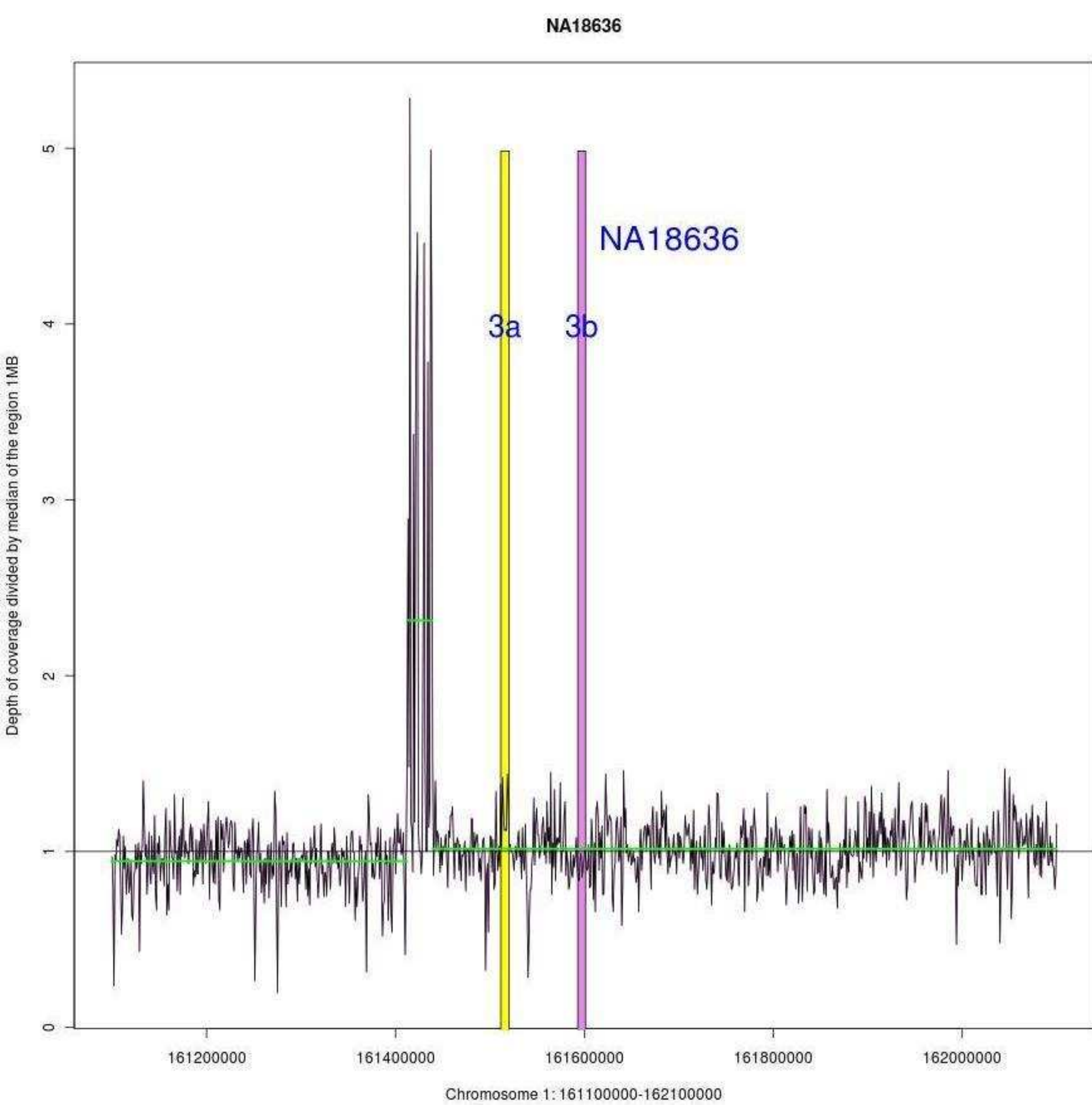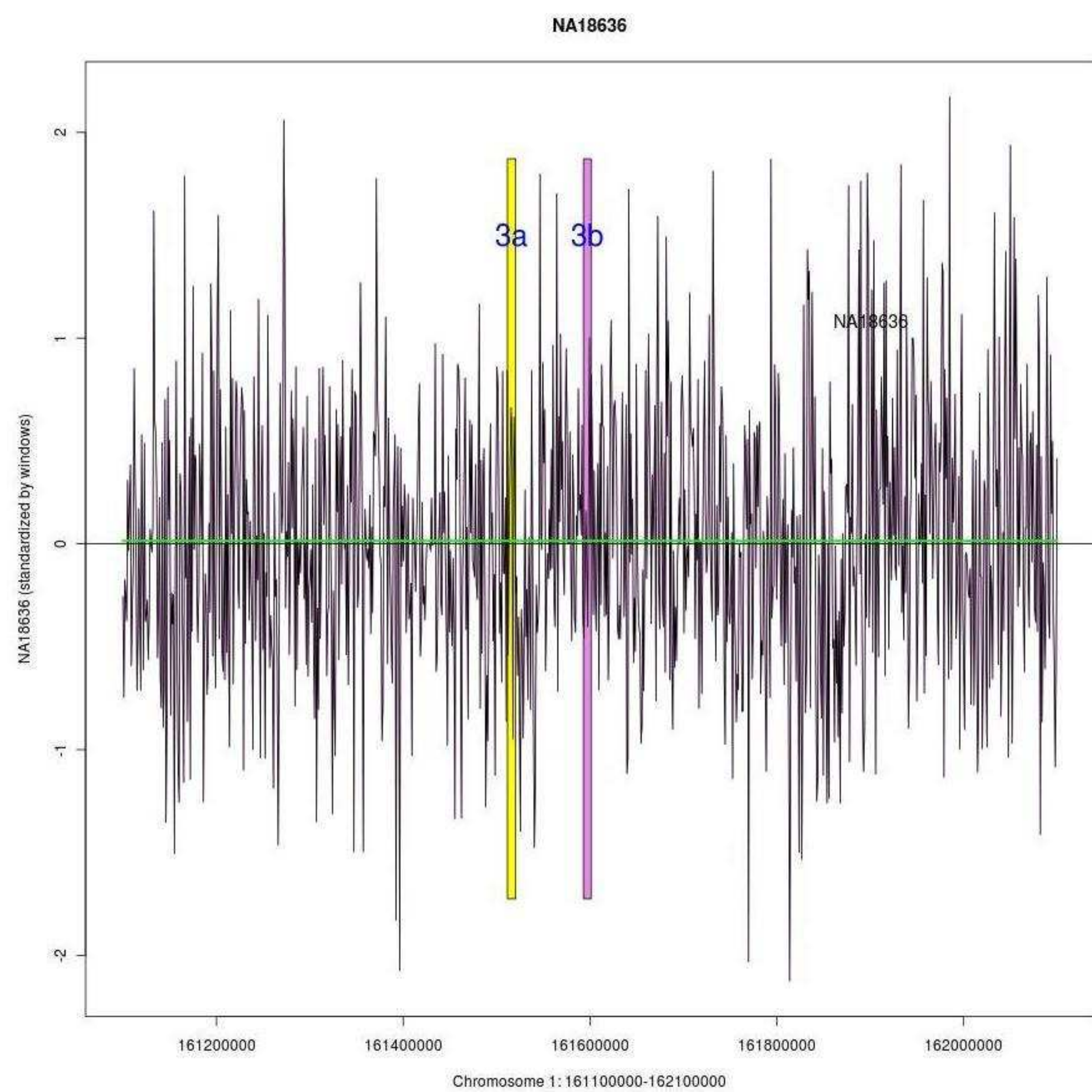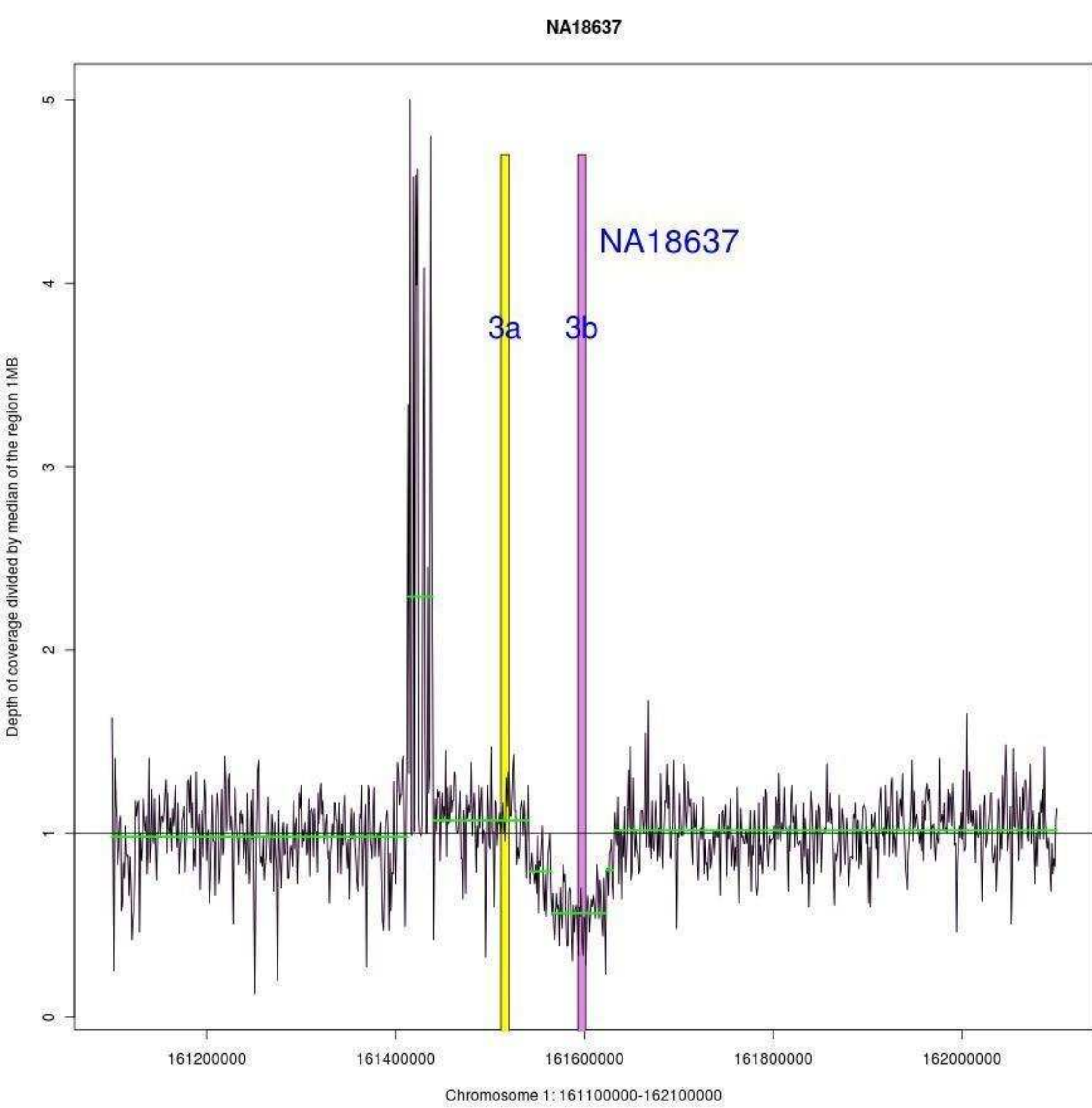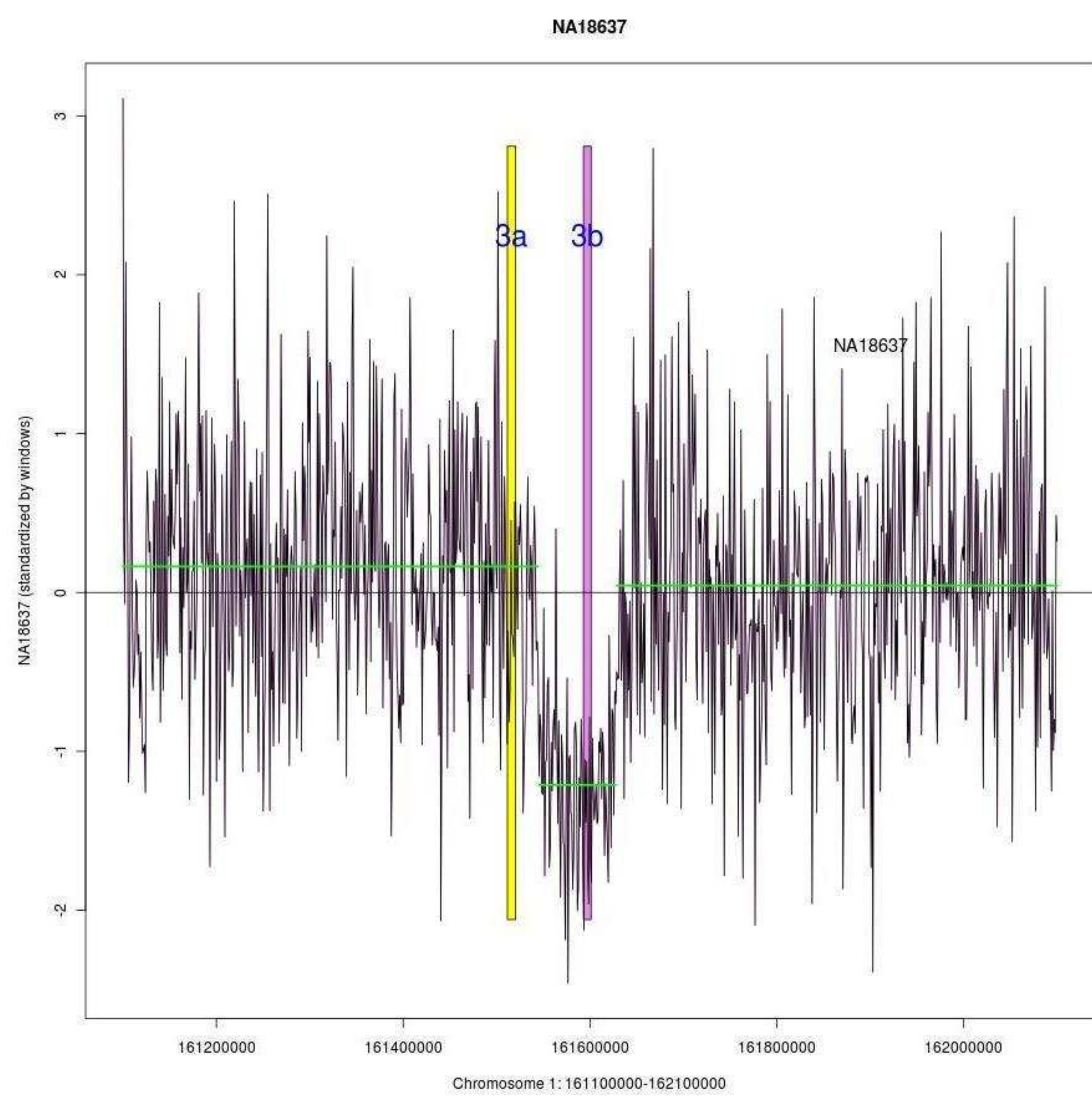

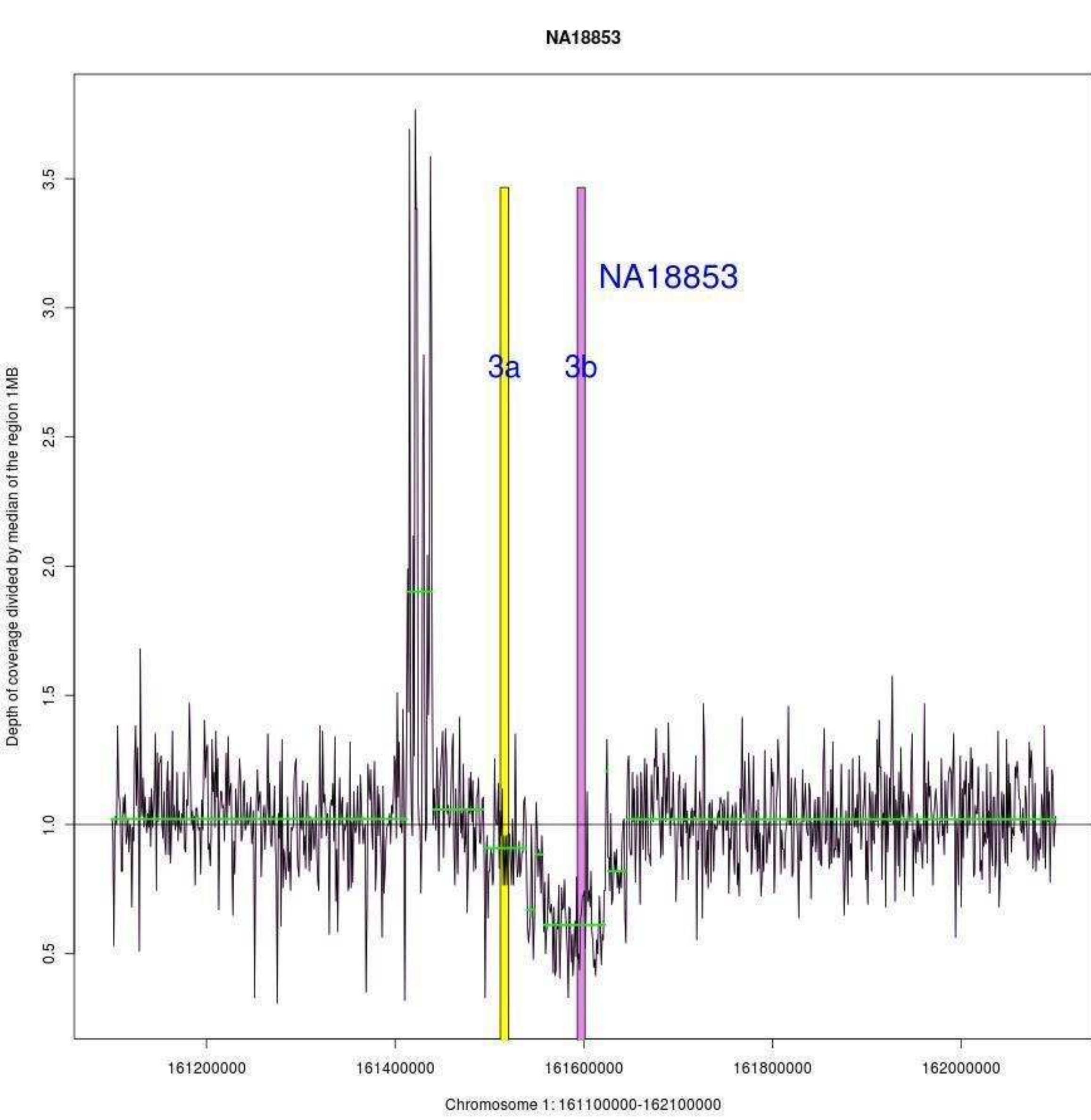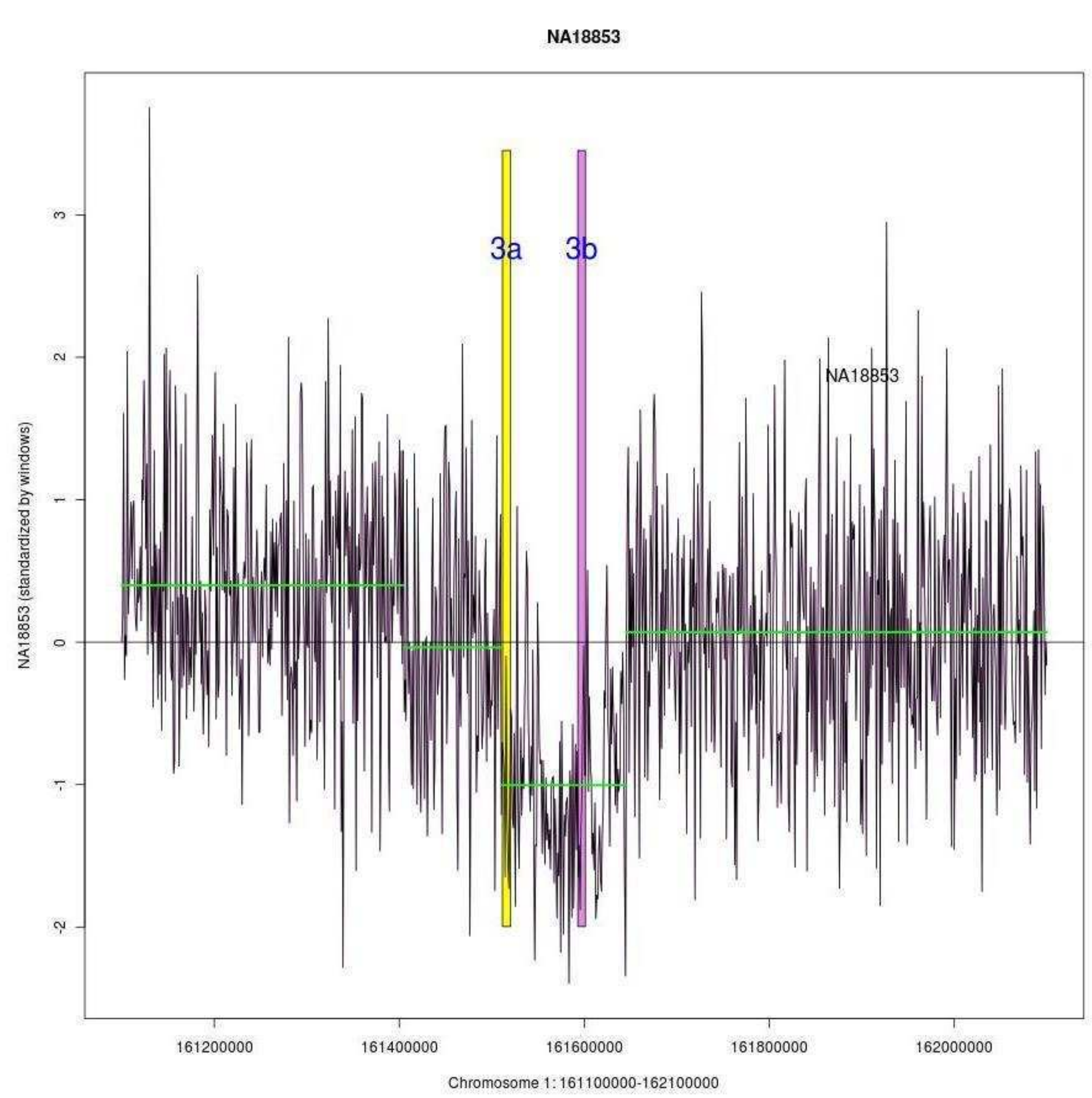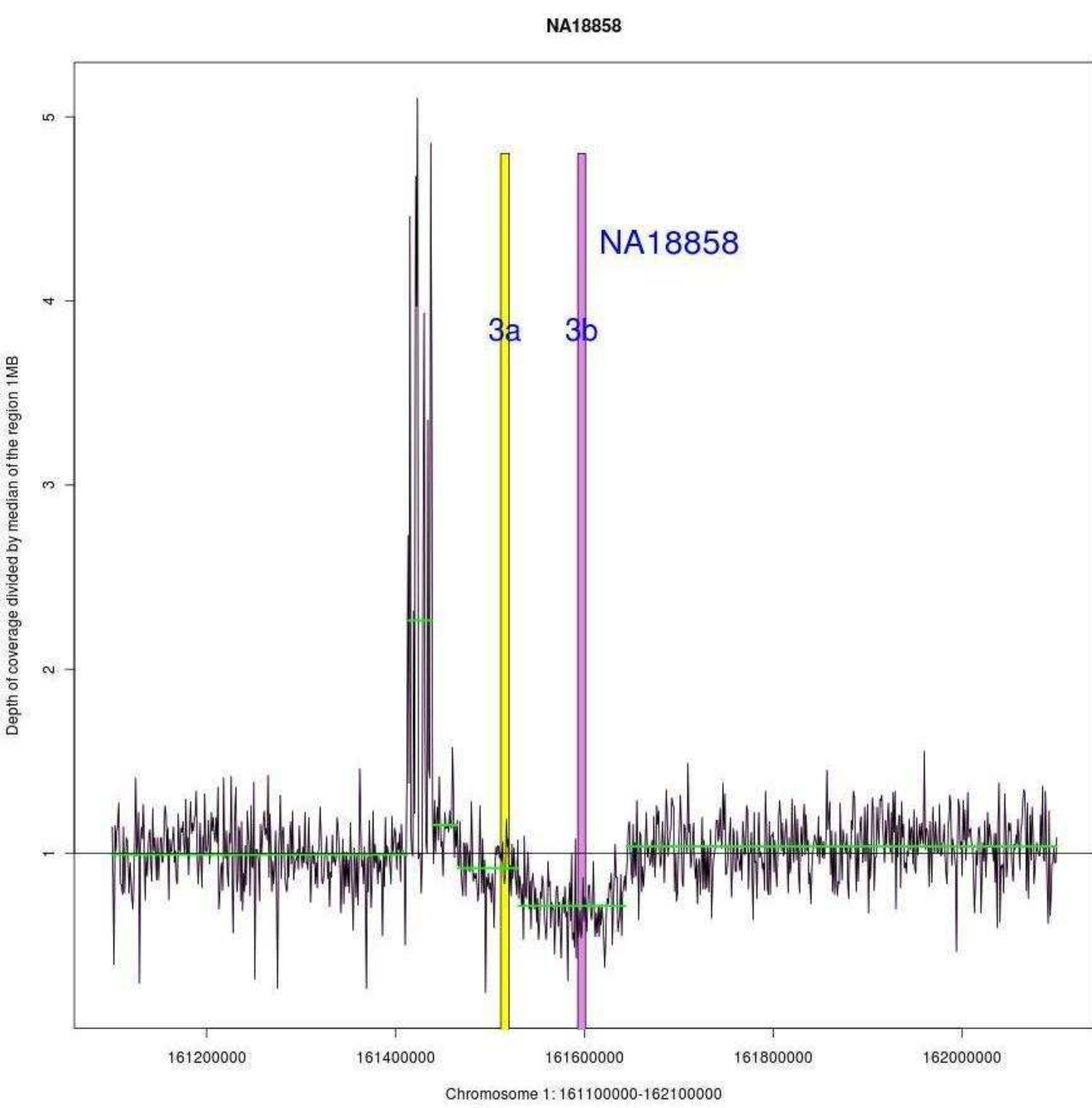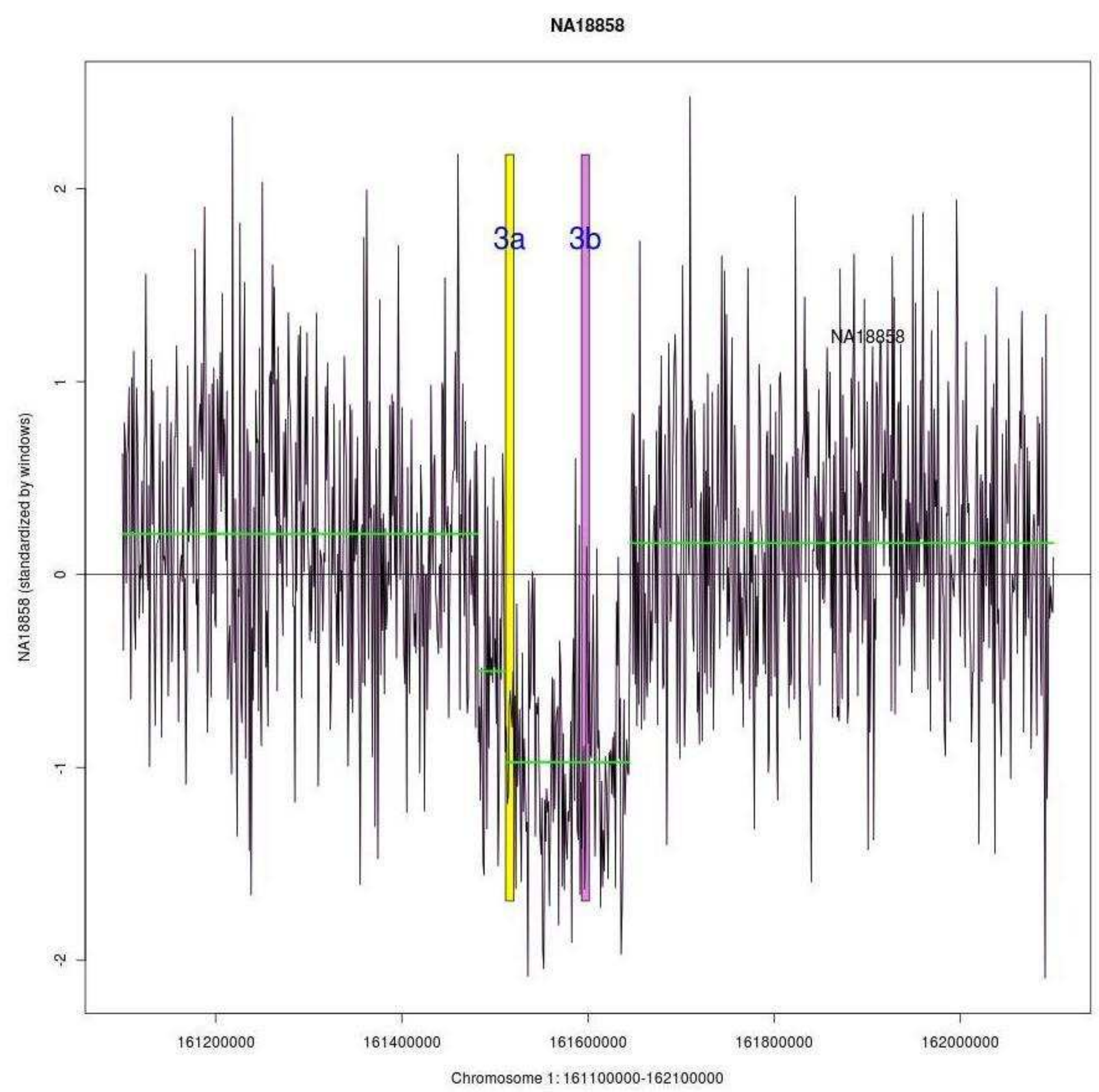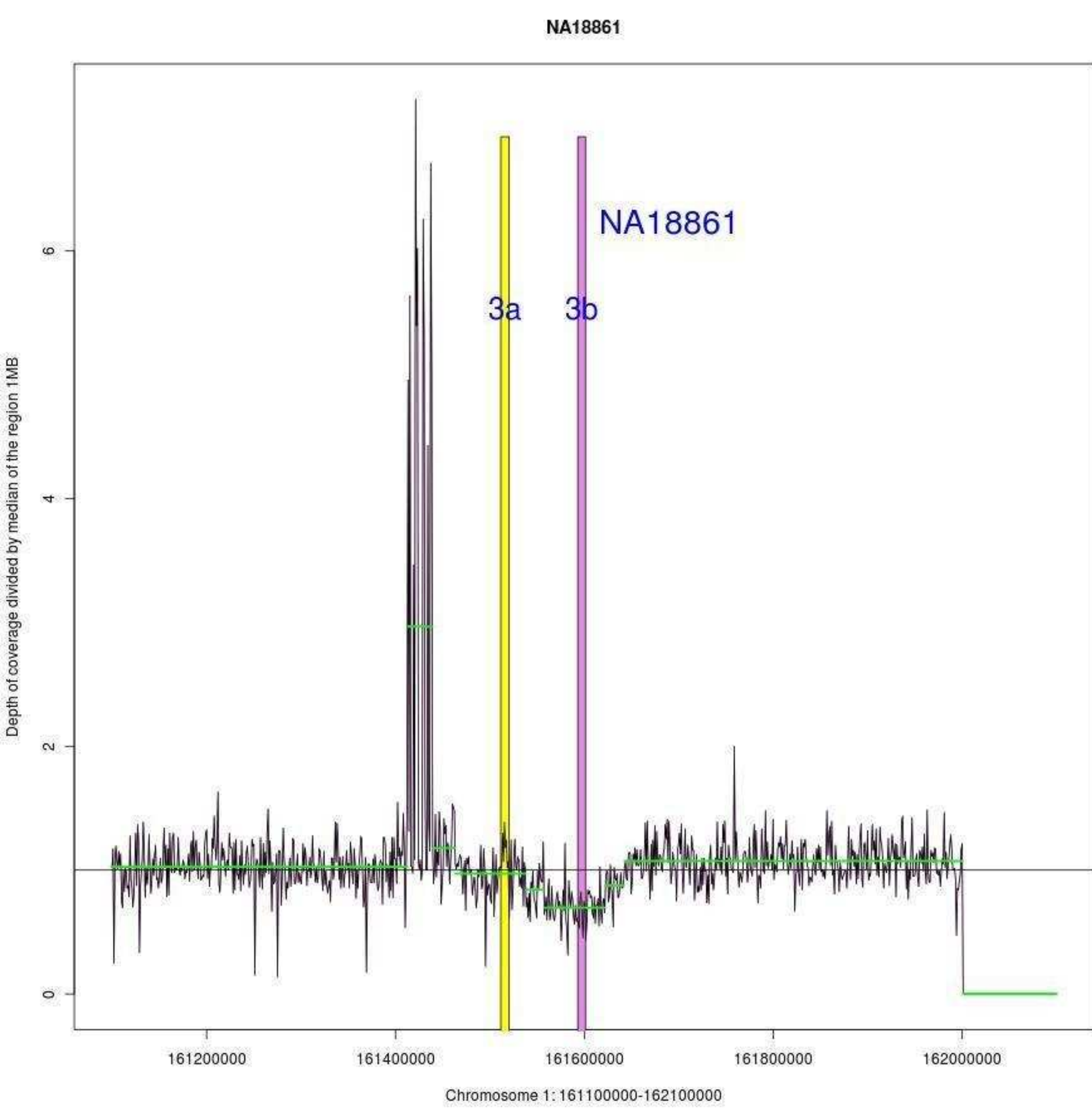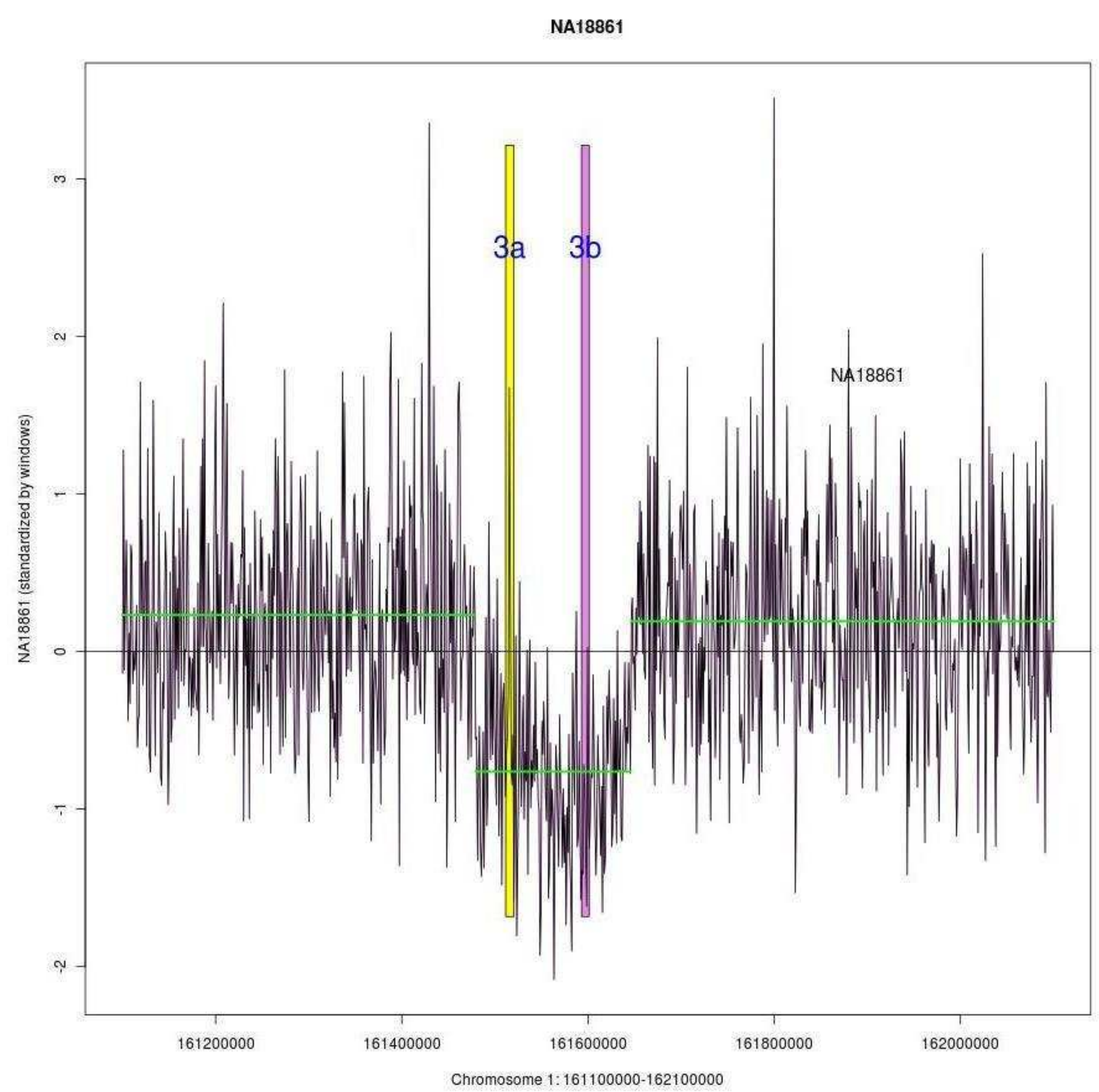

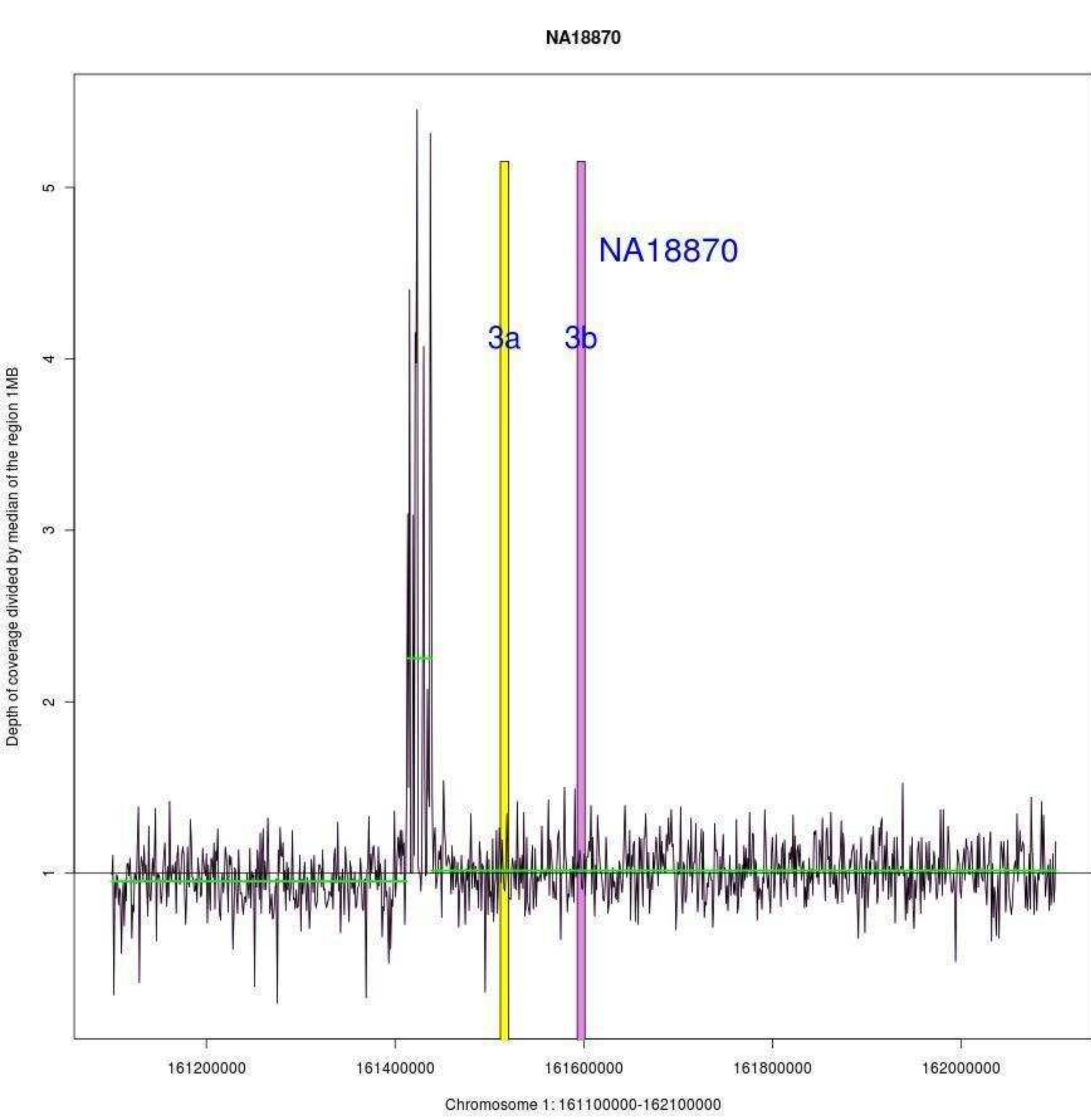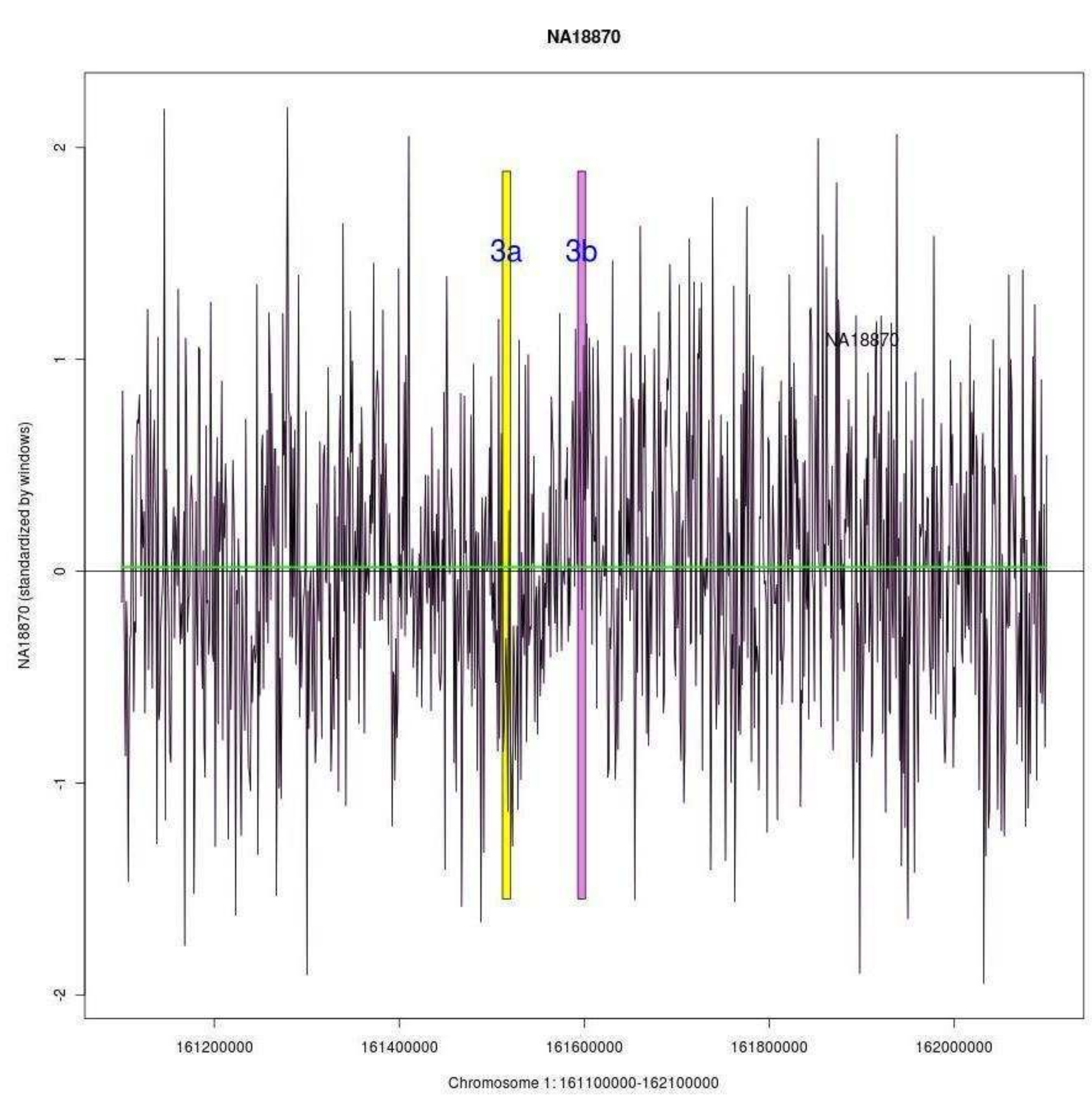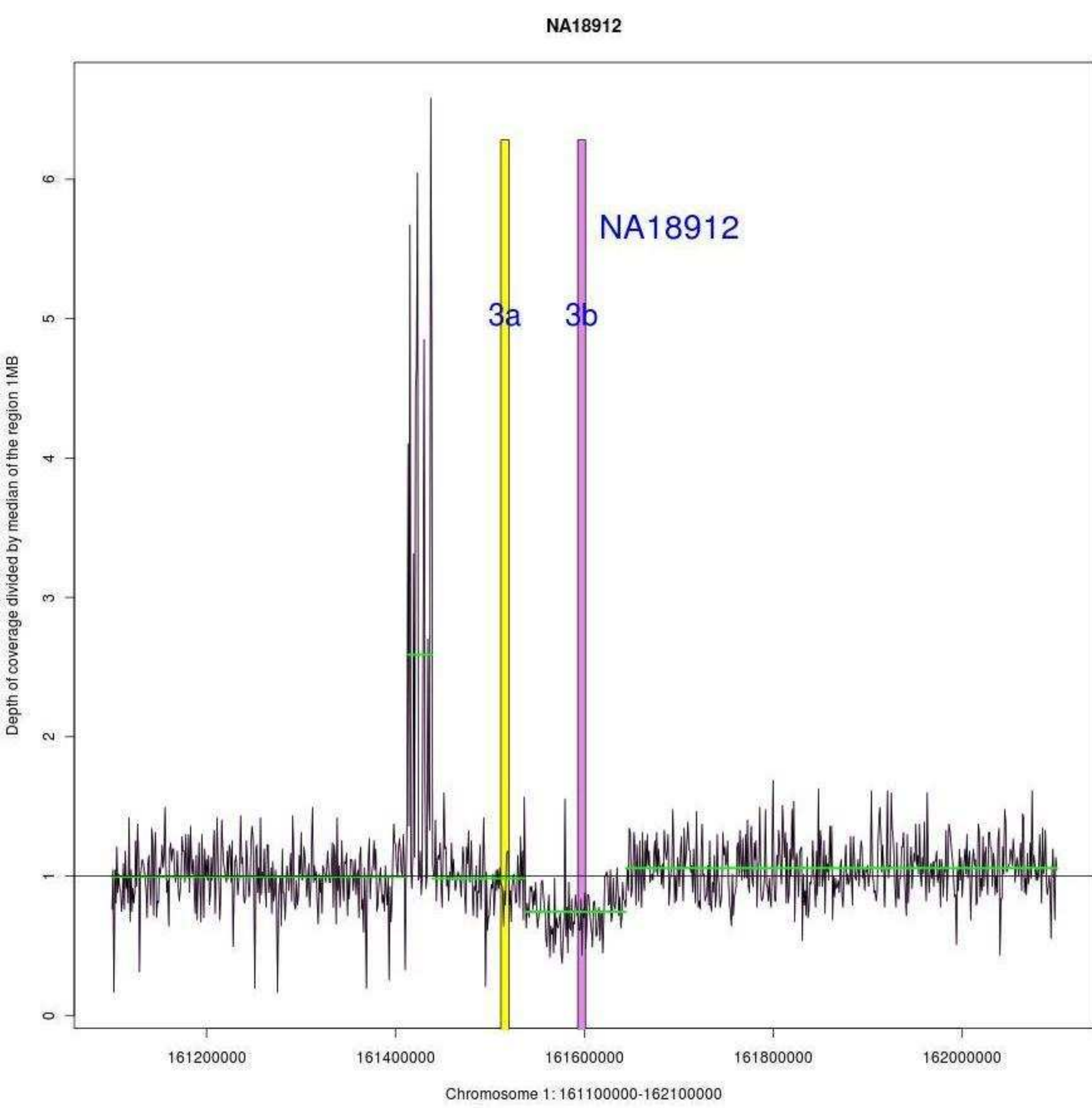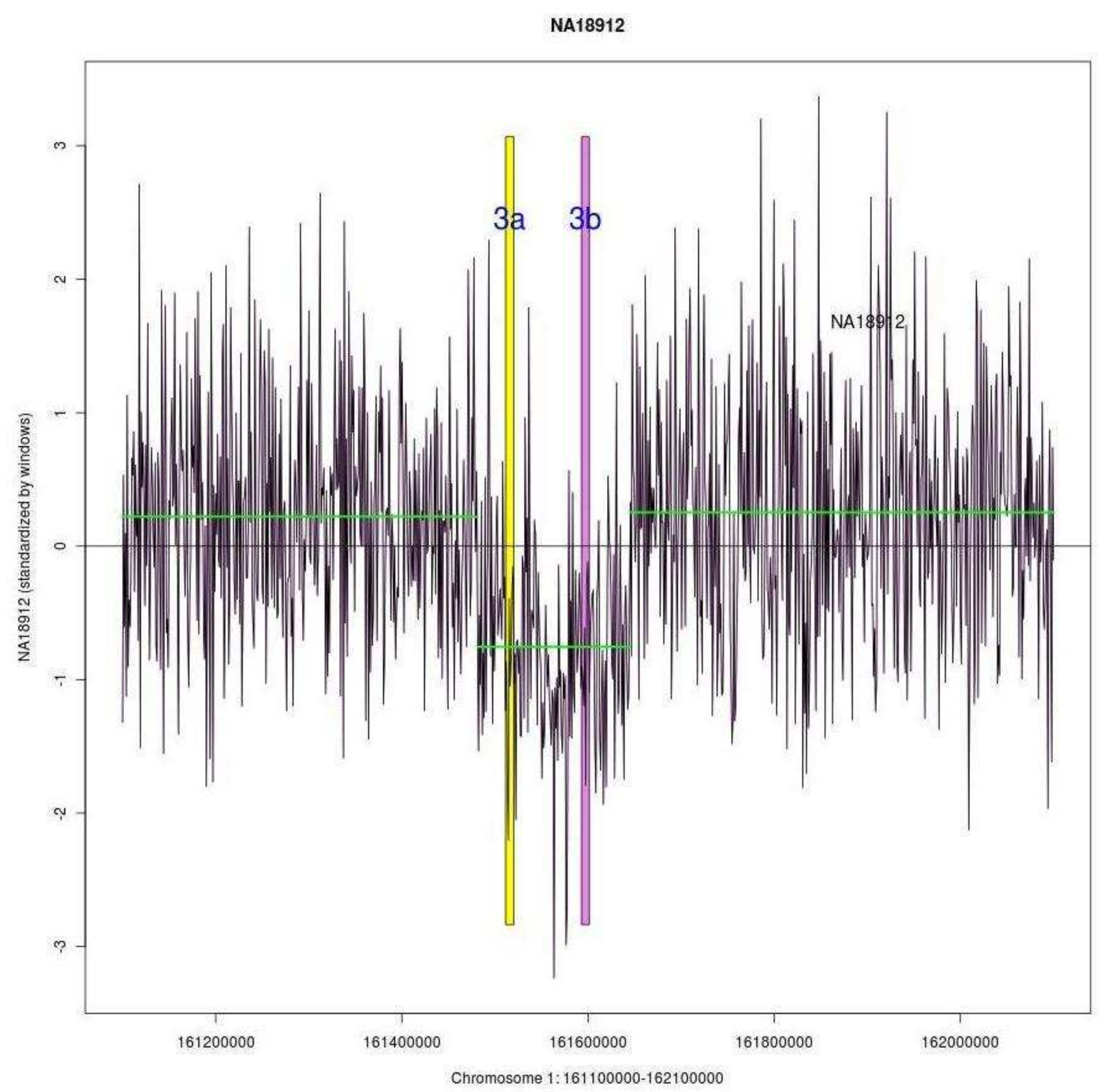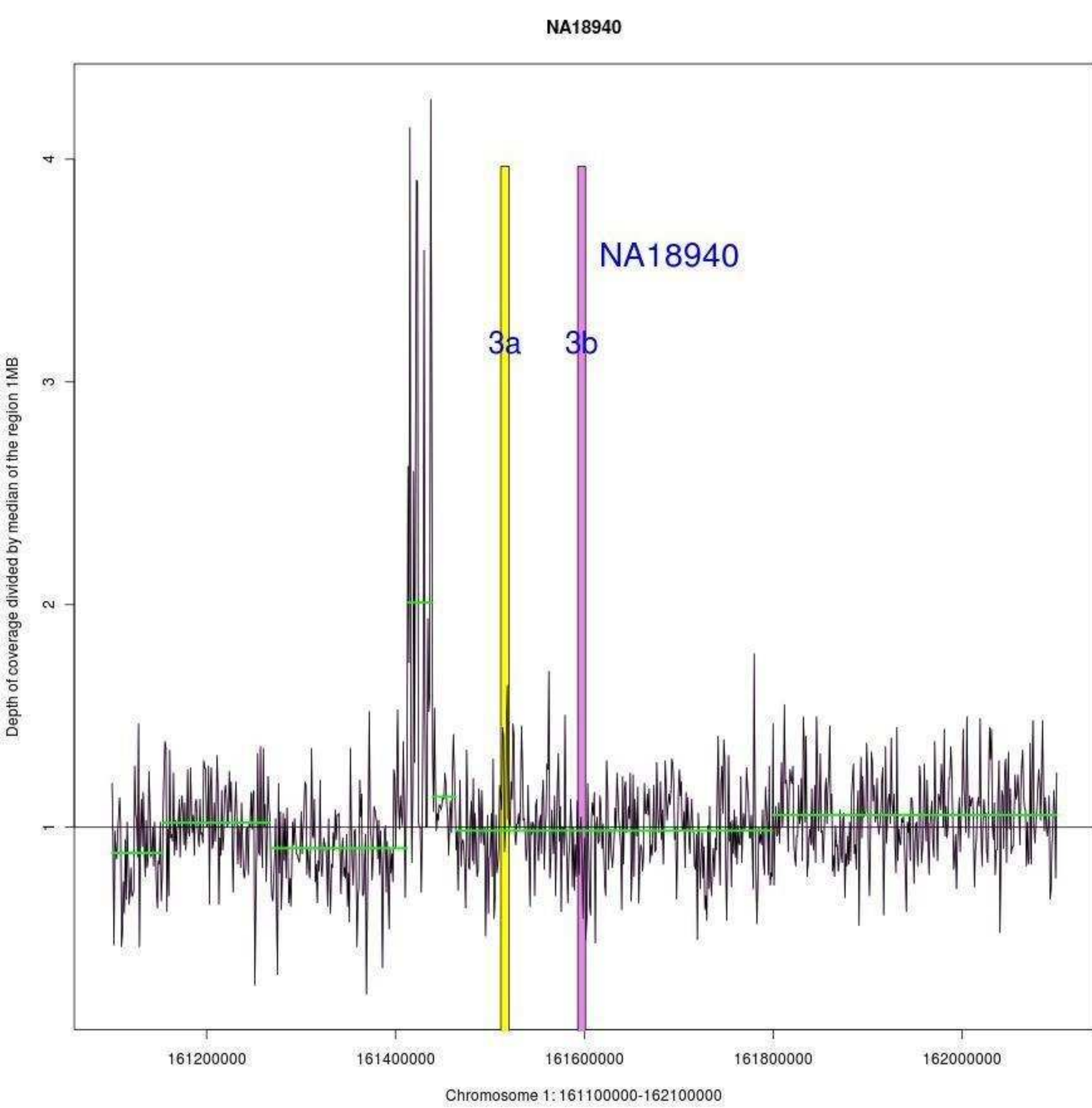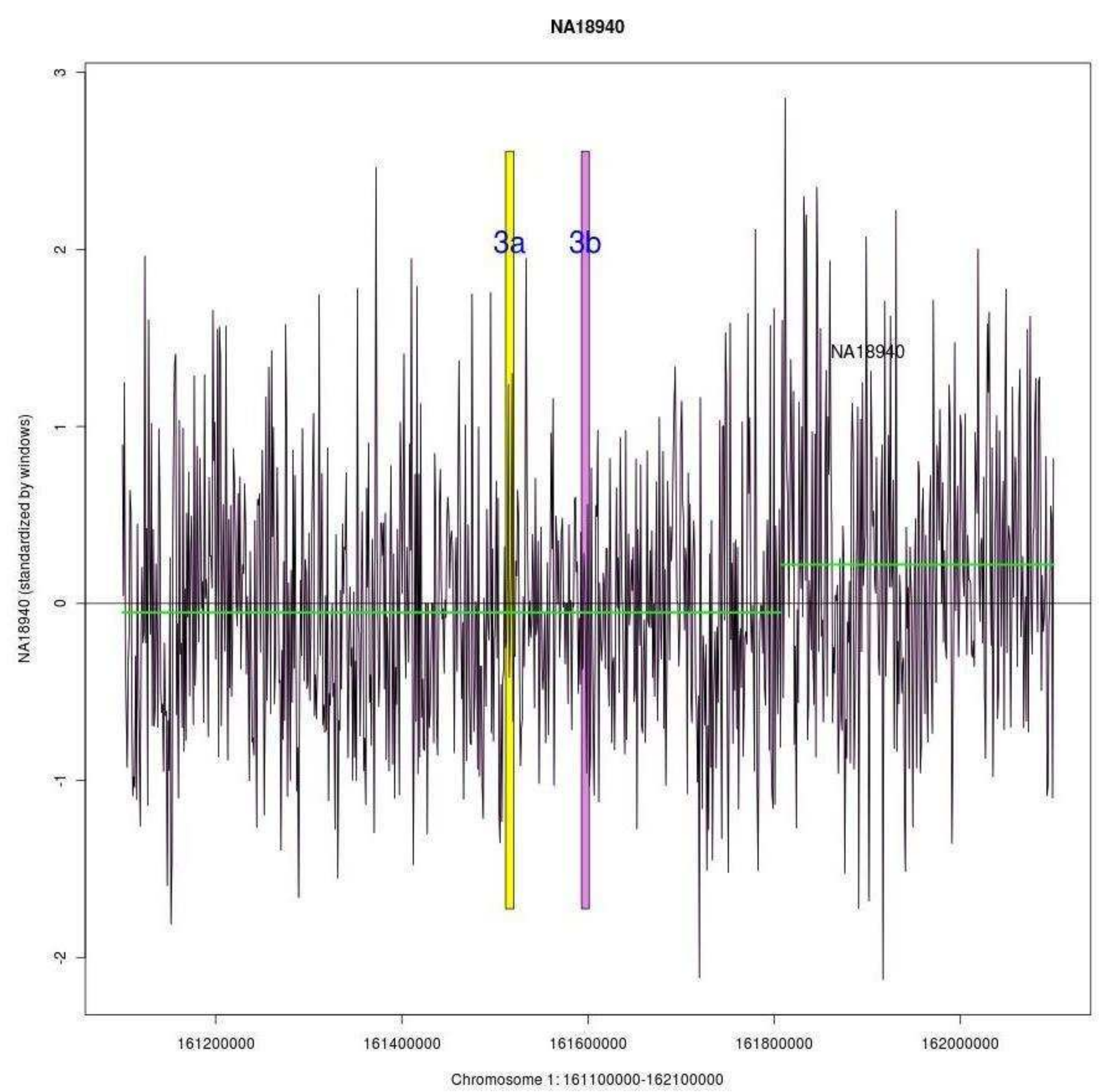

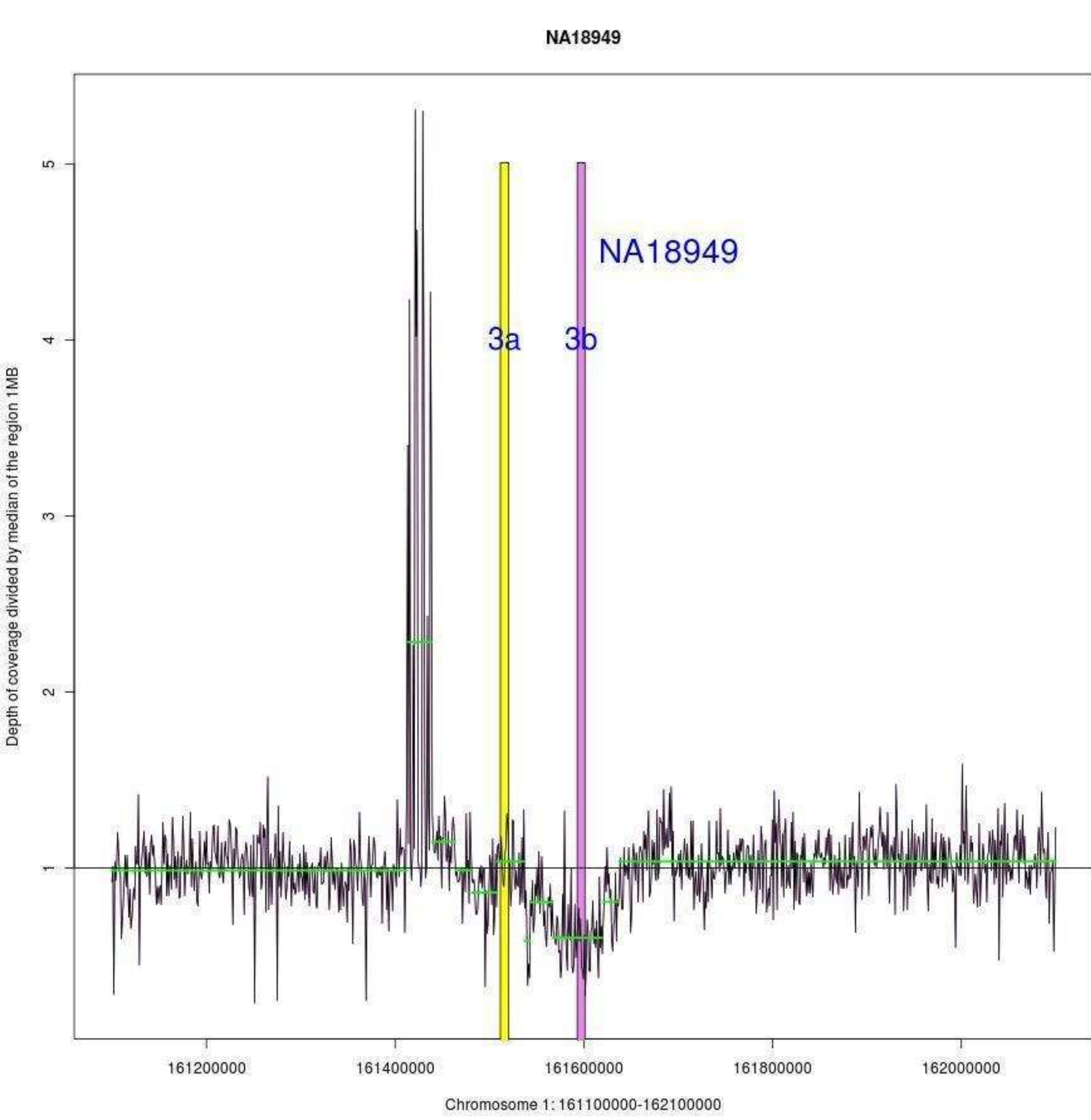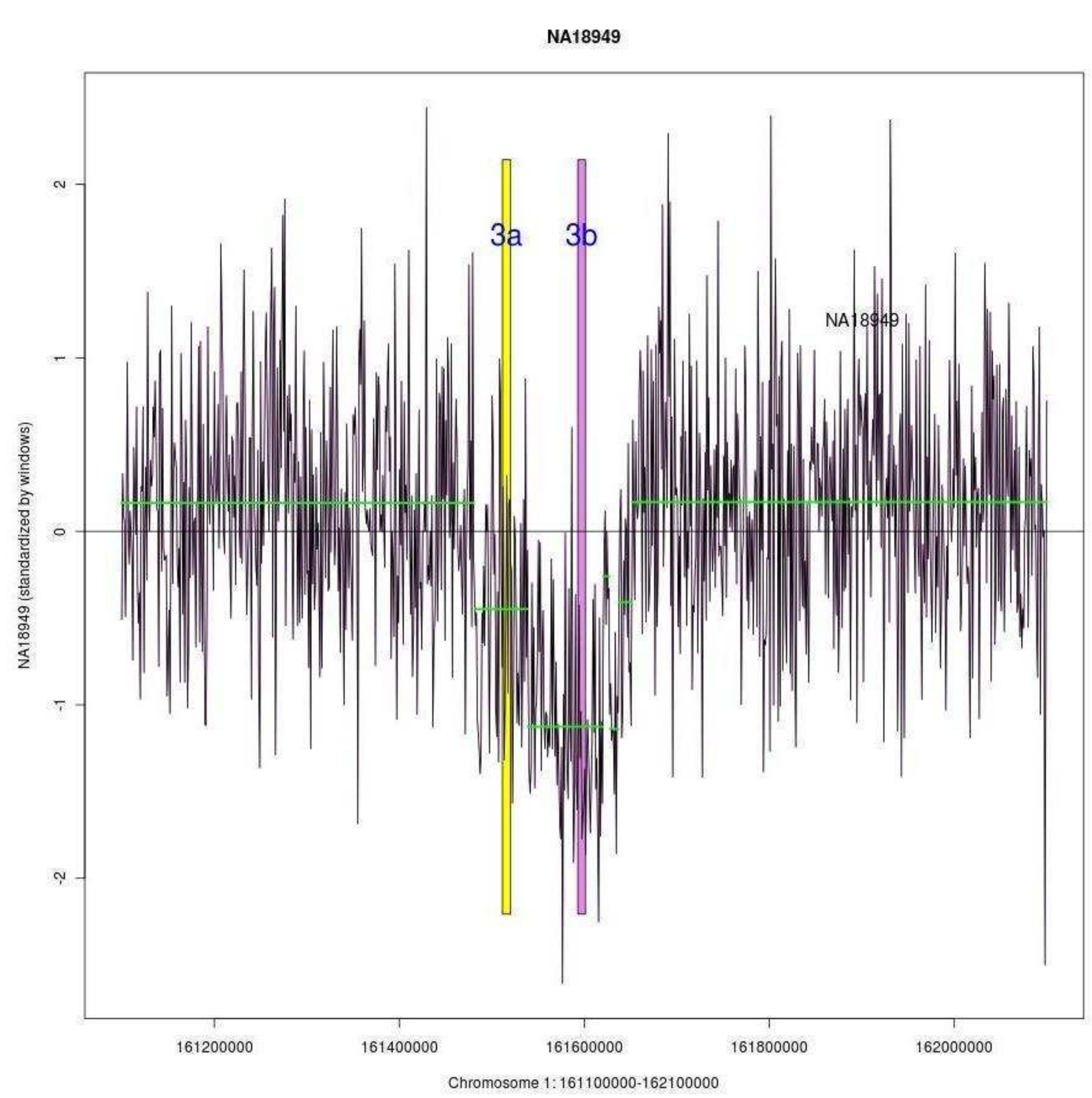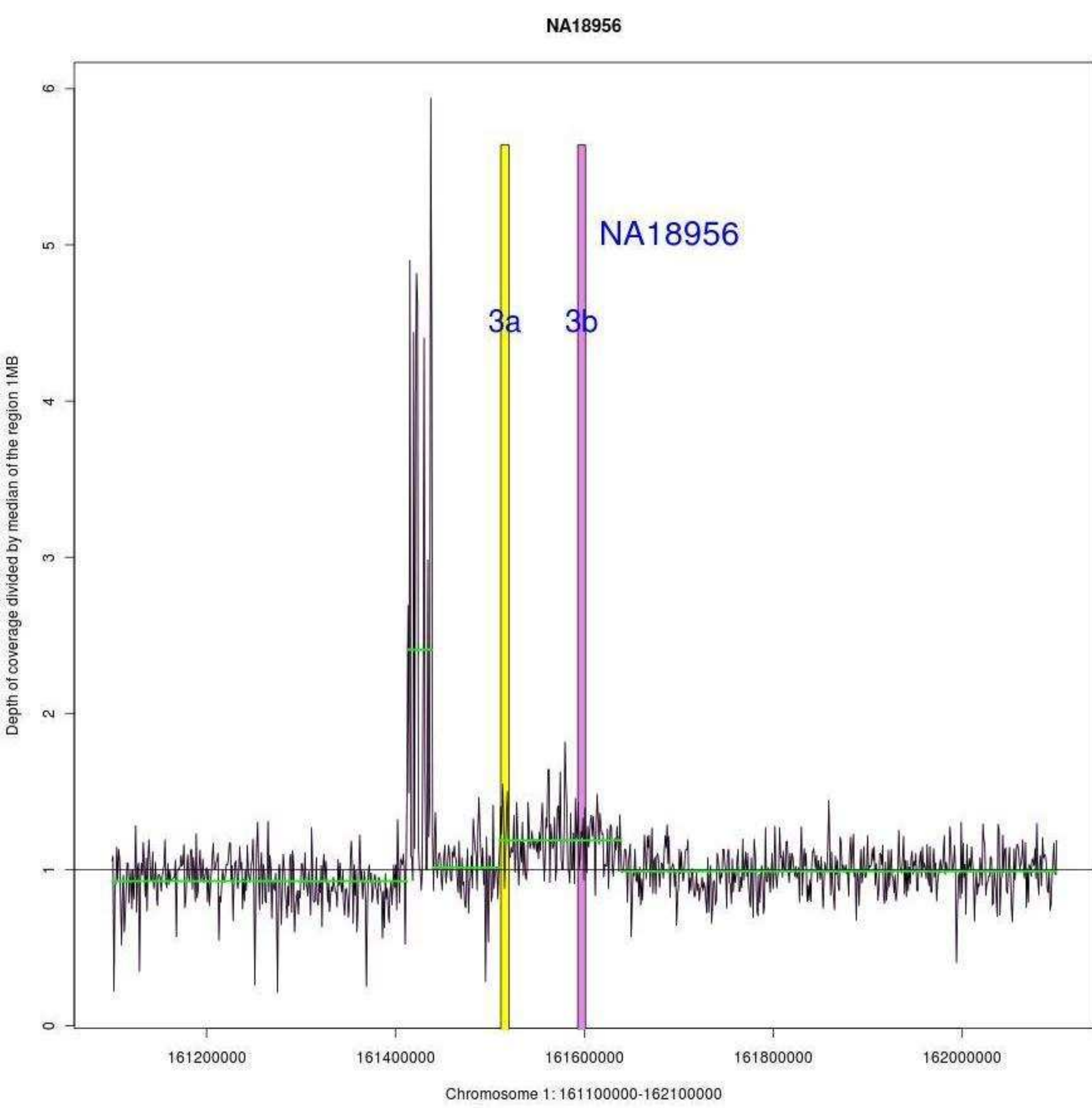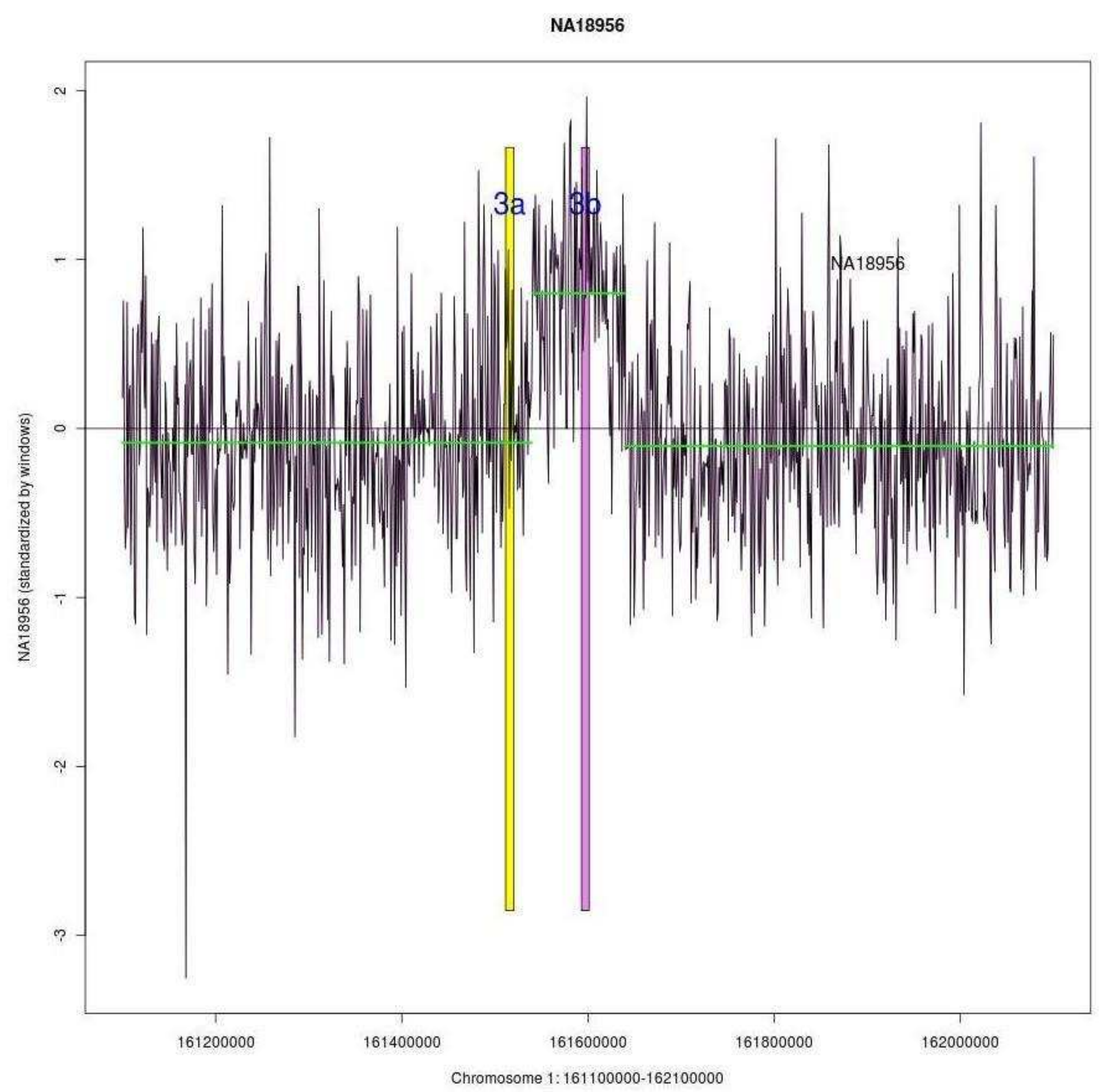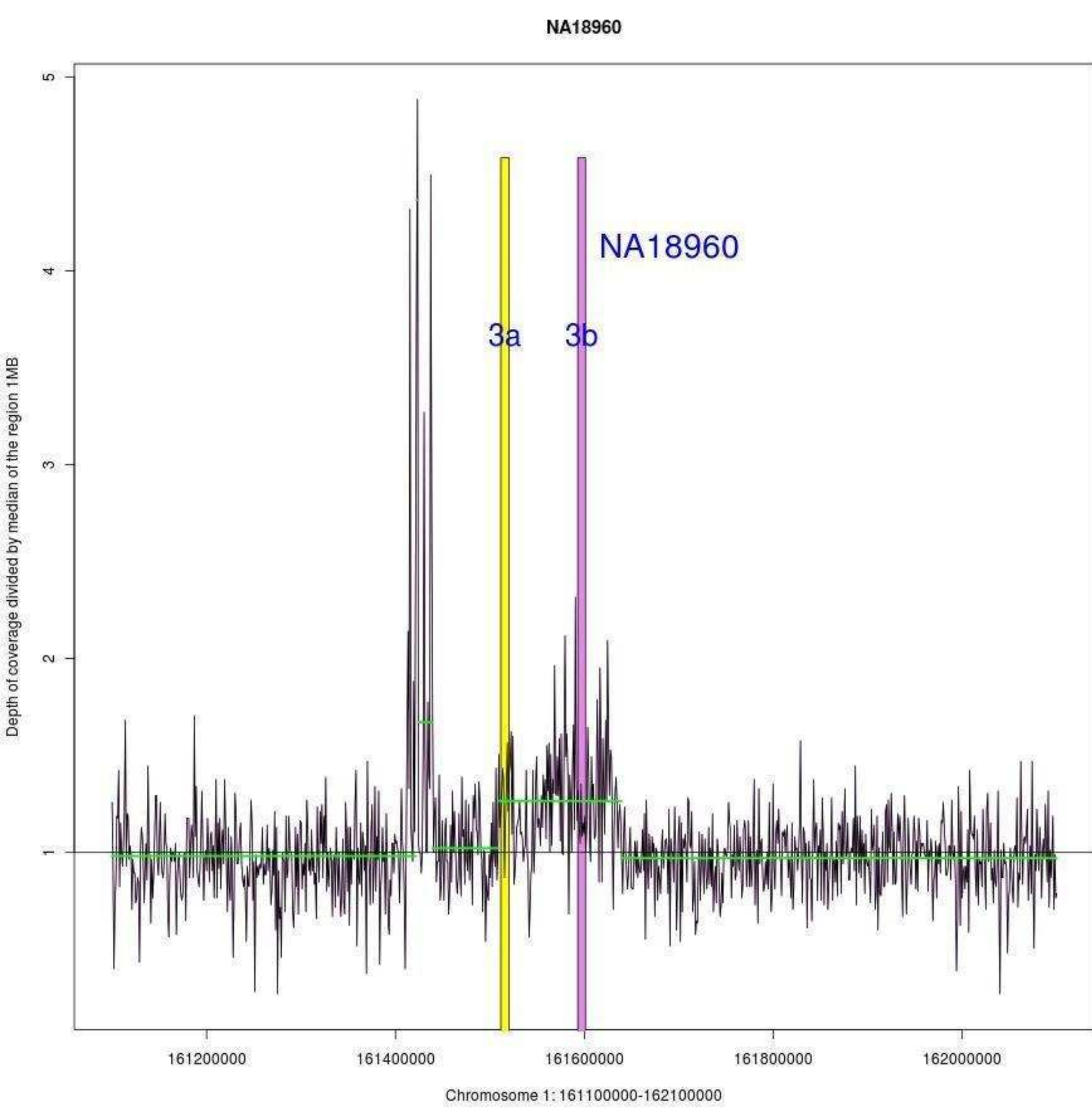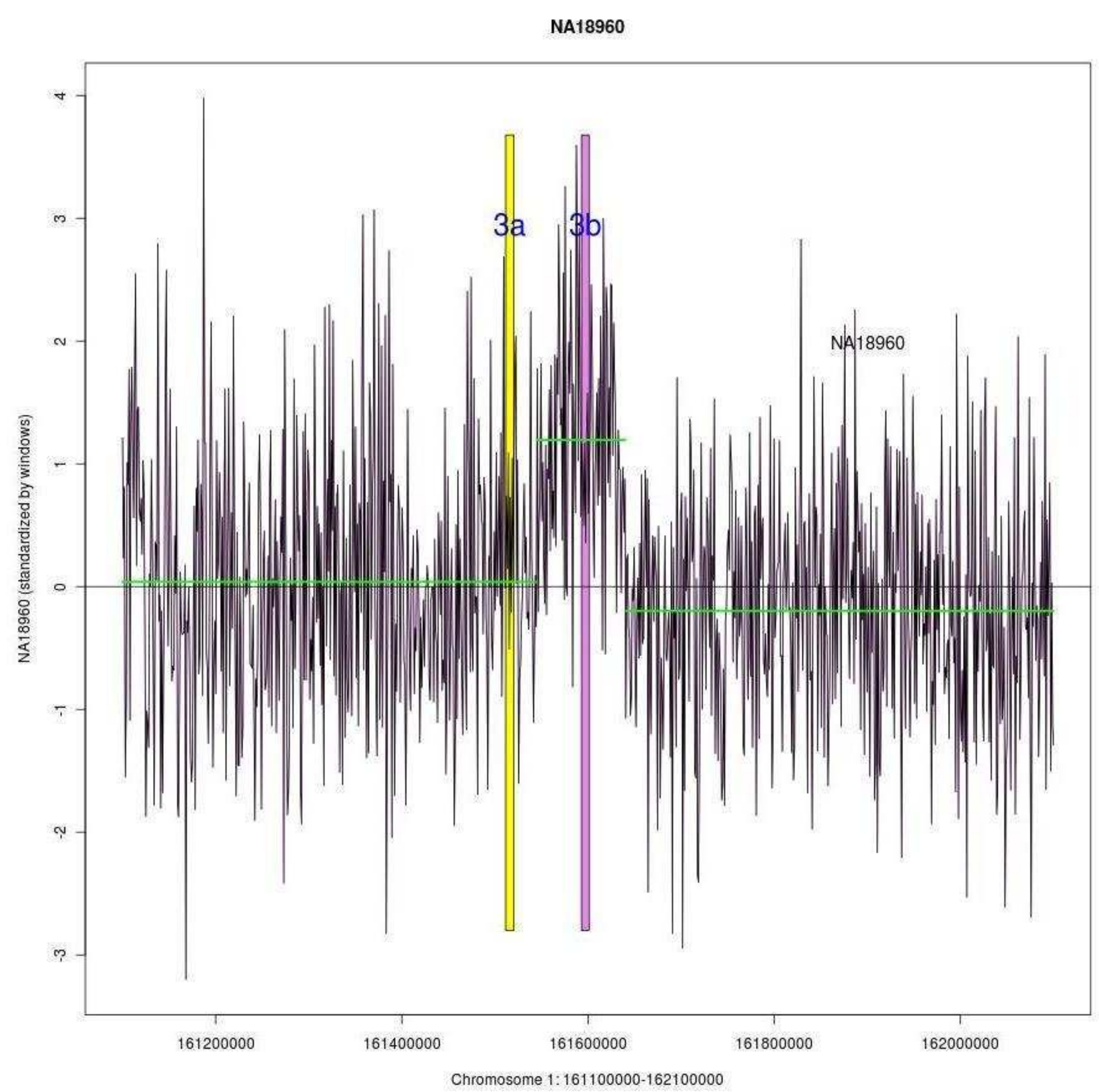

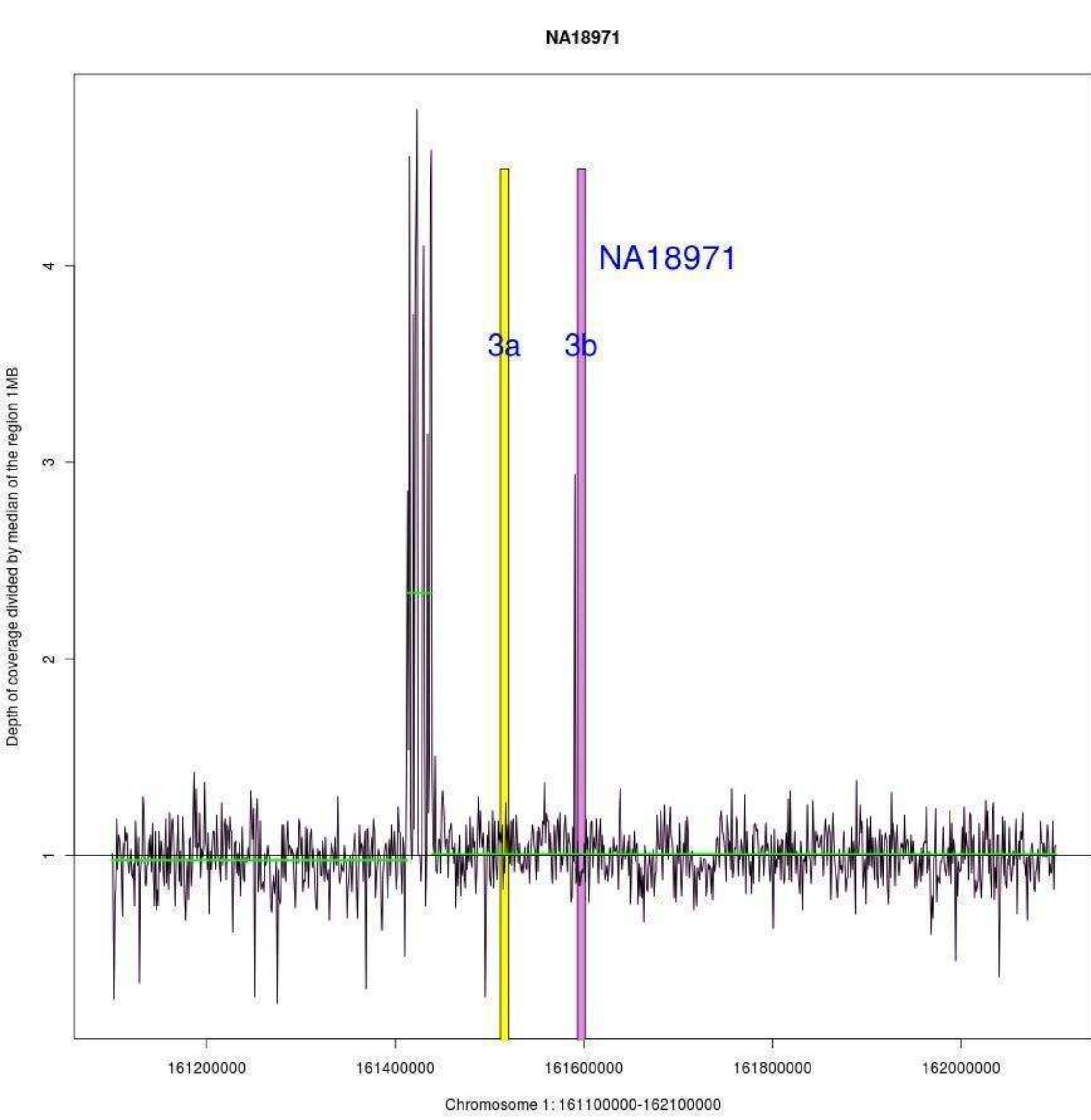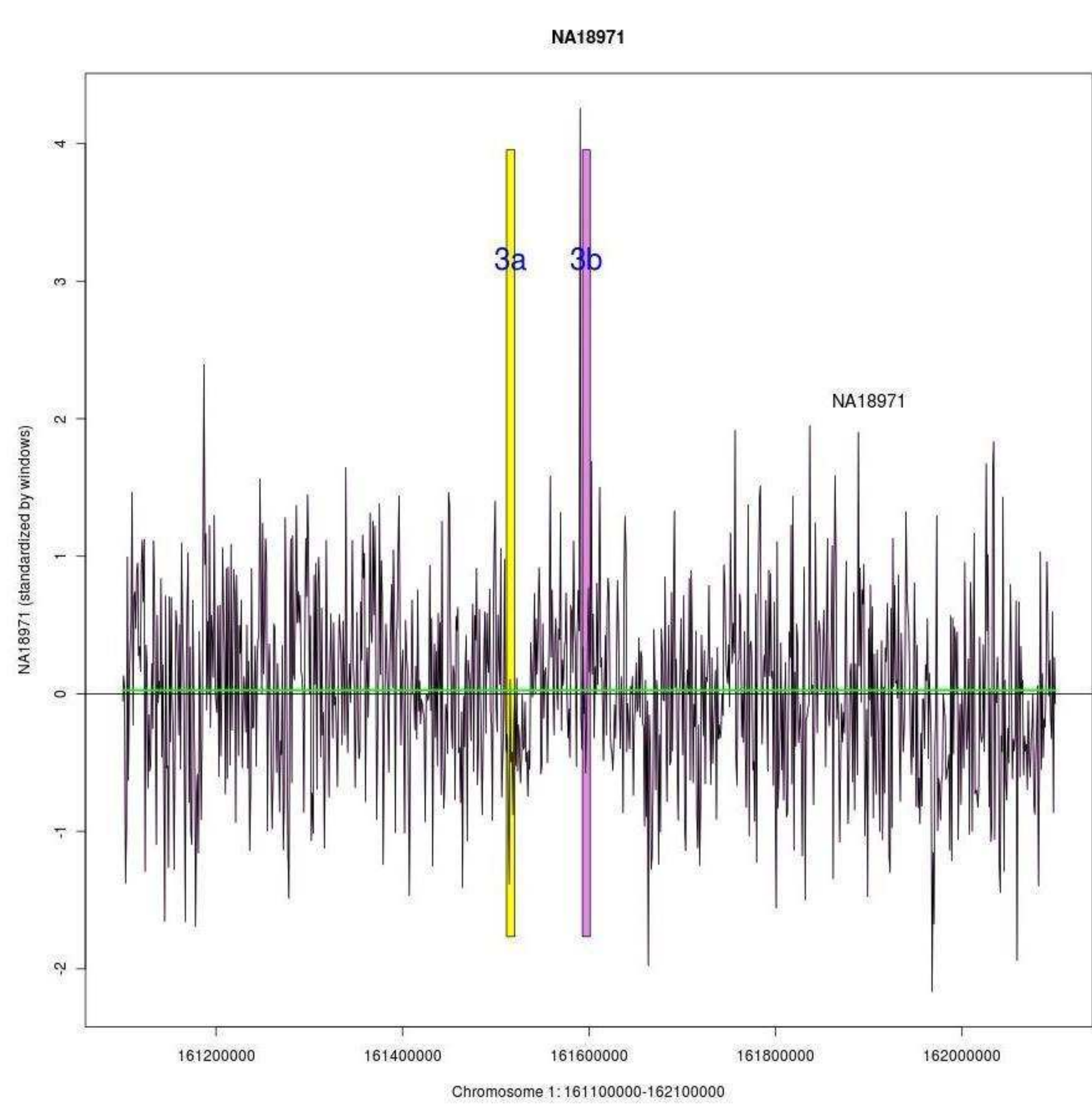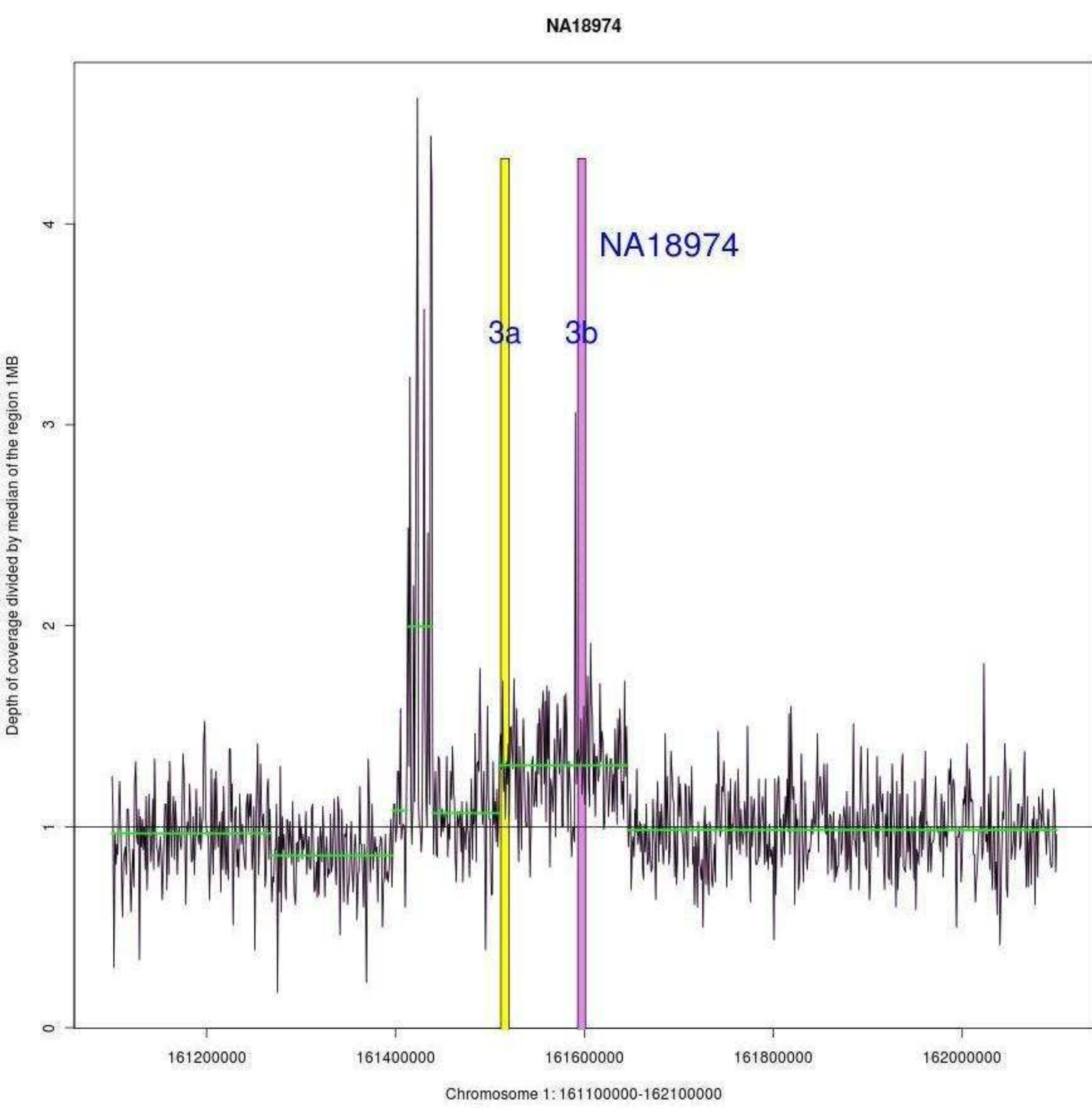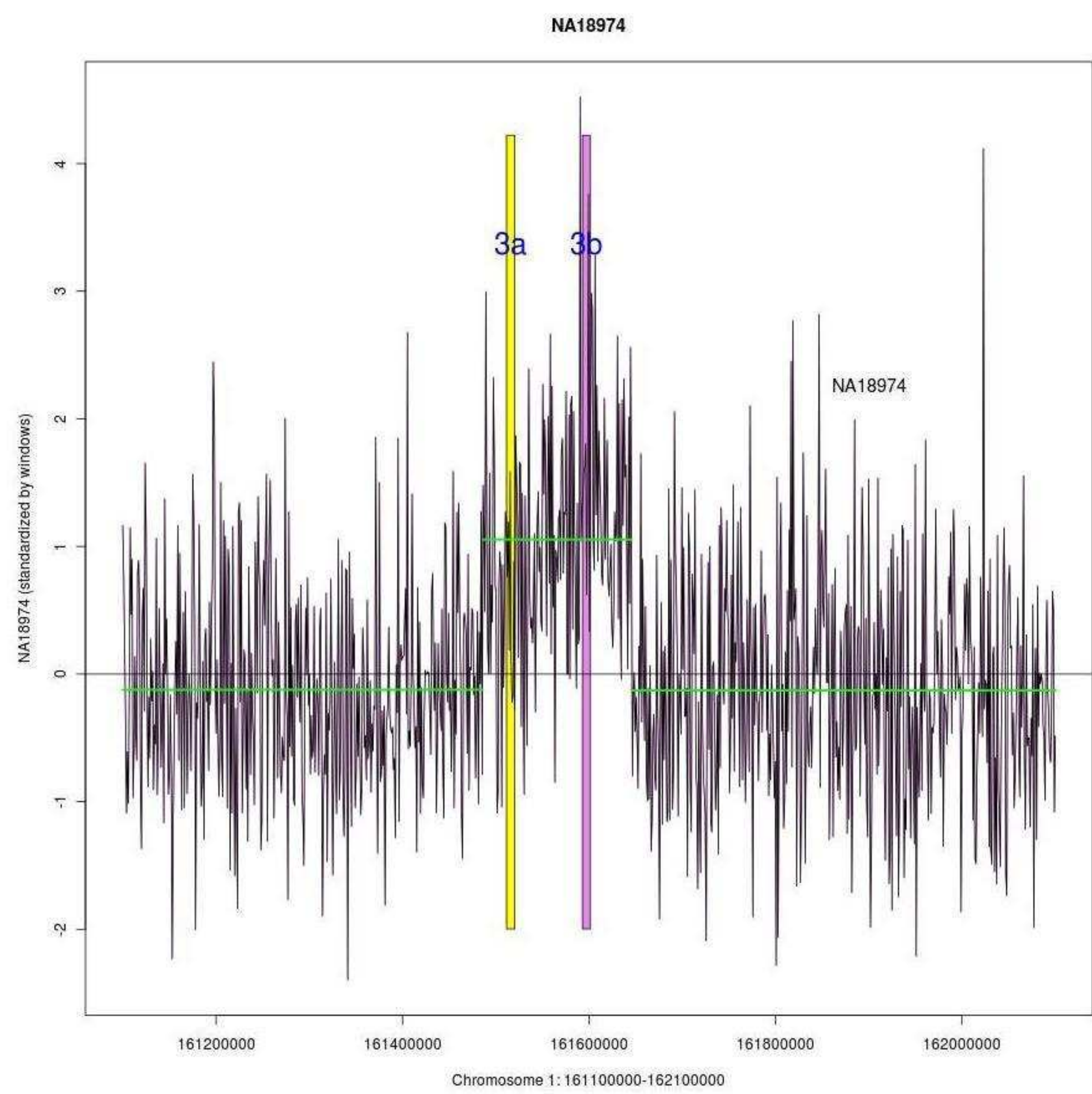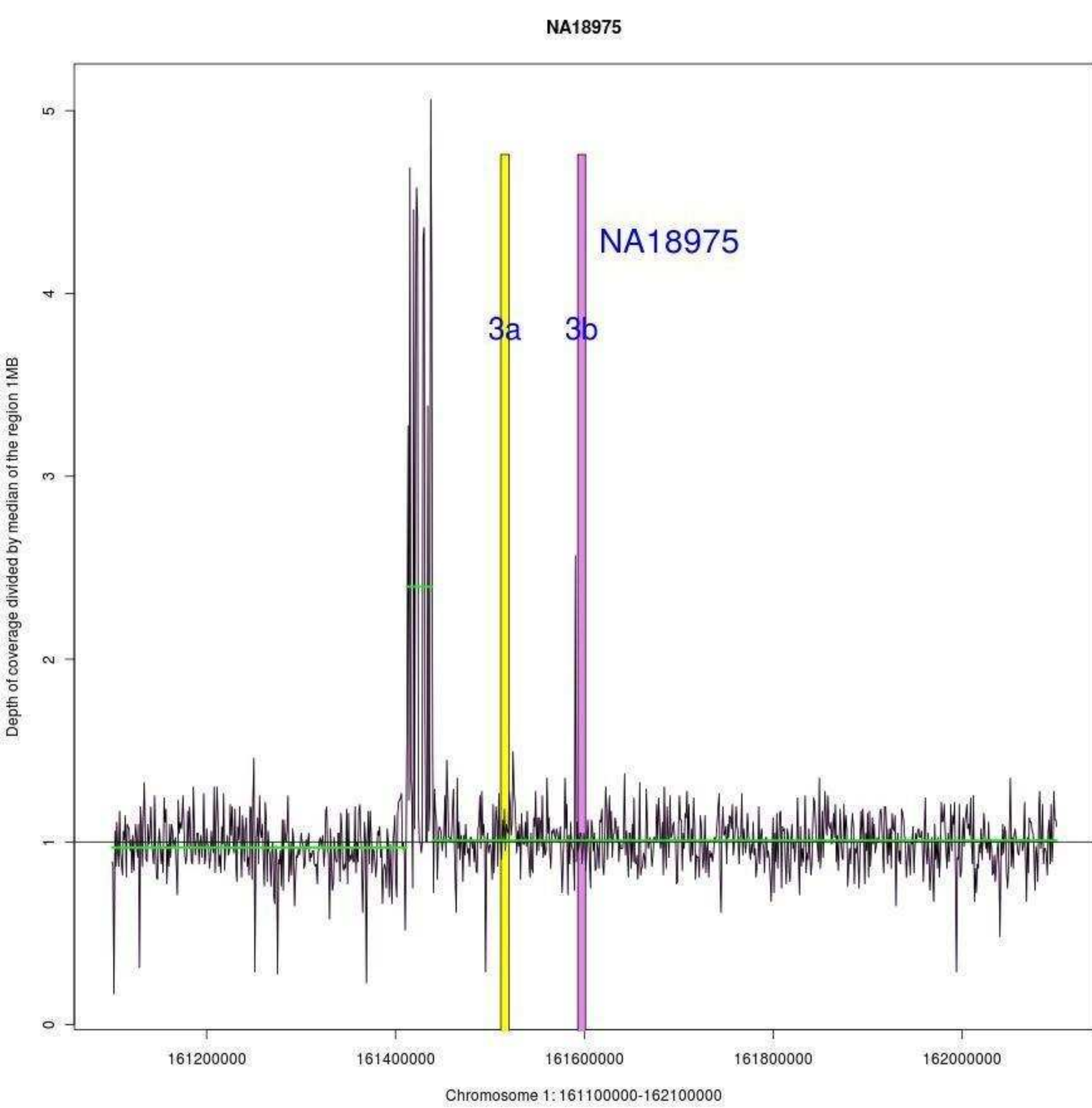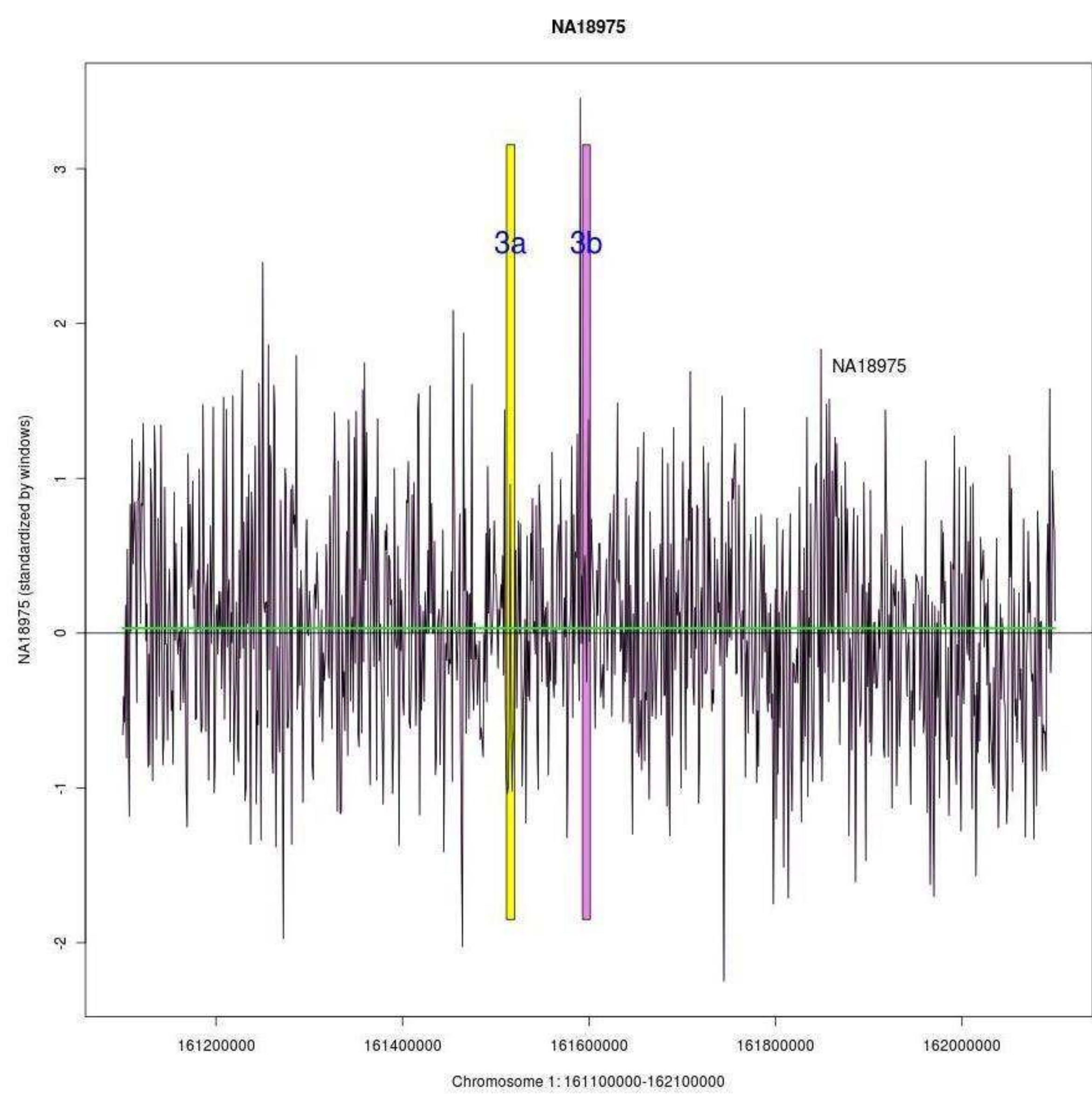

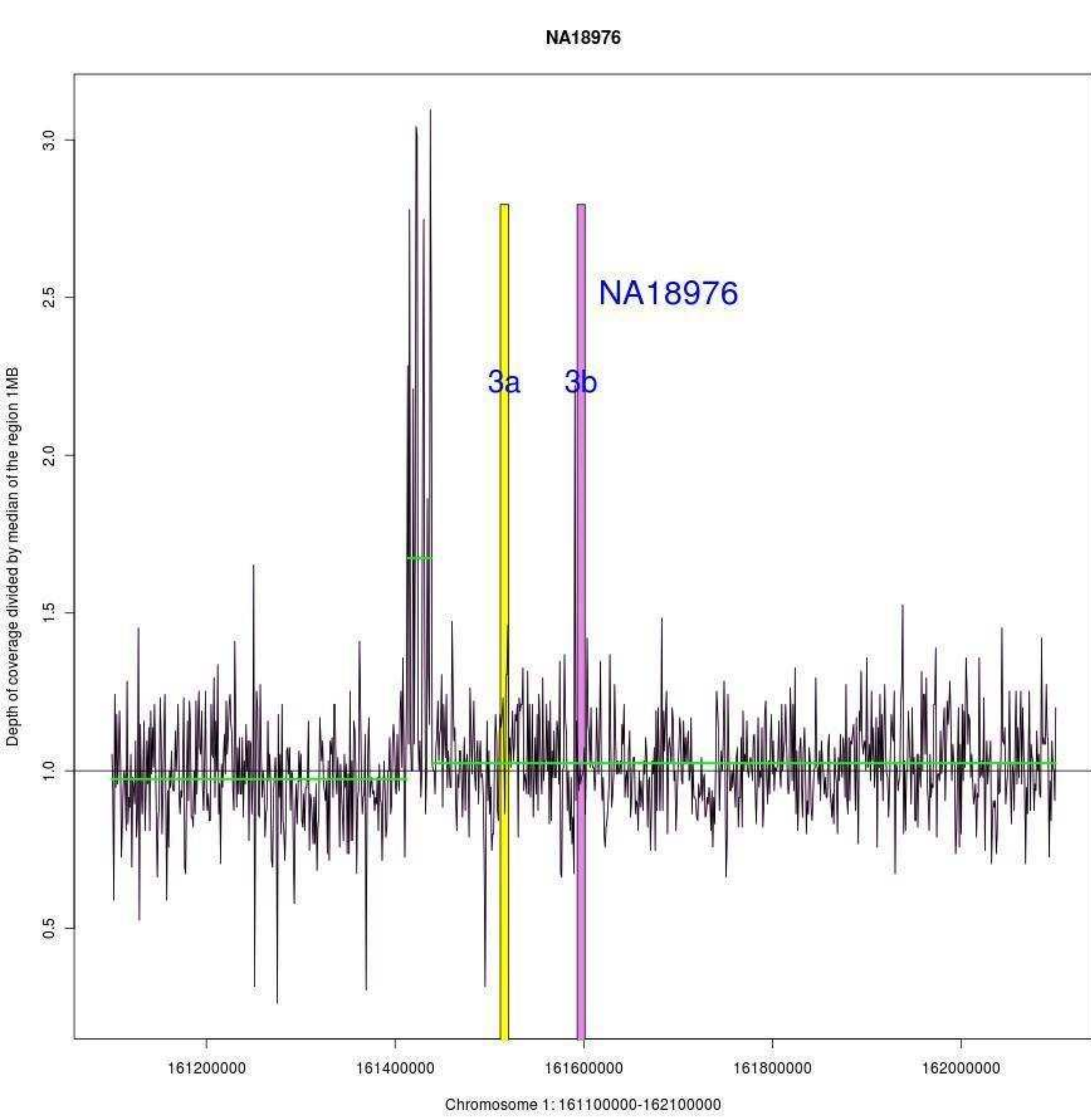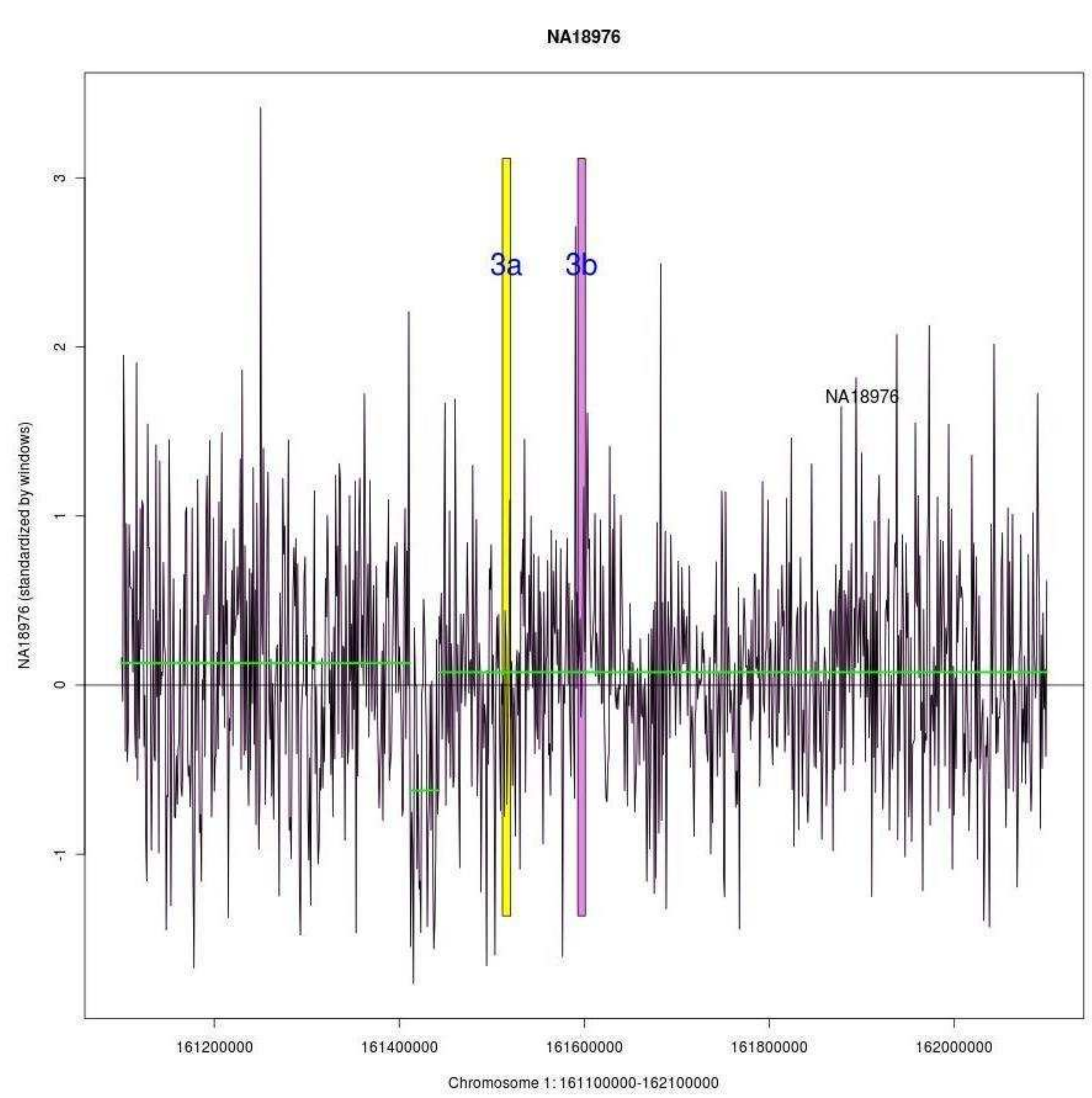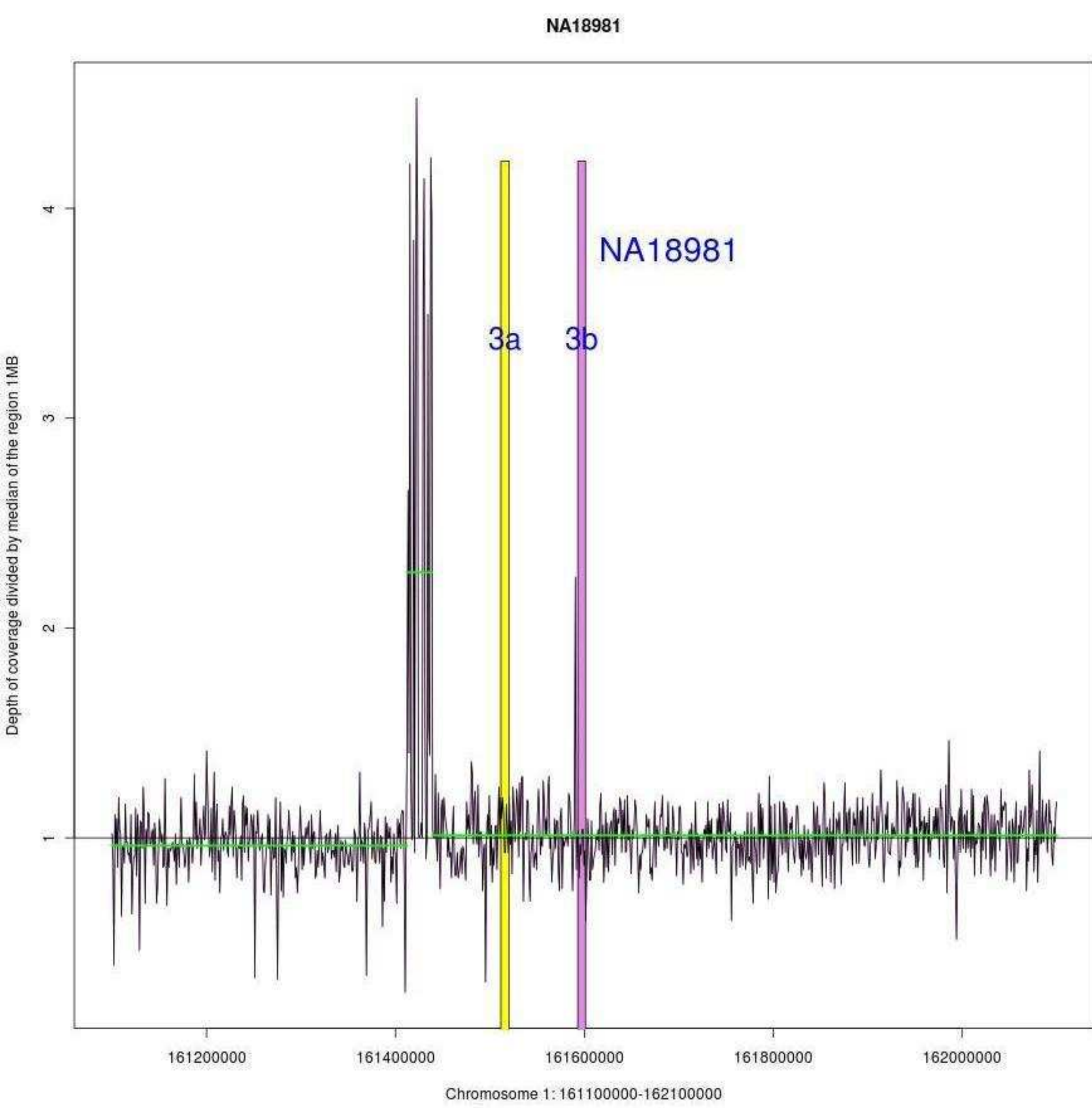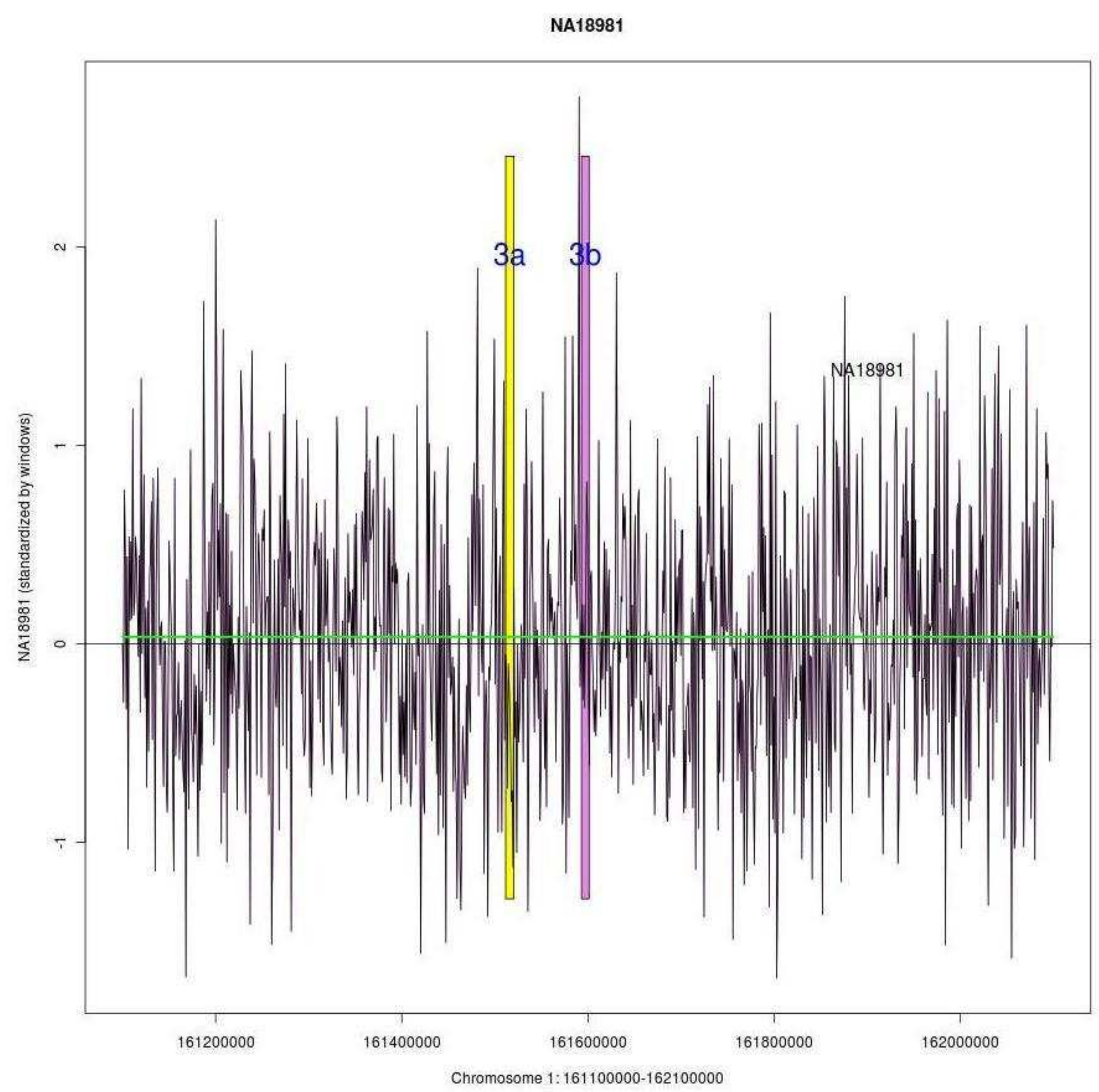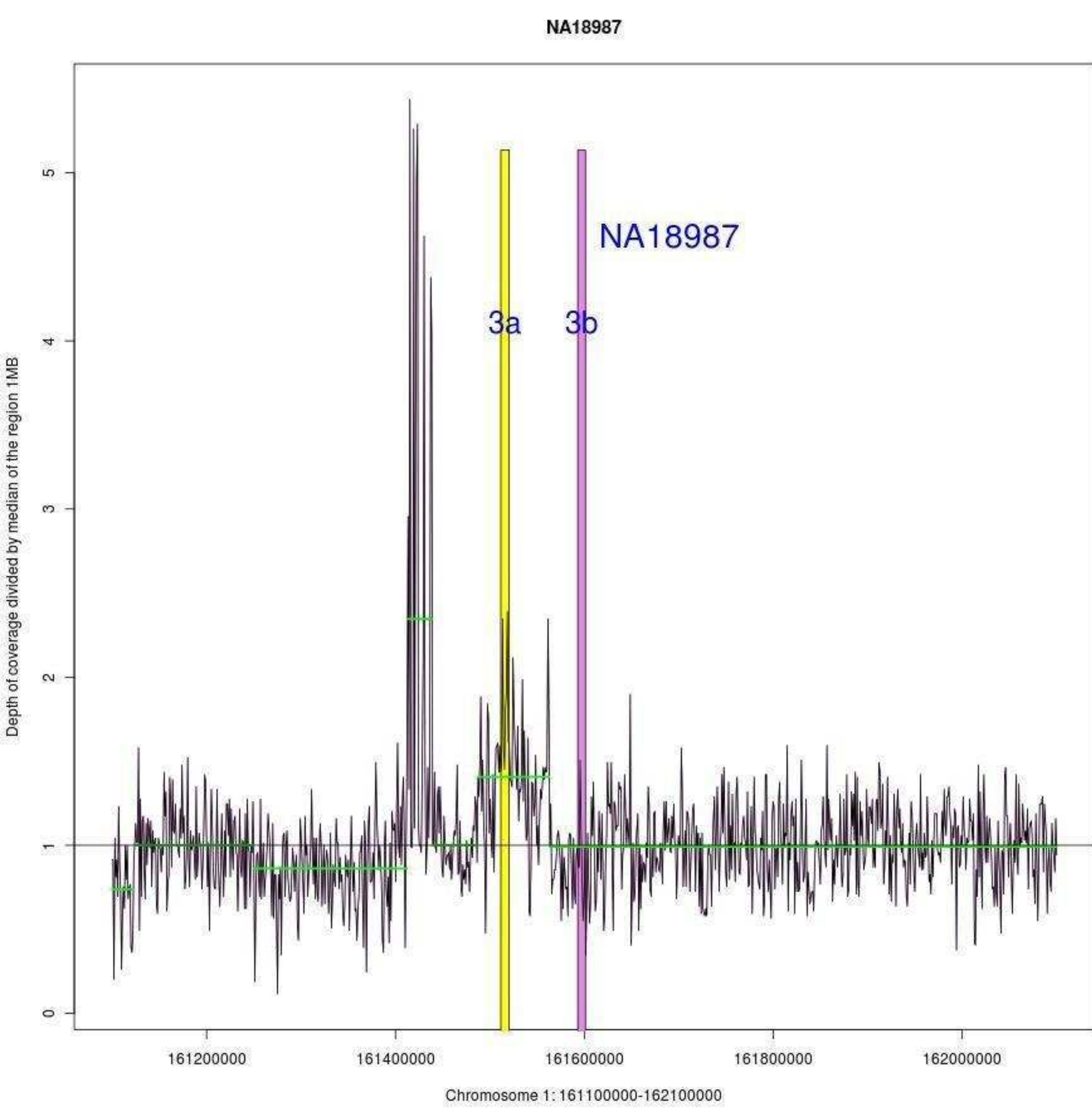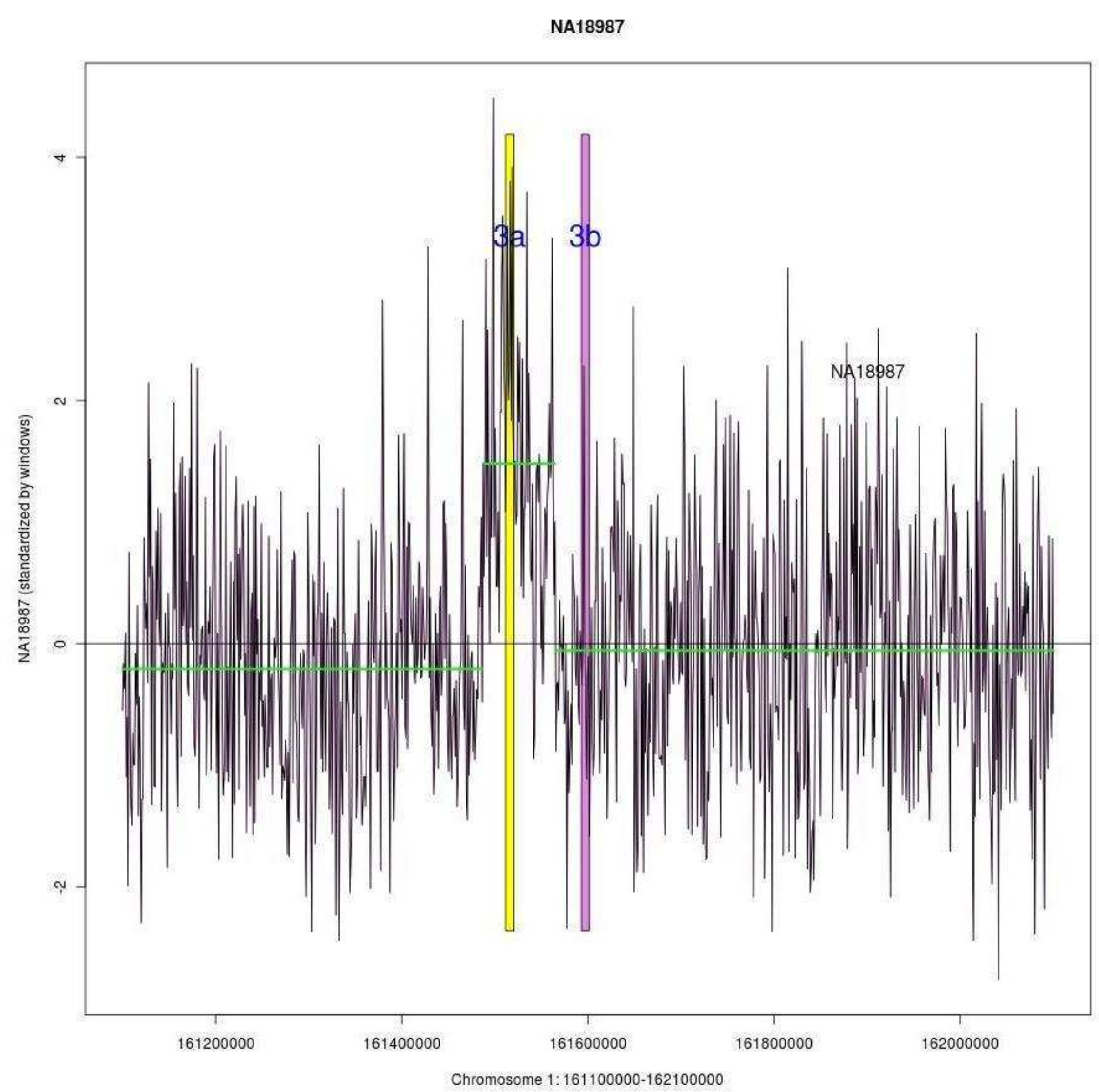

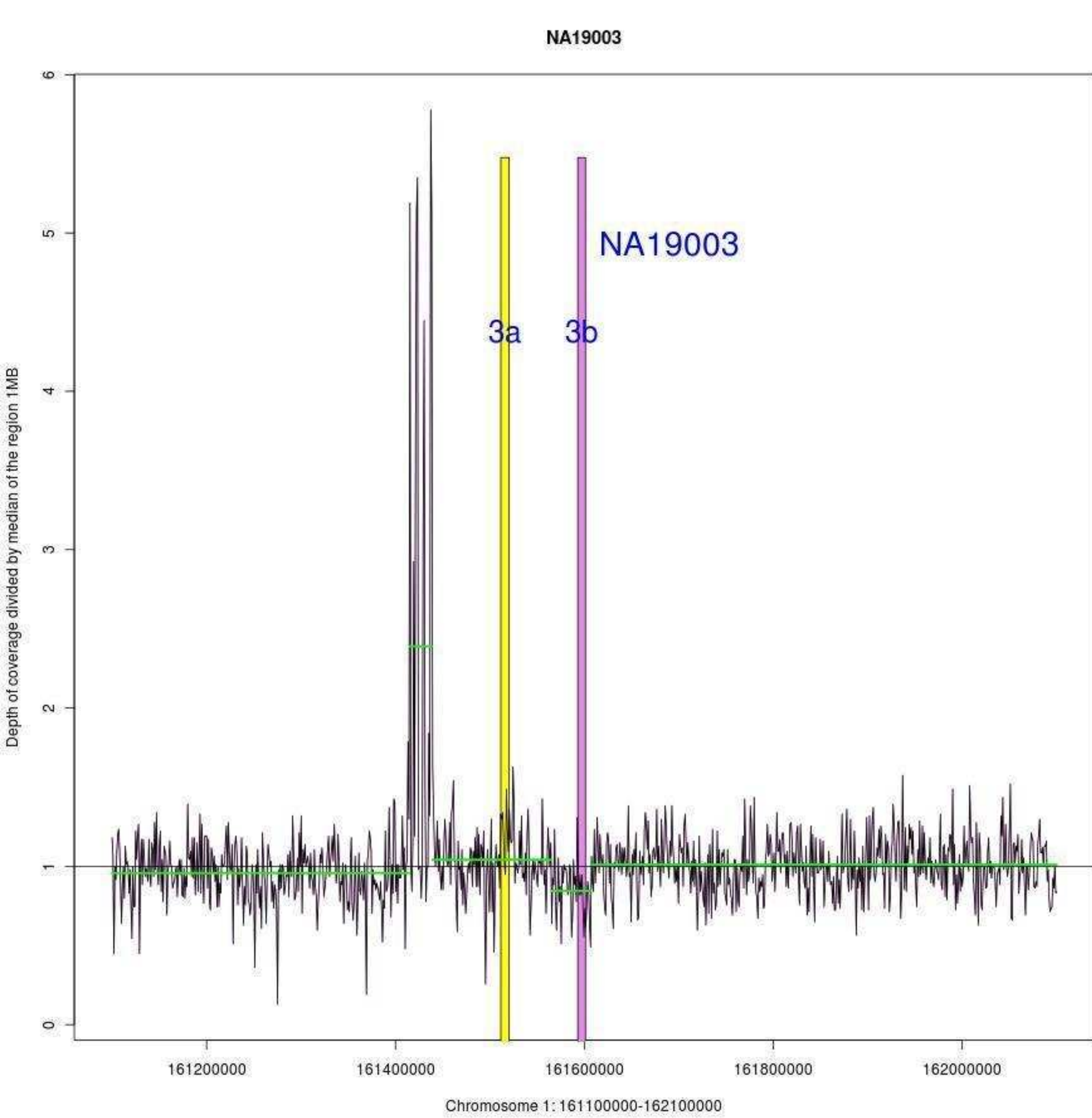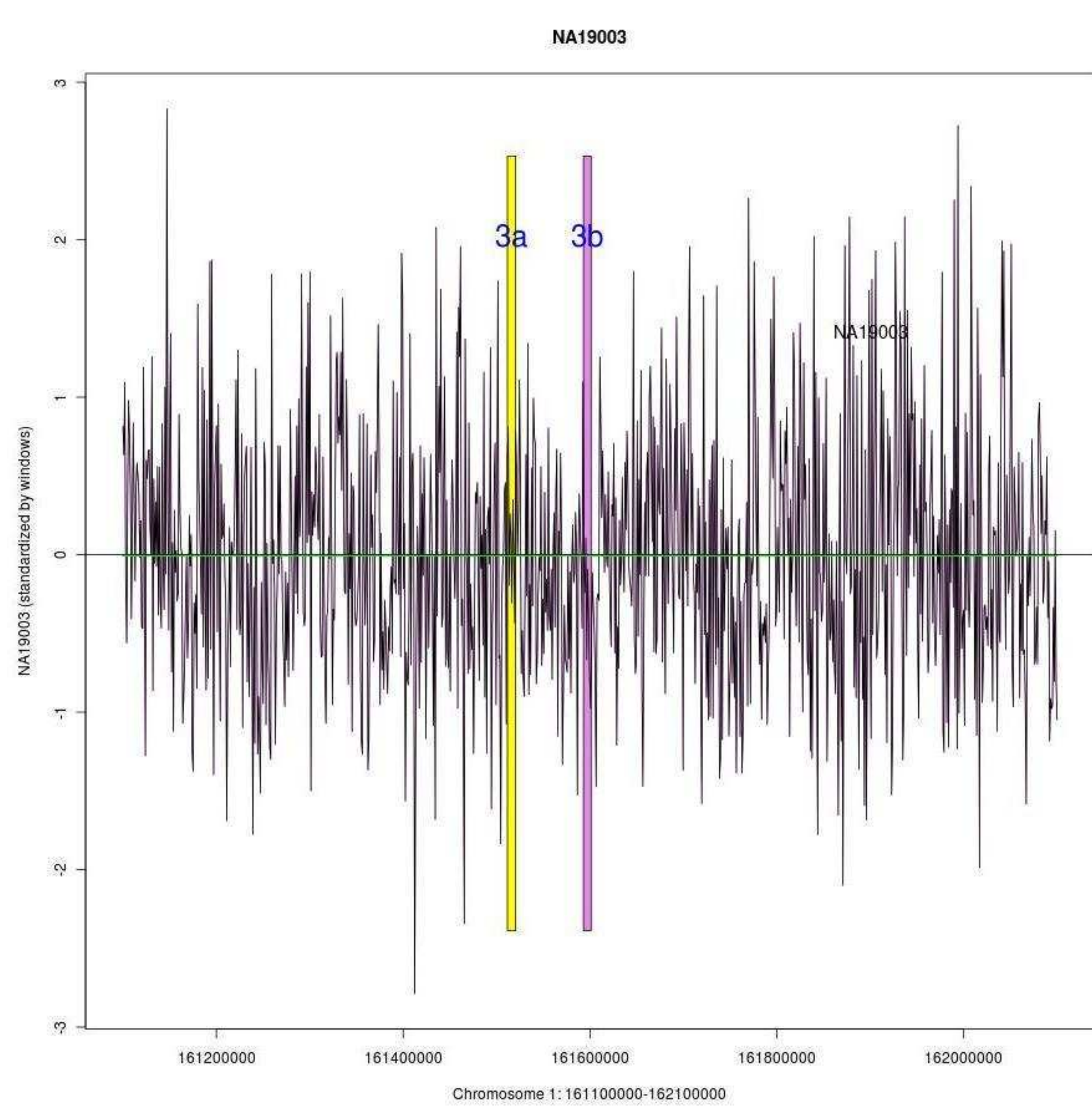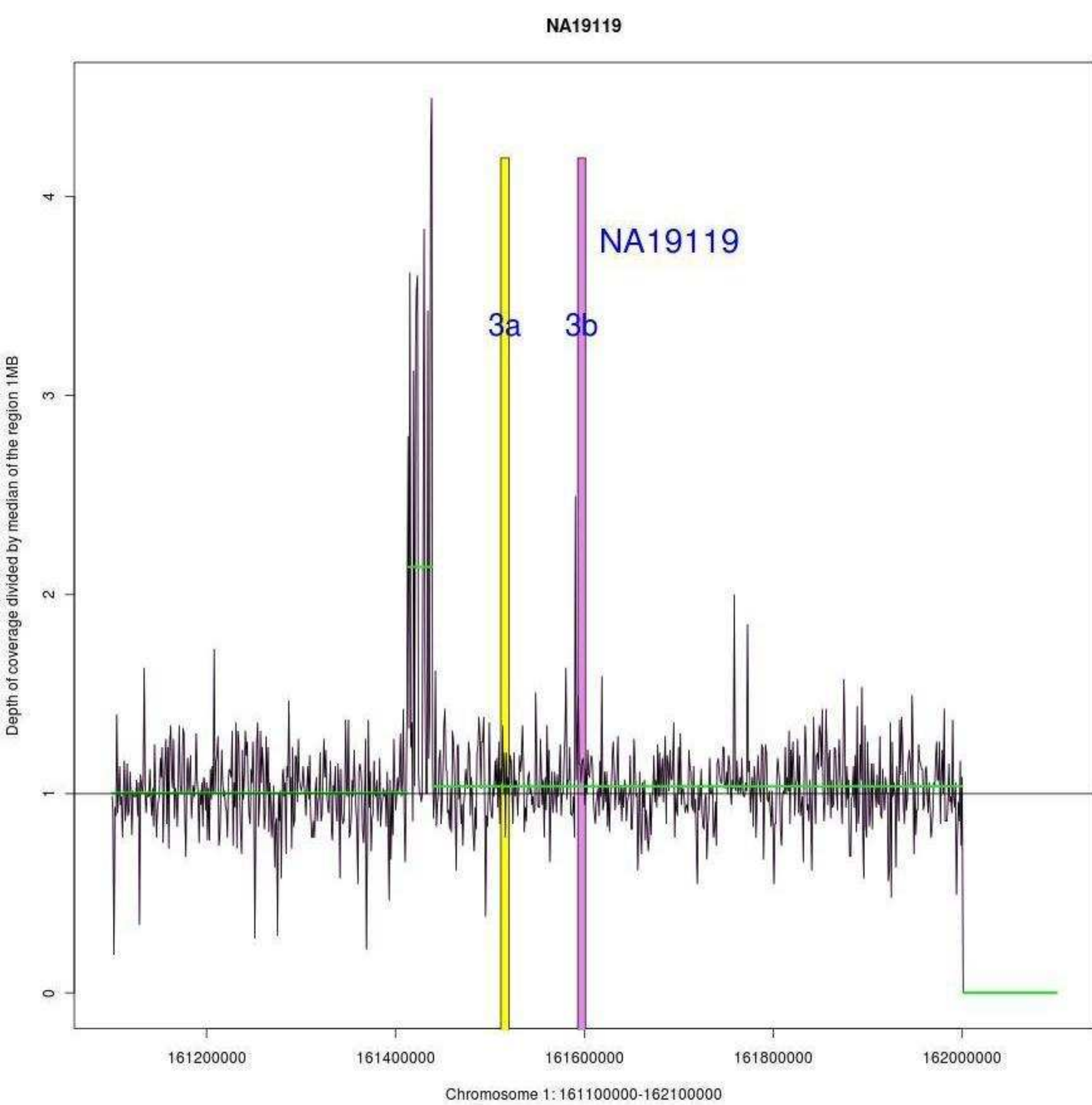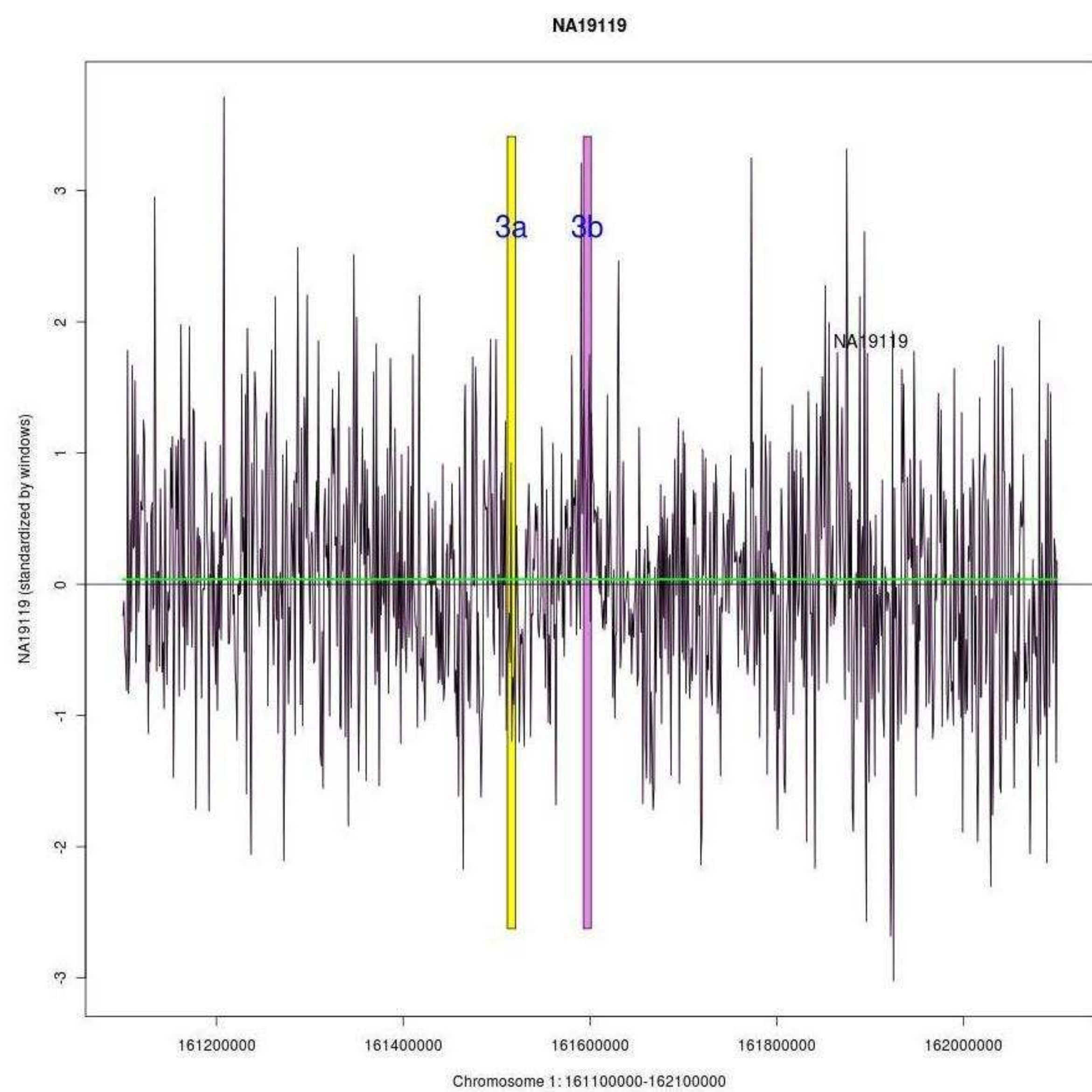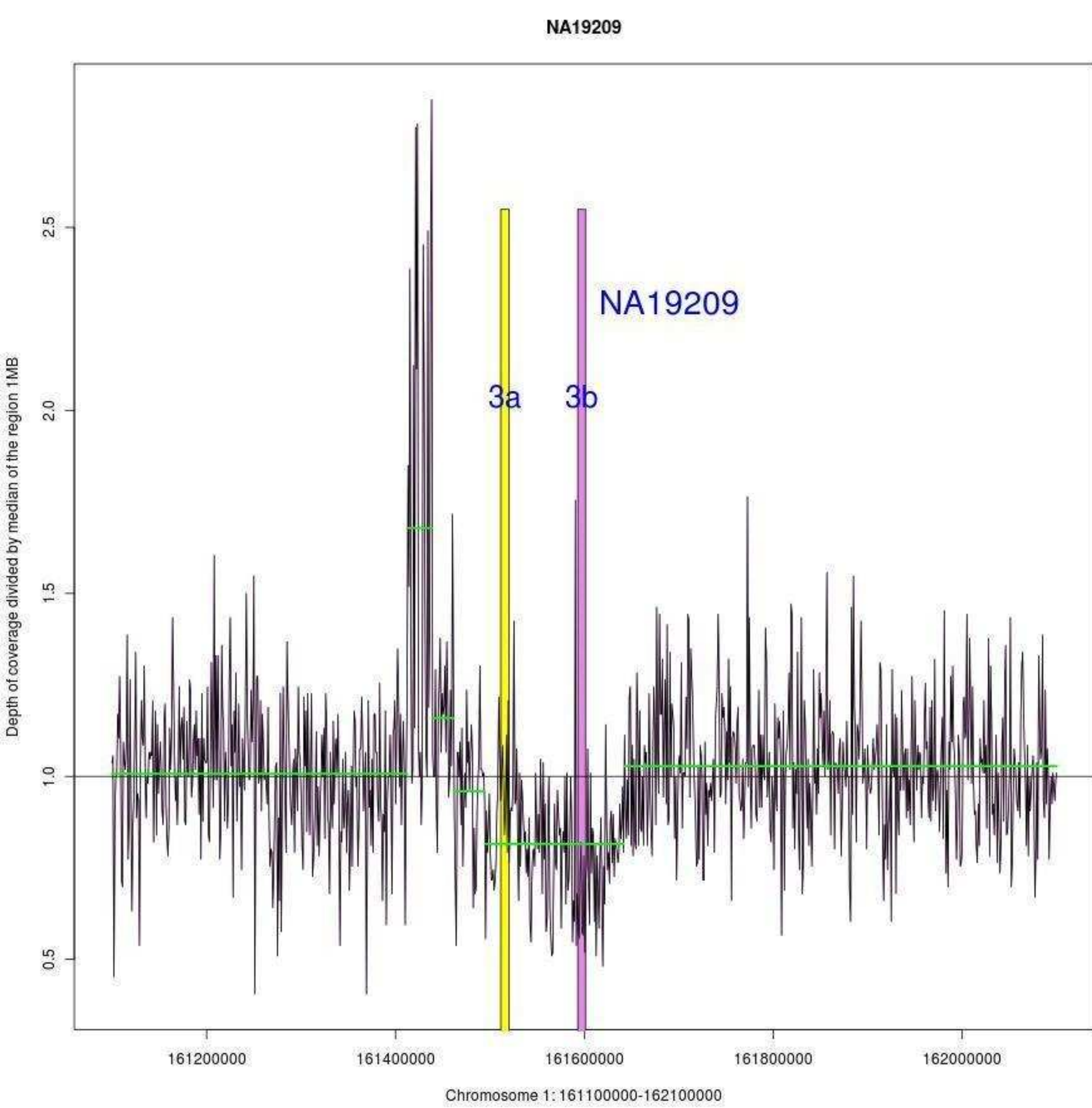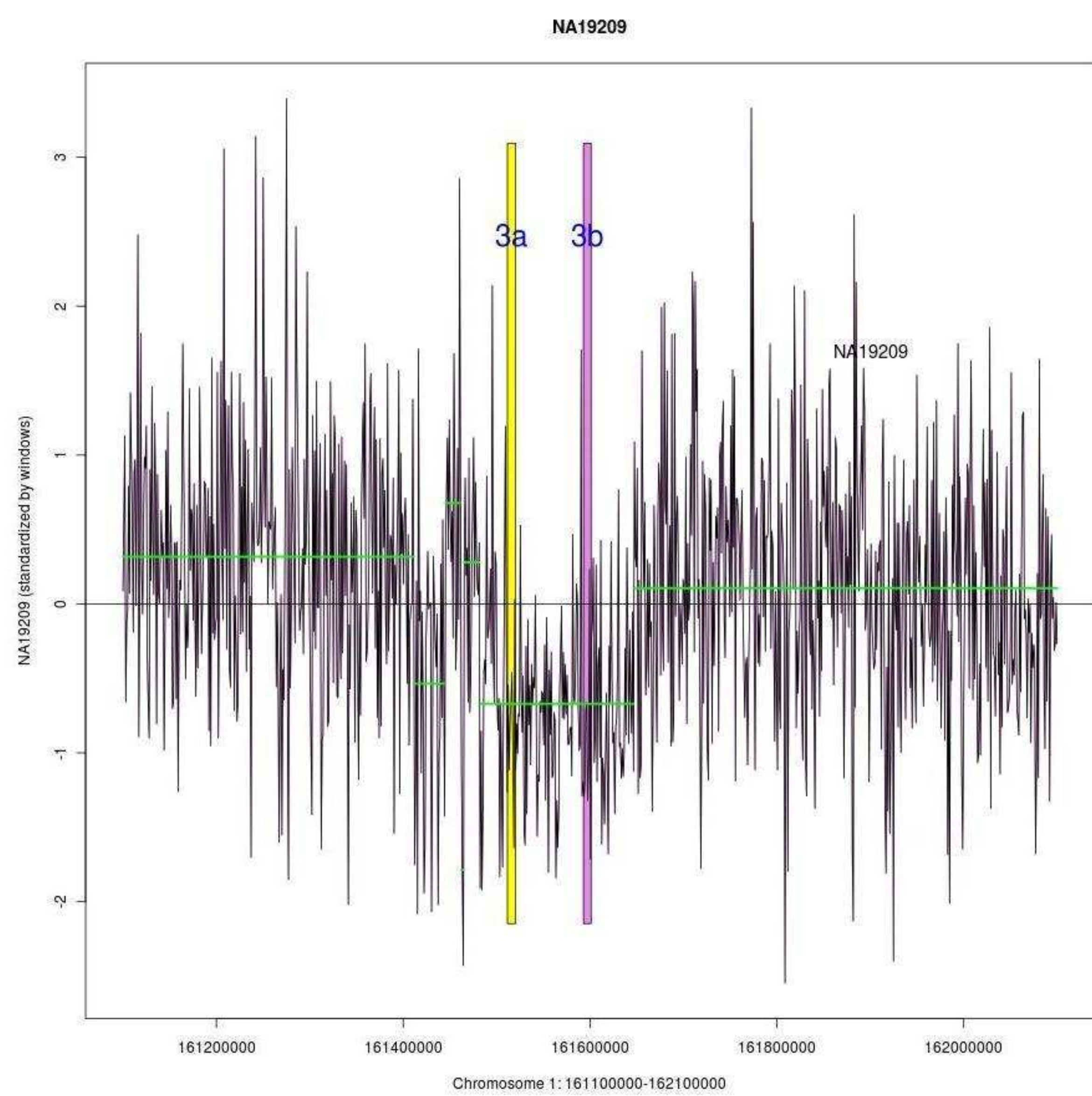

Supplement: Figure S4 — Traces of discordant samples between the three methods. On the left are the traces before standardizing across samples and on the right are the traces after standardizing across samples. 3a = position of FCGR3A and 3b = position of FCGR3B. The green horizontal lines are the mean values of regions from the step segmentation of the DNAcopy package. The file is Supplemental Figure 4.pdf. (PDF) [file pone.0063219.s004.pdf]

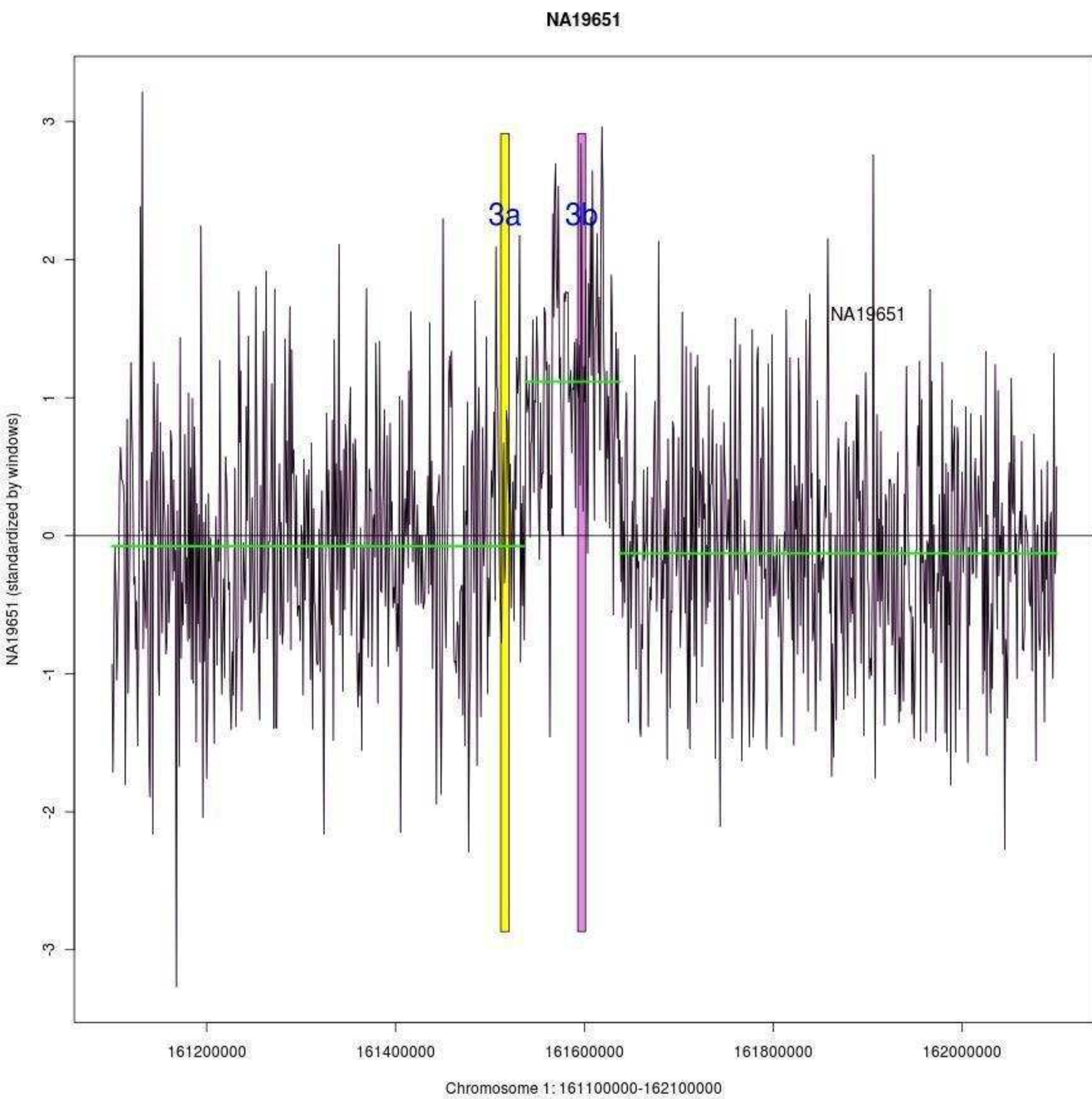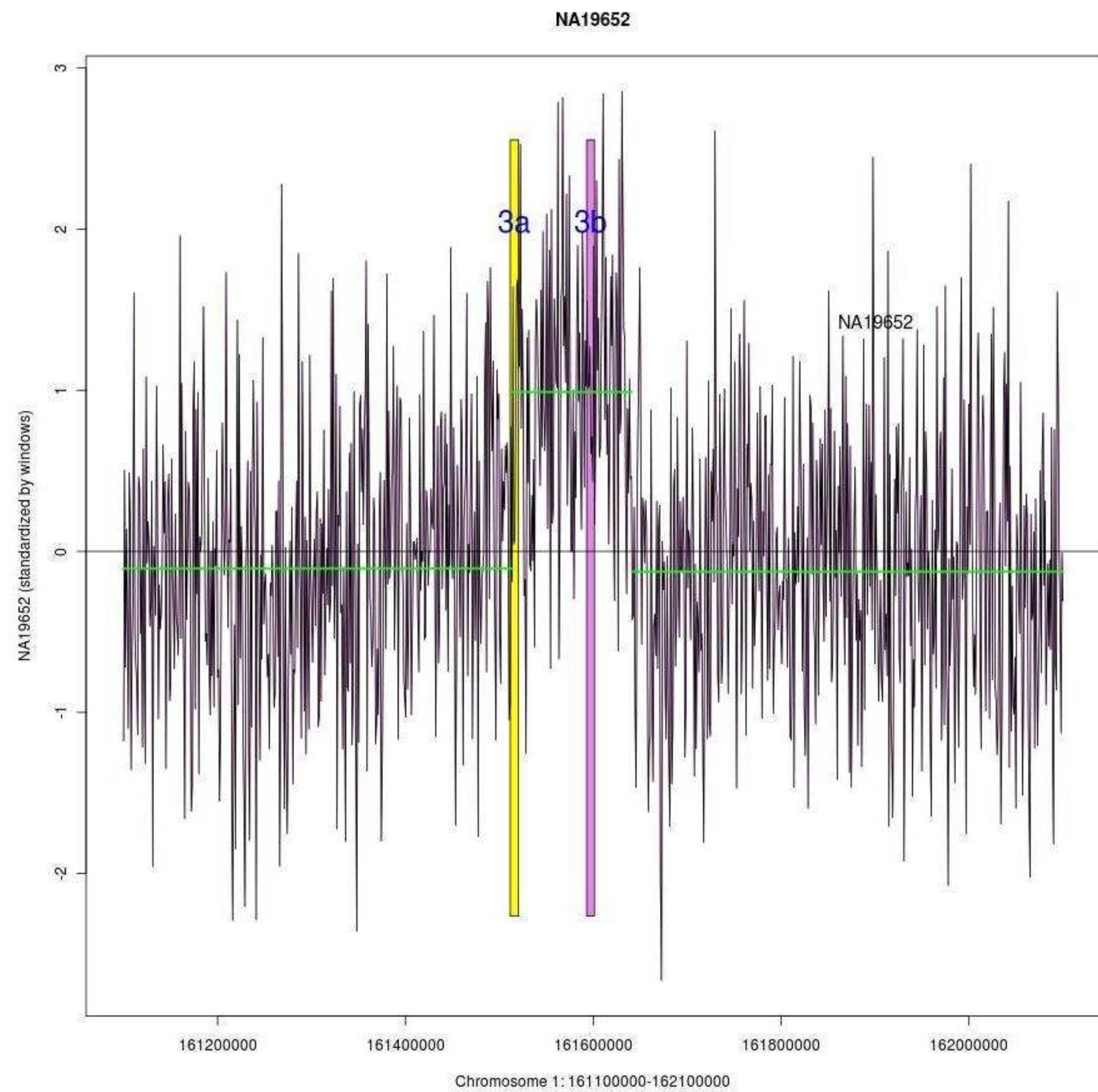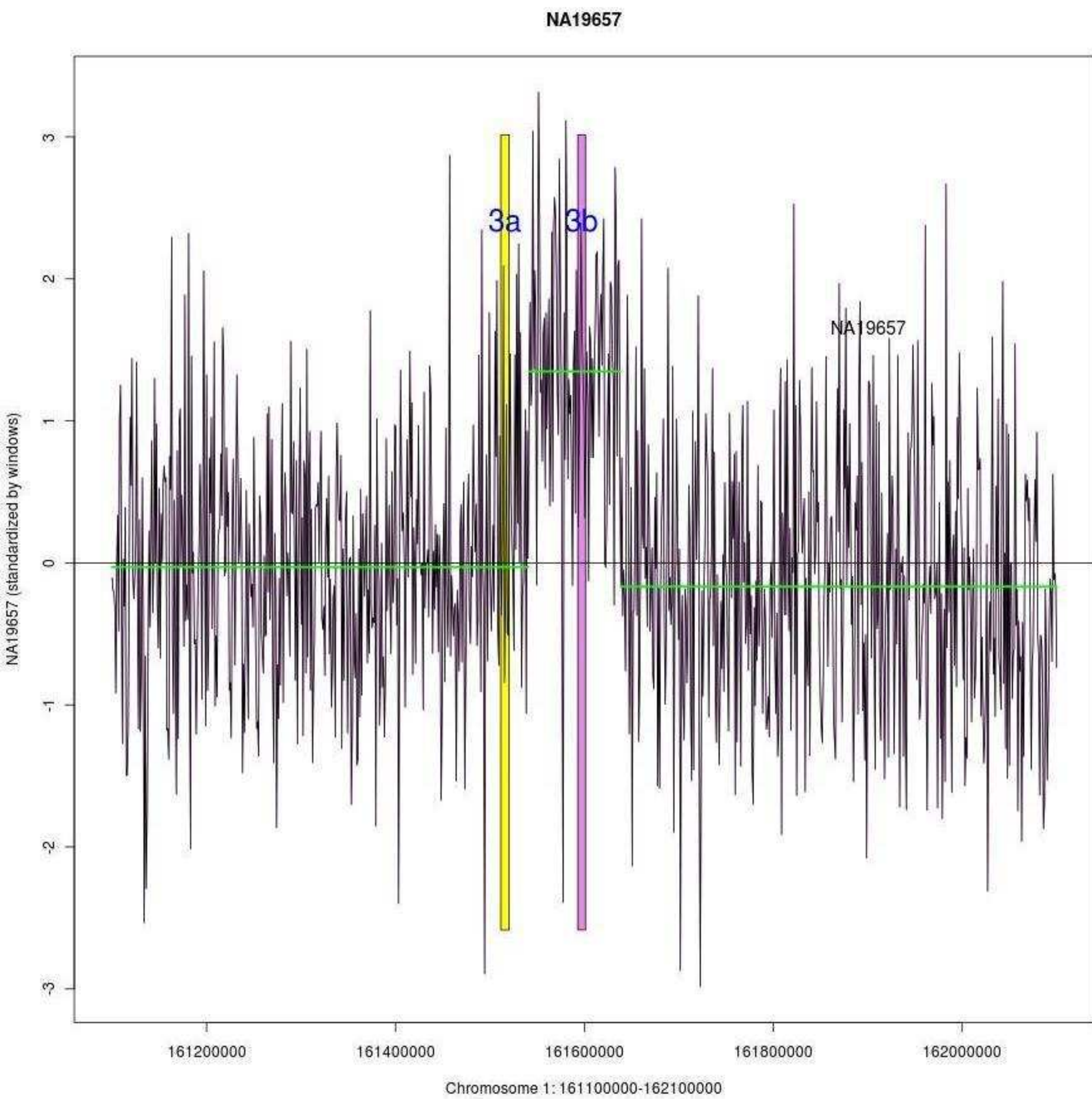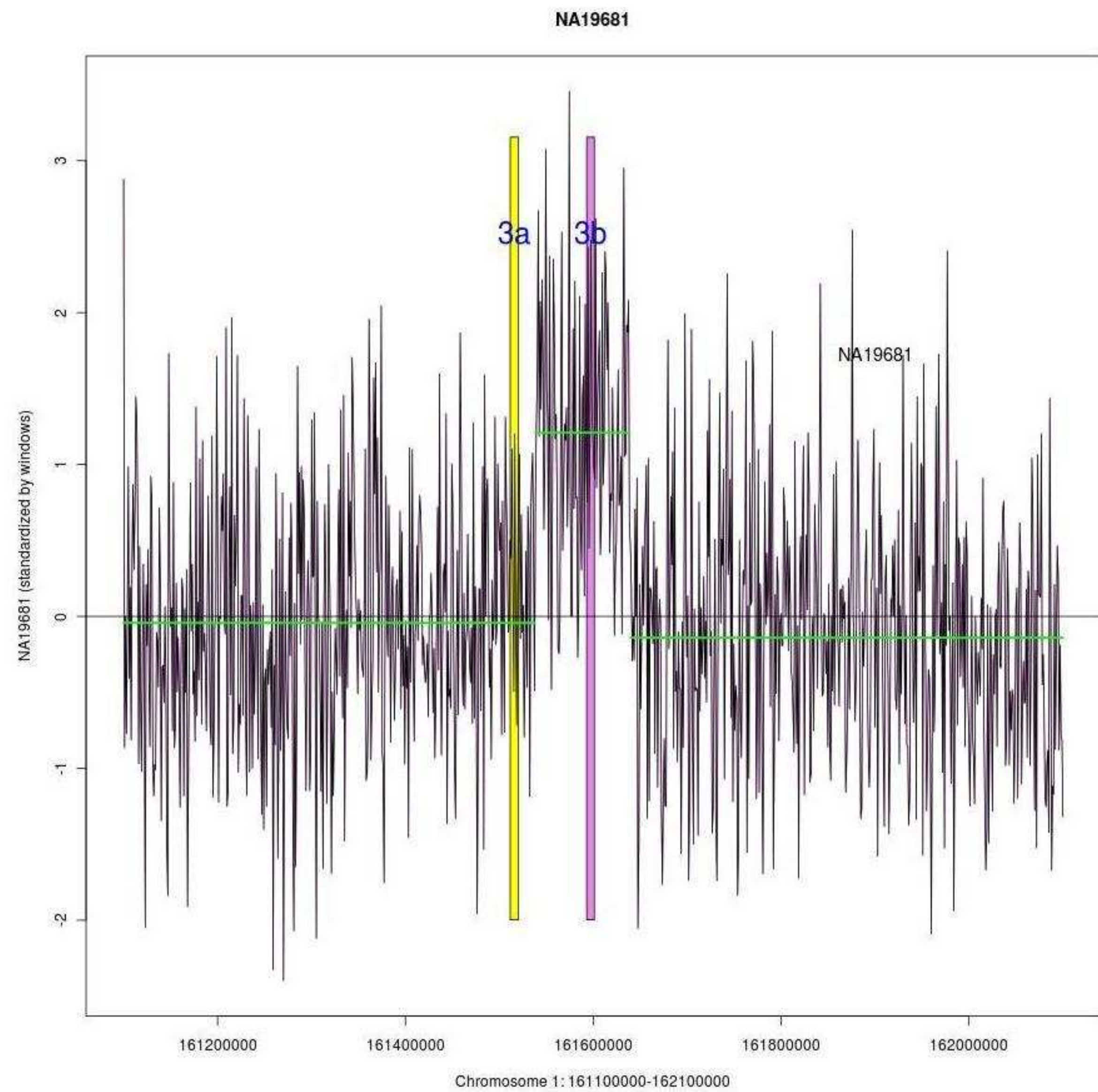

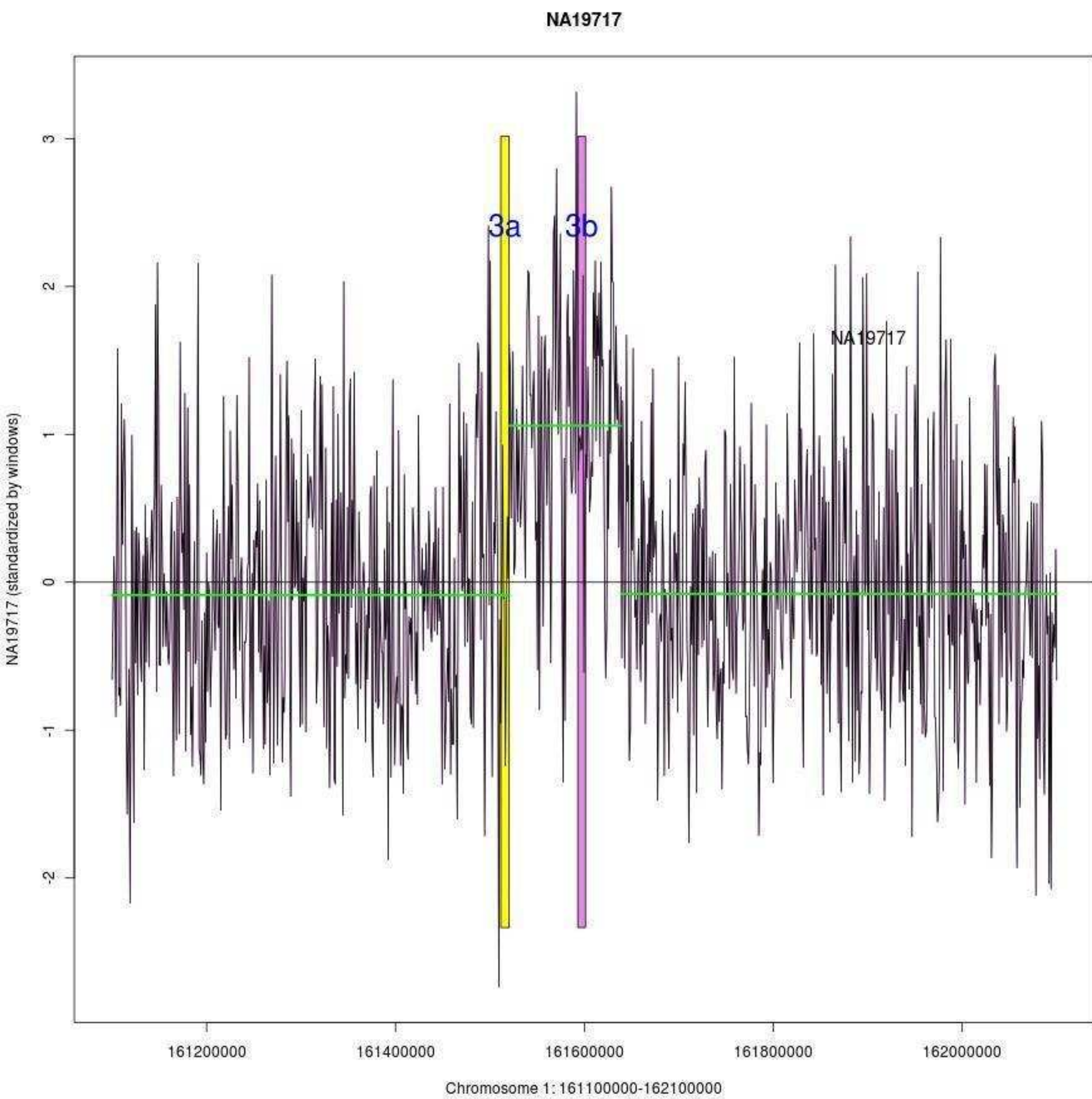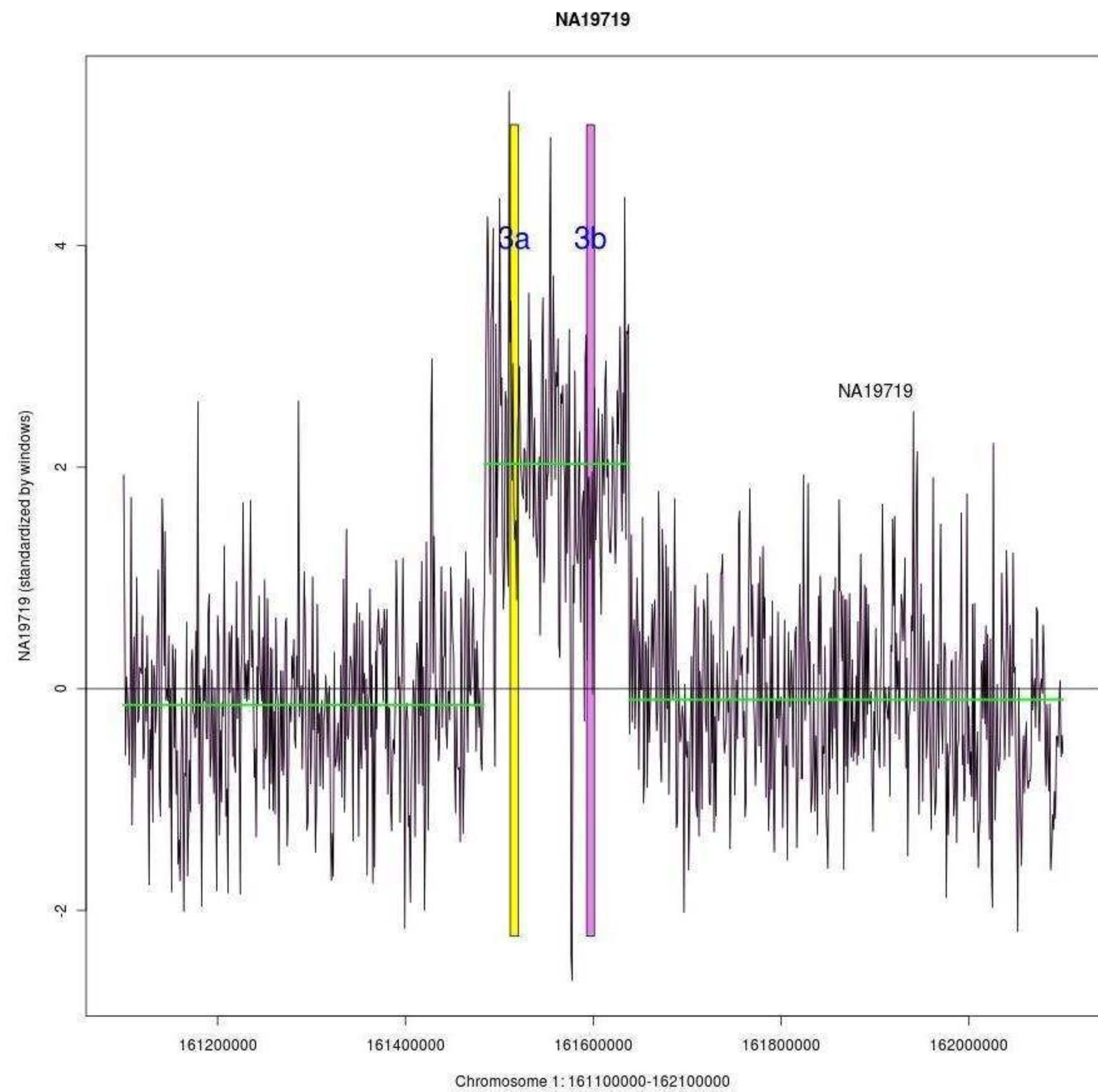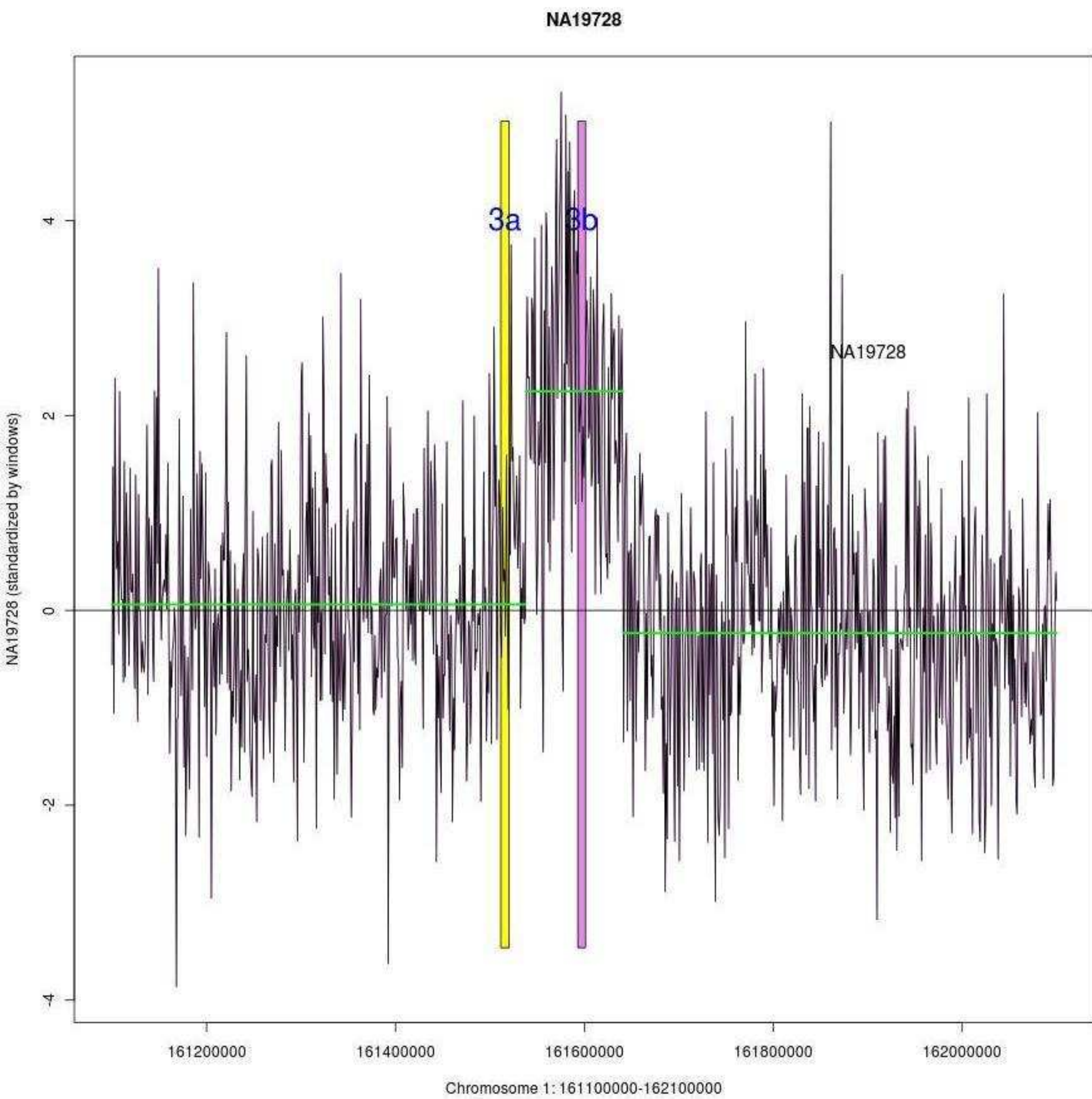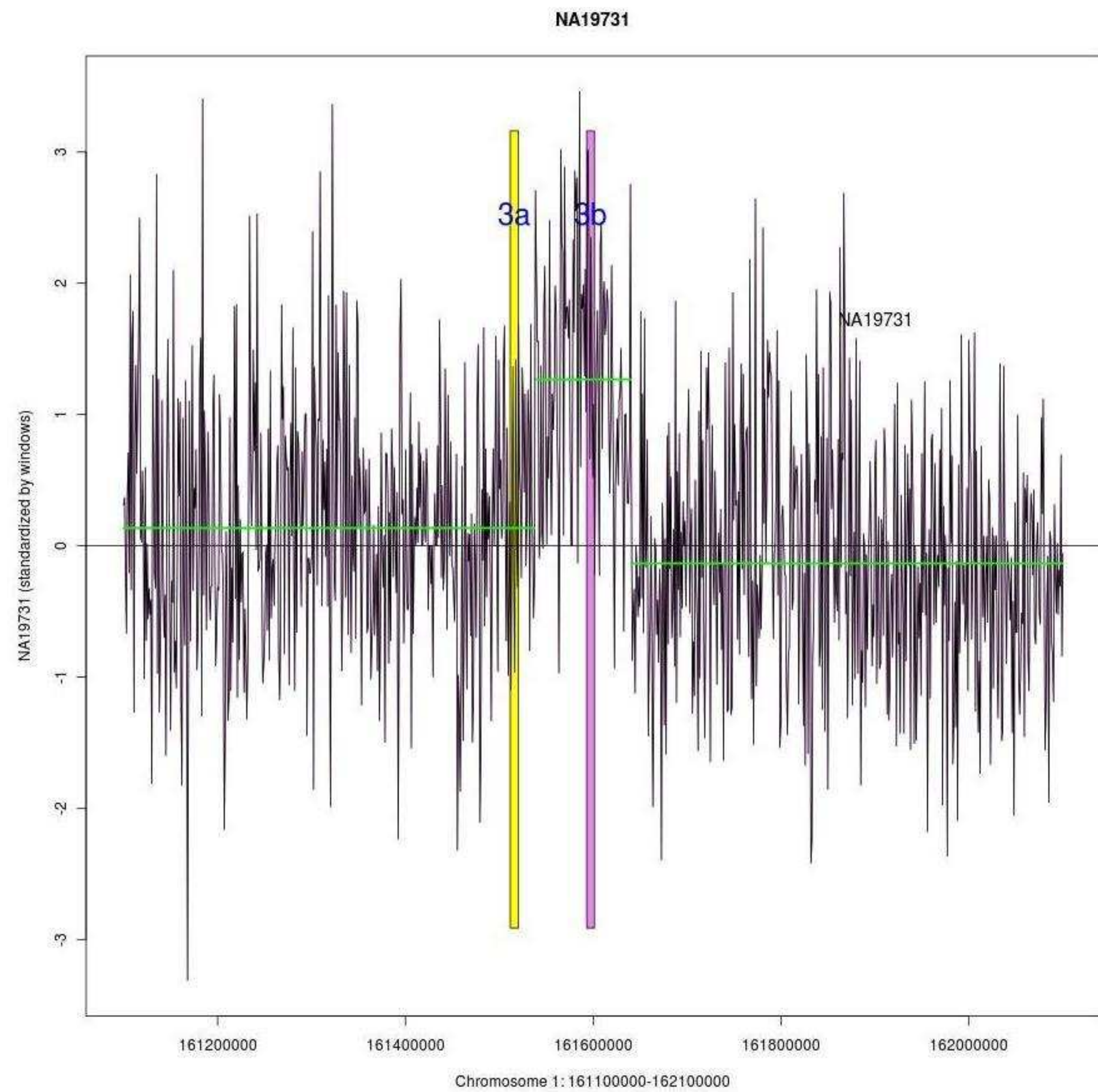

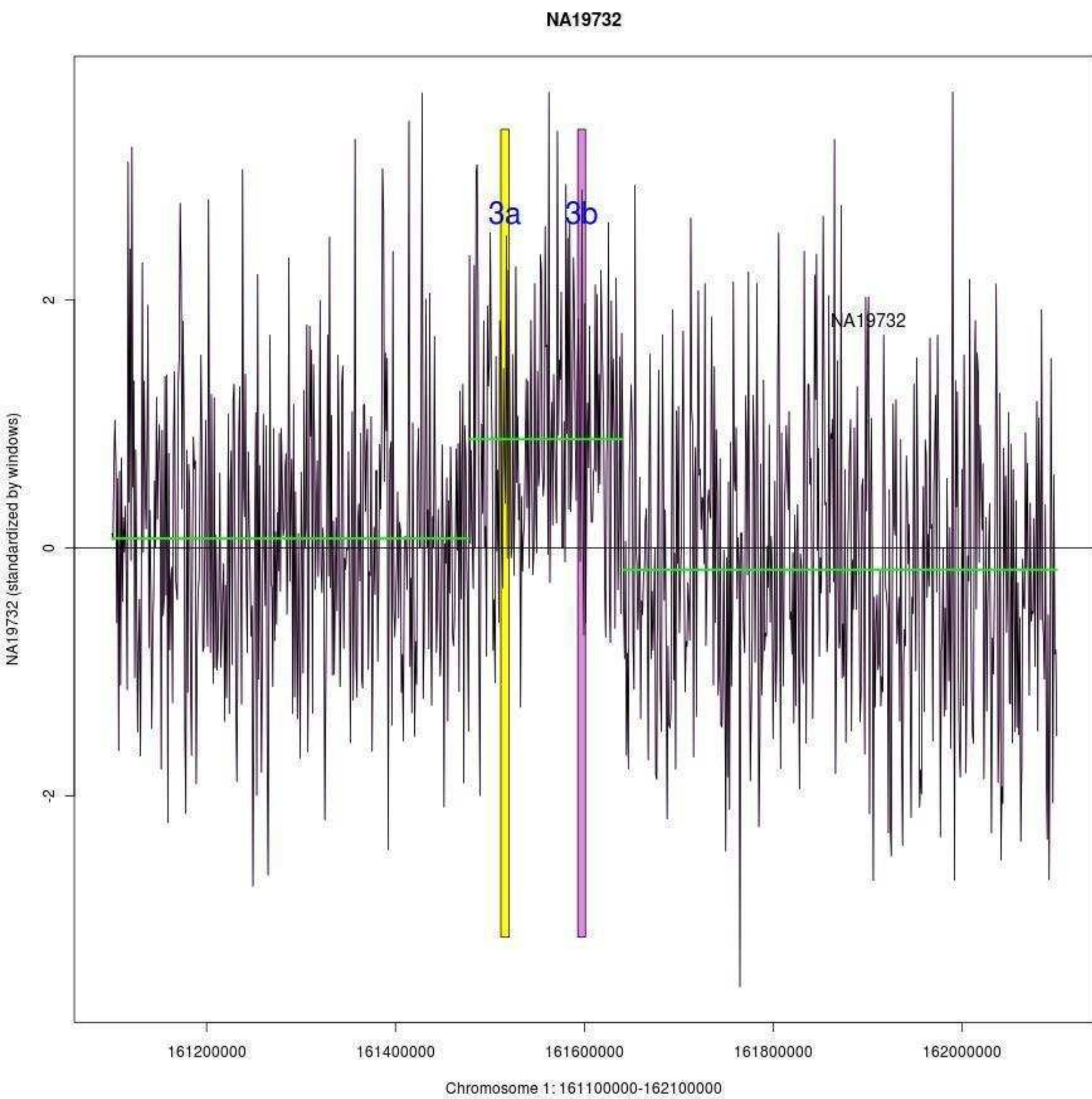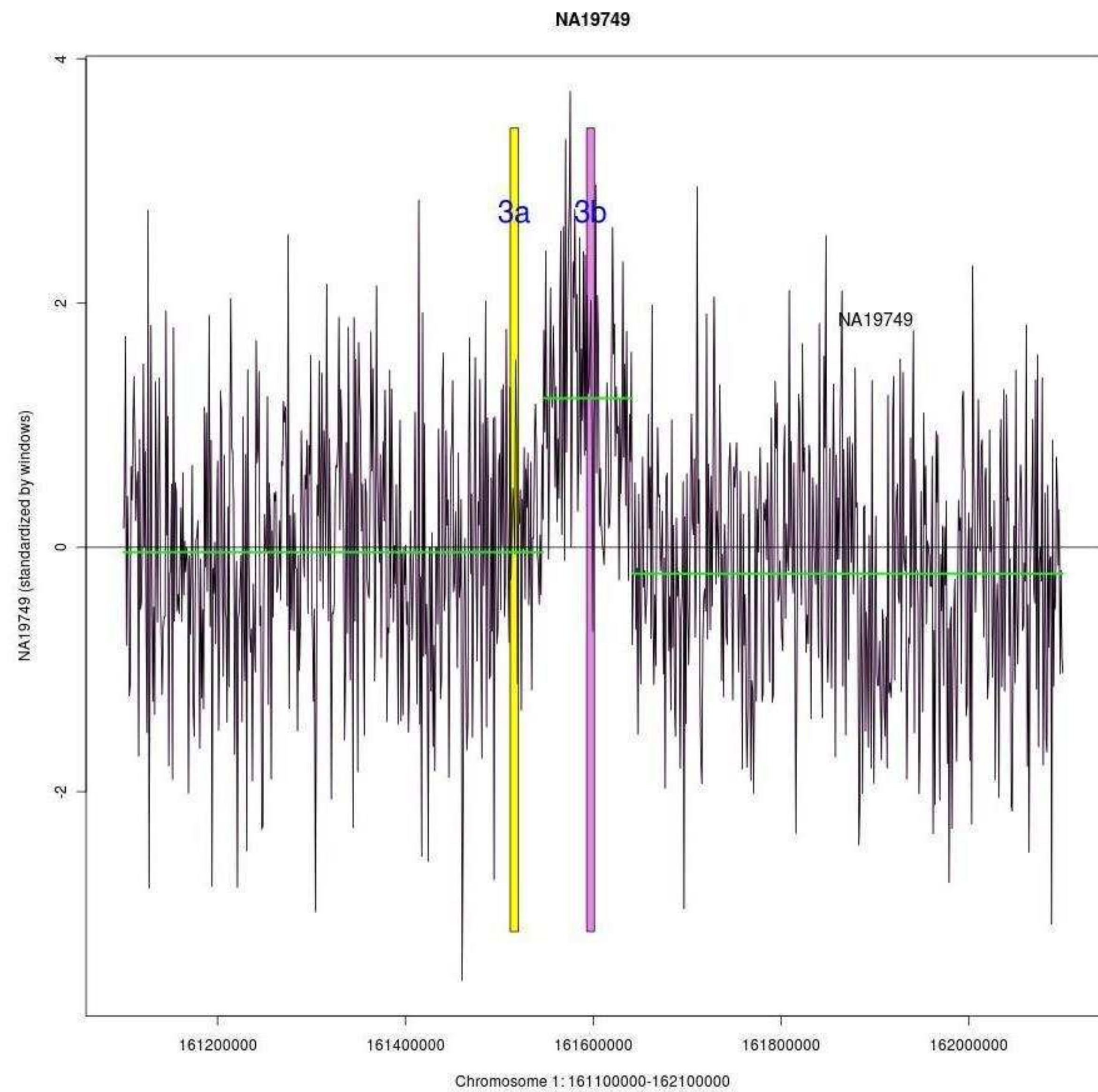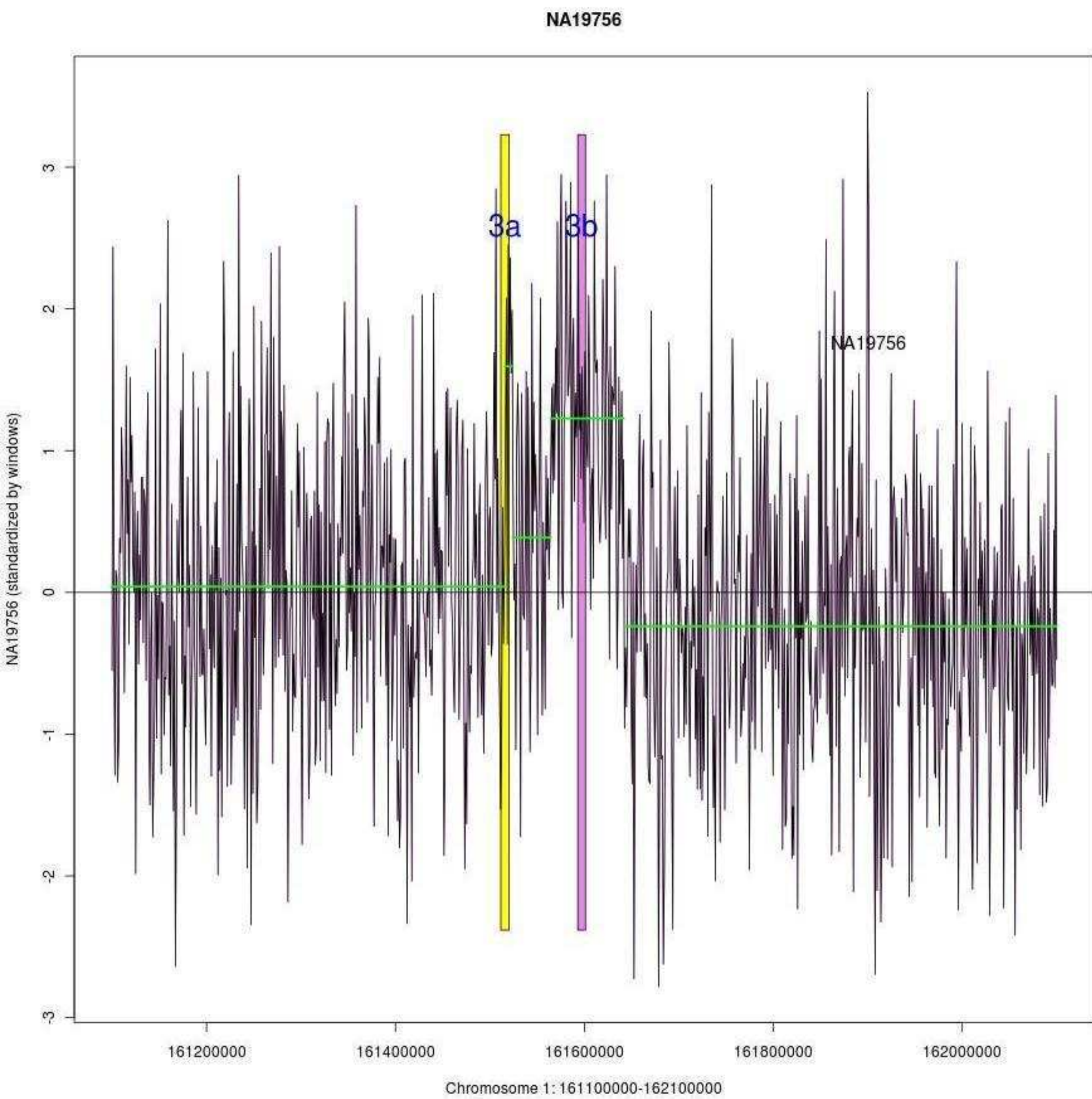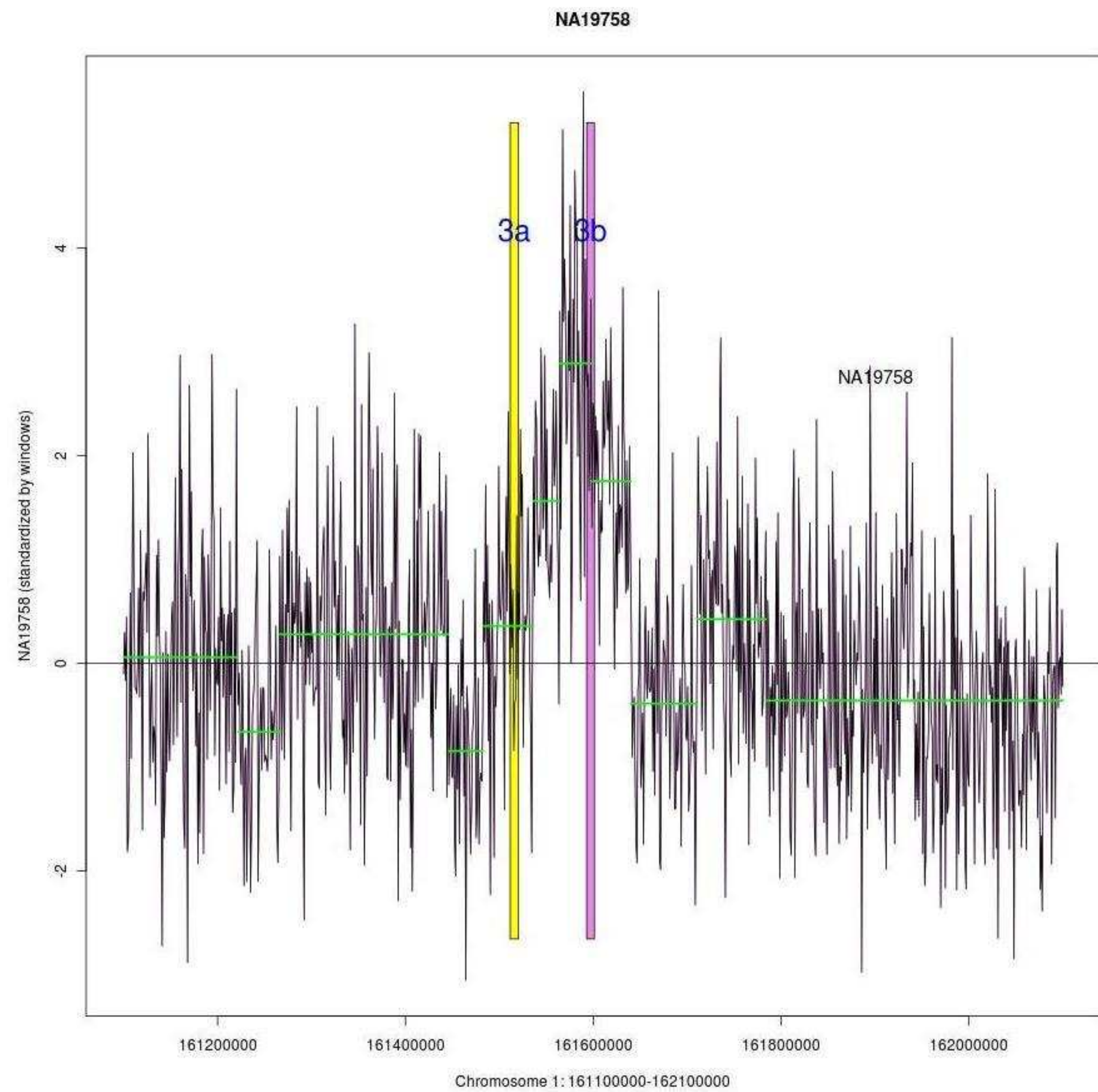

NA19762

NA19762 (standardized by windows)

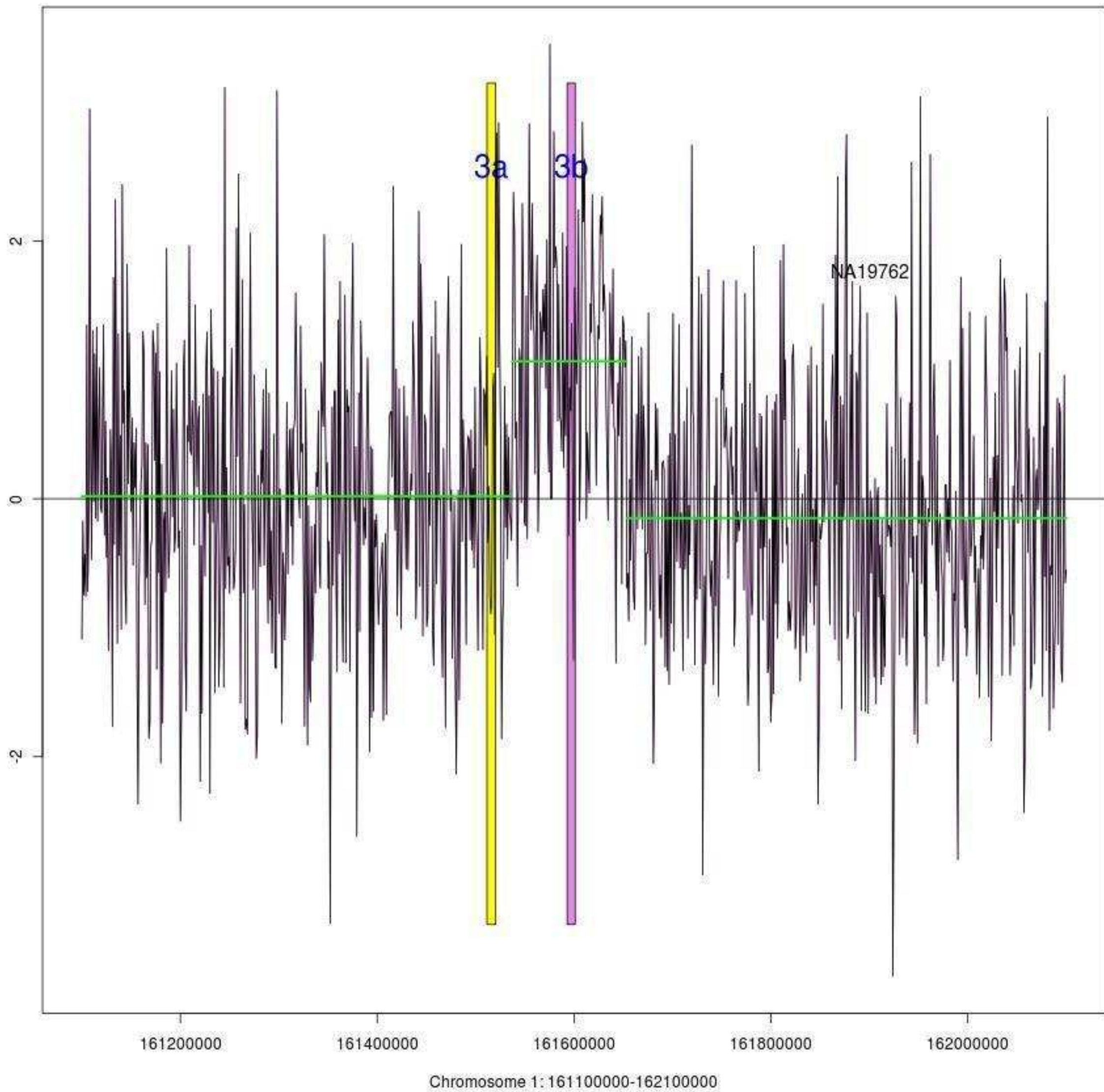

Supplement: Figure S5 — The 13 MXL individuals with FCGR3B duplication. The green horizontal lines are the mean values of regions from the step segmentation of the DNAcopy package. The file is Supplemental Figure 5a.pdf. (PDF) [file pone.0063219.s005.pdf]

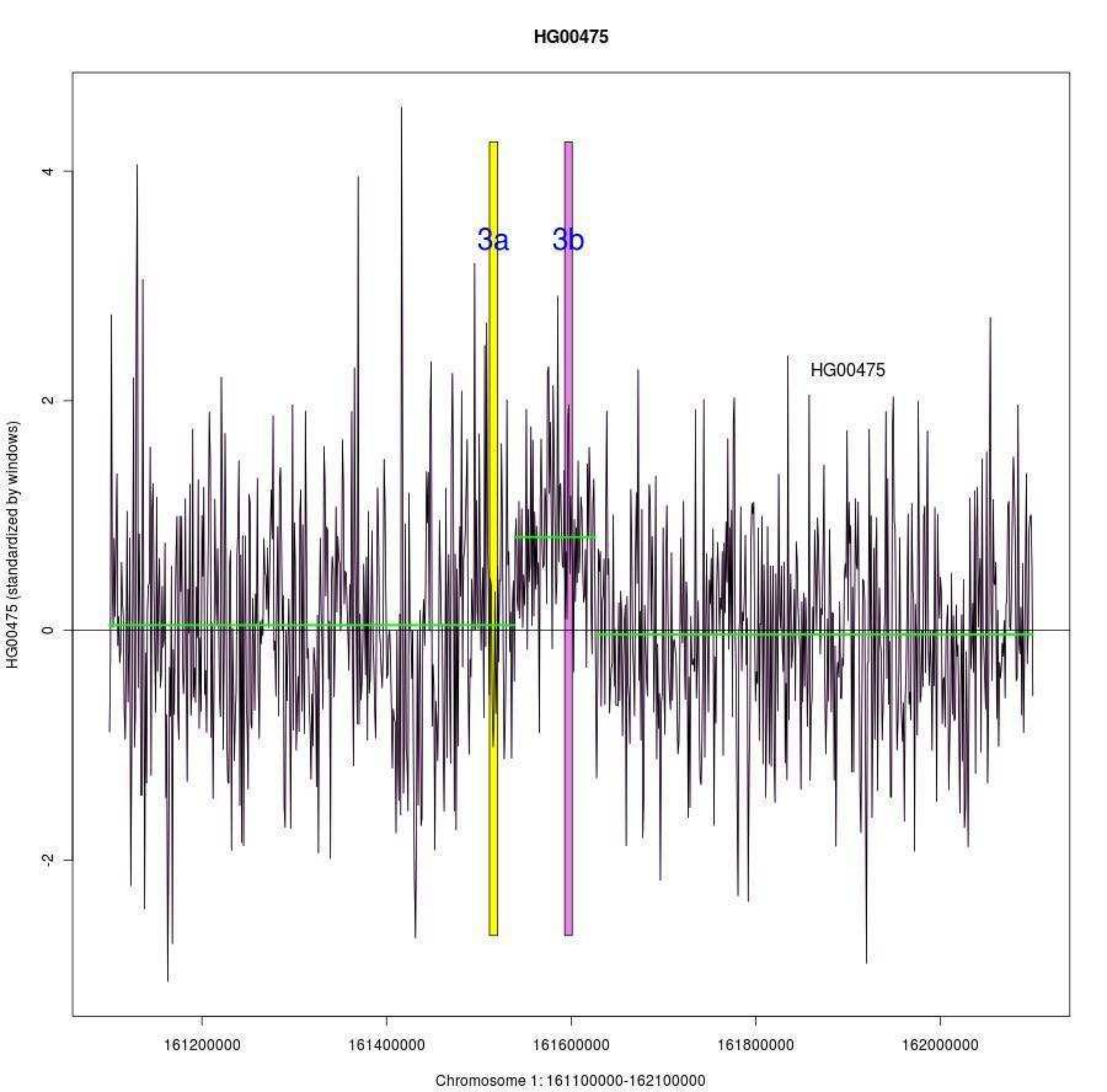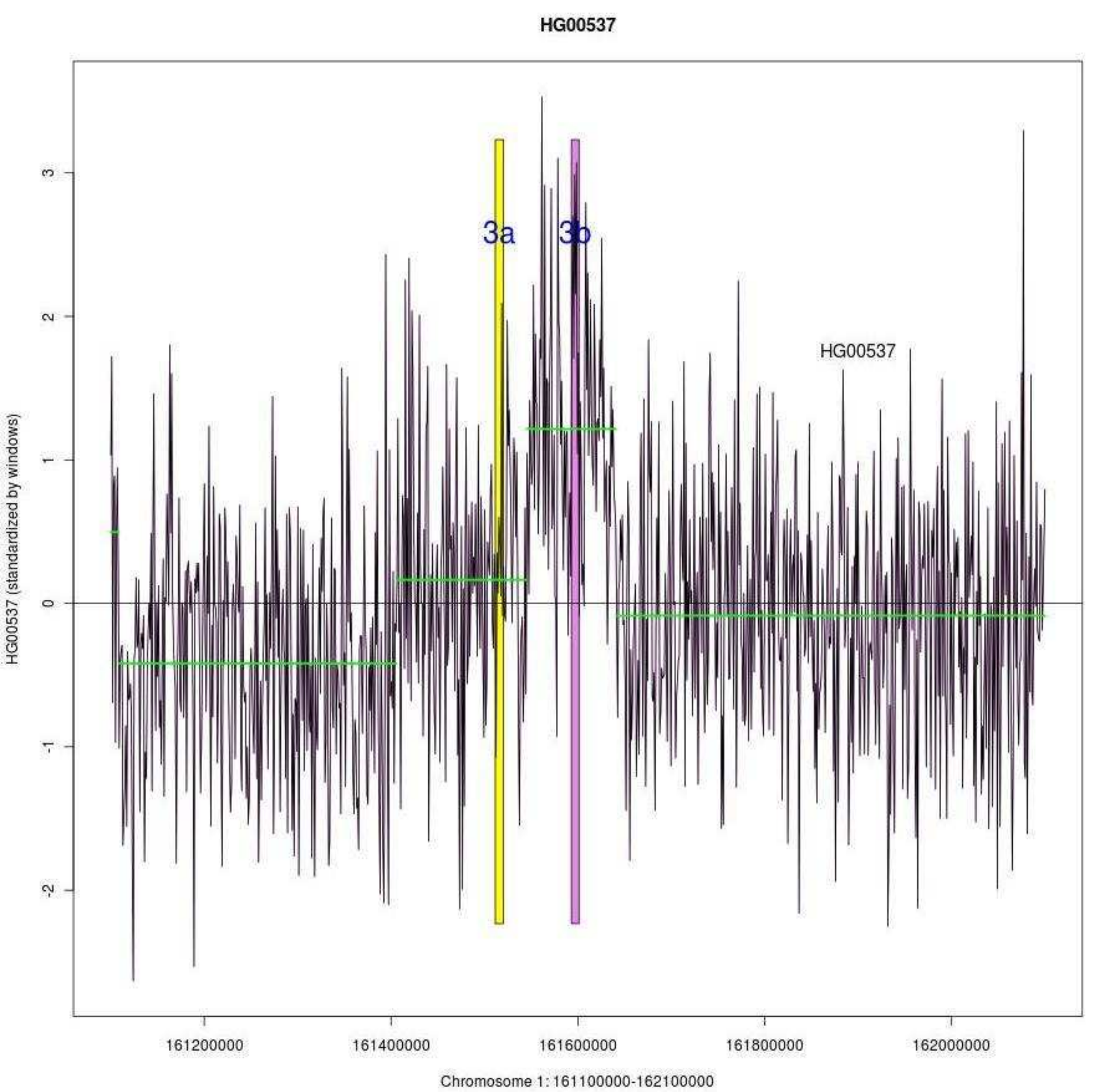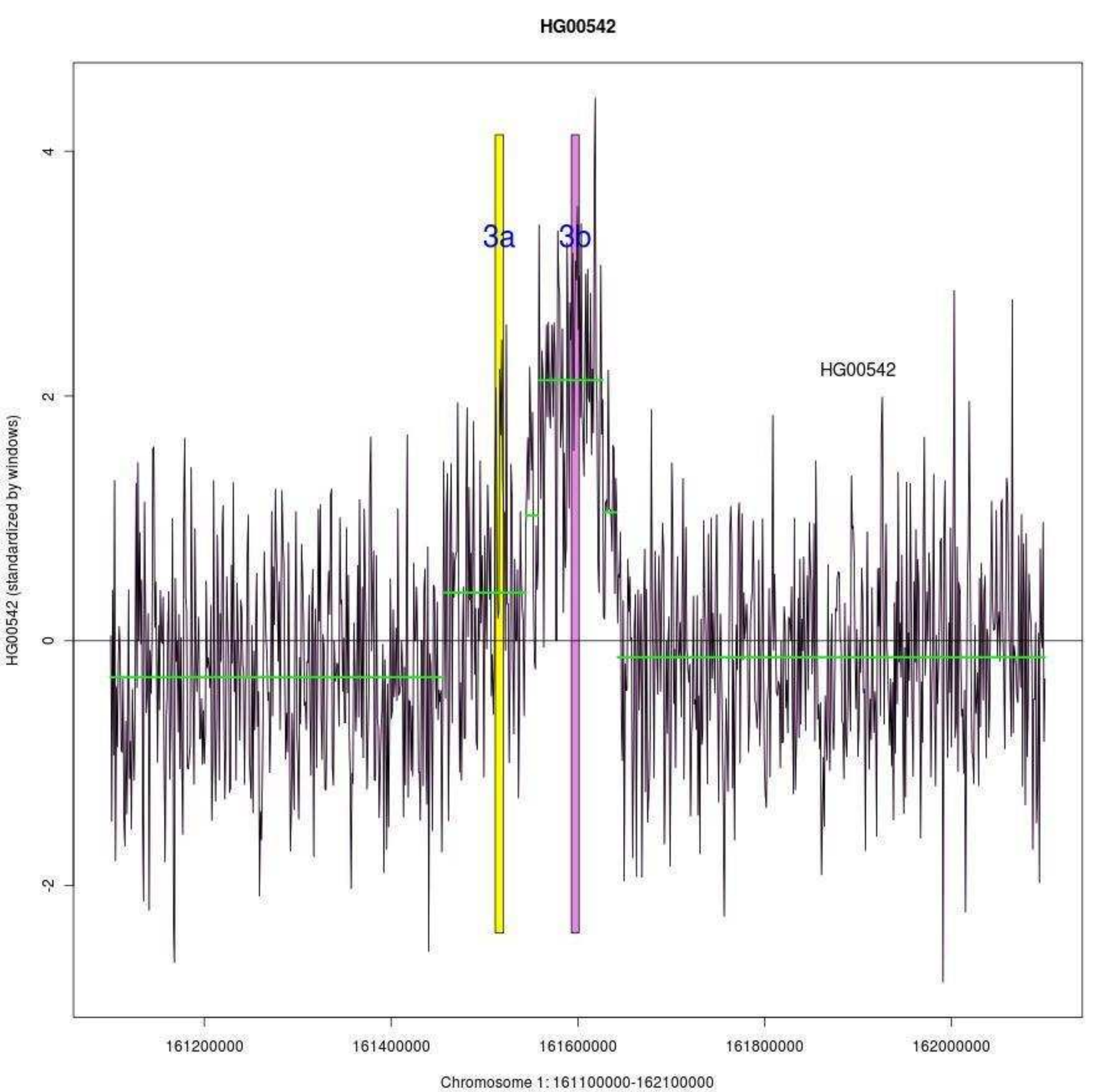

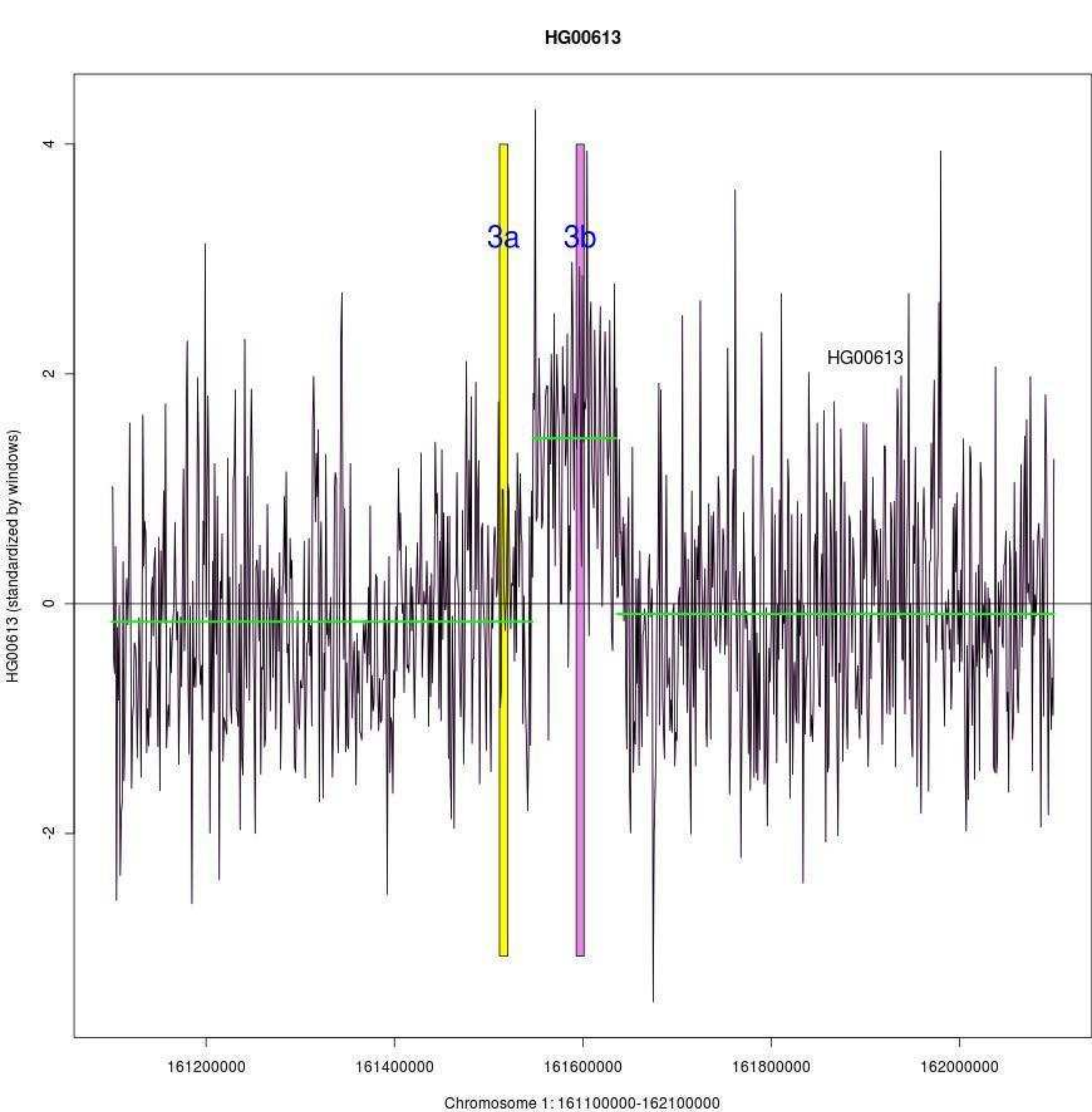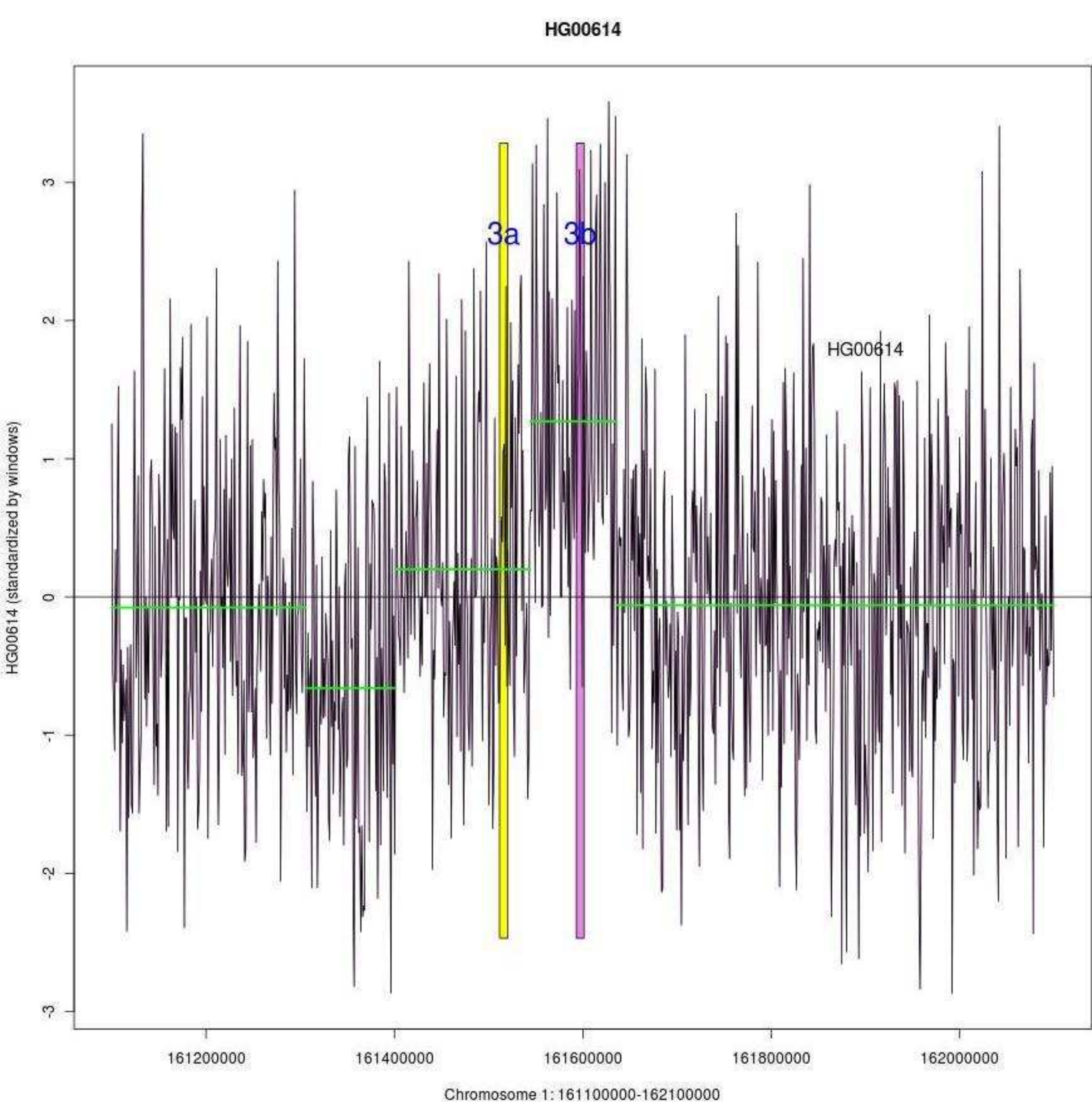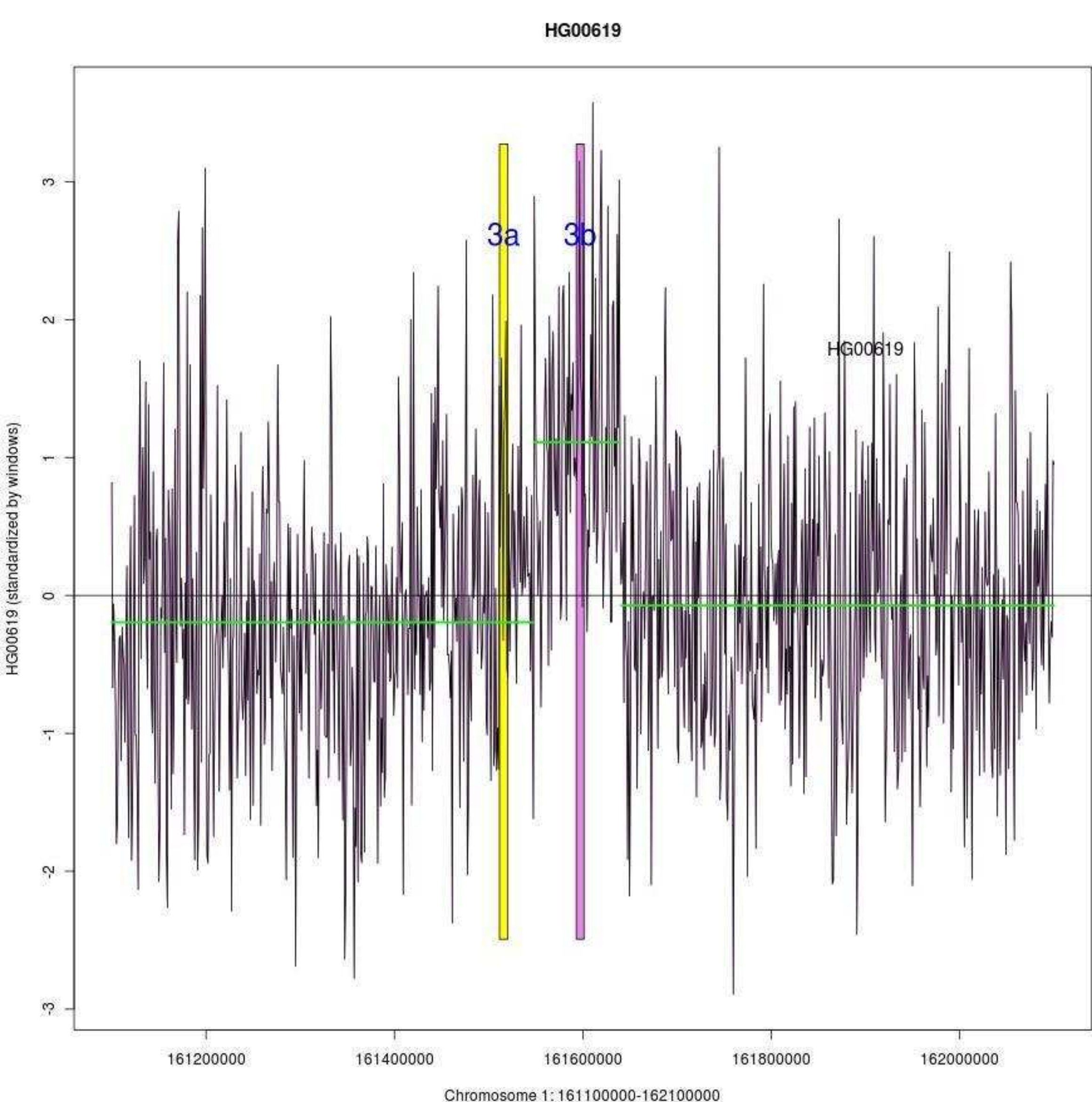

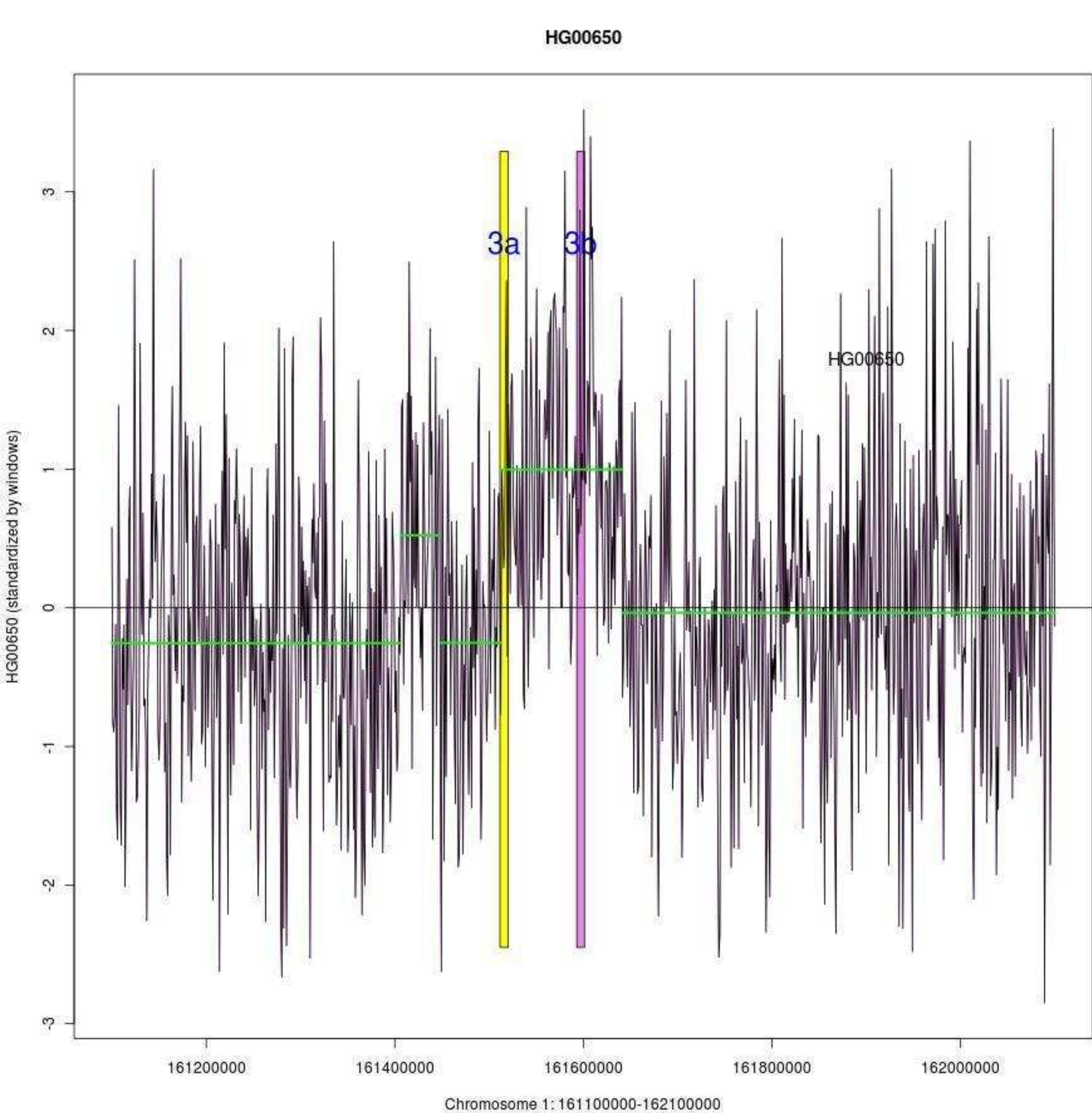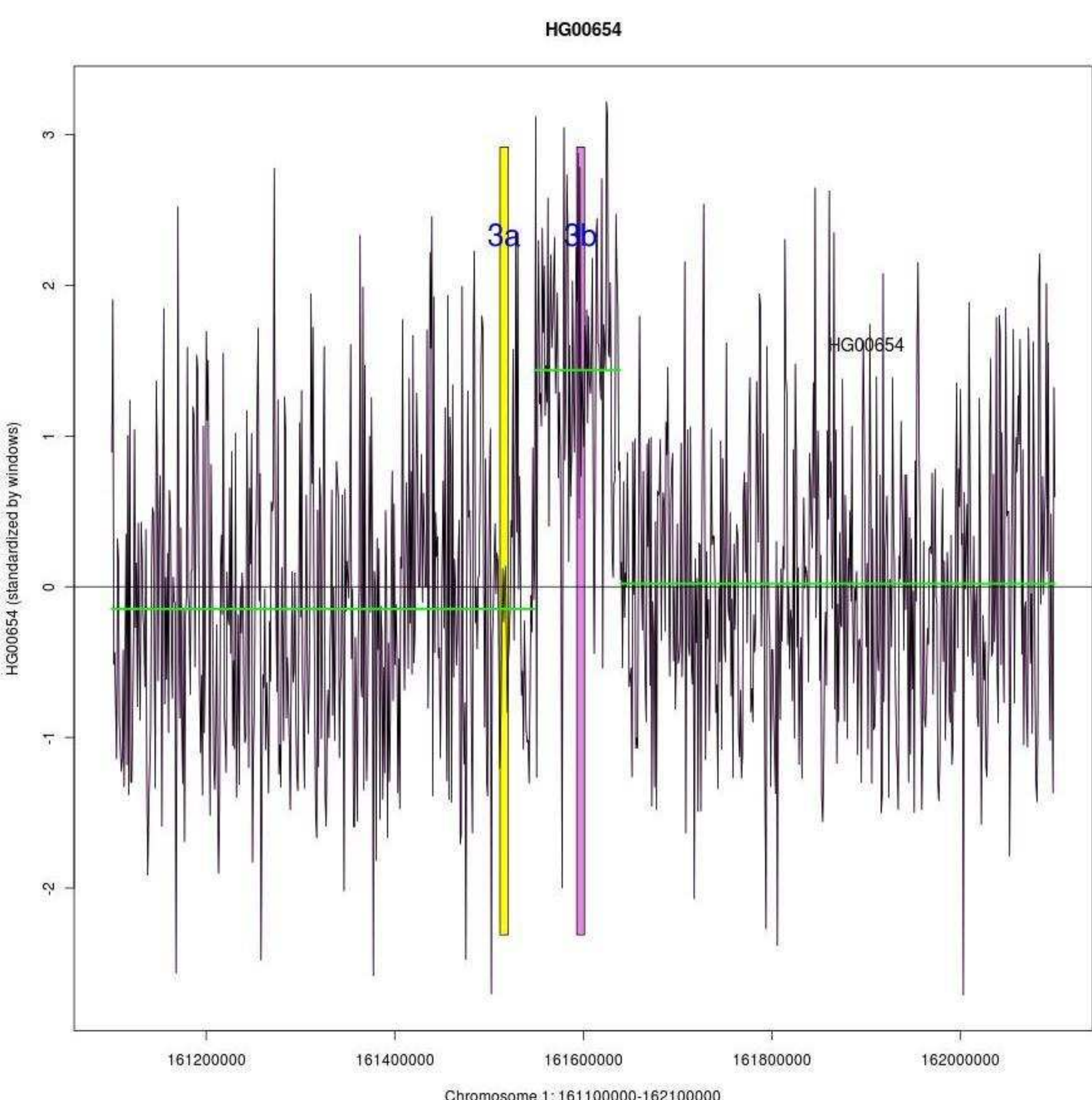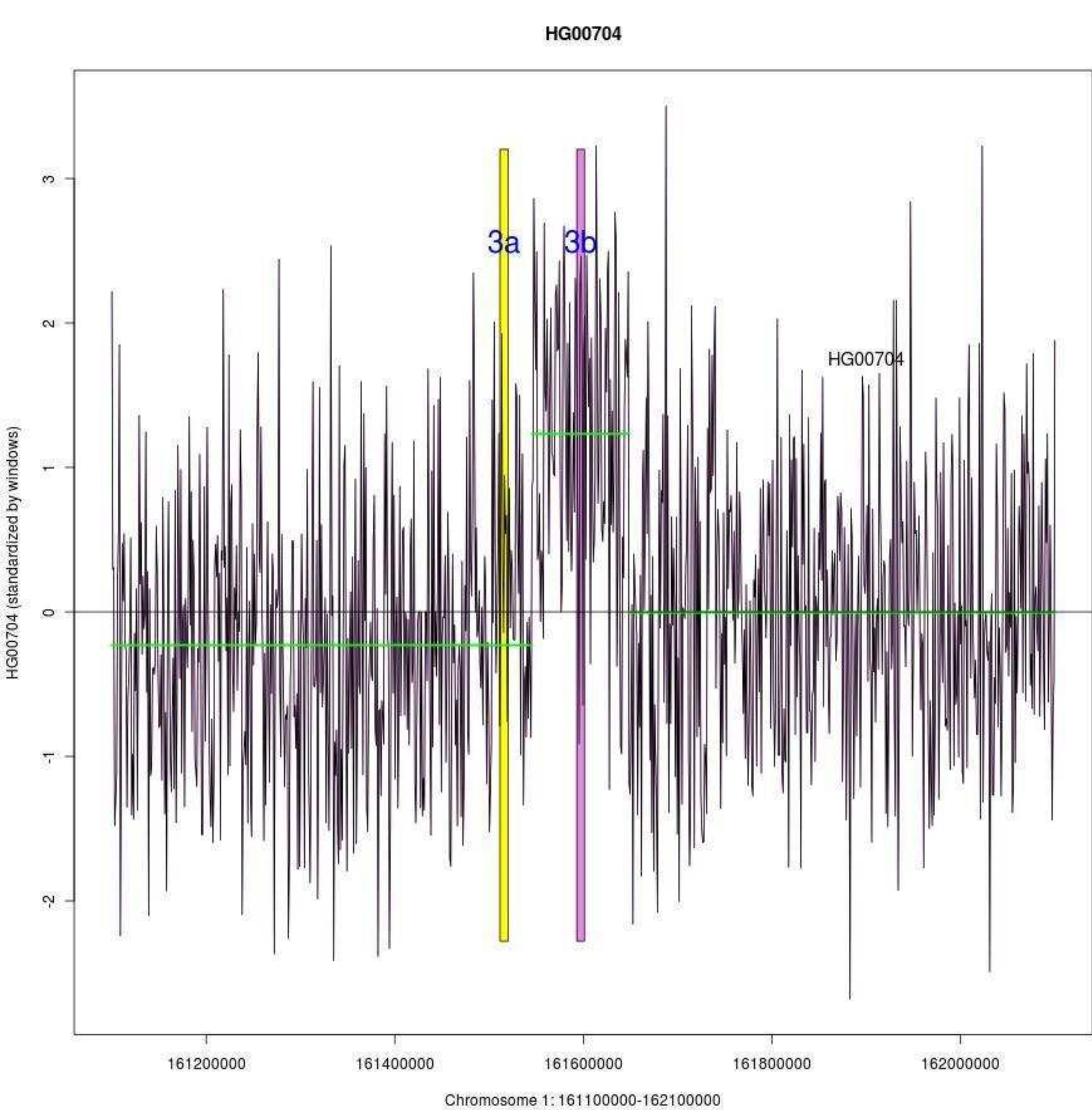

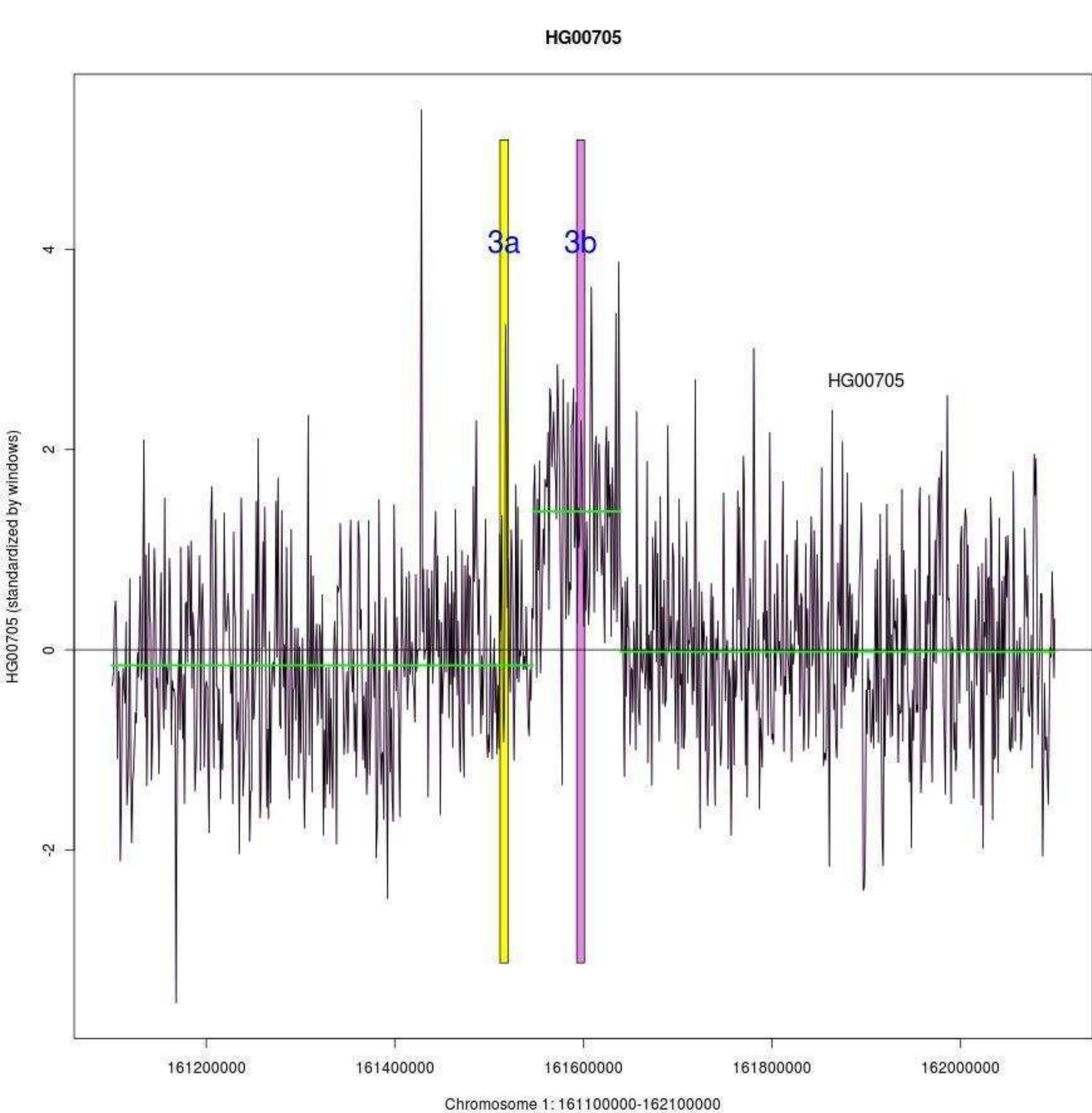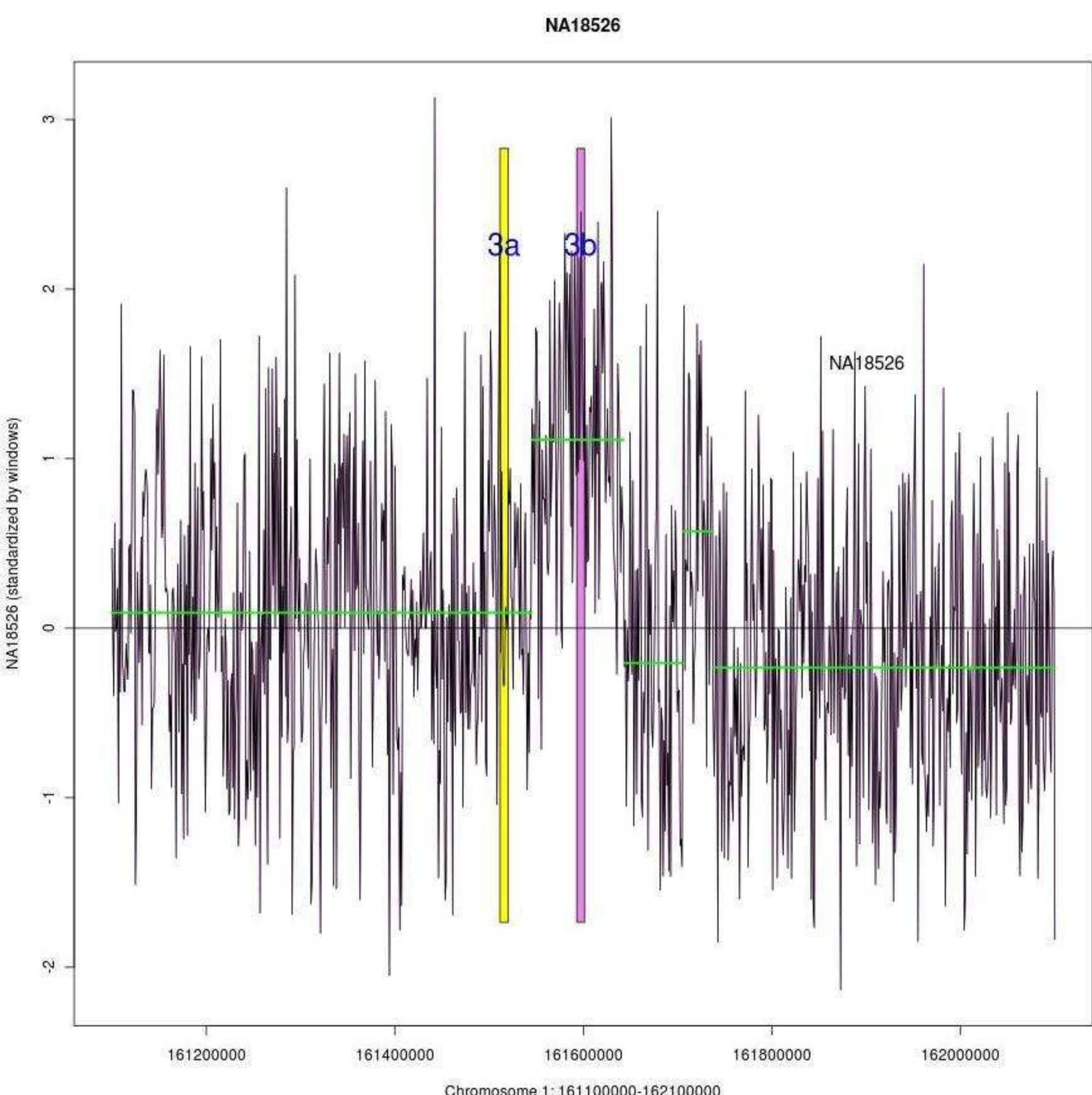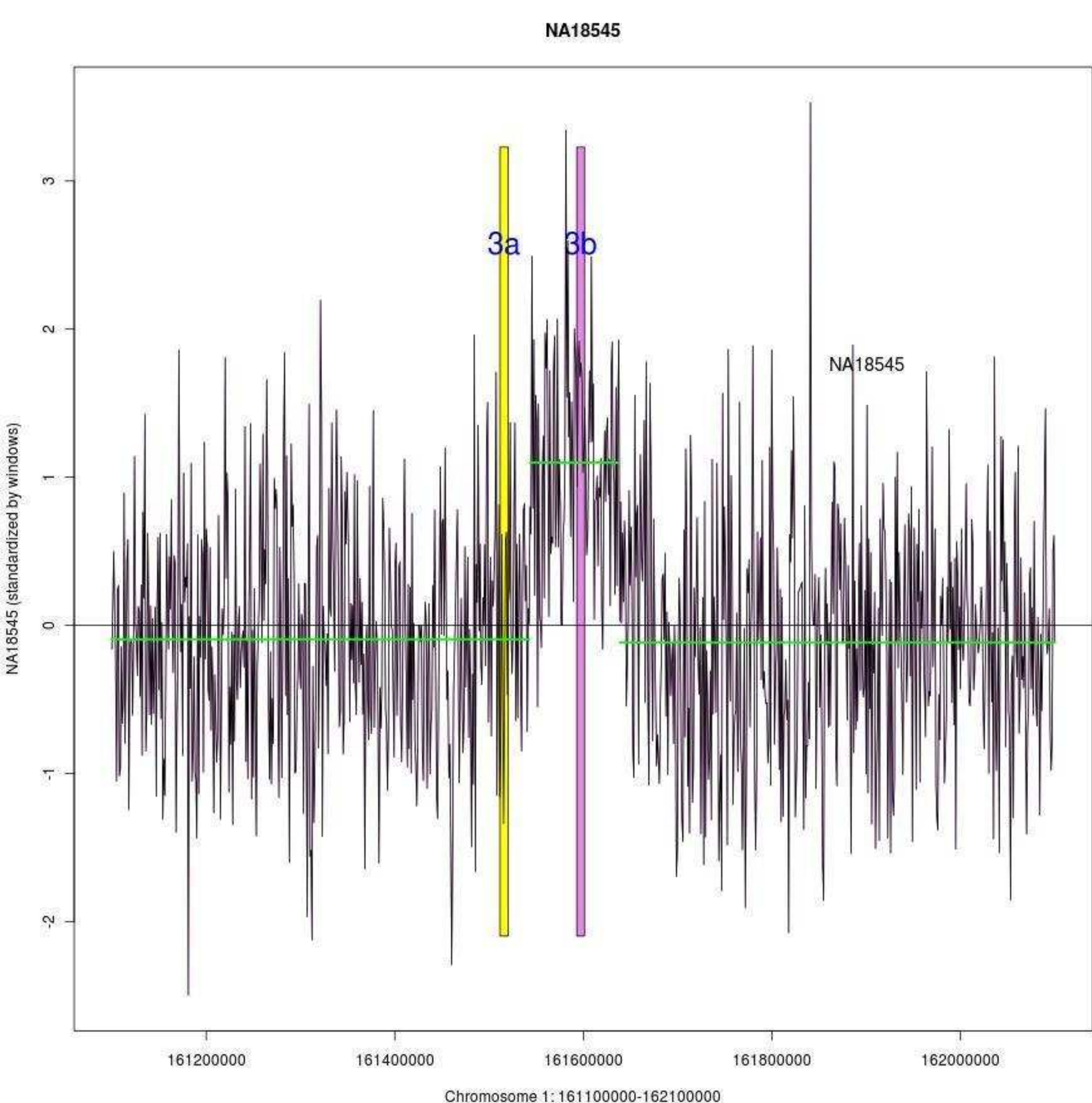

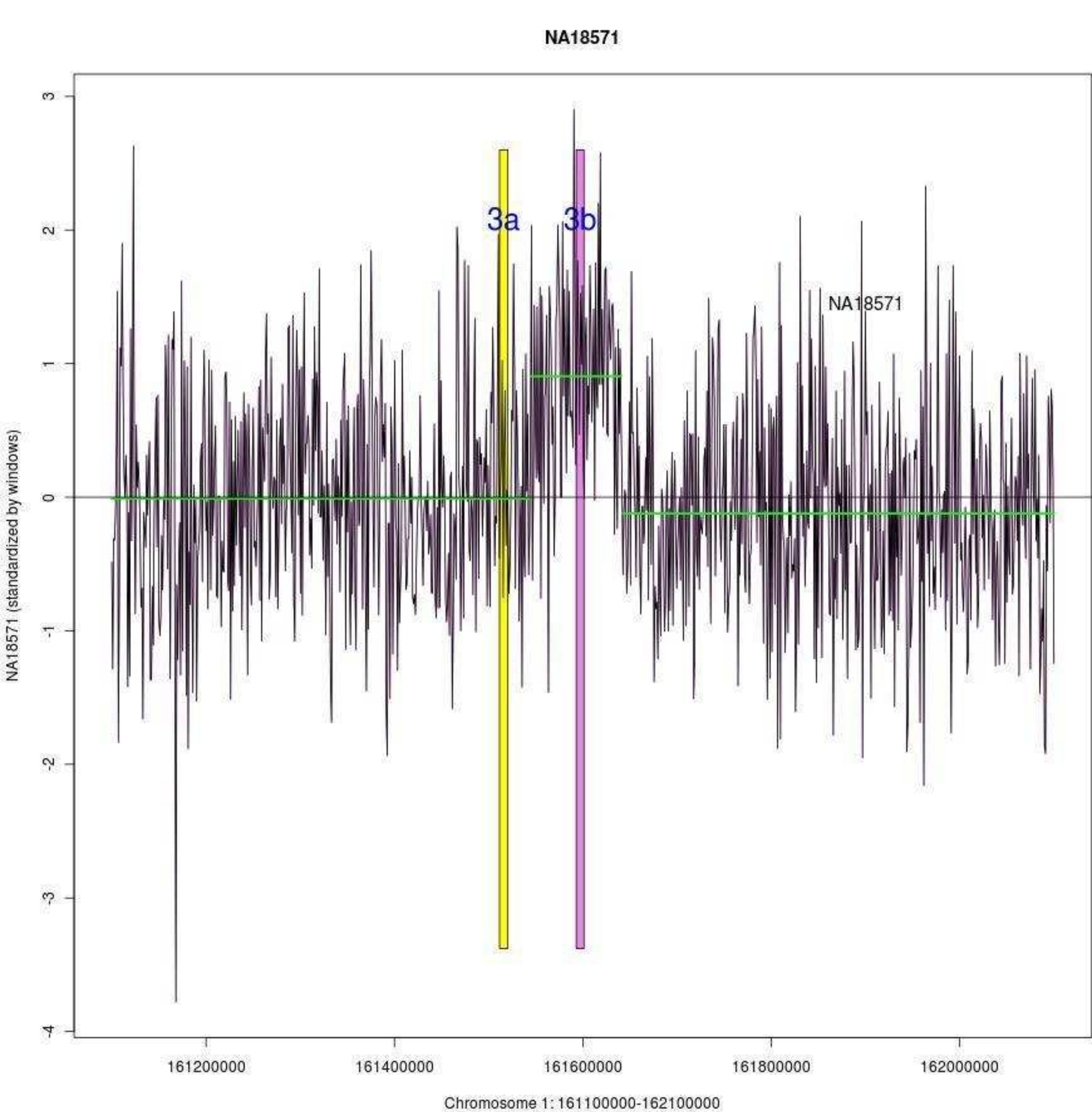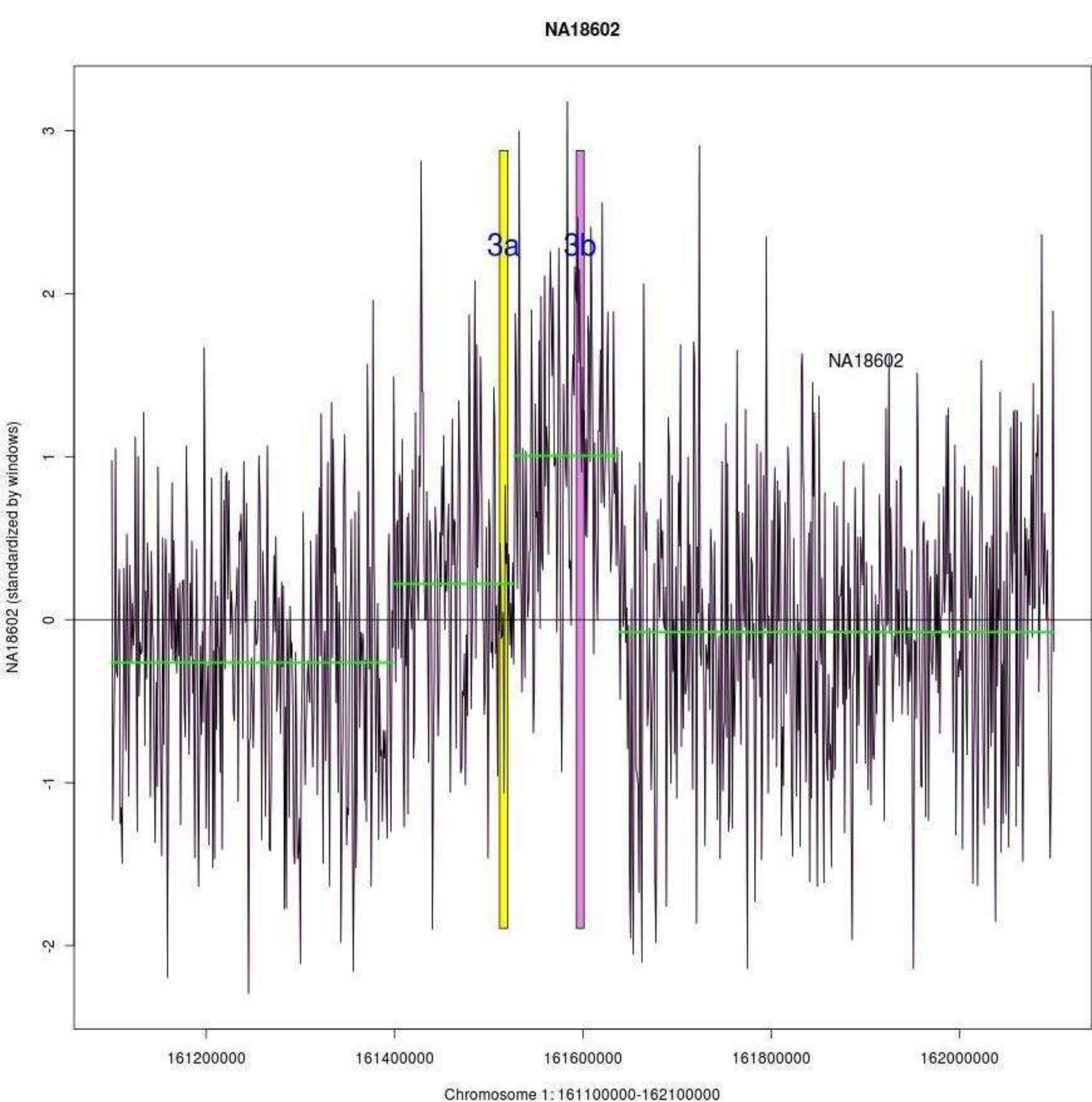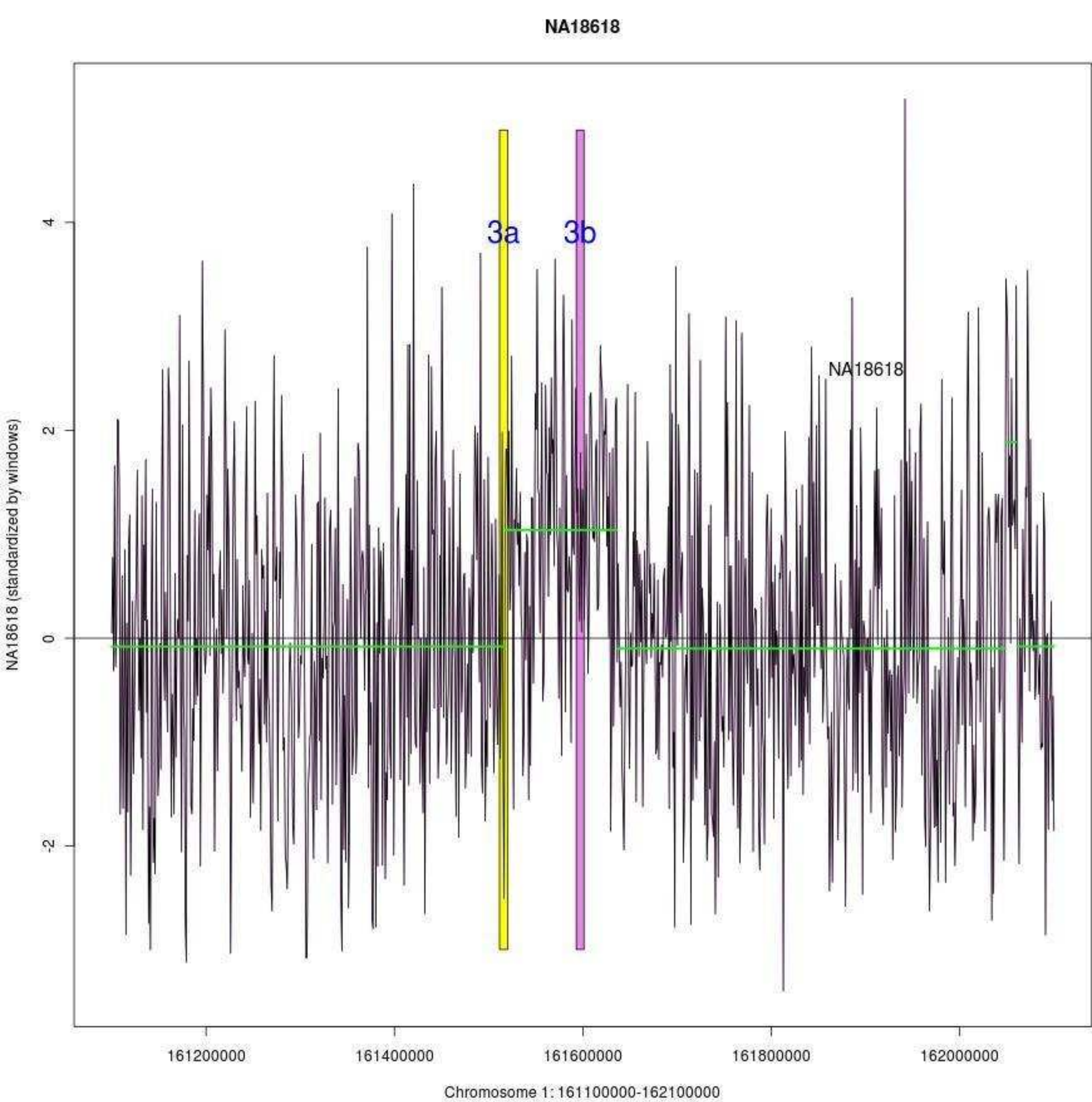

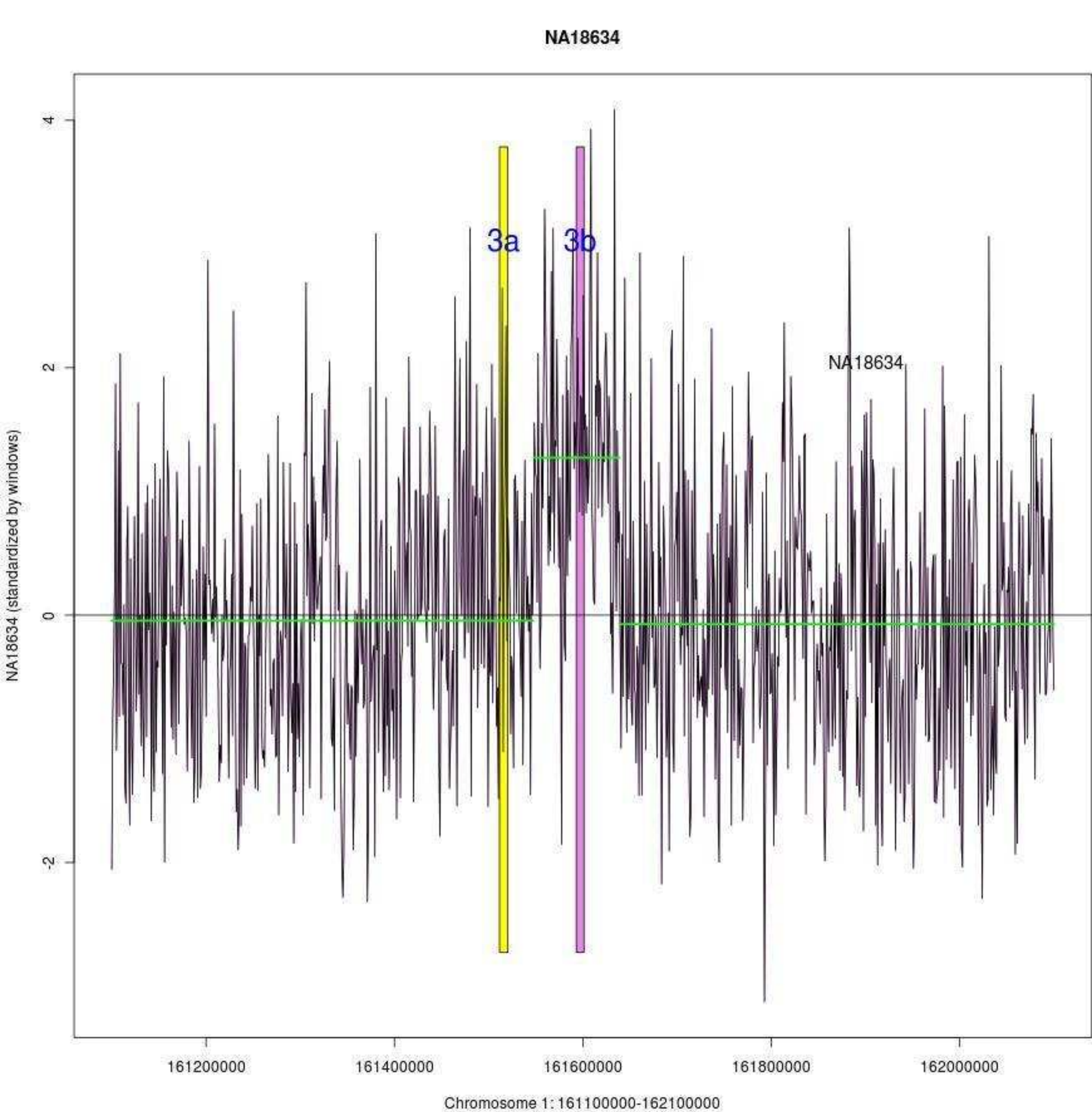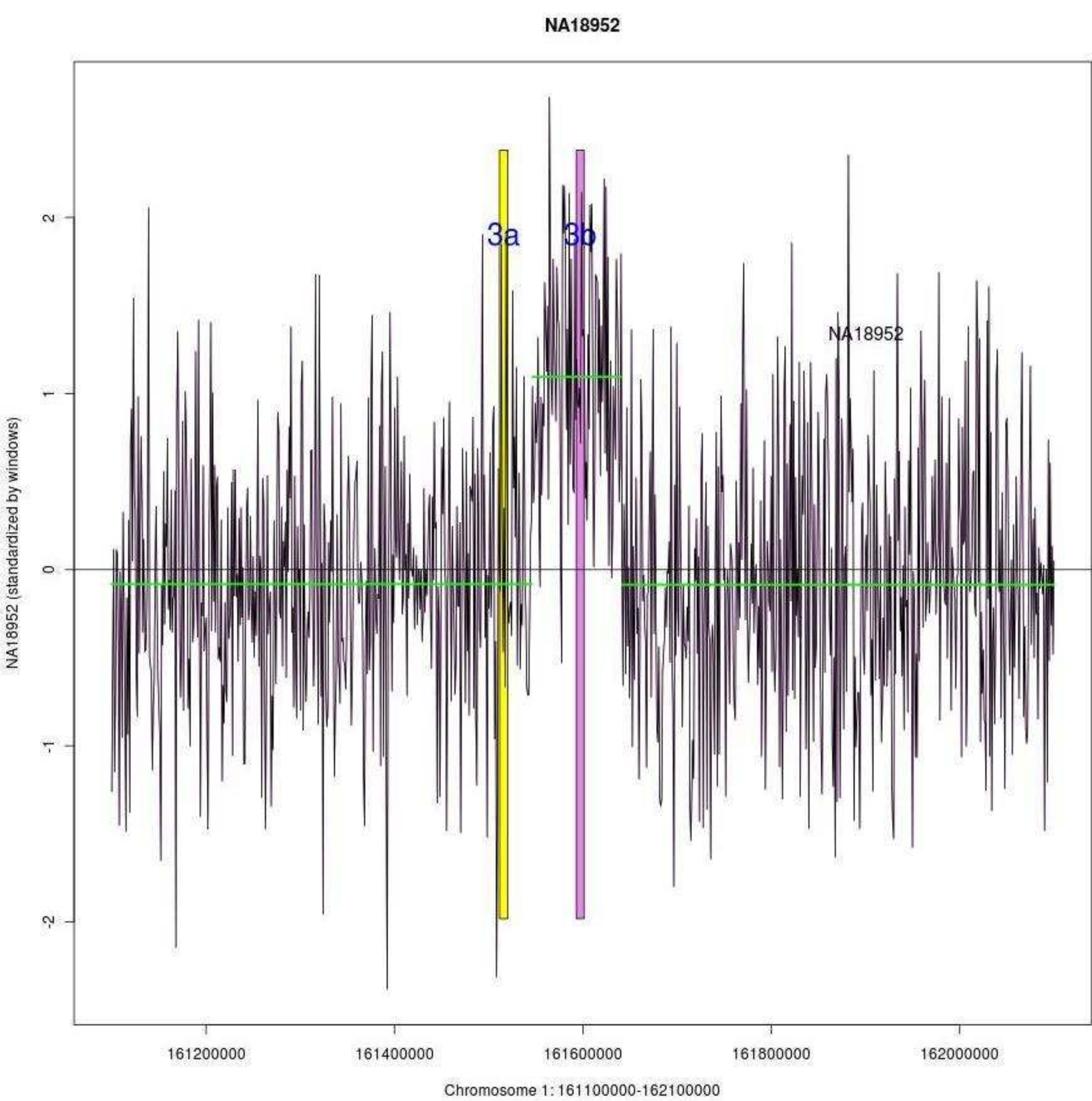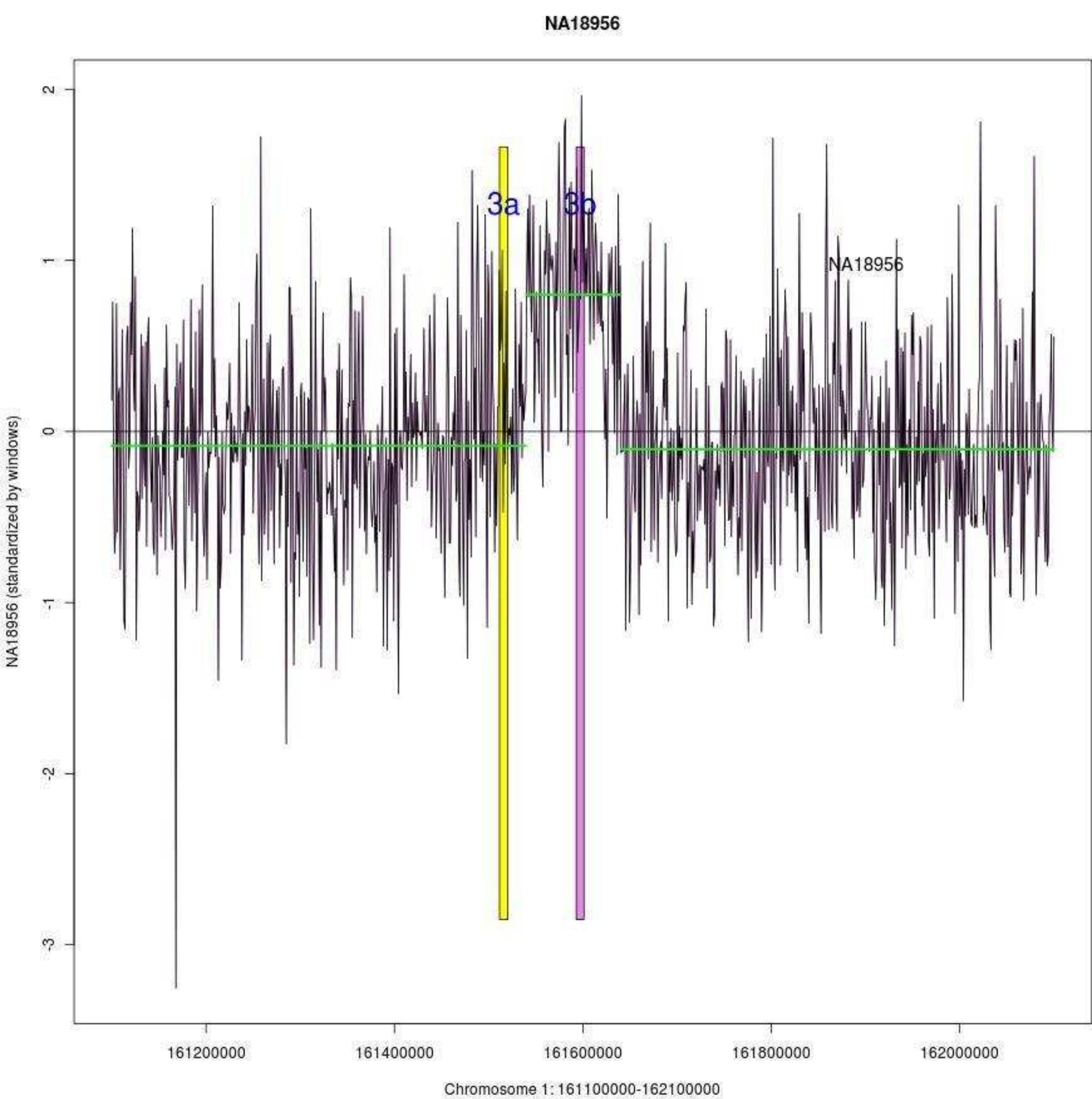

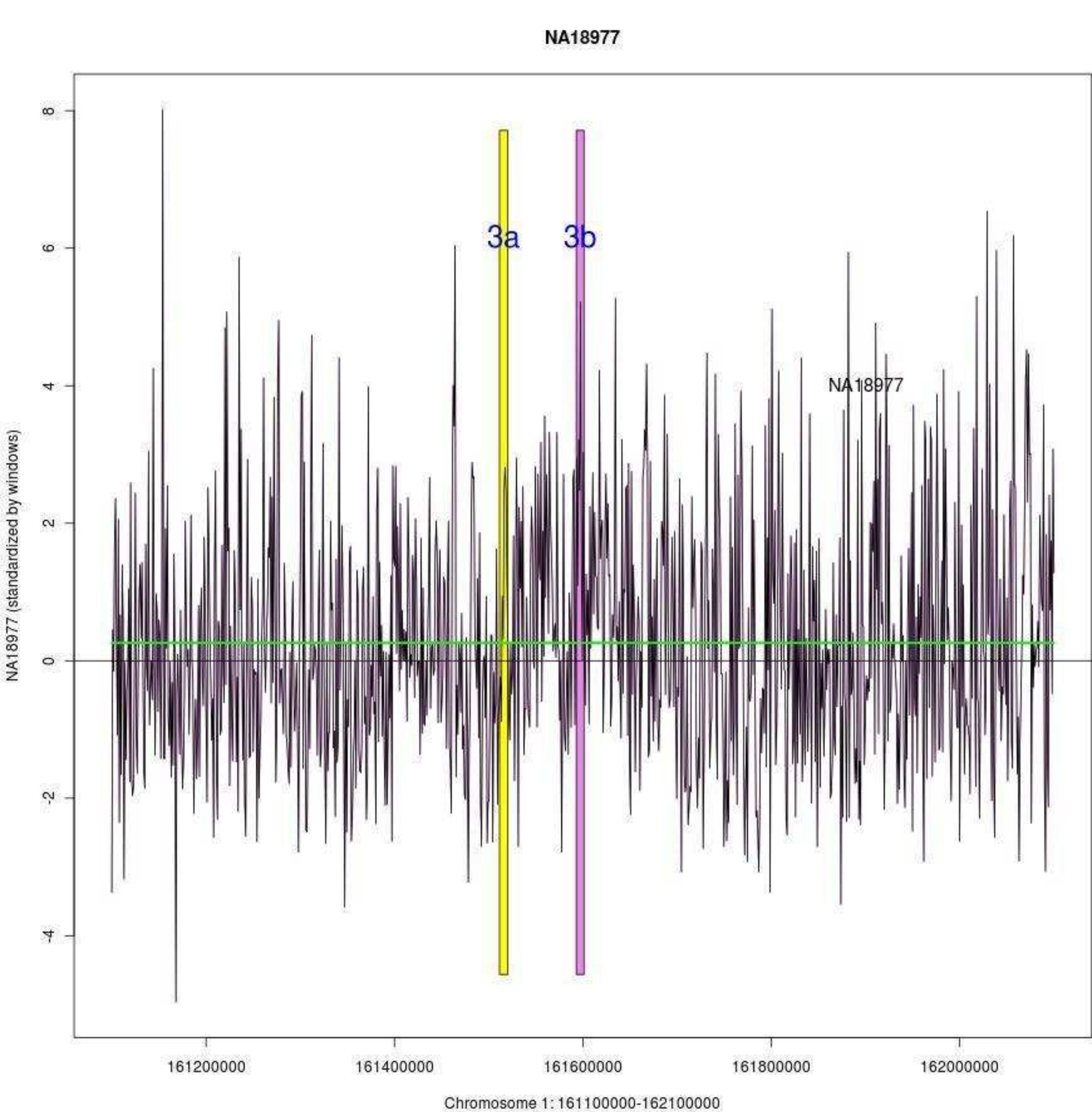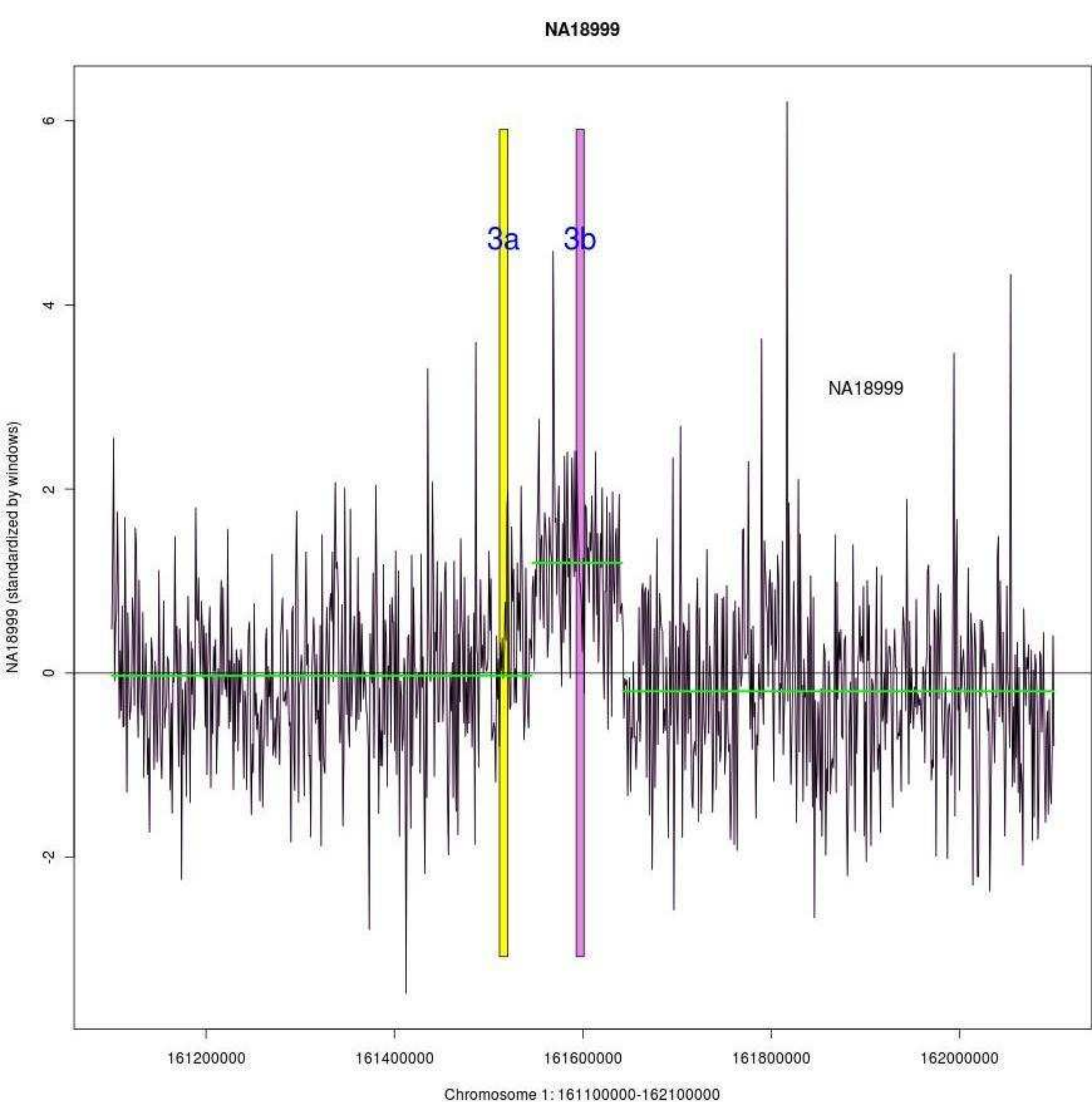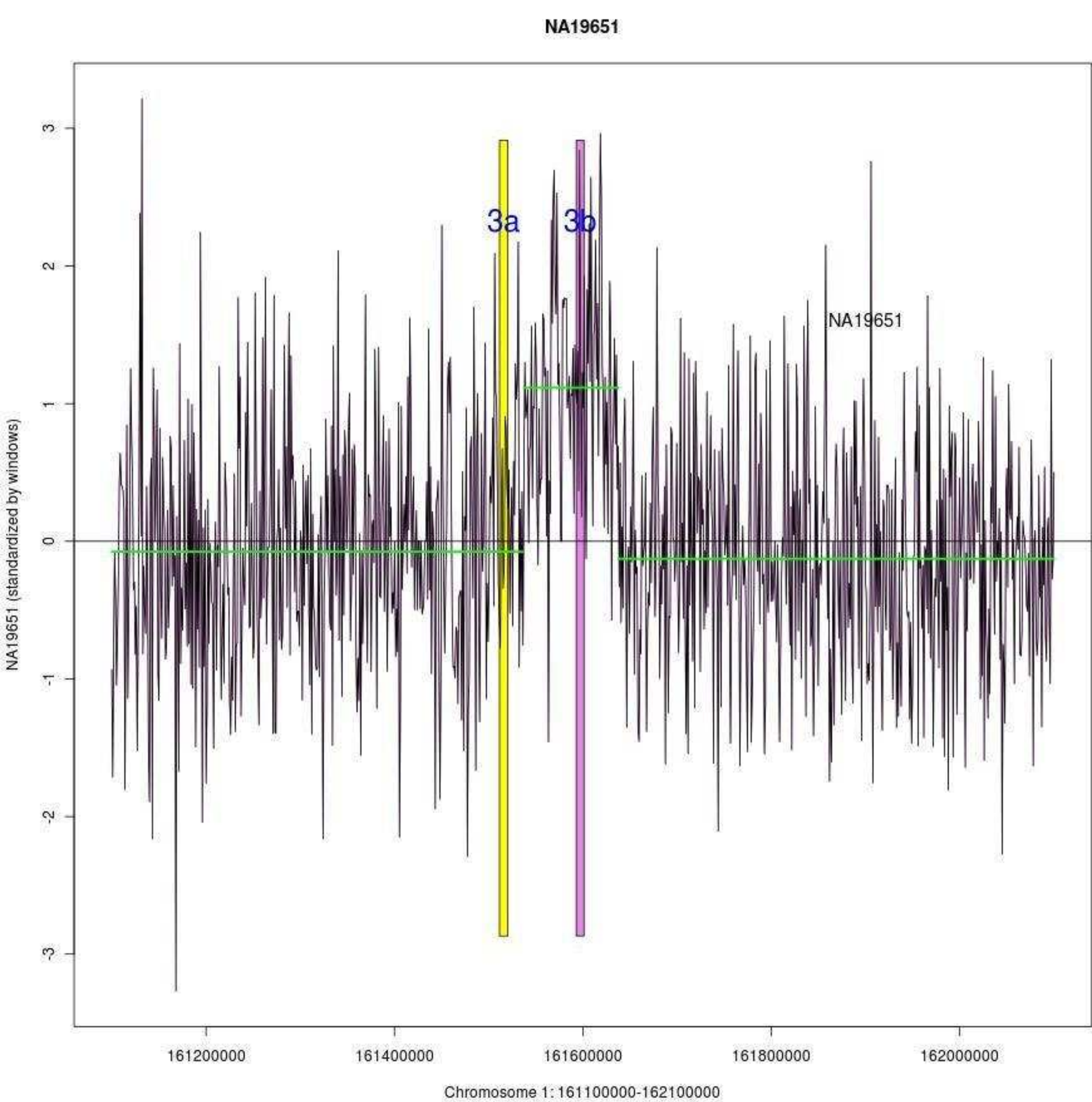

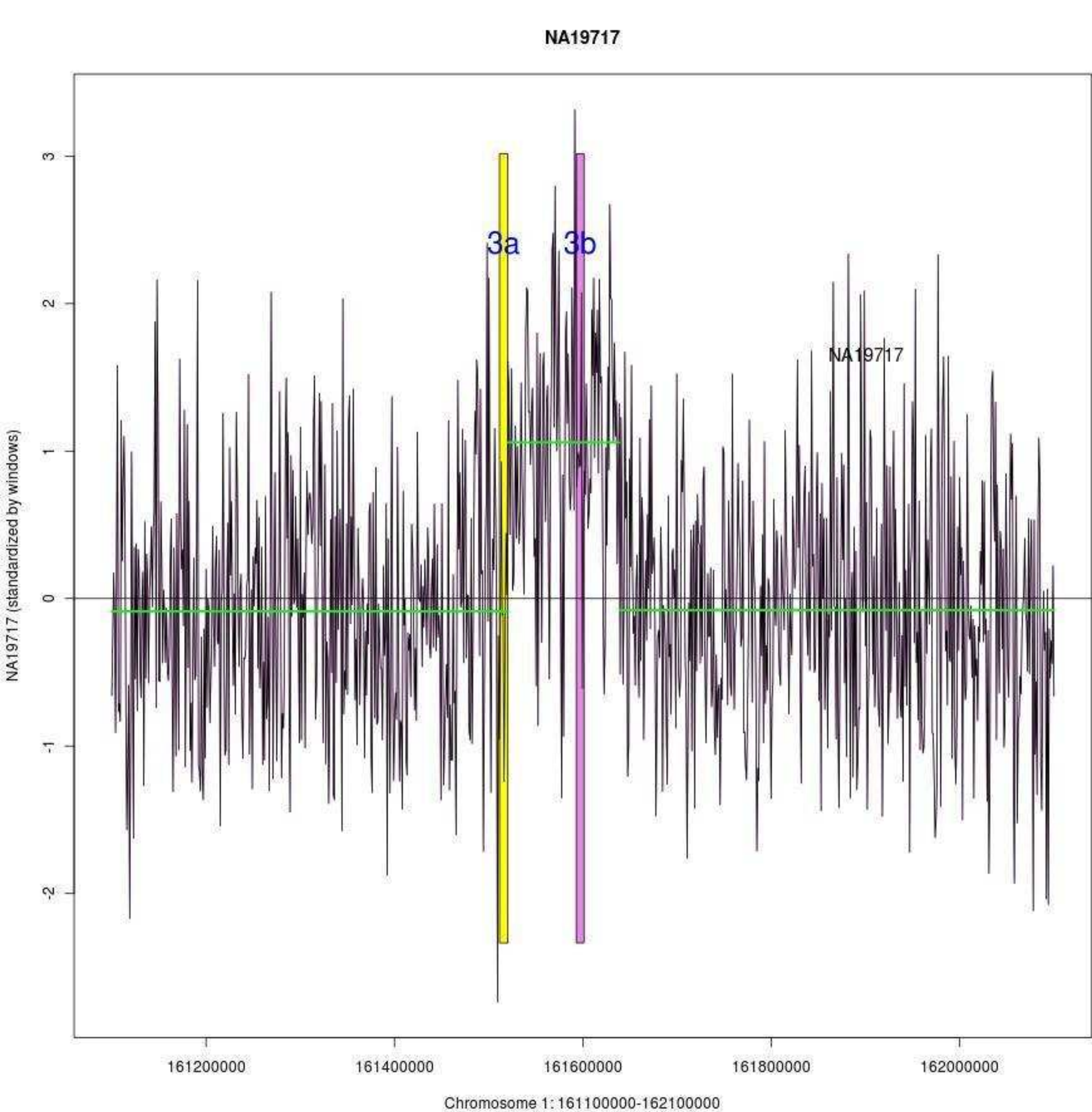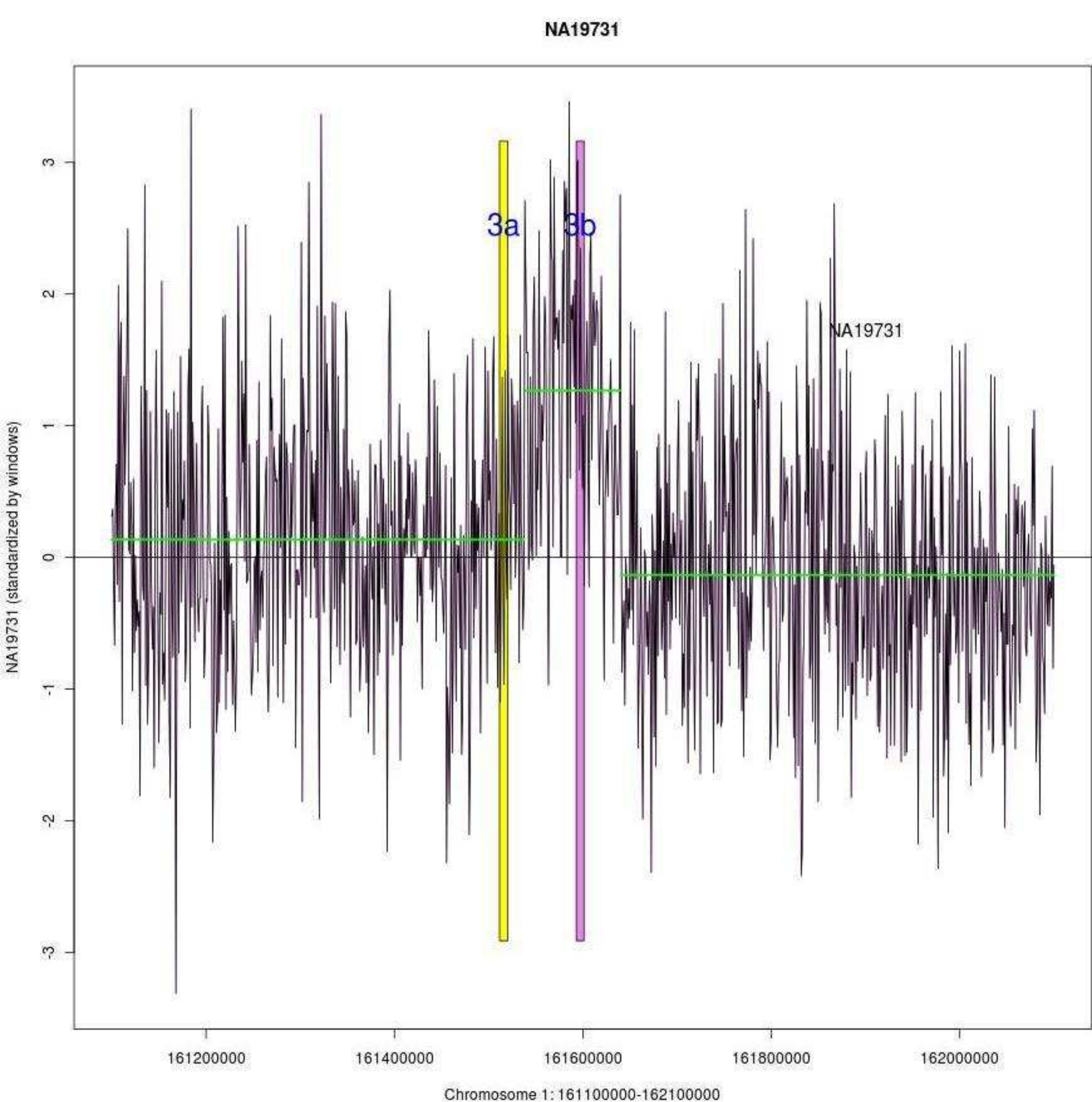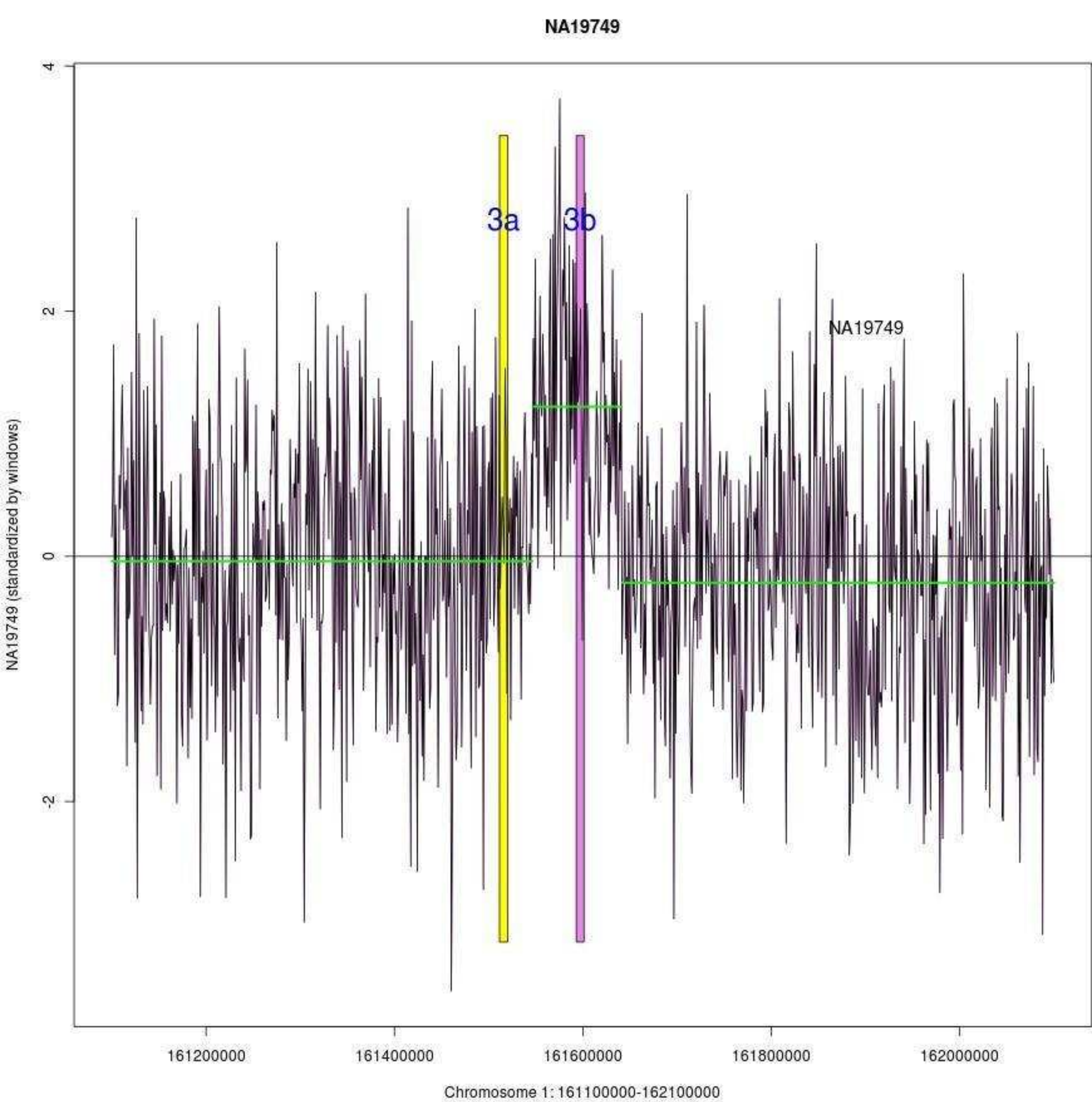

# NA19756

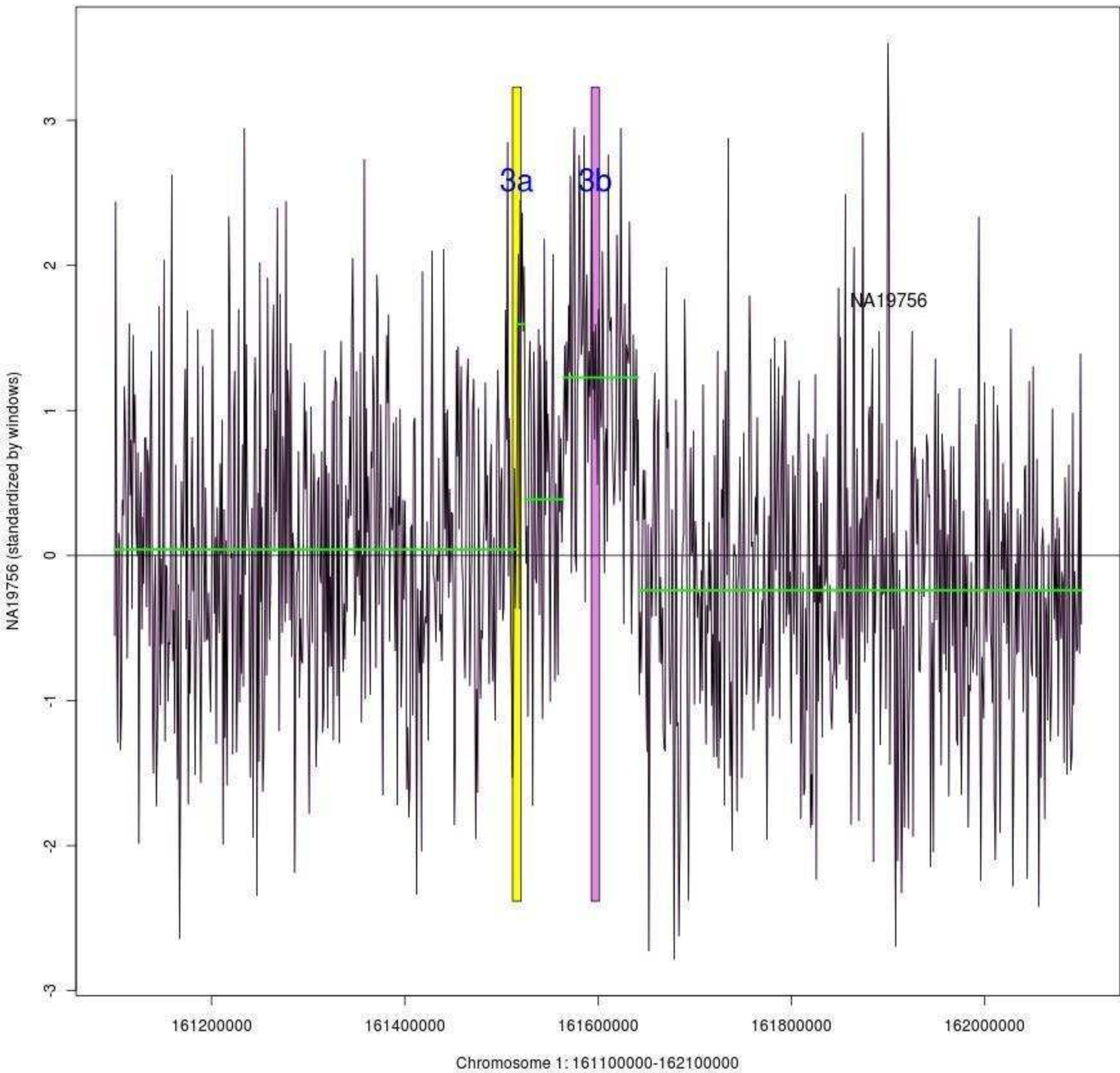

Supplement: Figure S6 — The 25 MXL/CHB/CHS/JPT individuals CN = 2 at FCGR3A, CN = 3 at FCGR3B with minor allele of rs117435514. The green horizontal lines are the mean values of regions from the step segmentation of the DNAcopy package. The file is Supplemental Figure 5b.pdf. (PDF) [file pone.0063219.s006.pdf]

**FCGR2A**

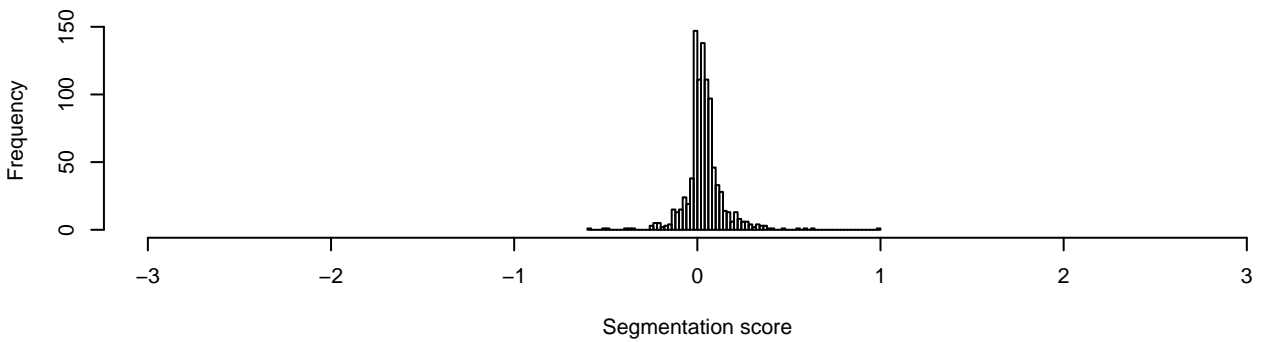

**FCGR2B**

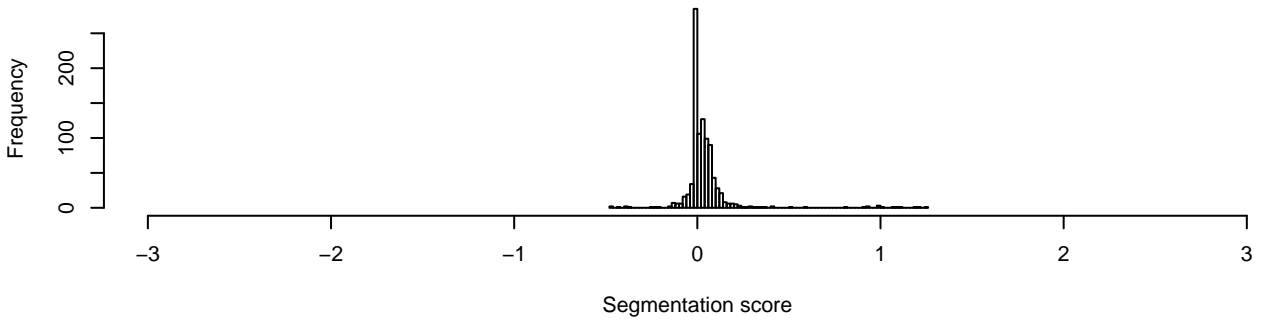

**FCGR2C**

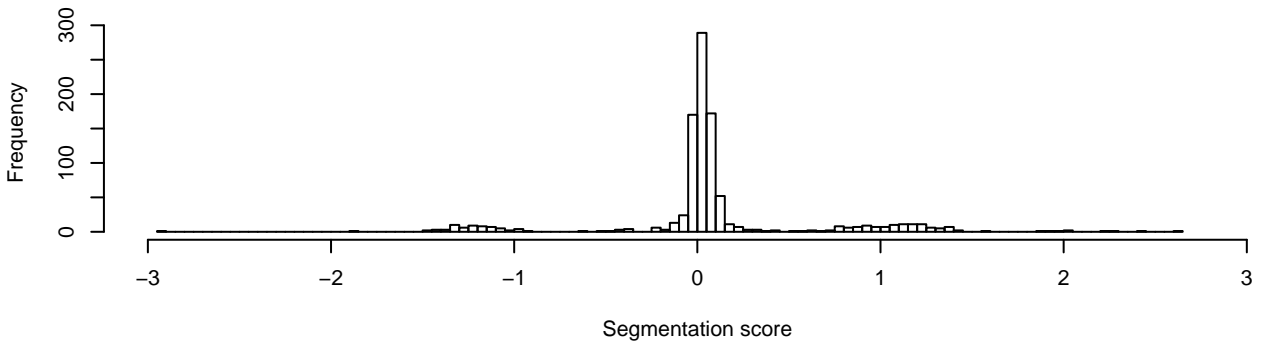

Supplement: Figure S7 — Segmentation scores at FCGR2A, FCGR2B and FCGR2C derived from DNAcopy. (PDF) [file pone.0063219.s007.pdf]

89.6% identity

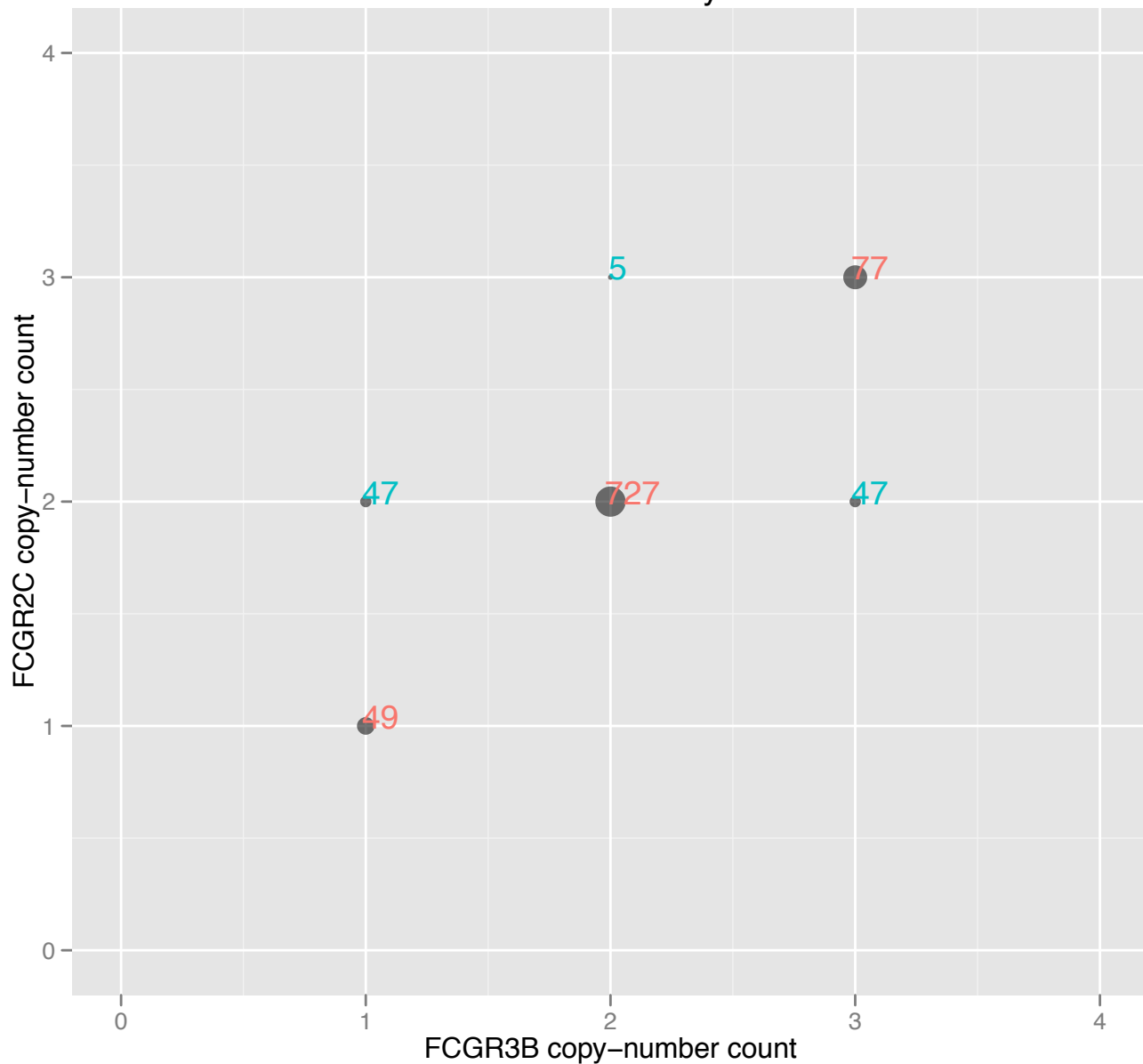

Supplement: Figure S8 — Concordance between CNVrd CN assignments at FCGR2C and FCGR3B. 0/1 is deletion, 2 is CN = 2 and 3/4 is duplication. (PDF) [file pone.0063219.s008.pdf]
