# Supplementary material for: CNVrd, a Read-Depth Algorithm for Assigning Copy-Number at the FCGR Locus: Population-Specific Tagging of Copy Number Variation at FCGR3B
Source: PLoS One. 2013 Apr 30;8(4):e63219. doi: 10.1371/journal.pone.0063219 (PMC3640002; doi:10.1371/journal.pone.0063219)
Supplement: Table S1 — (DOC) [file pone.0063219.s009.doc]

**Table S1**

| **SNPs** | **SNP (position)** | **Alleles** | **Position in duplicated region** | **Allele(s) of duplicated position** | **Start1** | **End1** | **Start2** | **End2** | **% identity** | The number of alignments | **SNP at the 2^nd^ position** | **PSV/MSV** |
| --- | --- | --- | --- | --- | --- | --- | --- | --- | --- | --- | --- | --- |
|  | 161528550 | G/A | 161609899 | G | 161528501 | 161528600 | 161609850 | 161609949 | 99.00% | 2 | No | No |
|  | 161530185 | C/T | 161611530 | C | 161530136 | 161530235 | 161611481 | 161611580 | 99.00% | 2 | Yes | MSV |
| rs34642771 | 161586285 | C/T | 168176940 | C | 161586236 | 161586335 | 168176891 | 168176990 | 94.00% | 272 | No | No |
| **rs34015117** | **161601736** | **G/A** | **161520381** | **G/A** | **161601687** | **161601786** | **161520332** | **161520421** | **98.00%** | **2** | **Yes (G/A)** | **MSV** |
|  | 161602517 | C/T | 161521154 | C | 161602468 | 161602567 | 161521105 | 161521204 | 100.00% | 2 | No | No |
| **rs115043605** | **161607874** | **C/T** | **161526524** | **T** | **161607825** | **161607924** | **161526475** | **161526574** | **99.00%** | **153** | **No** | **PSV** |
| ***rs117435514*** | ***161610869*** | ***A/G*** | ***161529521*** | ***A*** | ***161610820*** | ***161610919*** | ***161529472*** | ***161529571*** | ***99.00%*** | ***2*** | ***No*** | ***No*** |
|  | 161610920 | T/C | 161529572 | T | 161610871 | 161610970 | 161529523 | 161529622 | 99.00% | 2 | No | No |
| rs61803049 | 161612839 | A/G | 161531494 | A | 161612790 | 161612889 | 161531445 | 161531544 | 99.00% | 2 | No | No |
|  | 161616926 | C/T | 161535536 | C | 161616877 | 161616976 | 161535487 | 161535585 | 100.00% | 2 | No | No |
|  | 161617022 | C/G | 161535631 | C | 161616973 | 161617072 | 161535582 | 161535681 | 100.00% | 2 | No | No |
| rs6674499 | 161618151 | T/C | 161536758 | T | 161618102 | 161618201 | 161536709 | 161536808 | 100.00% | 2 | No | No |
