# Supplementary material for: CNVrd, a Read-Depth Algorithm for Assigning Copy-Number at the FCGR Locus: Population-Specific Tagging of Copy Number Variation at FCGR3B
Source: PLoS One. 2013 Apr 30;8(4):e63219. doi: 10.1371/journal.pone.0063219 (PMC3640002; doi:10.1371/journal.pone.0063219)
Supplement: Table S2 — Copy number of the 1000 Genomes Project samples analyzed by Hollox, CNVrd, CNVnator at FCGR3A and FCGR3B (1: deletion, 2: normal, 3: duplication). (DOC) [file pone.0063219.s010.doc]

***Table S2*** *Copy number of the 1000 Genomes Project samples analyzed by Hollox, CNVrd, CNVnator at FCGR3A and* FCGR3B (1: deletion, 2: normal, 3: duplication).

|  |  |  |  |  |  |
| --- | --- | --- | --- | --- | --- |
| Sample | Population | Hollox | CVNnator | CNVrd | Consensus |
| NA06994 | CEU | 1A:2B | 1A:2B | 1A:2B | 1A*:2B* |
| NA07000 | CEU | 2A:2B | 2A:2B | 2A:2B | 2A*:2B* |
| NA07048 | CEU | 3A:3B | 2A:2B | 2A:2B | 2A:2B |
| NA07056 | CEU | 3A:2B | 2A:2B | 2A:2B | 2A:2B* |
| NA10847 | CEU | 2A:2B | 2A:2B | 2A:2B | 2A*:2B* |
| NA10851 | CEU | 2A:3B | 3A:3B | 2A:3B | 3A:3B* |
| NA11829 | CEU | 2A:2B | 2A:2B | 2A:2B | 2A*:2B* |
| NA11830 | CEU | 2A:2B | 2A:2B | 2A:2B | 2A*:2B* |
| NA11831 | CEU | 2A:2B | 2A:2B | 2A:2B | 2A*:2B* |
| NA11992 | CEU | 2A:2B | 2A:2B | 2A:2B | 2A*:2B* |
| NA11993 | CEU | 2A:3B | 3A:3B | 2A:3B | 3A:3B* |
| NA11994 | CEU | 3A:2B | 3A:2B | 3A:2B | 3A*:2B* |
| NA11995 | CEU | 2A:2B | 2A:2B | 2A:2B | 2A*:2B* |
| NA12003 | CEU | 1A:2B | 2A:2B | 2A:2B | 2A:2B* |
| NA12004 | CEU | 3A:2B | 2A:2B | 2A:2B | 2A:2B* |
| NA12006 | CEU | 2A:2B | 2A:2B | 2A:2B | 2A*:2B* |
| NA12043 | CEU | 2A:2B | 2A:2B | 2A:2B | 2A*:2B* |
| NA12044 | CEU | 2A:2B | 2A:2B | 2A:2B | 2A*:2B* |
| NA12144 | CEU | 2A:2B | 2A:2B | 2A:2B | 2A*:2B* |
| NA12716 | CEU | 2A:2B | 2A:2B | 2A:2B | 2A*:2B* |
| NA12717 | CEU | 3A:1B | 3A:2B | 3A:2B | 3A*:2B |
| NA12750 | CEU | 1A:3B | 2A:3B | 1A:3B | 2A:3B* |
| NA12751 | CEU | 2A:2B | 2A:2B | 2A:2B | 2A*:2B* |
| NA12761 | CEU | 2A:3B | 2A:2B | 2A:2B | 2A*:2B |
| NA12763 | CEU | 2A:2B | 2A:2B | 2A:2B | 2A*:2B* |
| NA12878 | CEU | 2A:2B | 2A:2B | 2A:2B | 2A*:2B* |
| NA12891 | CEU | 2A:2B | 2A:2B | 2A:2B | 2A*:2B* |
| NA12892 | CEU | 2A:2B | 2A:2B | 2A:2B | 2A*:2B* |
| NA18501 | YRI | 2A:2B | 2A:2B | 2A:2B | 2A*:2B* |
| NA18502 | YRI | 2A:2B | 2A:2B | 2A:2B | 2A*:2B* |
| NA18504 | YRI | 3A:1B | 2A:1B | 1A:1B | 2A:1B* |
| NA18507 | YRI | 2A:2B | 2A:2B | 2A:2B | 2A*:2B* |
| NA18516 | YRI | 2A:2B | 2A:2B | 2A:2B | 2A*:2B* |
| NA18517 | YRI | 1A:2B | 1A:2B | 1A:2B | 1A*:2B* |
| NA18522 | YRI | 2A:2B | 2A:2B | 2A:2B | 2A*:2B* |
| NA18526 | CHB | 2A:3B | 2A:3B | 2A:3B | 2A*:3B* |
| NA18532 | CHB | 2A:2B | 2A:2B | 2A:2B | 2A*:2B* |
| NA18537 | CHB | 3A:3B | 2A:2B | 2A:2B | 2A:2B |
| NA18542 | CHB | 2A:2B | 2A:2B | 2A:2B | 2A*:2B* |
| NA18545 | CHB | 3A:2B | 2A:3B | 2A:3B | 2A:3B |
| NA18547 | CHB | 3A:2B | 2A:1B | 1A:1B | 2A:1B |
| NA18550 | CHB | 2A:2B | 2A:2B | 2A:2B | 2A*:2B* |
| NA18552 | CHB | 2A:3B | 2A:2B | 2A:2B | 2A*:2B |
| NA18555 | CHB | 2A:3B | 3A:3B | 2A:3B | 3A:3B* |
| NA18558 | CHB | 3A:2B | 3A:2B | 3A:3B | 3A*:2B |
| NA18561 | CHB | 2A:2B | 2A:2B | 2A:2B | 2A*:2B* |
| NA18562 | CHB | 2A:2B | 2A:2B | 2A:2B | 2A*:2B* |
| NA18564 | CHB | 1A:1B | 1A:1B | 1A:1B | 1A*:1B* |
| NA18566 | CHB | 2A:2B | 2A:2B | 2A:2B | 2A*:2B* |
| NA18570 | CHB | 2A:3B | 2A:2B | 2A:2B | 2A*:2B |
| NA18571 | CHB | 2A:3B | 2A:2B | 2A:3B | 2A*:3B |
| NA18572 | CHB | 2A:2B | 2A:2B | 2A:2B | 2A*:2B* |
| NA18573 | CHB | 2A:1B | 2A:1B | 1A:1B | 2A:1B* |
| NA18576 | CHB | 2A:2B | 2A:2B | 2A:2B | 2A*:2B* |
| NA18577 | CHB | 3A:2B | 2A:2B | 2A:2B | 2A:2B* |
| NA18579 | CHB | 2A:3B | 3A:3B | 2A:3B | 3A:3B* |
| NA18582 | CHB | 2A:1B | 2A:1B | 2A:1B | 2A*:1B* |
| NA18592 | CHB | 3A:3B | 3A:3B | 3A:3B | 3A*:3B* |
| NA18593 | CHB | 2A:2B | 2A:2B | 2A:2B | 2A*:2B* |
| NA18603 | CHB | 3A:3B | 3A:3B | 3A:3B | 3A*:3B* |
| NA18605 | CHB | 2A:3B | 3A:2B | 2A:3B | 3A:3B |
| NA18608 | CHB | 2A:2B | 2A:2B | 2A:2B | 2A*:2B* |
| NA18609 | CHB | 2A:2B | 2A:2B | 2A:2B | 2A*:2B* |
| NA18611 | CHB | 2A:2B | 2A:2B | 2A:2B | 2A*:2B* |
| NA18612 | CHB | 2A:3B | 3A:3B | 2A:3B | 3A:3B* |
| NA18620 | CHB | 2A:2B | 2A:2B | 2A:2B | 2A*:2B* |
| NA18621 | CHB | 2A:3B | 2A:2B | 2A:2B | 2A*:2B |
| NA18622 | CHB | 2A:2B | 2A:2B | 2A:2B | 2A*:2B* |
| NA18623 | CHB | 2A:2B | 2A:2B | 2A:2B | 2A*:2B* |
| NA18632 | CHB | 2A:2B | 2A:2B | 2A:2B | 2A*:2B* |
| NA18633 | CHB | 2A:3B | 3A:3B | 2A:3B | 3A:3B* |
| NA18635 | CHB | 2A:2B | 2A:2B | 2A:2B | 2A*:2B* |
| NA18636 | CHB | 3A:2B | 2A:2B | 2A:2B | 2A:2B* |
| NA18637 | CHB | 2A:2B | 2A:1B | 2A:1B | 2A*:1B |
| NA18853 | YRI | 3A:2B | 2A:1B | 1A:1B | 2A:1B |
| NA18856 | YRI | 2A:2B | 2A:2B | 2A:2B | 2A*:2B* |
| NA18858 | YRI | 2A:1B | 2A:2B | 1A:1B | 2A:1B |
| NA18861 | YRI | 2A:2B | 2A:1B | 1A:1B | 2A:1B |
| NA18870 | YRI | 3A:3B | 2A:2B | 2A:2B | 2A:2B |
| NA18871 | YRI | 2A:2B | 2A:2B | 2A:2B | 2A*:2B* |
| NA18912 | YRI | 2A:2B | 2A:2B | 1A:1B | 2A:2B |
| NA18940 | JPT | 2A:1B | 2A:2B | 2A:2B | 2A*:2B |
| NA18942 | JPT | 2A:2B | 2A:2B | 2A:2B | 2A*:2B* |
| NA18943 | JPT | 2A:2B | 2A:2B | 2A:2B | 2A*:2B* |
| NA18944 | JPT | 2A:2B | 2A:2B | 2A:2B | 2A*:2B* |
| NA18945 | JPT | 2A:2B | 2A:2B | 2A:2B | 2A*:2B* |
| NA18948 | JPT | 2A:2B | 2A:2B | 2A:2B | 2A*:2B* |
| NA18949 | JPT | 2A:1B | 2A:1B | 1A:1B | 2A:1B* |
| NA18951 | JPT | 2A:2B | 2A:2B | 2A:2B | 2A*:2B* |
| NA18952 | JPT | 2A:3B | 2A:3B | 2A:3B | 2A*:3B* |
| NA18953 | JPT | 2A:2B | 2A:2B | 2A:2B | 2A*:2B* |
| NA18956 | JPT | 2A:3B | 2A:2B | 2A:3B | 2A*:3B |
| NA18959 | JPT | 2A:2B | 2A:2B | 2A:2B | 2A*:2B* |
| NA18960 | JPT | 2A:3B | 3A:3B | 2A:3B | 3A:3B* |
| NA18961 | JPT | 2A:2B | 2A:2B | 2A:2B | 2A*:2B* |
| NA18964 | JPT | 2A:2B | 2A:2B | 2A:2B | 2A*:2B* |
| NA18965 | JPT | 2A:2B | 2A:2B | 2A:2B | 2A*:2B* |
| NA18968 | JPT | 2A:2B | 2A:2B | 2A:2B | 2A*:2B* |
| NA18971 | JPT | 3A:3B | 2A:2B | 2A:2B | 2A:2B |
| NA18973 | JPT | 2A:2B | 2A:2B | 2A:2B | 2A*:2B* |
| NA18974 | JPT | 2A:3B | 3A:3B | 3A:3B | 3A:3B* |
| NA18975 | JPT | 3A:3B | 2A:2B | 2A:2B | 2A:2B |
| NA18976 | JPT | 3A:3B | 2A:2B | 2A:2B | 2A:2B |
| NA18980 | JPT | 2A:2B | 2A:2B | 2A:2B | 2A*:2B* |
| NA18981 | JPT | 3A:3B | 2A:2B | 2A:2B | 2A:2B |
| NA18987 | JPT | 3A:3B | 3A:2B | 3A:2B | 3A*:2B |
| NA18990 | JPT | 2A:2B | 2A:2B | 2A:2B | 2A*:2B* |
| NA18999 | JPT | 2A:3B | 2A:3B | 2A:3B | 2A*:3B* |
| NA19000 | JPT | 2A:2B | 2A:2B | 2A:2B | 2A*:2B* |
| NA19003 | JPT | 2A:3B | 2A:2B | 2A:2B | 2A*:2B |
| NA19005 | JPT | 2A:2B | 2A:2B | 2A:2B | 2A*:2B* |
| NA19007 | JPT | 2A:2B | 2A:2B | 2A:2B | 2A*:2B* |
| NA19012 | JPT | 2A:2B | 2A:2B | 2A:2B | 2A*:2B* |
| NA19093 | YRI | 2A:2B | 2A:2B | 2A:2B | 2A*:2B* |
| NA19098 | YRI | 2A:2B | 2A:2B | 2A:2B | 2A*:2B* |
| NA19099 | YRI | 2A:2B | 2A:2B | 2A:2B | 2A*:2B* |
| NA19102 | YRI | 2A:1B | 2A:1B | 2A:1B | 2A*:1B* |
| NA19116 | YRI | 2A:2B | 2A:2B | 2A:2B | 2A*:2B* |
| NA19119 | YRI | 3A:3B | 2A:2B | 2A:2B | 2A:2B |
| NA19131 | YRI | 2A:3B | 2A:3B | 2A:3B | 2A*:3B* |
| NA19137 | YRI | 2A:2B | 2A:2B | 2A:2B | 2A*:2B* |
| NA19152 | YRI | 2A:2B | 2A:2B | 2A:2B | 2A*:2B* |
| NA19160 | YRI | 2A:2B | 2A:2B | 2A:2B | 2A*:2B* |
| NA19171 | YRI | 2A:2B | 2A:2B | 2A:2B | 2A*:2B* |
| NA19172 | YRI | 2A:2B | 2A:2B | 2A:2B | 2A*:2B* |
| NA19200 | YRI | 2A:2B | 2A:2B | 2A:2B | 2A*:2B* |
| NA19204 | YRI | 2A:2B | 2A:2B | 2A:2B | 2A*:2B* |
| NA19207 | YRI | 2A:2B | 2A:2B | 2A:2B | 2A*:2B* |
| NA19209 | YRI | 2A:1B | 2A:2B | 1A:1B | 2A:2B |
| NA19223 | YRI | 2A:2B | 2A:2B | 2A:2B | 2A*:2B* |
| NA19238 | YRI | 2A:2B | 2A:2B | 2A:2B | 2A*:2B* |
| NA19239 | YRI | 2A:2B | 2A:2B | 2A:2B | 2A*:2B* |
| NA19240 | YRI | 2A:2B | 2A:2B | 2A:2B | 2A*:2B* |

(*: very certain)
