# Supplementary material for: CNVrd, a Read-Depth Algorithm for Assigning Copy-Number at the FCGR Locus: Population-Specific Tagging of Copy Number Variation at FCGR3B
Source: PLoS One. 2013 Apr 30;8(4):e63219. doi: 10.1371/journal.pone.0063219 (PMC3640002; doi:10.1371/journal.pone.0063219)
Supplement: Table S3 — Correlation of SNPs with copy number in the FCGR locus (A = FCRG3A, B = FCGR3B). (DOC) [file pone.0063219.s011.doc]

**Table S3** Correlation of SNPs with copy number in the FCGR locus (A = *FCRG3A, B = FCGR3B*).

Duplication

| **Population** | **SNP** | **SNP (position)** | **A n = 2** | **B n = 5** | **AB n = 8** | **Normal n = 36** | **p.values** | **r2-Spearman** |
| --- | --- | --- | --- | --- | --- | --- | --- | --- |
| **MXL** |  |  |  |  |  |  |  |  |
|  | . | 161530185 | 0.00 | 1.00 | 1.00 | 0.03 | 6.92E-08 | 0.79 |
|  | rs117435514 | 161610869 | 0.00 | 1.00 | 1.00 | 0.03 | 6.92E-08 | 0.79 |
|  | . | 161610920 | 0.00 | 1.00 | 1.00 | 0.03 | 6.92E-08 | 0.79 |
|  | . | 161616926 | 0.00 | 1.00 | 1.00 | 0.03 | 6.92E-08 | 0.79 |
|  | . | 161617022 | 0.00 | 1.00 | 1.00 | 0.03 | 6.92E-08 | 0.79 |
|  | . | 161528550 | 0.00 | 1.00 | 1.00 | 0.06 | 5.36E-07 | 0.71 |
|  | rs34015117 | 161601736 | 0.00 | 1.00 | 0.88 | 0.03 | 1.05E-06 | 0.69 |
|  | . | 161602517 | 0.00 | 1.00 | 0.88 | 0.03 | 1.05E-06 | 0.69 |
|  | rs115043605 | 161607874 | 0.00 | 1.00 | 0.88 | 0.03 | 1.05E-06 | 0.69 |
|  | rs58783368 | 161619455 | 0.00 | 1.00 | 0.88 | 0.03 | 1.05E-06 | 0.69 |
|  | rs61803049 | 161612839 | 0.00 | 1.00 | 1.00 | 0.19 | 1.10E-04 | 0.44 |
|  | rs6674499 | 161618151 | 0.00 | 1.00 | 1.00 | 0.19 | 1.10E-04 | 0.44 |
|  | rs56159502 | 161618545 | 0.00 | 1.00 | 1.00 | 0.19 | 1.10E-04 | 0.44 |
|  | . | 161536879 | 0.50 | 0.60 | 0.63 | 0.00 | 1.45E-03 | 0.51 |
|  | rs4656324 | 161617113 | 0.00 | 0.80 | 1.00 | 0.19 | 1.45E-03 | 0.38 |
|  | rs76110710 | 161759216 | 0.50 | 1.00 | 0.88 | 0.19 | 2.39E-03 | 0.45 |
|  | . | 161609122 | 1.00 | 0.60 | 0.25 | 0.97 | 1.07E-02 | 0.33 |
|  | . | 161630959 | 1.00 | 0.20 | 0.00 | 0.72 | 1.55E-02 | 0.27 |
|  | . | 161570541 | 0.00 | 0.60 | 0.88 | 0.14 | 3.07E-02 | 0.31 |
|  | rs1806532 | 161972227 | 1.00 | 1.00 | 0.38 | 1.00 | 3.07E-02 | 0.23 |
|  | rs2490422 | 161974584 | 1.00 | 1.00 | 0.38 | 1.00 | 3.07E-02 | 0.21 |
|  | rs114193592 | 161582570 | 0.00 | 0.80 | 0.38 | 0.03 | 3.12E-02 | 0.29 |
|  | rs114835251 | 161582571 | 0.00 | 0.80 | 0.38 | 0.03 | 3.12E-02 | 0.29 |
|  | rs80145784 | 161586897 | 0.00 | 0.80 | 0.38 | 0.03 | 3.12E-02 | 0.29 |
|  | rs113947281 | 161587961 | 0.00 | 0.80 | 0.38 | 0.03 | 3.12E-02 | 0.29 |
|  | . | 161588132 | 0.00 | 0.80 | 0.38 | 0.03 | 3.12E-02 | 0.29 |
|  | rs35962658 | 161590514 | 0.00 | 0.80 | 0.38 | 0.03 | 3.12E-02 | 0.29 |
|  | . | 161593128 | 0.00 | 0.80 | 0.38 | 0.03 | 3.12E-02 | 0.29 |
|  | rs58054947 | 162817467 | 1.00 | 0.00 | 0.63 | 0.06 | 3.12E-02 | 0.25 |
|  | . | 161504244 | 0.50 | 1.00 | 0.75 | 0.22 | 3.66E-02 | 0.28 |
|  | rs10917593 | 162733109 | 1.00 | 1.00 | 0.50 | 0.19 | 3.97E-02 | 0.22 |
|  | rs10919282 | 161498069 | 0.00 | 0.60 | 0.38 | 0.00 | 4.26E-02 | 0.32 |
|  | rs6666482 | 161738478 | 1.00 | 0.00 | 0.38 | 0.81 | 4.26E-02 | 0.17 |
|  | rs7519514 | 161739190 | 1.00 | 0.00 | 0.38 | 0.81 | 4.26E-02 | 0.17 |
|  | rs905594 | 161741274 | 1.00 | 0.00 | 0.38 | 0.81 | 4.26E-02 | 0.17 |
|  | rs1847383 | 161749812 | 1.00 | 0.00 | 0.38 | 0.81 | 4.26E-02 | 0.16 |
|  | rs115094929 | 161767656 | 1.00 | 0.00 | 0.38 | 0.81 | 4.26E-02 | 0.16 |
|  | rs1006310 | 161811555 | 1.00 | 0.00 | 0.38 | 0.81 | 4.26E-02 | 0.16 |
|  | rs61802510 | 161812478 | 1.00 | 0.00 | 0.38 | 0.81 | 4.26E-02 | 0.16 |
|  | rs2257107 | 161823011 | 1.00 | 0.00 | 0.38 | 0.81 | 4.26E-02 | 0.16 |
|  | rs931778 | 161823618 | 1.00 | 0.00 | 0.38 | 0.81 | 4.26E-02 | 0.16 |
|  | rs4656336 | 161831898 | 1.00 | 0.00 | 0.38 | 0.81 | 4.26E-02 | 0.16 |
|  | rs2340720 | 161832842 | 1.00 | 0.00 | 0.38 | 0.81 | 4.26E-02 | 0.16 |
|  | rs2340721 | 161849385 | 1.00 | 0.00 | 0.38 | 0.81 | 4.26E-02 | 0.16 |
|  | rs6683830 | 161860512 | 1.00 | 0.00 | 0.38 | 0.81 | 4.26E-02 | 0.16 |
|  | rs10918137 | 161862555 | 1.00 | 0.00 | 0.38 | 0.81 | 4.26E-02 | 0.16 |
|  | rs12124509 | 161867489 | 1.00 | 0.00 | 0.38 | 0.81 | 4.26E-02 | 0.16 |
|  | rs12023612 | 162722152 | 0.50 | 1.00 | 0.25 | 0.11 | 4.26E-02 | 0.15 |
|  |  |  |  |  |  |  |  |  |
| **CHB** |  |  | n = 4 | n = 6 | n = 13 | n = 63 |  |  |
|  | rs117435514 | 161610869 | 0.00 | 1.00 | 0.85 | 0.11 | 7.76E-06 | 0.46 |
|  | rs6674499 | 161618151 | 0.00 | 1.00 | 0.77 | 0.09 | 7.76E-06 | 0.44 |
|  | rs56159502 | 161618545 | 0.00 | 1.00 | 0.77 | 0.09 | 7.76E-06 | 0.44 |
|  | . | 161562113 | 0.25 | 1.00 | 0.92 | 0.17 | 1.03E-05 | 0.43 |
|  | . | 161593128 | 0.00 | 1.00 | 0.77 | 0.11 | 2.18E-05 | 0.40 |
|  | . | 161617022 | 0.00 | 1.00 | 0.69 | 0.09 | 2.69E-05 | 0.38 |
|  | rs34642771 | 161586285 | 0.00 | 0.83 | 0.77 | 0.09 | 4.25E-05 | 0.41 |
|  | rs80145784 | 161586897 | 0.00 | 0.83 | 0.77 | 0.09 | 4.25E-05 | 0.41 |
|  | rs113947281 | 161587961 | 0.00 | 0.83 | 0.77 | 0.09 | 4.25E-05 | 0.41 |
|  | . | 161588132 | 0.00 | 0.83 | 0.77 | 0.09 | 4.25E-05 | 0.41 |
|  | . | 161602517 | 0.00 | 1.00 | 0.77 | 0.13 | 4.25E-05 | 0.36 |
|  | rs61803049 | 161612839 | 0.00 | 1.00 | 0.69 | 0.11 | 4.26E-05 | 0.34 |
|  | rs115953596 | 161557154 | 0.25 | 1.00 | 0.85 | 0.17 | 4.91E-05 | 0.38 |
|  | rs111828362 | 161557528 | 0.25 | 1.00 | 0.85 | 0.17 | 4.91E-05 | 0.38 |
|  | . | 161559571 | 0.25 | 0.83 | 0.92 | 0.17 | 4.91E-05 | 0.40 |
|  | rs78603008 | 161559720 | 0.25 | 0.83 | 0.92 | 0.17 | 4.91E-05 | 0.40 |
|  | . | 161616926 | 0.00 | 1.00 | 0.62 | 0.09 | 5.09E-05 | 0.33 |
|  | rs114193592 | 161582570 | 0.00 | 0.83 | 0.69 | 0.09 | 1.98E-04 | 0.35 |
|  | rs114835251 | 161582571 | 0.00 | 0.83 | 0.69 | 0.09 | 1.98E-04 | 0.35 |
|  | rs35962658 | 161590514 | 0.00 | 0.67 | 0.77 | 0.09 | 1.98E-04 | 0.37 |
|  | . | 161555461 | 0.25 | 0.83 | 0.85 | 0.17 | 4.88E-04 | 0.34 |
|  | rs114945036 | 161559256 | 0.25 | 0.83 | 0.85 | 0.17 | 4.88E-04 | 0.34 |
|  | rs34015117 | 161601736 | 0.00 | 0.83 | 0.69 | 0.11 | 5.10E-04 | 0.31 |
|  | . | 161609572 | 0.00 | 0.83 | 0.69 | 0.11 | 5.10E-04 | 0.31 |
|  | . | 161610920 | 0.00 | 0.83 | 0.69 | 0.11 | 5.10E-04 | 0.31 |
|  | rs58783368 | 161619455 | 0.00 | 0.67 | 0.69 | 0.09 | 1.34E-03 | 0.32 |
|  | rs115043605 | 161607874 | 0.00 | 0.83 | 0.62 | 0.11 | 2.57E-03 | 0.26 |
|  | . | 161553321 | 0.00 | 1.00 | 0.92 | 0.39 | 3.37E-03 | 0.18 |
|  | rs4656324 | 161617113 | 0.00 | 0.83 | 0.54 | 0.09 | 3.37E-03 | 0.25 |
|  | . | 161562143 | 0.25 | 1.00 | 0.92 | 0.35 | 3.92E-03 | 0.19 |
|  | rs111971126 | 161563696 | 0.25 | 1.00 | 0.92 | 0.35 | 3.92E-03 | 0.19 |
|  | rs61801819 | 161564093 | 0.25 | 1.00 | 0.92 | 0.39 | 1.62E-02 | 0.16 |
|  | rs2050886 | 161548971 | 0.75 | 0.83 | 1.00 | 0.41 | 1.73E-02 | 0.22 |
|  | . | 161549744 | 0.00 | 0.50 | 0.31 | 0.00 | 2.41E-02 | 0.23 |
|  | rs13374477 | 162710982 | 0.75 | 0.33 | 0.00 | 0.02 | 2.91E-02 | 0.04 |
|  |  |  |  |  |  |  |  |  |
| **JPT** |  |  | n = 5 | n = 4 | n = 10 | n = 54 |  |  |
|  | rs34642771 | 161586285 | 0.20 | 1.00 | 0.80 | 0.02 | 3.06E-07 | 0.60 |
|  | rs80145784 | 161586897 | 0.20 | 1.00 | 0.80 | 0.02 | 3.06E-07 | 0.60 |
|  | rs113947281 | 161587961 | 0.20 | 1.00 | 0.80 | 0.02 | 3.06E-07 | 0.60 |
|  | . | 161588132 | 0.20 | 1.00 | 0.80 | 0.02 | 3.06E-07 | 0.60 |
|  | rs35962658 | 161590514 | 0.00 | 1.00 | 0.80 | 0.02 | 3.06E-07 | 0.57 |
|  | . | 161593128 | 0.00 | 1.00 | 0.80 | 0.04 | 1.33E-06 | 0.51 |
|  | rs114193592 | 161582570 | 0.20 | 1.00 | 0.80 | 0.04 | 1.36E-06 | 0.54 |
|  | rs114835251 | 161582571 | 0.20 | 1.00 | 0.80 | 0.04 | 1.36E-06 | 0.54 |
|  | rs34015117 | 161601736 | 0.00 | 1.00 | 0.80 | 0.06 | 2.85E-06 | 0.45 |
|  | . | 161609572 | 0.00 | 1.00 | 0.80 | 0.06 | 2.85E-06 | 0.45 |
|  | rs117435514 | 161610869 | 0.00 | 1.00 | 0.80 | 0.06 | 2.85E-06 | 0.45 |
|  | rs61803049 | 161612839 | 0.00 | 1.00 | 0.80 | 0.06 | 2.85E-06 | 0.45 |
|  | . | 161528550 | 0.00 | 1.00 | 0.60 | 0.02 | 1.41E-05 | 0.43 |
|  | . | 161530185 | 0.00 | 0.75 | 0.70 | 0.02 | 2.77E-05 | 0.45 |
|  | rs115043605 | 161607874 | 0.00 | 1.00 | 0.70 | 0.06 | 2.77E-05 | 0.39 |
|  | . | 161610920 | 0.00 | 1.00 | 0.70 | 0.06 | 2.77E-05 | 0.39 |
|  | rs4656324 | 161617113 | 0.00 | 1.00 | 0.60 | 0.04 | 6.05E-05 | 0.37 |
|  | . | 161602517 | 0.00 | 1.00 | 0.70 | 0.07 | 1.05E-04 | 0.34 |
|  | . | 161616926 | 0.00 | 0.75 | 0.70 | 0.04 | 1.33E-04 | 0.39 |
|  | . | 161617022 | 0.00 | 0.75 | 0.70 | 0.04 | 1.33E-04 | 0.39 |
|  | rs6674499 | 161618151 | 0.00 | 0.75 | 0.70 | 0.04 | 1.33E-04 | 0.39 |
|  | rs56159502 | 161618545 | 0.00 | 0.75 | 0.70 | 0.04 | 1.33E-04 | 0.39 |
|  | rs115953596 | 161557154 | 0.60 | 1.00 | 0.90 | 0.22 | 4.25E-04 | 0.32 |
|  | rs111828362 | 161557528 | 0.60 | 1.00 | 0.90 | 0.22 | 4.25E-04 | 0.32 |
|  | . | 161562113 | 0.60 | 1.00 | 0.90 | 0.22 | 4.25E-04 | 0.34 |
|  | . | 161612990 | 0.00 | 0.75 | 0.40 | 0.00 | 8.41E-04 | 0.33 |
|  | rs58783368 | 161619455 | 0.00 | 0.75 | 0.60 | 0.04 | 8.77E-04 | 0.33 |
|  | rs6681076 | 161484324 | 0.00 | 0.50 | 0.50 | 0.00 | 1.50E-03 | 0.35 |
|  | . | 161549744 | 0.00 | 0.50 | 0.50 | 0.00 | 1.50E-03 | 0.35 |
|  | rs35983472 | 161575768 | 0.40 | 1.00 | 1.00 | 0.35 | 4.26E-03 | 0.17 |
|  | . | 161555461 | 0.60 | 1.00 | 0.80 | 0.22 | 5.31E-03 | 0.27 |
|  | rs114945036 | 161559256 | 0.60 | 1.00 | 0.80 | 0.22 | 5.31E-03 | 0.26 |
|  | . | 161559571 | 0.60 | 1.00 | 0.80 | 0.22 | 5.31E-03 | 0.26 |
|  | rs78603008 | 161559720 | 0.60 | 1.00 | 0.80 | 0.22 | 5.31E-03 | 0.26 |
|  | rs34820280 | 161574290 | 0.40 | 1.00 | 1.00 | 0.37 | 6.27E-03 | 0.14 |
|  | rs35989200 | 161574305 | 0.40 | 1.00 | 1.00 | 0.37 | 6.27E-03 | 0.14 |
|  | rs61802298 | 161574740 | 0.40 | 1.00 | 1.00 | 0.37 | 6.27E-03 | 0.14 |
|  | rs35835689 | 161575447 | 0.40 | 1.00 | 1.00 | 0.37 | 6.27E-03 | 0.15 |
|  | rs34472144 | 161575744 | 0.40 | 1.00 | 1.00 | 0.37 | 6.27E-03 | 0.15 |
|  | rs3843301 | 161614401 | 0.20 | 1.00 | 1.00 | 0.41 | 8.47E-03 | 0.11 |
|  | . | 161583970 | 0.40 | 1.00 | 1.00 | 0.39 | 9.94E-03 | 0.13 |
|  | . | 161618810 | 0.00 | 0.50 | 0.40 | 0.00 | 9.94E-03 | 0.29 |
|  | rs72700199 | 162324646 | 0.00 | 0.00 | 0.00 | 0.54 | 2.09E-02 | 0.22 |
|  | rs11801397 | 161497962 | 0.00 | 0.50 | 0.50 | 0.04 | 3.44E-02 | 0.23 |
|  | . | 161595608 | 0.00 | 0.50 | 0.50 | 0.04 | 3.44E-02 | 0.23 |
|  | rs115687887 | 161485099 | 0.00 | 0.75 | 0.90 | 0.35 | 4.66E-02 | 0.09 |
|  |  |  |  |  |  |  |  |  |
| **CHS** |  |  | n = 7 | n = 10 | n = 8 | n = 63 |  |  |
|  | rs117435514 | 161610869 | 0.00 | 1.00 | 0.75 | 0.05 | 1.45E-09 | 0.49 |
|  | rs61803049 | 161612839 | 0.00 | 1.00 | 0.75 | 0.05 | 1.45E-09 | 0.49 |
|  | . | 161616926 | 0.00 | 1.00 | 0.75 | 0.05 | 1.45E-09 | 0.49 |
|  | . | 161617022 | 0.00 | 1.00 | 0.75 | 0.05 | 1.45E-09 | 0.49 |
|  | rs4656324 | 161617113 | 0.00 | 1.00 | 0.75 | 0.05 | 1.45E-09 | 0.49 |
|  | rs6674499 | 161618151 | 0.00 | 1.00 | 0.75 | 0.05 | 1.45E-09 | 0.49 |
|  | rs56159502 | 161618545 | 0.00 | 1.00 | 0.75 | 0.05 | 1.45E-09 | 0.49 |
|  | rs113947281 | 161587961 | 0.00 | 0.90 | 0.75 | 0.03 | 9.55E-09 | 0.50 |
|  | rs115043605 | 161607874 | 0.00 | 0.90 | 0.75 | 0.05 | 4.98E-08 | 0.45 |
|  | . | 161610920 | 0.00 | 0.90 | 0.75 | 0.05 | 4.98E-08 | 0.45 |
|  | . | 161528550 | 0.00 | 0.90 | 0.63 | 0.03 | 6.33E-08 | 0.44 |
|  | rs34642771 | 161586285 | 0.00 | 0.90 | 0.63 | 0.03 | 6.33E-08 | 0.44 |
|  | rs80145784 | 161586897 | 0.00 | 0.90 | 0.63 | 0.03 | 6.33E-08 | 0.44 |
|  | rs35962658 | 161590514 | 0.14 | 0.90 | 0.75 | 0.05 | 6.42E-08 | 0.48 |
|  | . | 161593128 | 0.14 | 0.90 | 0.75 | 0.05 | 6.42E-08 | 0.48 |
|  | . | 161588132 | 0.00 | 0.80 | 0.75 | 0.03 | 1.19E-07 | 0.46 |
|  | . | 161609572 | 0.00 | 0.90 | 0.75 | 0.06 | 1.32E-07 | 0.41 |
|  | . | 161530185 | 0.00 | 0.90 | 0.63 | 0.05 | 2.79E-07 | 0.40 |
|  | rs34015117 | 161601736 | 0.00 | 0.90 | 0.63 | 0.05 | 2.79E-07 | 0.40 |
|  | rs58783368 | 161619455 | 0.00 | 0.80 | 0.75 | 0.05 | 5.17E-07 | 0.41 |
|  | . | 161602517 | 0.00 | 0.90 | 0.63 | 0.06 | 1.00E-06 | 0.36 |
|  | . | 161549744 | 0.14 | 0.80 | 0.50 | 0.02 | 1.10E-06 | 0.41 |
|  | rs1771572 | 161619640 | 0.00 | 0.90 | 0.88 | 0.16 | 3.45E-06 | 0.29 |
|  | rs2165088 | 161481036 | 0.14 | 0.70 | 0.75 | 0.05 | 9.54E-06 | 0.40 |
|  | rs114193592 | 161582570 | 0.00 | 0.70 | 0.63 | 0.03 | 1.25E-05 | 0.36 |
|  | rs114835251 | 161582571 | 0.00 | 0.70 | 0.63 | 0.03 | 1.25E-05 | 0.36 |
|  | rs115953596 | 161557154 | 0.57 | 0.90 | 0.88 | 0.19 | 2.89E-05 | 0.33 |
|  | rs111828362 | 161557528 | 0.57 | 0.90 | 0.88 | 0.19 | 2.89E-05 | 0.33 |
|  | rs78603008 | 161559720 | 0.43 | 0.90 | 0.88 | 0.19 | 5.34E-05 | 0.30 |
|  | rs11811241 | 161493580 | 0.43 | 0.80 | 1.00 | 0.21 | 5.68E-05 | 0.36 |
|  | rs76016754 | 161561156 | 0.00 | 0.50 | 0.63 | 0.02 | 1.07E-04 | 0.34 |
|  | . | 161555461 | 0.57 | 0.80 | 0.88 | 0.17 | 1.17E-04 | 0.33 |
|  | rs452673 | 161506025 | 0.43 | 0.80 | 1.00 | 0.22 | 1.17E-04 | 0.34 |
|  | rs114945036 | 161559256 | 0.57 | 0.90 | 0.75 | 0.19 | 2.01E-04 | 0.28 |
|  | . | 161559571 | 0.43 | 0.90 | 0.75 | 0.19 | 4.35E-04 | 0.26 |
|  | rs6681076 | 161484324 | 0.00 | 0.40 | 0.63 | 0.02 | 4.94E-04 | 0.31 |
|  | . | 161618810 | 0.00 | 0.50 | 0.50 | 0.02 | 5.75E-04 | 0.29 |
|  | rs2499429 | 161497585 | 0.00 | 0.70 | 0.75 | 0.13 | 7.29E-04 | 0.22 |
|  | . | 161612990 | 0.00 | 0.40 | 0.38 | 0.00 | 2.48E-03 | 0.26 |
|  | rs4656314 | 161517721 | 0.00 | 0.50 | 0.38 | 0.02 | 3.13E-03 | 0.24 |
|  | . | 161518172 | 0.14 | 0.80 | 0.88 | 0.25 | 5.55E-03 | 0.18 |
|  | . | 161553321 | 0.14 | 0.80 | 0.88 | 0.25 | 5.55E-03 | 0.17 |
|  | . | 161562113 | 0.57 | 0.90 | 0.75 | 0.27 | 9.35E-03 | 0.20 |
|  | . | 161595608 | 0.00 | 0.30 | 0.38 | 0.00 | 1.45E-02 | 0.23 |
|  | rs75875385 | 161463308 | 0.00 | 0.40 | 0.50 | 0.03 | 1.47E-02 | 0.21 |
|  | . | 161614955 | 0.00 | 0.40 | 0.50 | 0.03 | 1.47E-02 | 0.21 |
|  | . | 161565981 | 0.00 | 0.40 | 0.00 | 0.00 | 2.06E-02 | 0.11 |
|  | . | 161514197 | 0.29 | 0.70 | 0.88 | 0.24 | 3.14E-02 | 0.17 |
|  | rs4657093 | 161693003 | 0.00 | 0.60 | 0.75 | 0.19 | 3.58E-02 | 0.12 |
|  | rs61801819 | 161564093 | 0.57 | 0.90 | 0.88 | 0.35 | 4.54E-02 | 0.15 |

Deletion

| **Population** | **SNP** | **SNP (position)** | **Normal n = 54** | **B n = 9** | **p.values** | **r2-Spearman** |
| --- | --- | --- | --- | --- | --- | --- |
| LWK | rs12076636 | 161498624 | 0.00 | 0.78 | 8.18E-04 | 0.75 |
|  | . | 161489416 | 0.02 | 0.78 | 2.11E-03 | 0.64 |
|  | rs115041585 | 161501963 | 0.02 | 0.78 | 2.11E-03 | 0.64 |
|  | rs6694457 | 161487881 | 0.04 | 0.78 | 3.95E-03 | 0.55 |
|  | . | 161491042 | 0.04 | 0.78 | 3.95E-03 | 0.55 |
|  | rs41297650 | 161493618 | 0.04 | 0.78 | 3.95E-03 | 0.55 |
|  | rs114558763 | 161510125 | 0.04 | 0.78 | 3.95E-03 | 0.55 |
|  | rs60056757 | 161467730 | 0.00 | 0.56 | 2.50E-02 | 0.52 |
|  | rs114811483 | 161651232 | 0.00 | 0.56 | 2.50E-02 | 0.52 |
|  | . | 161523201 | 0.04 | 0.67 | 3.97E-02 | 0.44 |
|  |  |  |  |  |  |  |
| TSI | SNP (rs) | SNP (position) | n = 78 | B n = 10 | p.values | r2-Spearman |
|  | rs61802308 | 161578935 | 0.81 | 0.10 | 7.45E-03 | 0.16 |
|  | rs41299282 | 161579084 | 0.79 | 0.10 | 7.45E-03 | 0.15 |
|  | rs2002405 | 161583107 | 0.96 | 0.40 | 7.45E-03 | 0.05 |
|  | . | 161583970 | 0.83 | 0.10 | 7.45E-03 | 0.17 |
|  | rs34772855 | 161585297 | 0.96 | 0.40 | 7.45E-03 | 0.05 |
|  | rs34107302 | 161585406 | 0.96 | 0.40 | 7.45E-03 | 0.05 |
|  | rs35544618 | 161585456 | 0.96 | 0.40 | 7.45E-03 | 0.05 |
|  | rs36021445 | 161585647 | 0.96 | 0.40 | 7.45E-03 | 0.05 |
|  | rs35112428 | 161585848 | 0.96 | 0.40 | 7.45E-03 | 0.05 |
|  | rs35918824 | 161586027 | 0.96 | 0.40 | 7.45E-03 | 0.05 |
|  | rs71519216 | 161586377 | 0.96 | 0.40 | 7.45E-03 | 0.05 |
|  | rs61802323 | 161587821 | 0.96 | 0.40 | 7.45E-03 | 0.05 |
|  | rs61802325 | 161588097 | 0.96 | 0.40 | 7.45E-03 | 0.05 |
|  | . | 161588257 | 0.96 | 0.40 | 7.45E-03 | 0.05 |
|  | rs61803001 | 161592959 | 0.96 | 0.40 | 7.45E-03 | 0.05 |
|  | rs61803007 | 161594100 | 0.96 | 0.40 | 7.45E-03 | 0.06 |
|  | rs2290832 | 161596704 | 0.96 | 0.40 | 7.45E-03 | 0.06 |
|  | rs61803015 | 161597987 | 0.96 | 0.40 | 7.45E-03 | 0.06 |
|  | rs34612244 | 161598278 | 0.96 | 0.40 | 7.45E-03 | 0.06 |
|  | . | 161600345 | 0.96 | 0.40 | 7.45E-03 | 0.05 |
|  | rs3883934 | 161600832 | 0.96 | 0.40 | 7.45E-03 | 0.06 |
|  | rs10799853 | 161606589 | 0.96 | 0.40 | 7.45E-03 | 0.08 |
|  | rs7518642 | 161607355 | 0.96 | 0.40 | 7.45E-03 | 0.06 |
|  | rs56101643 | 161608201 | 0.96 | 0.40 | 7.45E-03 | 0.06 |
|  | rs61144011 | 161608214 | 0.96 | 0.40 | 7.45E-03 | 0.06 |
|  | rs4047522 | 161608256 | 0.96 | 0.40 | 7.45E-03 | 0.06 |
|  | rs4047523 | 161608317 | 0.96 | 0.40 | 7.45E-03 | 0.05 |
|  | rs58670133 | 161608323 | 0.96 | 0.40 | 7.45E-03 | 0.05 |
|  | rs2878011 | 161612476 | 0.96 | 0.40 | 7.45E-03 | 0.06 |
|  | rs60751059 | 161612666 | 0.96 | 0.40 | 7.45E-03 | 0.06 |
|  | rs3843301 | 161614401 | 0.81 | 0.10 | 7.45E-03 | 0.15 |
|  | rs55970405 | 161617820 | 0.69 | 0.00 | 7.45E-03 | 0.17 |
|  | . | 161626481 | 0.68 | 0.00 | 1.00E-02 | 0.17 |
|  | . | 161541517 | 0.67 | 0.00 | 1.28E-02 | 0.17 |
|  | . | 161626116 | 0.67 | 0.00 | 1.28E-02 | 0.17 |
|  | . | 161626153 | 0.67 | 0.00 | 1.28E-02 | 0.16 |
|  | . | 161611747 | 0.95 | 0.40 | 1.47E-02 | 0.08 |
|  | rs7540959 | 161612229 | 0.95 | 0.40 | 1.47E-02 | 0.06 |
|  | rs80076517 | 161542027 | 0.65 | 0.00 | 1.53E-02 | 0.17 |
|  | . | 161545154 | 0.65 | 0.00 | 1.53E-02 | 0.17 |
|  | rs61804163 | 161622861 | 0.65 | 0.00 | 1.53E-02 | 0.15 |
|  | . | 161625832 | 0.64 | 0.00 | 1.94E-02 | 0.15 |
|  | rs78217950 | 161625877 | 0.64 | 0.00 | 1.94E-02 | 0.15 |
|  | rs61804171 | 161625964 | 0.64 | 0.00 | 1.94E-02 | 0.15 |
|  | . | 161544124 | 0.63 | 0.00 | 2.50E-02 | 0.16 |
|  | rs4102485 | 161606635 | 0.06 | 0.60 | 2.50E-02 | 0.25 |
|  | rs61803040 | 161612410 | 0.94 | 0.40 | 2.50E-02 | 0.03 |
|  | . | 161584357 | 0.92 | 0.40 | 4.58E-02 | 0.16 |
|  | rs76714703 | 161599693 | 0.92 | 0.40 | 4.58E-02 | 0.04 |
