# Supplementary material for: CNVrd, a Read-Depth Algorithm for Assigning Copy-Number at the FCGR Locus: Population-Specific Tagging of Copy Number Variation at FCGR3B
Source: PLoS One. 2013 Apr 30;8(4):e63219. doi: 10.1371/journal.pone.0063219 (PMC3640002; doi:10.1371/journal.pone.0063219)
Supplement: Table S4 — Correlation of duplication at FCGR3B with SNPs in data sets downloaded from 1000 Genomes in September 2012. (DOC) [file pone.0063219.s012.doc]

**Table S4** Correlation of duplication at *FCGR3B* with SNPs in data sets downloaded from 1000 Genomes in September 2012.

| **Population** | | **SNP (ID)** | | **SNP (position)** | | **Normal n = 67** | | **A n = 5** | | **B n = 4** | | **AB n = 0** | | **p.values** | | **r2-Spearman** | |
| --- | --- | --- | --- | --- | --- | --- | --- | --- | --- | --- | --- | --- | --- | --- | --- | --- | --- |
| CDX | | rs117435514 | | 161610869 | | 0 | | 0 | | 0.75 | | 0 | | 6.29E-02 | | 0.35 | |
|  | | rs34642771 | | 161586285 | | 0 | | 0 | | 0.25 | | 0 | | 9.65E-01 | | 0.11 | |
|  | | rs61803049 | | 161612839 | | 0.01 | | 0 | | 0.75 | | 0 | | 1.46E-01 | | 0.25 | |
|  | | rs6674499 | | 161618151 | | 0.01 | | 0 | | 0.25 | | 0 | | 1.00E+00 | | 0.04 | |
|  |  | |  | |  | |  | |  | |  | |  | |  | |  |
|  | |  | |  | | n = 51 | | n = 2 | | n = 12 | | n = 0 | |  | |  | |
| GIH | | rs61803049 | | 161612839 | | 0.08 | | 0 | | 0 | | 0 | | 1.00E+00 | | 0.02 | |
|  | | rs6674499 | | 161618151 | | 0.06 | | 0 | | 0 | | 0 | | 1.00E+00 | | 0.01 | |
|  |  | |  | |  | |  | |  | |  | |  | |  | |  |
|  | |  | |  | | n = 56 | | A n = 5 | | B n = 4 | | AB n = 0 | |  | |  | |
| IBS | | rs34642771 | | 161586285 | | 0.02 | | 0 | | 0 | | 0 | | 1.00E+00 | | 0 | |
|  | | rs117435514 | | 161610869 | | 0.02 | | 0 | | 0 | | 0 | | 1.00E+00 | | 0 | |
|  | | rs61803049 | | 161612839 | | 0.18 | | 0.2 | | 0.5 | | 0 | | 1.00E+00 | | 0.02 | |
|  | | rs6674499 | | 161618151 | | 0.14 | | 0 | | 0.5 | | 0 | | 1.00E+00 | | 0.01 | |
|  |  | |  | |  | |  | |  | |  | |  | |  | |  |
|  | |  | |  | | Normal n = 58 | | A n = 0 | | B n = 6 | | AB n = 2 | |  | |  | |
| KHV | | rs61803049 | | 161612839 | | 0.03 | | 0 | | 0.83 | | 0 | | 1.02E-02 | | 0.36 | |
|  | | rs34642771 | | 161586285 | | 0.02 | | 0 | | 0.67 | | 0 | | 6.28E-02 | | 0.33 | |
|  | | rs6674499 | | 161618151 | | 0.02 | | 0 | | 0.67 | | 0 | | 6.28E-02 | | 0.33 | |
|  | | rs117435514 | | 161610869 | | 0.03 | | 0 | | 0.5 | | 0 | | 7.98E-01 | | 0.16 | |
|  |  | |  | |  | |  | |  | |  | |  | |  | |  |
|  | |  | |  | | Normal n = 26 | | A n = 0 | | B n = 19 | | AB n = 2 | |  | |  | |
| PEL | | rs61803049 | | 161612839 | | 0.19 | | 0 | | 1 | | 1 | | 4.50E-05 | | 0.63 | |
|  | | rs34642771 | | 161586285 | | 0.12 | | 0 | | 0.68 | | 0.5 | | 2.25E-02 | | 0.3 | |
|  | | rs6674499 | | 161618151 | | 0.12 | | 0 | | 0.63 | | 0.5 | | 6.64E-02 | | 0.26 | |
|  | | rs117435514 | | 161610869 | | 0.15 | | 0 | | 0.58 | | 1 | | 7.85E-02 | | 0.25 | |
|  |  | |  | |  | |  | |  | |  | |  | |  | |  |
|  | |  | |  | | Normal n = 43 | | A n = 0 | | B n = 3 | | AB n = 0 | |  | |  | |
| ACB | | rs34642771 | | 161586285 | | 0.07 | | 0 | | 0 | | 0 | | 1.00E+00 | | 0 | |
|  | | rs117435514 | | 161610869 | | 0.05 | | 0 | | 0 | | 0 | | 1.00E+00 | | 0 | |
|  | | rs61803049 | | 161612839 | | 0.05 | | 0 | | 0 | | 0 | | 1.00E+00 | | 0 | |
|  | | rs6674499 | | 161618151 | | 0.05 | | 0 | | 0 | | 0 | | 1.00E+00 | | 0 | |
