# Supplementary material for: CNVrd, a Read-Depth Algorithm for Assigning Copy-Number at the FCGR Locus: Population-Specific Tagging of Copy Number Variation at FCGR3B
Source: PLoS One. 2013 Apr 30;8(4):e63219. doi: 10.1371/journal.pone.0063219 (PMC3640002; doi:10.1371/journal.pone.0063219)
Supplement: Table S5 — Standardized ratios of observed and expected reads at MXL, CHB, CHS and JPT samples called CN = 2 at FCGR3A and CN = 3 at FCGR3B. (DOC) [file pone.0063219.s013.doc]

**Table S5** Standardized ratios of observed and expected reads at MXL, CHB, CHS and JPT samples called CN=2 at *FCGR3A* and CN=3 at *FCGR3B.*

| **Samples** | **Ratios at FCGR3A** | **Ratios at FCGR3B** | **Z-score at FCGR3A** | **Z-core at FCGR3B** |
| --- | --- | --- | --- | --- |
| HG00475 | 1.07 | 1.14 | -0.38 | 1.18 |
| HG00537 | 1.18 | 1.40 | 0.27 | 2.36 |
| HG00542 | 1.44 | 1.62 | 1.91 | 3.37 |
| HG00613 | 1.15 | 1.32 | 0.10 | 1.99 |
| HG00614 | 1.19 | 1.20 | 0.32 | 1.45 |
| HG00619 | 1.35 | 1.32 | 1.33 | 1.99 |
| HG00650 | 1.28 | 1.18 | 0.87 | 1.37 |
| HG00654 | 1.03 | 1.25 | -0.63 | 1.66 |
| HG00704 | 1.25 | 1.18 | 0.73 | 1.33 |
| HG00705 | 1.33 | 1.18 | 1.20 | 1.34 |
| NA18526 | 1.11 | 1.22 | -0.16 | 1.56 |
| NA18545 | 1.12 | 1.25 | -0.10 | 1.69 |
| NA18571 | 1.14 | 1.11 | 0.01 | 1.04 |
| NA18602 | 1.16 | 1.35 | 0.17 | 2.12 |
| NA18618 | 1.22 | 1.17 | 0.55 | 1.32 |
| NA18634 | 1.24 | 1.31 | 0.66 | 1.94 |
| NA18952 | 1.23 | 1.16 | 0.60 | 1.26 |
| NA18956 | 1.20 | 1.12 | 0.43 | 1.06 |
| NA18977 | 1.37 | 1.45 | 1.44 | 2.61 |
| NA18999 | 1.33 | 1.30 | 1.23 | 1.90 |
| NA19651 | 1.17 | 1.21 | 0.19 | 1.48 |
| NA19717 | 1.11 | 1.12 | -0.14 | 1.06 |
| NA19731 | 1.11 | 1.28 | -0.13 | 1.80 |
| NA19749 | 1.28 | 1.25 | 0.87 | 1.65 |
| NA19756 | 1.27 | 1.24 | 0.84 | 1.65 |
