# Supplementary material for: CNVrd, a Read-Depth Algorithm for Assigning Copy-Number at the FCGR Locus: Population-Specific Tagging of Copy Number Variation at FCGR3B
Source: PLoS One. 2013 Apr 30;8(4):e63219. doi: 10.1371/journal.pone.0063219 (PMC3640002; doi:10.1371/journal.pone.0063219)
Supplement: Methods S1 — (DOC) [file pone.0063219.s014.doc]

**SUPPLEMENTAL MATERIAL**

**CNVrd, a read-depth algorithm for assigning copy-number at the FCGR locus: Population-specific tagging of copy number variation at *FCGR3B***

Hoang Tan Nguyen^1,2^, Tony R Merriman^1^*, Michael A Black^1^*

Departments of ^1^Biochemistry, and ^2^Mathematics and Statistics, University of Otago, Box 56, Dunedin, New Zealand

*Corresponding author: email, tony.merriman@otago.ac.nz

**Supplemental Methods**

To verify whether SNPs could be paralogous sequence variations (PSVs) or multi-site sequence variations (MSVs) we chose 12 of the top-ranked CN tagSNPs. For each SNP a FASTA region (100bp) flanking both sides of the SNP of the reference genome was obtained followed by the use of BLAT to align the FASTA region back to the reference genome and generally obtained two hits having > =98% identity. Data from rs34642771 were not used owing to >200 hits of low identity. Two positions were checked - the annotated position of the SNP and the second position on the duplicated region aligned by BLAT (Table S1). If the reference bases of the two positions were different and one base was the same as the minor allele of the other base, it was likely to be a PSV (rs115053605). If there were SNPs at both positions, it was more likely to be a MSV (rs34015117).

**We used the Affymetrix Axiom data set (**<http://www.affymetrix.com/support/technical/sample_data/axiom_db/axiomdb_data.affx>**) containing SNP rs6674499 and the genotypes determined by Hollox et al [1] to further validate tag SNPs. In the 43 CHB and 45 JPT samples genotyped by Affymetrix Axiom and Hollox et al [1], the statistics for tagging of duplication at *FCGR3B* by *rs6674499* were *P*=0.006; r^2^=0.21 and *P*=0.003; r^2^=0.27 for CHB (n=40 samples) and JPT (n=39), respectively, compared to *P*=1.0; r^2^=0.00 and *P*=0.40, r^2^=0.01 for CEU (n=78) and YRI (n=69), respectively.**

**References**

1.Hollox EJ, Detering JC, Dehnugara T (2009) An integrated approach for measuring copy number variation at the FCGR3 (CD16) locus. Hum Mutation 30: 477-484.
